# *Lonicera caerulea* Berry Polyphenols Extract Alleviates Exercise Fatigue in Mice by Reducing Oxidative Stress, Inflammation, Skeletal Muscle Cell Apoptosis, and by Increasing Cell Proliferation

**DOI:** 10.3389/fnut.2022.853225

**Published:** 2022-03-09

**Authors:** Suwen Liu, Fanna Meng, Dong Zhang, Donglin Shi, Junyi Zhou, Shuo Guo, Xuedong Chang

**Affiliations:** ^1^College of Food Science and Technology, Hebei Normal University of Science and Technology, Qinhuangdao, China; ^2^Hebei Yanshan Special Industrial Technology Research Institute, Qinhuangdao, China; ^3^Division of Sports Science and Physical Education, Tsinghua University, Beijing, China; ^4^Department of Physical Education, Hebei Sport University, Shijiazhuang, China; ^5^Research Center of Sports Science, Hebei Institute of Sports Science, Shijiazhuang, China

**Keywords:** polyphenols, exercise fatigue, oxidative stress, skeletal muscle, immunoprecipitation techniques

## Abstract

Exercise fatigue can exert deleterious effects on the body. This study evaluated the effects and mechanisms by which *Lonicera caerulea* berry polyphenols extract (LCBP) improved the treadmill endurance of mice. Comparison was performed between the effects at 25°C and low temperatures (-5°C). Energy storage, product metabolism, and other biochemical indices were determined using vitamin C (VC) as a positive control. Co-immunoprecipitation was performed to detect the interaction between different proteins. Dietary supplementation with LCBP significantly prolonged the exhaustion time during treadmill exercise by 20.4% (25 °C) and 27.4% (-5 °C). LCBP significantly regulated the expression of antioxidant and inflammatory proteins, Bcl-2 /Bax apoptosis proteins, and the PKCα -NOx2 / Nox4 pathway proteins, and activated the expression of AMPK-PGC1α -NRF1-TFAM proteins in skeletal muscle mitochondria. The gene and protein expression of miRNA-133a/IGF-1/PI3K/Akt/mTOR in skeletal muscle cells was also activated. Molecular docking confirmed that the main components of LCBP such as cyanidin-3-glucoside, catechin, and chlorogenic acid, have strong binding affinity toward AMPKα. LCBP alleviates exercise fatigue in mice by reducing oxidative stress, inflammation, and apoptosis of skeletal muscle cells, enhances mitochondrial biosynthesis and cell proliferation, reduces fatigue, and enhances performance. These effects are also significant in a low-temperature environment (Graphical Abstract). Consequently, these results provide novel insights into the anti- fatigue roles of LCBP in exercise fatigue.

## Graphical Abstract

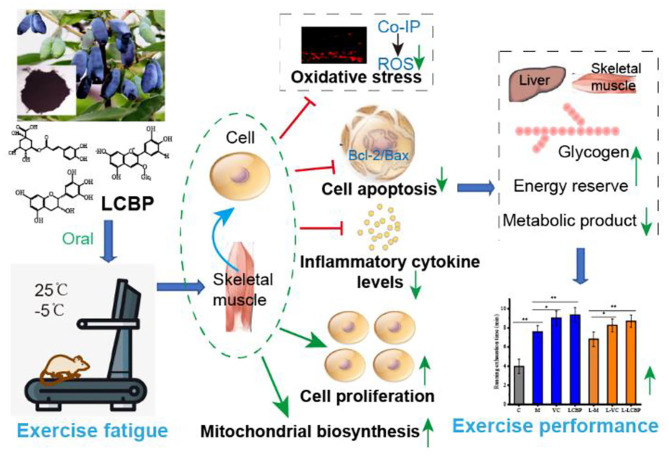

## Introduction

Exercise fatigue is a physiological phenomenon of temporary decline or loss of working ability that is caused by overloading over long periods. This is partly attributed to the accumulation of lactic acid (LA), ammonia, and blood urea nitrogen (BUN) in serum and muscle (1). Athletes who engage in competitive sports or professional fitness are subjected to intensive sports training every day, and this can easily lead to sports fatigue. Fatigue exerts a harmful impact on physical and mental health, as well as performance (2). High-intensity exercise promotes the consumption of energy sources, such as those present in the liver and muscle, and the accumulation of metabolites. Additionally, exercise fatigue can lead to the accumulation of free radicals, causing muscle injury and muscle fatigue by inducing lipid peroxidation and damage to the antioxidant defense system (3). It is therefore particularly important that exercise fatigue is delayed or eliminated to improve the effects of training and exercise.

The existing research is focused on the exploration of health supplements from natural sources that demonstrate potential in improving physical performance and in mitigating the effects of fatigue while accelerating the elimination of fatigue-related metabolites. Studies have shown that dietary bioactive ingredients can be used to effectively relieve exercise fatigue and to improve the symptoms that are associated with fatigue (4). Beef extract (5), ginseng extract (6), and conifer extract (7) reportedly exert beneficial effects on fatigue in animal swimming models. Plant polyphenols have been widely studied because of their diverse functions, wide sources, high specificity, and rare side effects, and fruit polyphenols are generally favored by consumers. Fruit polyphenols are structurally characterized by the presence of two or more hydroxyl groups attached to one or more benzene rings, providing both flavor and color characteristics of fruits and vegetables. A wide range of biological functions have been associated with fruit polyphenols derived from several different sources, including anti-aging, anti-cardiovascular disease, anti-radiation damage, and anti-diabetes activity (8). In recent years, several studies have reported the anti-fatigue potential of fruit polyphenols that demonstrate functions by reducing the accumulation of free radicals, leading to a deceleration in the rapid decline in exercise ability (9). Acute supplementation with 300 mg polyphenols 1 to 2 h before exercise can help improve performance by increasing the exercise capacity and/or endurance through the action of antioxidants. Daily polyphenol supplementation (>1,000 mg), both before and for 3 days or more after exercise, promotes recovery from muscle injury through antioxidant and anti-inflammatory mechanisms (10). Dark tea extracts stimulate the activation of the Akt/mTOR pathway to improve endurance and protein synthesis in the loaded soleus muscle (11). Resveratrol can significantly increase aerobic capacity by increasing running time and oxygen consumption in muscle fibers (12). Prunus mume vinegar, which contains substantial amounts of phenolic acid, has been found to promote the recovery of rats presenting with high-intensity exercise fatigue (13). Consumption of lychee extract for 30 days can increase the time of running to exhaustion at 80% of the maximum heart rate (14). The consumption of blackcurrant powder for 7 consecutive days was found to improve performance during timed cycling tests (15) and high-intensity interval running distance (16). Therefore, the development of fruit polyphenols with significant anti-exercise fatigue properties is of immense significance for those that are affected by long-term exercise fatigue.

Lonicera caerulea berry (*Lonicera edulis*) is a perennial deciduous shrub of the genus Lonicera in the family Caprifoliaceae, presenting with sweet and sour berries, and bright, dark rose-colored juice. Previous studies have confirmed that the Lonicera caerulea berry is rich in polyphenols, components that exhibit significant antioxidant and anti-inflammatory effects (17–20). However, few studies have investigated the effect exerted by LCBP on recovery following long-term exercise; further research that considers the use of this dietary component as a functional factor in reducing exercise fatigue is warranted. The purpose of this study was to investigate the anti-fatigue properties of LCBP in a long-term exercise-fatigued mouse model and to examine the anti-fatigue mechanism of LCBP associated with the inhibition of oxidative stress and inflammation, improvements in mitochondrial function, and the promotion of skeletal muscle proliferation. This study provides a theoretical basis for the application of LCBP to relieve exercise fatigue using specific food components.

## Materials and Methods

### Sample Preparation

Lonicera caerulea berries were collected from Baishan city in Jilin Province, China. The method used to extract the polyphenols was based on that used in our previous study (19). Briefly, 100 g of fruit was mashed and 70% acidified ethanol (0.1% hydrochloric acid) was added at a ratio of 1:10. The mixture was then stirred at 30°C for 1 h, following which extraction was performed under dark conditions and the extract was filtered. Rotary evaporators were used (N1100, Tokyo, Japan) at 40°C to avoid light concentration until the entire ethanol residue was removed. The filtrate was then purified using an AB-8 macroporous resin (Sigma), following which elution was performed using 70% acidified ethanol and the eluate was concentrated in a rotary vacuum at 40°C, after which it was maintained under freezing conditions overnight at −20°C and freeze-dried at −40°C (LGJ-S30, Beijing Sihuan Technology Co., LTD., China). The resulting powder was loaded into a brown tube and stored at −20°C. HPLC-DAD-ESI-MS/MS (Ultimate 3000, Thermo LTQXL, USA) was used to quantitatively analyze the composition and content of polyphenols.

### Animals Studied and Treatments Administered

Male BALB/c mice (age: 3 months) were selected for the study. The mice were housed under conditions of 12 h in white light and 12 h in darkness at 22 ± 1°C, and at humidity levels of 45–55%. Both food and water were freely available. All experimental procedures that included animals were performed in accordance with the guidelines provided by the Animal Care Committee and approved by the Ethics Committee of Hebei Normal University of Science and Technology. The animals were provided by Beijing HFK Biotechnology Co., Ltd. (License No. SCXK (Jing) 2020-0004). Reference for the establishment of the fatigue motion model was obtained from the literature and was modified (21). The mice were randomly divided into seven groups of ten individuals. Mice in the control group were fed conventionally without exercise and were provided with the same amount of normal saline intragastrically at the same time as the mice in the other groups received supplementation. The groups were subjected to exercise for 45 min/day, 6 days a week on a platform with a slope of 0 at 25°C or at −5°C for 6 weeks. Adaptive training during the first week consisted of subjecting the mice to exercise at an initial speed of 11 m/min, which was increased by 1 m/min each day. The speed was increased by 1 m/min per week from the second week onwards. The model groups (M and L-M) received the same amount of normal saline as the control group 1 h before exercise. The VC groups (VC at 25°C and L-VC at −5°C) were subjected to exercise using the same approach adopted for the model group for 6 weeks and were given 60 mg/kg body weight VC (A103537, Aladdin, China) intragastrically 1 h before subjection to exercise; those in the LCBP groups (LCBP at 25°C and L-LCBP at −5°C) were given 250 mg/kg body weight polyphenol intragastrically 1 h before subjection to exercise. The mice were sacrificed by administering an overdose of sodium pentobarbital narcosis after 6 weeks, and blood samples were obtained for investigation. Skeletal muscle (gastrocnemius of the hind leg) and visceral organs were collected and fixed with 4% paraformaldehyde. The organs were frozen in liquid nitrogen and stored at −70°C in an ultra-low temperature refrigerator (DW HL-668, Zhongcomerin, Anhui, China) for subsequent experimental analysis.

### Treadmill Fatigue Test

The mice were placed on a treadmill for 30 min at the end of the 6-week experiment and the speed was increased from 15 to 40 m/min over 3 min. The running time was monitored until, on three consecutive occasions, the mice failed to pace themselves with the increase in speed and could not continue with the exercise routine. The total running time was recorded immediately (22).

### Physical and Chemical Indices

Physiological and biochemical indicators were detected using kits that were used according to the manufacturer's instructions. The CK and LA levels in the serum and skeletal muscle of the mice were detected using an ELISA kit (A032, A019, Nanjing Jianceng Biology, China). The serum levels of BUN, lactate dehydrogenase activity (LDH) (WLA120, WLA072, Wanleibio, China), and Ca^2+^ (C004, Nanjing Jianceng Biology, China) were detected using a kit that could also be used to detect the hemoglobin content (C021, Nanjing Jiancheng Biology, China), glycogen levels in the liver and the skeletal muscle (Solarbio, BC0340, China), serum and skeletal muscle catalase activity (CAT) (A007, Nanjing Jiangjian Biology, China), total antioxidant capacity (TOC) (BC1310, Solarbio, China), glutathione peroxidase activity (GSH-PX), superoxide dismutase (SOD) activity, and malondialdehyde (MDA) content (WLA107, WLA110, WLA048, Wanleibio). The ATP and NO levels in the skeletal muscle were measured using an ELISA kit (S0026, Biyuntian, China; A013, Nanjing Jiancheng, China) (23), which was also used to detect the serum TNFα and IL-6 levels (WLE05; WLE04, Wanleibio, China) (24) and the VEGFA levels in skeletal muscle (WLE10, Wanleibio, China). The kit that was used to determine BCA protein concentration was used in Wanleibio, WLA004, China.

### DHE Staining

The skeletal muscle was frozen in the form of slices (frozen slicer, CM1860, Leica, Germany), which were subjected to washing steps using PBS and staining procedures with 10 μM DHE (DHE Kit, S0063, Biyuntian, China), followed by incubation at 37°C for 30 min; washing steps were conducted again with PBS, and the specimens were sealed using an anti-fluorescence quenching agent. The ROS levels were then observed and determined under a fluorescence microscope (DP73, Olympus, Japan) (200x).

### Hematoxylin and Eosin Staining

The skeletal muscle tissue was fixed in 4% paraformaldehyde and the sections were embedded in paraffin. The 5 μm-thick sections were then subjected to HE staining (H8070, Solarbio, China) using water, transparent, and neutral gum seals. Morphological changes in the skeletal muscle tissue were observed and recorded under a light microscope (DP73, Olympus, Japan) (200×).

### Immunofluorescence Double Standard Test

Skeletal muscle tissue samples were fixed in 4% paraformaldehyde and embedded in paraffin to obtain sections that were 5 μm-thick. The primary antibodies Nox4 (1:50) and Nox2 (1:100) were used, with FITC-labeled goat anti-rabbit IgG used as the secondary antibody. The primary antibody was PKCα (1:200), and Cy3-labeled goat anti-mouse IgG was secondary antibody. After reactions with the antibody were subjected to incubation, the fluorescent secondary antibody was added and both samples were diluted with PBS (1:200) followed by incubation for 90 min at 25°C. The sections were removed and DAPI (D106471-5 mg Aladdin, China) was added to completely cover the tissue and to restore the nuclei. A drop of the anti-fluorescence quenching agent (S2100, Solarbio, China) was added to a rubber head dropper and was used to cover the glass seal. The staining pattern and intensity was then observed under a fluorescence microscope (400 × magnification).

### Real-Time PCR

Total RNA extraction from 100 mg of skeletal muscle was performed using the TRIzol Kit (RP1001, BioTeke, Beijing, China) in accordance with the manufacturer's instructions. The frozen tissues were ground in liquid nitrogen at −70°C, and TRIzol, chloroform, and isopropyl alcohol were added. The samples were then centrifuged, the RNA concentration was quantified at an optical density of 260 nm (NANO 2000, Thermo, USA), and the samples were stored in a refrigerator at −80°C. Samples of cDNA were reverse-transcribed in a 20-μL system using a reverse transcription kit (BeyoRT II M-MLV reverse transcriptase, D7160L, Biyuntian, Shanghai, China). qPCR was performed using the SYBR Green (SY1020, Solarbio, Beijing, China) fluorescence method using the cDNA as a template. Synthesis of the first-strand cDNA from the miRNA (#B532451, Sangon, Shanghai) was conducted using the 2 × Taq PCR MasterMix (PC1150, Solarbio, Beijing). The primer sequences used are listed in Table 1. Reaction conditions were as follows: pre-degeneration at 95°C for 30 s; denaturation at 95°C for 5 s, annealing at 60°C for 20 s, and extension at 72°C for 1 min, for a total of 40 cycles. The primers were synthesized and designed by Kingsley Biotechnology Co., Ltd., China. All data were analyzed using the 2^−ΔΔCt^ method, and the experiment was repeated three times.

**Table 1:** Primer sequences used for qPCR.

**Genes**	**5' to 3' sequence**	**Tm (**°**C)**	**Size /bp**
MyoD F	TCTATGATGACCCGTGTTTCG	58.3	124
MyoD R	TGCACCGCAGTAGGGAAGT	58.7	
MyoG F	GGGCTATGAGCGGACTGA	56.3	230
MyoG R	GCAGGGTGCTCCTCTTCA	56.1	
IGF-1R F	GCCAAACTCAACCGTCTA	51	237
IGF-1R R	ATTGCCCAACCTGCTGT	53.5	
β-actin F	CTGTGCCCATCTACGAGGGCTAT	64.5	155
β-actin R	TTTGATGTCACGCACGATTTCC	63.2	
mmu-miR-133a-3p F	TTTGGTCCCCTTCAACCAGCTG		
U6 F	GCTTCGGCAGCACATATACT	55.6	134
U6 R	GTGCAGGGTCCGAGGTATTC	59.2	

*mmu-miR-133a-3p, reverse primer Gene_ID,MIMAT0000145*.

### Western Blotting

Protein extraction from skeletal muscle was performed using PIPA lysates (WLA004, Wanleibio, China). Then, 12% SDS-PAGE electrophoresis (DYZNZ-24DN, Beijing Liuyi, China) was conducted using 20-μL samples that contained 40 μg of protein. The samples were electrotransferred onto a PVDF membrane (IPVH00010, Millipore, USA) and the membranes were sealed with 5% skim milk PBS-T at 25°C for 1 h. The sealing solution was discarded and the corresponding primary antibody was added and diluted appropriately; Cytc (1:500, WL02410), p-AMPKα (1:500, WL05103), AMPKα (1:1000, WL02254), PGC-1α (1:1000, WL02123), HO-1 (1:500, WL02400), NQO1 (1:500, WL04860), iNOS (1:400, WL0992a), NF-κBp65 (1:1000, WL01980), TNFα (1:1000, WL01581), MCP-1 (1:1000, WL02966), PI3K p85 (1:500, WL02240), p-Akt (1:1000, WLP001a), Akt (1:500, WL0003b), p-mTOR (1:500, WL03694), mTOR (1:500, WL02477), p-GSK3β (1:400, WL03518), GSK3β (1:500, WL01456), Bcl-2 (1:1000, WL01556), Bax (1:500, WL01637), caspase-3/cleaved caspase-3 (1:500, WL01992), caspase-9/cleaved caspase-9 (1:500, WL03421), β-actin (1:1000, WL0984a), COX IV (1:1000, WL02203), Histone H3 (1:1000, WL01372) (purchased from Wanleibio), pan-PKC (1:200, sc-17769, Santa), p-PKC (Thr410) (1:10000, ab76129, Abcam), IGF-1 (1:1000, ab63926, Abcam), Nox2 (1:1000, 19013-1-AP, Proteintech), Nox4 (1:1000, 14347-1-AP, Proteintech), p-PI3K p85/p55 (1:1000, AF3242, affinity), NRF1 (1:1000, AF5298, affinity), and TFAM (1:500, A3173, Abclonal Technology Co., Ltd., China) were used and incubation was performed overnight at 4°C. The next day, 1 × PBS-T was used to perform washing steps (five times) for the samples for 5 min and goat anti-rabbit IgG secondary antibodies were added. P-PKC (Thr410) and sheep anti-mouse IgG-HRP were added (1:5,000, WLA023, Wanleibio) and the sample was incubated at 37°C for 45 min before subjection to washing steps (five times) with 1 × PBS-T for 5 min. ECL color rendering (WLA003, Wanleibio, China), darkroom exposure, and film scanning were followed by analysis of the optical density of the target band, conducted using a gel image processing system (Gel-Pro-Analyzer software; WD-9413B, Beijing Liuyi, China).

### Immunoprecipitation Technique (Co-IP)

Briefly, the antibody was subjected to immobilization and dilution was performed using 20 × crosslinking buffer and 1 × ultra-pure water (NW10LVF, Hong Kong, Heal Force) to prepare 2 mL of 1 × crosslinking buffer for use in each IP reaction. The volume of the antibody (10 μg) was adjusted to 200 μL using sufficient ultrapure water and 20 × crosslinking buffer at a concentration of 1 ×. One milligram of the lysate was added to the centrifuge column with 80 μL of control agarose resin slurry (40 μL solid phase resin). One milligram of the lysate was then added and the mixture was incubated at 4°C for 30 min. For each immunoprecipitation reaction, 1 × improved Duchenne PBS 2 mL was prepared using ultrapure water, following which 200 μL of IP cracking/washing buffer was added and the mixture were centrifuged. A volume of 5 μL of 5 × sample loading buffer was then added to a 20-μL sample via immunoprecipitation elution. The samples were incubated at 95–100°C for approximately 5 min and were cooled to room temperature prior to analysis via SDS-PAGE electrophoresis. Refer to 2.9 for details on western blotting.

### Molecular Docking

The software of MOE (v2018.01011, Montreal, QC, Canada) was used for the molecular docking simulation of AMPK subunit alpha-1 and LCBP components. The 3D structures of the AMPK subunit alpha-1 were constructed using AlphaFold. The 2D structures of the cyanidin-3-glucoside, catechin, and chlorogenic acid from the Pubchem database (https://pubchem.ncbi.nlm.nih.gov/) were downloaded, to be used as the docking ligands for the 3D structures in MOE by energy minimization. Prior to docking, the force field of AMBER10:EHT and the implicit solvation model of Reaction Field (R-field) were applied. The “induced fit” protocol was selected and ensured that the best binding mode is the one with the lowest free energy of binding. The binding site of AMPK protein and its natural ligand in the crystal structure was set as the binding pocket of the compound. The diagram of the intermolecular binding pattern was completed on PyMOL (www.pymol.org).

### Statistical Analyses

The SPSS software (version 19.0; IBM Corp., Armonk, NY) was used for statistical analysis. All experiments were repeated at least triplicates. The measurement results are expressed as means ± standard deviations. The one-way ANOVA was used for comparison between multiple groups with Tukey's multiple comparisons test. Results meeting the criteria of *p* < 0.05 and *p* < 0.01 were considered statistically significant.

## Results

### The LCBP Components

The results of the LCBP phenolic components determined using HPLC-DAD-ESI-MS/MS are shown in Table 2. The LCBP was found to mainly comprise cyanidin-3-glucoside, catechin, and chlorogenic acid, and the findings were consistent with the results reported in our previous study (19). The contents of the three monomers accounted for 43.9, 26.4, and 12.3% of the total phenols estimated, respectively. This finding was slightly different from that obtained previously, which might be attributable to differences in the climate, soil moisture, or extraction methods used.

**Table 2:** The main phenolic compounds in extracts of wild *Lonicera caerulea* berry.

**Component**	**MS (m/z)**	**MS/MS (m/z)**	**Content (mg/g dry wt)**
**Anthocyanins**			
Cyanidin-3-sophorose-5- glucoside	773 [M+H]	611, 287	1.14 ± 0.05
Cyanidin-3,5-glucoside	611 [M+H]	449, 287	16.31 ± 0.16
Cyanidin-3-glucoside	449 [M+H]	287	280.12 ± 2.17
Cyanidin-3-rutinoside	595 [M+H]	449,287	17.28 ± 0.12
Pelargonidin-3-glucoside	433 [M+H]	271	4.16 ± 0.09
Peonidin-3-glucoside	463 [M+H]	301	11.72 ± 0.18
Peonidin-3-rutinoside	609 [M+H]	463,301	1.49 ± 0.06
Delphinidin-3-rutinoside	611 [M+H]	465,303	0.58 ± 0.01
Total content			332.8
**Other phenolic components**			
Neochlorogenic acid	353 [M-H]	191	13.16 ± 0.12
Catechin	289 [M-H]	245,205,179	168.57 ± 2.68
Chlorogenic acid	353 [M-H]	191	78.16 ± 1.42
Procyanidin dimer	577 [M-H]	289	5.57 ± 0.13
Vanillic acid	167 [M-H]	123	1.36 ± 0.07
Procyanidin trimer	865 [M-H]	577,289	5.47 ± 0.11
Quercetin-3-O-rutinoside	609 [M-H]	301	1.68 ± 0.01
Hyperoside	463 [M-H]	301	21.23 ± 0.75
Quercetin-3-O-glucoside	463 [M-H]	301	1.76 ± 0.02
3,5-dicaffeoylquinic acid	515 [M-H]	353	5.22 ± 0.16
Luteolin-7-O-rutinoside	593 [M-H]	285	1.33 ± 0.05
Luteolin-7-O-glucoside	447 [M-H]	285	1.71 ± 0.07
Total content			305.22
Total phenolic content			638.02

*Values are expressed as means ± standard deviation. [M+H] and [M-H] indicate identification in the positive and negative ionization modes, respectively*.

### LCBP Improves the Physical and Chemical Indices

**Figure 1:**
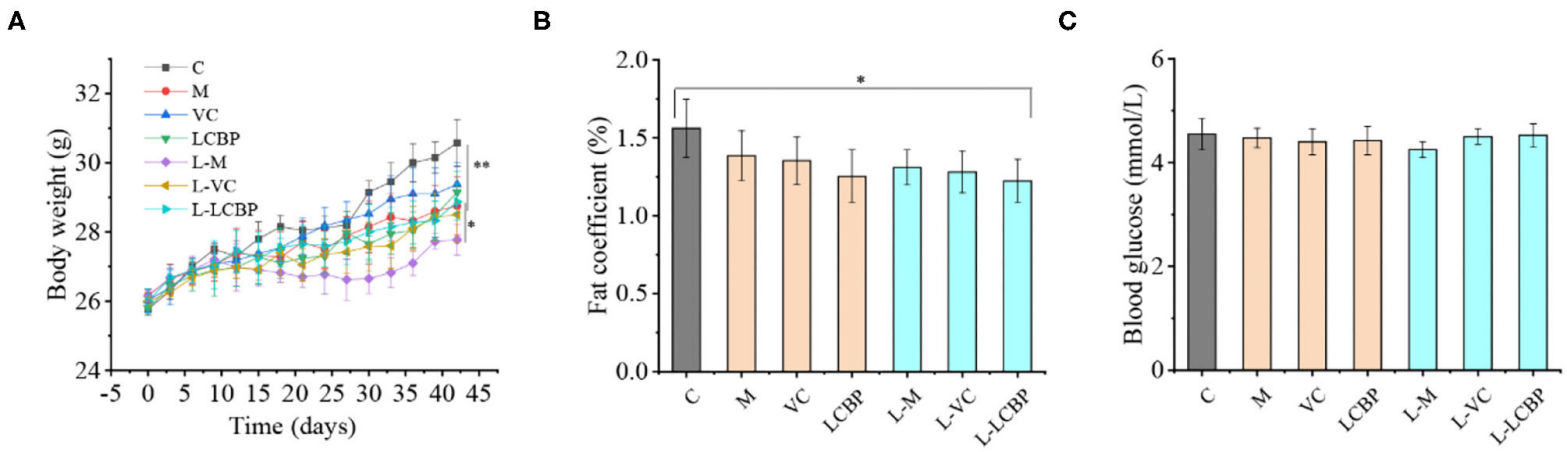
Effects of LCBP exerted on physical and chemical indices of long-term exercise-fatigued mice. **(A)** The weights of the mice were recorded every 3 days. **(B)** Epididymis, perirenal, and mesenteric adipose tissues were weighed, and the adipose coefficient was calculated. Adipose coefficient, adipose weight/body weight. **(C)** Blood glucose levels were detected using blood glucose dipstick after subjection to a 6-week routine treadmill experiment. M, VC and LCBP groups were treated at 25°C; L-M, L-VC and L-LCBP groups were treated at −5°C; **p < 0.01, *p < 0.05.

The effects of LCBP on the body weight, fat coefficient, and blood glucose levels of exercise-fatigued mice are shown in Figure 1. Compared with the inactive group, long-term exercise resulted in a decrease in the body weight and the fat coefficient. The body weight of the exercise-subjected groups was at least 6.86% lower than that of the inactive group (25°C) (*p* < 0.01), and the body weight of the group that was subjected to exercise at low temperature (-5°C) was 9.86% lower than the inactive group. Mice in both VC and LCBP groups exhibited higher body weight than those subjected to exercise at low temperature; however, no significant difference was observed between these groups and the model group (Figure 1A). Interestingly, the fat coefficient of the mice in the VC and LCBP groups was reduced at both 25 and −5°C (Figure 1B, *p* > 0.05). The results indicate that dietary supplementation with LCBP may reduce the fat content while increasing the body weight of mice subjected to exercise at 25 and −5°C over long durations. This may be attributable to an increase in the muscle mass, which is conducive to increasing exercise performance. Notably, the effect was observed regardless of the environmental temperature. The blood glucose content remained similar in mice subjected to exercise at both 25 and −5°C, and low temperature groups slightly increased (Figure 1C, *p* > 0.05). This may be due to the higher glucose consumption of LCBP group because of their higher exercising capacity. This was reflected in the unaltered glucose levels after exhaustion.

### LCBP Improves Oxidative Stress in Exercise-Fatigued Mice

The results obtained for oxidative stress-related indicators in skeletal muscle and serum are shown in Table 3. Compared with the control group I, long-term exercise fatigue seemed to significantly increase both the production of MDA and oxidation in groups M and L-M. This effect was more pronounced in the mice subjected to exercise in low-temperature (-5°C) environments, in which significant oxidative damage was observed in the skeletal muscle (*p* < 0.01). Dietary supplementation with LCBP significantly reduced MDA production in the blood and skeletal muscle of the M and LCBP groups subjected to exercise under ambient temperature conditions, leading to the achievement of values of 47.6 and 47.4%, respectively, while decreases of 49.8 and 51.8% were observed at −5°C (*p* < 0.01) (L-M vs. L-LCBP). Long-term exercise significantly reduced the total antioxidant content (T-AOC) in skeletal muscle and blood (*p* < 0.01), and the effect was exacerbated at low temperatures (-5°C). Dietary supplementation with LCBP was observed to significantly increase the total antioxidant content of the exercise-fatigued mice (*p* < 0.01). Compared with the M group, the T-AOC in blood and skeletal muscle of the LCBP mice increased by 46.2 and 71.9%, respectively, at 25°C, and by 77.5 and 121%, respectively, at −5°C. These results suggest that LCBP is more effective in improving the total antioxidant capacity of exercise-fatigued mice in a low-temperature environment. It is speculated that this is attributable to the enhanced activity of antioxidant enzymes such as SOD, CAT, and GSH-Px. Compared with the M group, dietary supplementation with LCBP or VC significantly increased the activities of the SOD, CAT, and GSH-Px enzymes in blood and skeletal muscle (*p* < 0.01), thereby improving antioxidant capacity and reducing oxidative stress injuries in mice with long-term exercise fatigue.

**Table 3:** Skeletal muscle tissue and serum antioxidant capacity in mice.

**Groups**	**MDA**		**T-AOC**		**SOD**		**CAT**		**GSH-PX**	
	**Serum** **(nmol/mL)**	**Skeletal muscle** **(nmol/mg protein)**	**Serum** **(μmol/mL)**	**Skeletal muscle** **(μmol/mg protein)**	**Serum** **(U/mL)**	**Skeletal muscle** **(U/mg protein)**	**Serum** **(U/mL)**	**Skeletal muscle** **(U/mg protein)**	**Serum** **(U/mL)**	**Skeletal muscle** **(U/mg protein)**
C	5.60 ± 0.38	3.26 ± 0.32	1.99 ± 0.11	0.65 ± 0.05	59.58 ± 2.29	32.42 ± 1.68	22.67 ± 0.76	12.83 ± 0.48	190.79 ± 4.99	26.91 ± 0.63
M	13.88 ± 0.63	8.14 ± 0.34	1.19 ± 0.09	0.32 ± 0.02	30.34 ± 1.48	17.88 ± 1.12	10.81 ± 0.63	5.36 ± 0.31	95.06 ± 8.03	11.36 ± 0.81
VC	8.37 ± 0.36	5.21 ± 0.37	1.53 ± 0.11	0.45 ± 0.02	42.89 ± 1.80	24.70 ± 1.51	14.86 ± 0.56	8.79 ± 0.70	127.75 ± 6.18	18.06 ± 0.79
LCBP	7.27 ± 0.42**	4.28 ± 0.39**	1.74 ± 0.07**	0.55 ± 0.03**	47.42 ± 1.94**	27.68 ± 1.34**	16.97 ± 0.71**	10.30 ± 0.64**	152.36 ± 6.58**	21.73 ± 0.87**
L-M	17.94 ± 0.36	10.42 ± 0.45	0.80 ± 0.06	0.19 ± 0.01	18.92 ± 1.28	11.39 ± 0.78	6.76 ± 0.42	3.22 ± 0.23	66.40 ± 5.75	7.59 ± 0.74
L-VC	11.82 ± 0.44	6.51 ± 0.35	1.25 ± 0.08	0.35 ± 0.02	33.85 ± 1.54	19.48 ± 1.12	12.42 ± 0.56	6.65 ± 0.34	114.61 ± 5.19	14.82 ± 1.35
L-LCBP	9.00 ± 0.398^##^	5.02 ± 0.22^##^	1.42 ± 0.05^##^	0.42 ± 0.03^##^	41.49 ± 2.20^##^	22.63 ± 0.78^##^	14.50 ± 0.47^##^	8.67 ± 0.59^##^	132.13 ± 4.99^##^	18.69 ± 0.72^##^

*Values are expressed as mean ± standard deviation. M vs. LCBP*,

** *p < 0.01; L-M vs. L-LCBP*,

## *p < 0.01; LCBP, Lonicera Caerulea berry polyphenols; VC, vitamin C. Individuals in C, the control group, were fed for 6 weeks without exercise and were provided with the same amount of normal saline intragastrically at the same time as other groups at 22 ± 1 C; M and L-M. The normal temperature exercise model group and the low-temperature exercise model group were subjected to exercise at 25 C and −5°C for 6 weeks, respectively, 6 days a week, for 45 min/d, on a platform with a slope of 0. The first week included subjection to adaptive training, in which the initial speed of 11 m/min was increased at a rate of 1 m/min every day. From second week onward, the speed was increased by 1 m/min every week, and the same amount of normal saline was administered 1 h before exercise. VC and L-VC mice in the positive groups were subjected to exercise in the same way as the model group for 6 weeks, and they received VC intragastric administration (60 mg/kg) at 1 h before subjection to exercise at 25 °C and −5°C. LCBP and L-LCBP (250 mg/kg) were administered intragastrically 1 h before subjection to exercise at 25 and −5°C*.

### LCBP Improves the Anti-fatigue Physiological Indices of Mice Subjected to Long-Term Exercise

**Figure 2:**
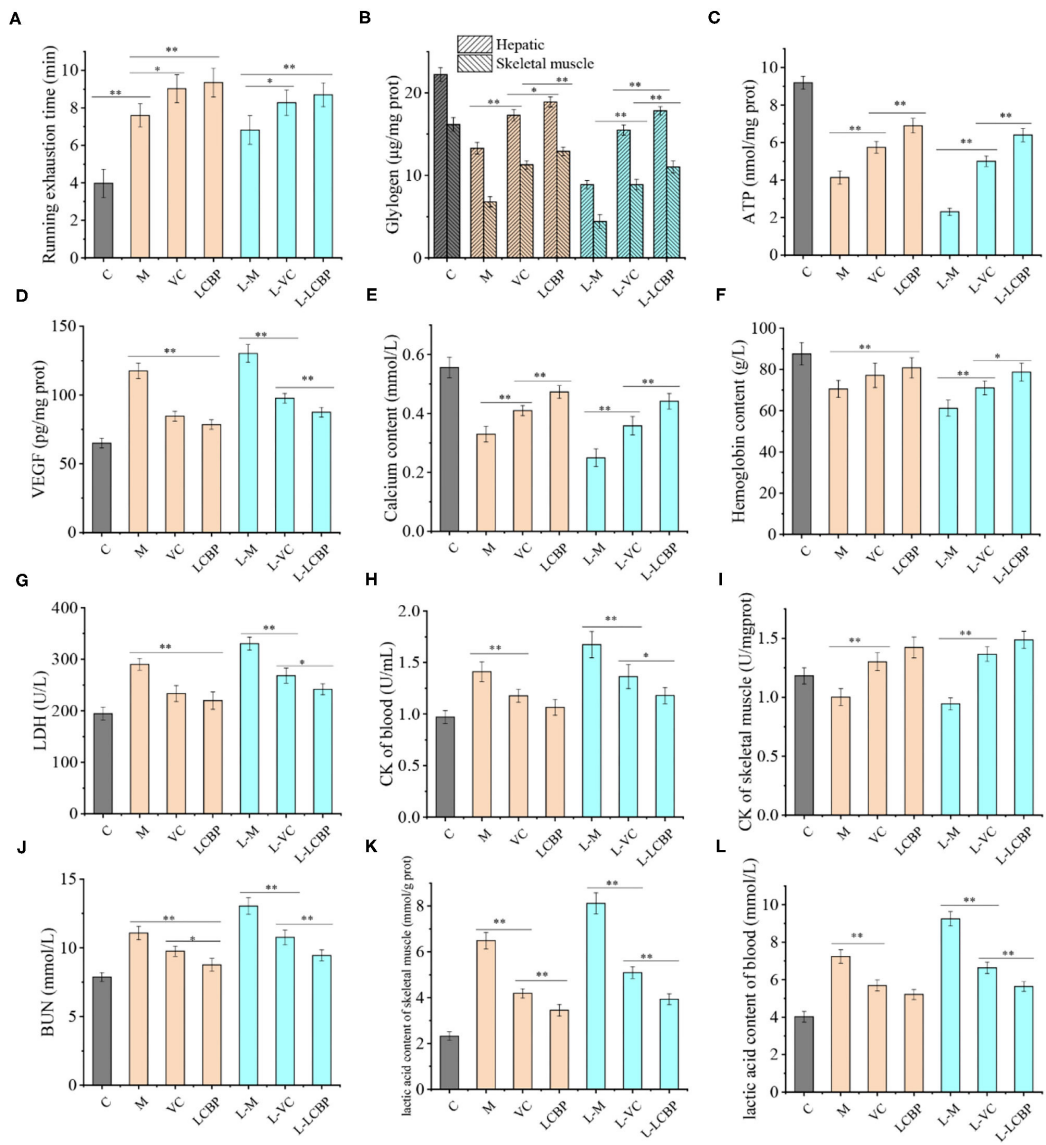
LCBP improves the fatigue physiological indices of mice subjected to long-term exercise fatigue. M, VC and LCBP groups were treated at 25°C; L-M, L-VC and L-LCBP groups were treated at −5°C; **p < 0.01, *p < 0.05.

The measurement results obtained for physiological indicators related to fatigue in mice subjected to exercise over long periods are shown in Figure 2. Compared with the inactive group, long-term exercise significantly increased the exhaustion time of mice from 3.98 min to 7.60 min (Figure 2A), and dietary supplementation with LCBP further increased the exhaustion time to 9.15 min (*p* < 0.01), which was 0.12 min longer than that observed in the VC control group. Low temperature (-5°C) exposure has been reported to be harmful during exercise. Supporting this finding, the exhaustion time was found to be lower at −5°C than that in the high-temperature (25°C) group. However, the effects of LCBP did not change significantly with temperature, and the exhaustion time was extended only from 6.83 to 8.70 min as a result of exposure to a different temperature. Compared with the inactive group, subjection to exercise led to a significant depletion of the glycogen content in the liver and skeletal muscle (Figure 2B). Compared with the M group, dietary supplement LCBP the amount of glycogen stored in the liver and skeletal muscle was significantly increased by 42.7 and 90.0%, respectively, (*p* < 0.01). Increase in glucose production improved athletic performance. The glycogen content stored in the liver and skeletal muscle of the L-LCBP group was 2.0 times and 2.49 times higher than that in the L-M group under low-temperature conditions (-5°C), which was better than the effects observed in the group that was subjected to exercise at 25°C. The increase observed in the LCBP group (250 mg/kg) was higher than that observed in the VC (60 mg/kg) group (*p* < 0.01 or *p* < 0.05). The ability of LCBP to increase glycogen storage can be inferred from the data presented in Figure 2C. Both glycogen consumption and ATP production decreased following subjection to exhaustive exercise (*p* < 0.01) and dietary supplementation with LCBP was observed to significantly increase the production of ATP in skeletal muscle (at both−5 and 25°C) (*p* < 0.01). The VEGFA levels in skeletal muscle, hemoglobin levels, and Ca^2+^ levels in the serum have been depicted in Figures 2D–F. Long-term exercise fatigue significantly increased the VEGFA levels in skeletal muscle (*p* < 0.01), especially when exercise was performed in a low-temperature environment. Compared with the model group, dietary LCBP supplementation significantly reduced the VEGFA levels by 33.3 and 32.8% at −5 and 25°C, respectively. Prolonged subjection to intense exercise can reduce blood hemoglobin, Ca^2+^ levels (Figures 2E,F), and oxygen retention. Compared with the model group, dietary LCBP supplementation was found to significantly reverse this phenomenon at both−5 and 25°C (*p* < 0.01), and the increase was more significant than that observed in the positive control VC group. The results for BUN and lactate dehydrogenase activity (LDH) are shown in Figures 2G,J, and the results for CK and LA in serum and skeletal muscle are shown in Figures 2H,I,K,L. Dietary LCBP supplementation significantly reduced serum LDH levels in mice compared with the model group (*p* < 0.01). LDH can catalyze pyruvate breakdown to eventually generate lactic acid, indicating that reduction in the accumulation of lactic acid will alleviate a decrease in exercise ability that results from muscle fatigue. This result is consistent with the finding presented in Figures 2K,I. The accumulation of LA in the serum and skeletal muscle of individuals in the LCBP group was significantly lower than that observed in the model group (*p* < 0.01), which might be attributed to an improvement in the rate at which ATP was synthesized in skeletal muscle during exercise, thereby improving oxygen uptake and utilization, reducing the production and accumulation of lactic acid, and relieving exercise fatigue. This was consistent with the finding illustrated in Figure 2C. Moreover, subjection to exercise at low temperature (−5°C) increased the production of LDH and LA, but did not alter the effects associated with LCBP supplementation. With subjection to prolonged periods of an exercise regimen or with the performance of an intensive exercise, proteins and amino acids can undergo catabolism, causing the urea nitrogen content in the serum to increase markedly. The results showed that supplementation with LCBP significantly reduced the BUN levels in the serum of mice after subjection to exercise (*p* < 0.01) (Figure 2J); this was followed by decreases of 20.9 and 27.6% at 25 and −5°C, respectively. These results suggest that dietary supplementation with LCBP can help reduce protein catabolism and mitigate against muscle injury. CK is generally located in the muscle cells (Figure 2I), and elevated CK levels in blood indicate the occurrence of muscle injury (either complete or active) (25). Compared with the inactive group, increased content of CK was detected in the serum of mice subjected to exercise, both at 25 and −5°C (*p* < 0.01), and dietary LCBP supplementation significantly reduced the CK content in the serum by 24.5 and 29.5% at 25 and −5°C, respectively. The above-mentioned data indicate that dietary LCBP improves fatigue significantly when exercise is performed in a low-temperature environment.

### LCBP Improves ROS and Related Protein Expression in Exercise Fatigue

**Figure 3:**
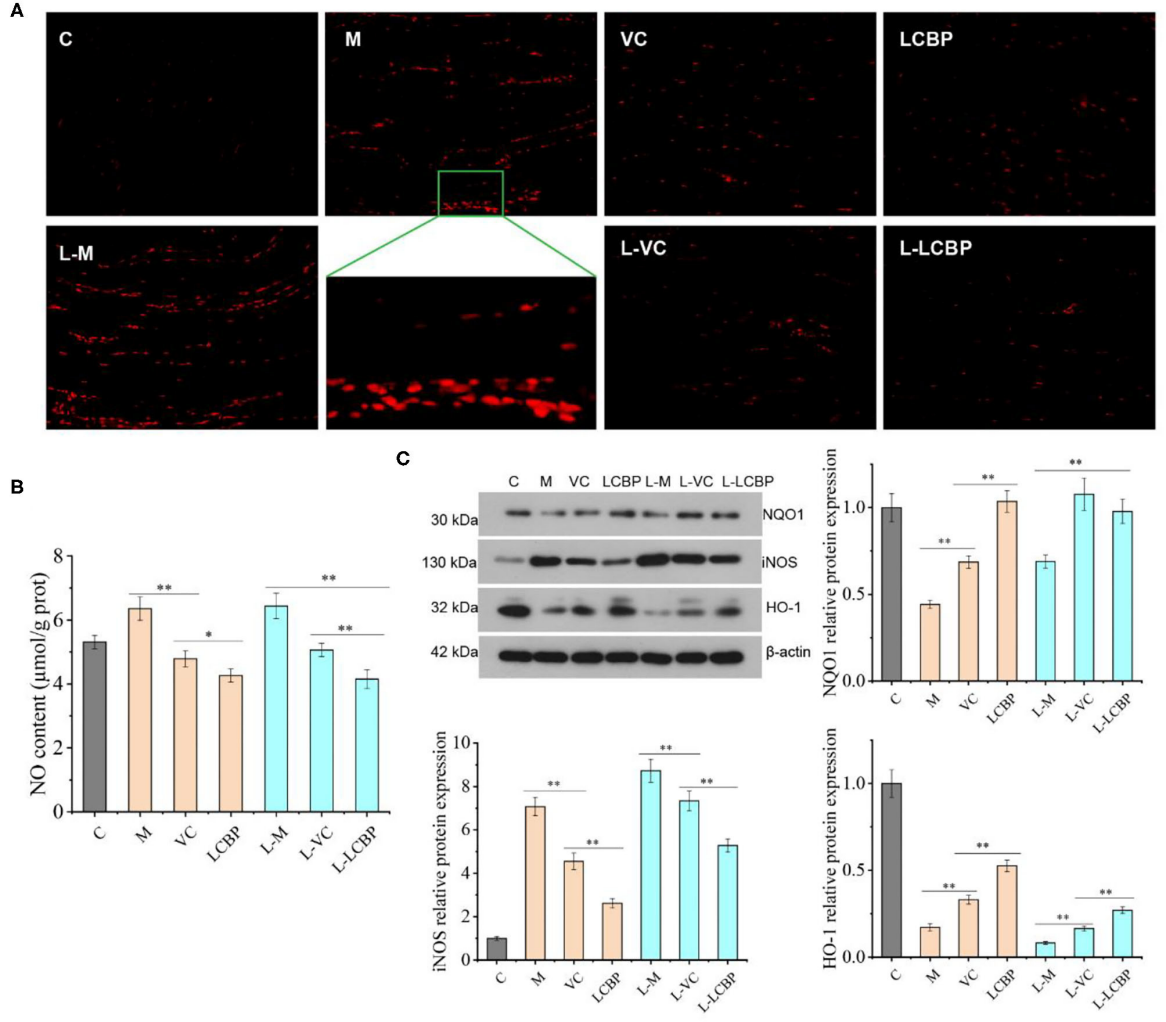
LCBP regulates the expression of ROS and related proteins under oxidative stress conditions in mice subjected to long-term exercise fatigue. **(A)** Reactive oxygen species (ROS) fluorescence map of skeletal muscle (magnification: 200×), with red dots representing ROS production. **(B)** NO levels in the skeletal muscle. **(C)** Protein expression in the skeletal muscle. M, VC and LCBP groups were treated at 25°C; L-M, L-VC and L-LCBP groups were treated at −5°C; **p < 0.01, *p < 0.05.

The fatigue that results from subjection to long-term exercise can lead to oxidative damage in the skeletal muscle (Figure 3). The results of DHE staining performed for analysis of the ROS levels in skeletal muscle are shown in Figure 2A, with the red dots representing ROS production. The number of red fluorescent dots increased significantly in the skeletal muscle of mice subjected to exercise at both 25 and −5°C; however, this was more noticeable in the −5°C environment, indicating that exercising at low temperatures accelerated the production of ROS. Dietary supplementation with VC or LCBP can significantly reduce ROS production and oxidative damage that occurs in skeletal muscle. Reduction in ROS production was significantly improved in the LCBP groups as compared to the VC groups. The performance of long-term exercise also leads to the production of excessive NO, the levels of which increase at low temperatures, exacerbating oxidative damage (Figure 3B). LCBP was found to significantly inhibit NO production (*p* < 0.01) and confer protection to cells against oxidative stress, leading to a reduction in NO production by 32.9 and 35.6%, respectively, as compared to that observed in the 25 and −5°C model groups. The effect of NO reduction was also better in the LCBP group than that in the positive control VC group (*p* < 0.05, *p* < 0.01). Western blotting was performed to detect the expression of oxidative stress-related proteins (Figure 3C). Long-term exercise was found to significantly upregulate the expression of the iNOS protein (*p* < 0.01) while significantly downregulating the expression of the NQO1 and HO-1 proteins (*p* < 0.01). Dietary LCBP supplementation significantly reversed such a phenomenon. These results indicate that LCBP can regulate the expression of oxidative stress-related proteins to improve the oxidative damage that occurs in skeletal muscle as a result of subjection to long-term exercise, and that this effect also occurs in a low-temperature environment.

### LCBP Alleviates the Inflammation Associated With Fatigue in Exercise-Subjected Mice

**Figure 4:**
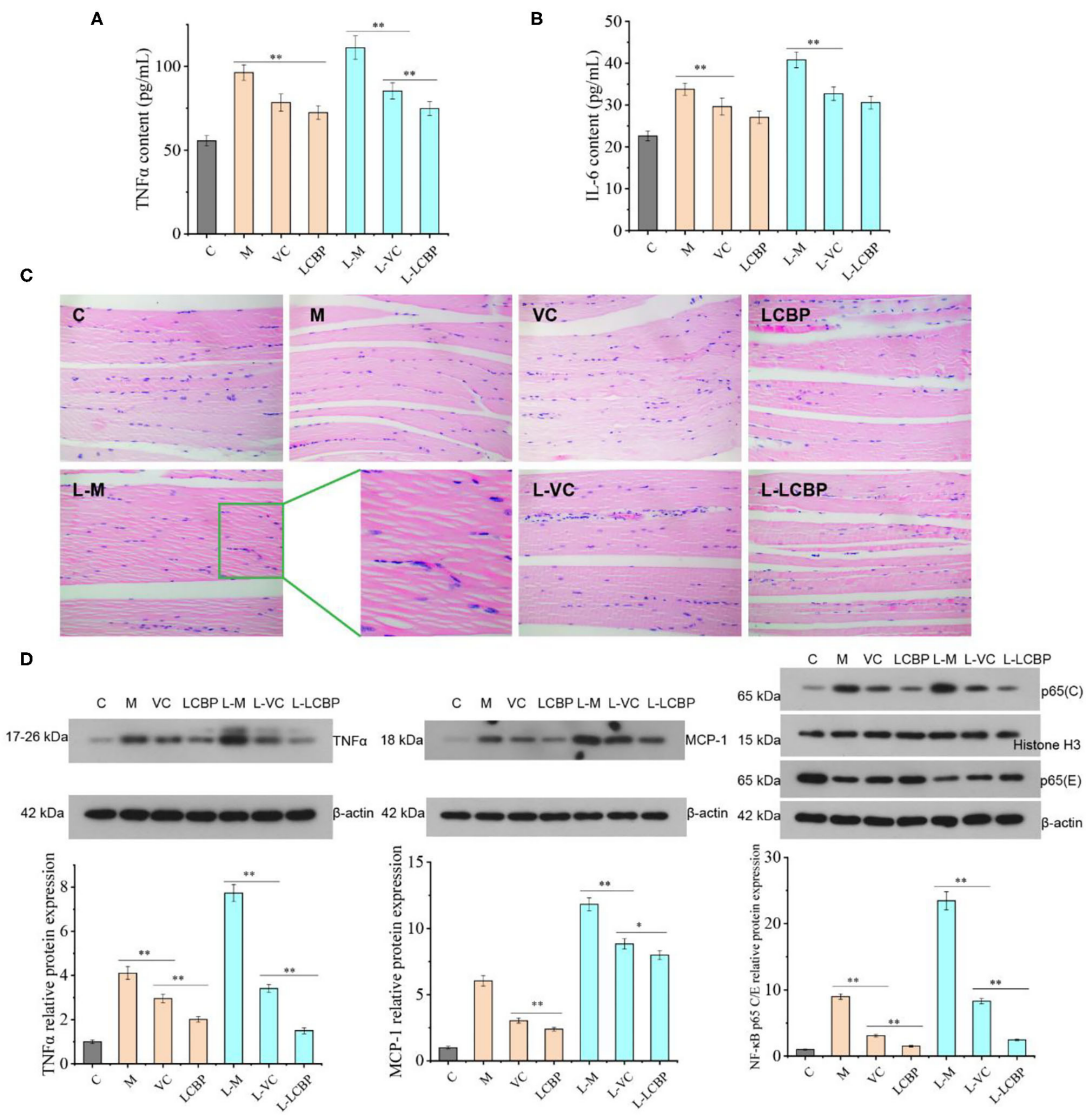
LCBP alleviates the inflammatory state in mice subjected to long-term exercise fatigue. **(A,B)** Serum levels of TNFα and IL-6 **(C)** HE staining of skeletal muscle tissues. **(D)** Expression of inflammatory proteins was detected *via* western blotting. NF-κB p65 cytoblast/endochylema relative protein expression. M, VC and LCBP groups were treated at 25°C; L-M, L-VC and L-LCBP groups were treated at −5°C; **p < 0.01, *p < 0.05.

Long-term exercise leads to an increase in inflammation (Figure 4). The results for the serum inflammatory cytokines TNFα and IL-6 have been shown in Figures 4A,B. Compared with the 25 and −5°C model groups, dietary supplementation with LCBP was observed to significantly reduce the secretion of the serum inflammatory factor TNFα by 24.7 and 31.9%, respectively. Exposure to cold temperatures results in the exacerbation of inflammation. However, the results obtained for the group subjected to treatment with LCBP were similar to those obtained for the 25°C group, indicating that exercising at low temperatures did not affect the anti-inflammatory effect of LCBP. However, the anti-inflammatory effect was better in the LCBP group than that in the VC group, and the IL-6 data demonstrated the same trend. The inflammatory damage to skeletal muscle that results from long-term exercise fatigue was also observed in the HE-stained sections (Figure 4C). After subjection to long-term exercise, the skeletal muscle fibers of the mice changed and demonstrated features such as blurring at the boundaries of the muscle bundles, irregularly arranged muscle fibers, interlaminar diffusion, enlarged spaces, connective tissue hyperplasia, inflammatory cell infiltration, and apoptosis, and these changes were the most remarkable in the low-temperature (-5°C) exercise group. Dietary supplementation with LCBP or VC can help improve this phenomenon, reduce inflammatory infiltration, and reduce the spaces between the muscle fibers. Further analysis of the proteins expressed as a result of inflammation (Figure 4D) showed that the nuclear/cytoplasmic levels of TNFα, MCP-1, and NF-κB P65 were significantly upregulated in the normal temperature- and cold temperature-subjected exercise models (*p* < 0.01). The upregulation observed in the −5°C environment indicate that low temperatures increase inflammation. Dietary LCBP supplementation significantly downregulates the expression of these proteins (*p* < 0.01) and inhibits excessive inflammation, and such findings are consistent with the results depicted in Figures 4A,B. Both LCBP and VC downregulated TNFα and NF-κB P65 expression more efficiently than MCP-1. Additionally, the anti-inflammatory effect of LCBP remained significant at low temperatures.

### LCBP Regulates the Expression of Apoptosis-Related Proteins in Skeletal Muscle Mitochondria

**Figure 5:**
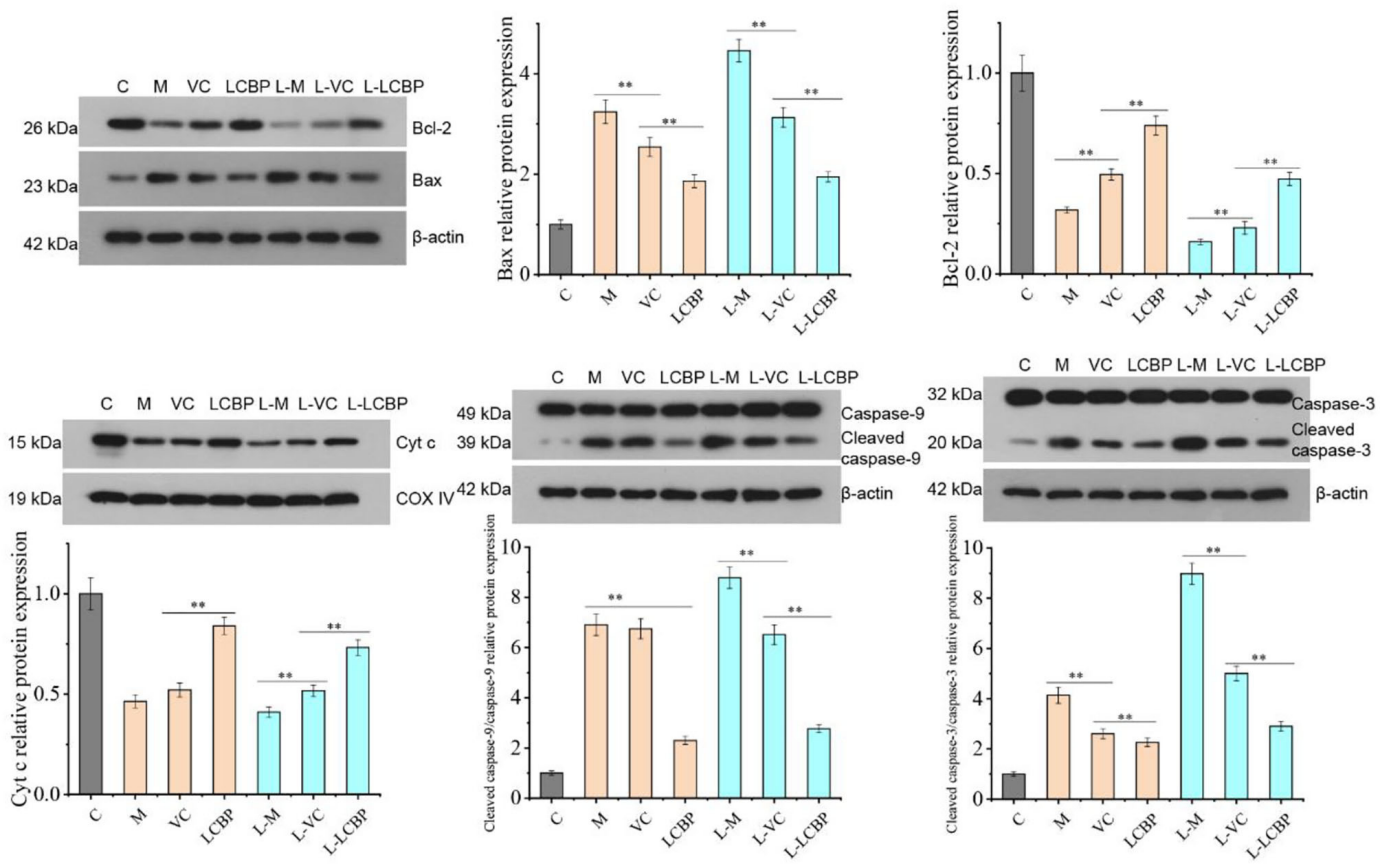
Western blot analysis of LCBP effect on the expression of apoptosis-related proteins in skeletal muscle mitochondria of long-term exercise-fatigued mice. M, VC and LCBP groups were treated at 25°C; L-M, L-VC and L-LCBP groups were treated at −5°C; **p < 0.01.

Performance of long-term exercise can induce apoptosis in skeletal muscle mitochondria (Figure 5). Compared with the inactive group, the levels of mitochondrial apoptosis-related proteins Bcl-2 and Cyt C were significantly downregulated (*p* < 0.01), while those of Bax, caspase-9, and caspase-3 were significantly upregulated (*p* < 0.01) in both the 25 and −5°C exercise groups. Low temperatures were found to exacerbate this phenomenon. Compared with the M or L-M group, dietary LCBP supplementation significantly inhibited the expression of the Bax protein and promoted the expression of the Bcl-2 protein, increasing the Bcl-2/Bax ratio, and further downregulated the protein expression of Caspase-9 and Caspase-3, and inhibited or delayed apoptosis in the skeletal muscle cells, thus delaying exercise fatigue. LCBP upregulates the expression of the Cyt C protein, thus regulating mitochondrial energy metabolism and improving oxygen utilization to exert an anti-fatigue effect. VC exerted no significant effect on caspase-9 expression at 25°C (*p* > 0.05); however, this effect was significantly different at low temperature (*p* < 0.01), with the demonstration of an increased expression of Caspase-9 in the model group.

### LCBP Regulates the PKC-NOX2/Nox4 Pathway to Reduce ROS Production

**Figure 6:**
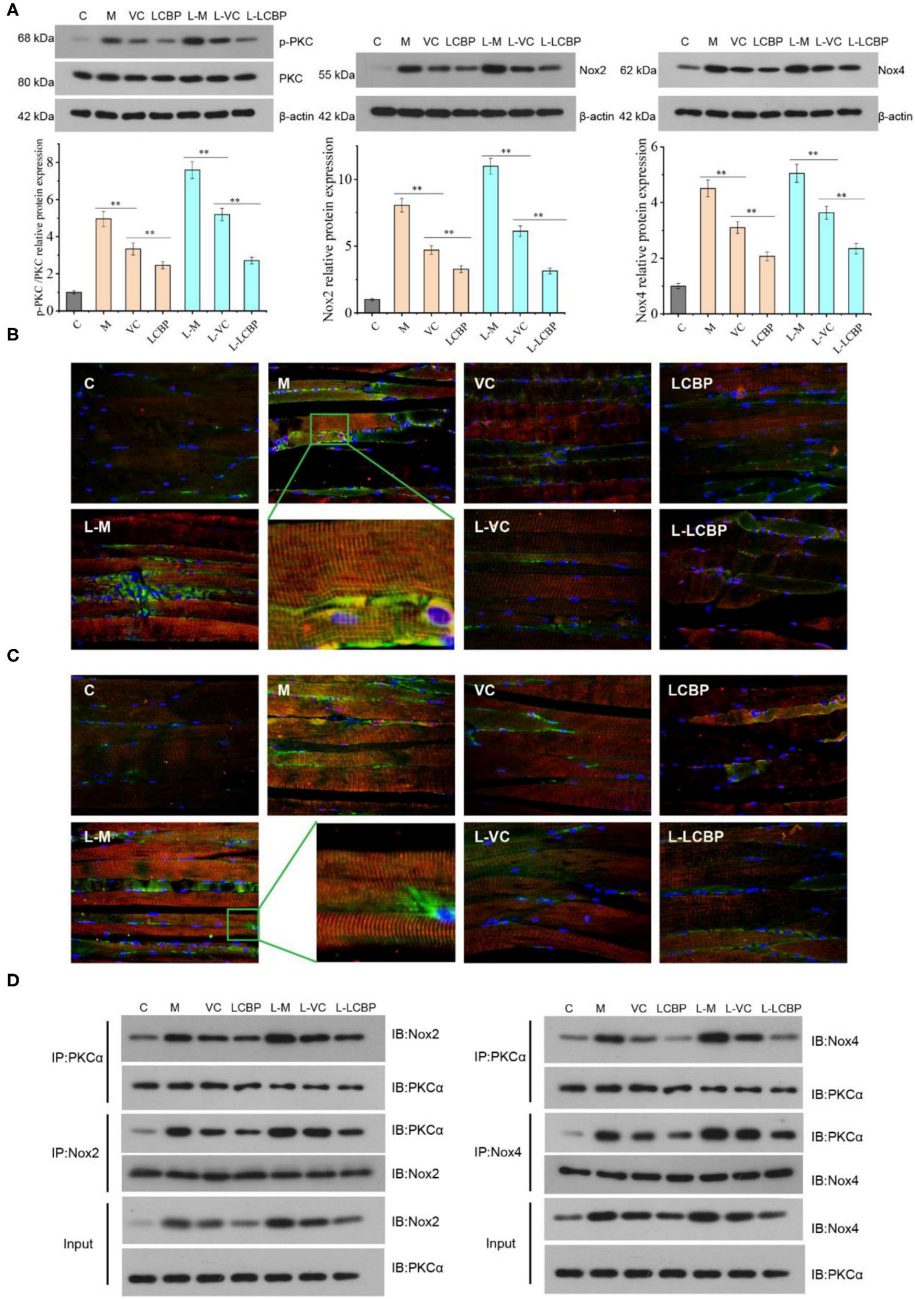
LCBP regulates the PKCα- Nox2 /Nox4 pathway in skeletal muscle of mice presenting with long-term exercise fatigue. **(A)** Western blotting was performed to detect related protein expression; **(B)** PKCα+Nox2 protein expression was detected via fluorescence analysis. Red fluorescence represents PKCα, green fluorescence represents Nox2; **(C)** PKCα+Nox4 protein expression was detected via fluorescence analysis. Red fluorescence represents PKCα and green fluorescence represents Nox4; **(D)** the interaction between PKCα and Nox2/Nox4 was detected *via* immunoprecipitation, that is, PKC antibody was used to precipitate proteins, and western blotting was performed to analyze the Nox4/Nox2 protein content in the precipitate. IgG is not expressed, not displayed. M, VC and LCBP groups were treated at 25°C; L-M, L-VC and L-LCBP groups were treated at −5°C; **p < 0.01.

The phosphorylation levels of PKC and the expression of Nox2 and Nox4 proteins were found to be significantly increased in the skeletal muscle of mice subjected to stimulation by long-term exercise fatigue (*p* < 0.01) This effect was found to be intensified at −5°C compared to that at 25°C (Figure 6A). Dietary supplementation with LCBP was found to significantly downregulate the expression of these proteins (*p* < 0.01), with the downregulation of expression of Nox2 and Nox4 by 71.4 and 53.7% at −5°C, respectively, as compared to the model group. The PKCα/Nox4 and PKCα/Nox2 in skeletal muscle was detected using the immunofluorescence double-labeling method (Figures 6B,C), and the results have been illustrated in Figure 6A. In Figure 6B, PKCα is represented by red fluorescence and Nox2 is represented by green fluorescence. The levels of the two proteins were low in the inactive group, while the co-expression of PKCα and Nox2 was observed to increase in the skeletal muscle of the long-term exercise model group. Both LCBP and VC significantly reversed this phenomenon, especially LCBP. The coexpression increased at low temperatures. In Figure 6C, red fluorescence represents PKCα expression and the green fluorescence represents Nox4 expression. We found that in contrast to the findings presented in Figure 6B, more red fluorescence was observed than green fluorescence in the M and L-M groups, indicating that the expression of PKCα increased when it was co-expressed with Nox4. LCBP and VC significantly reversed this phenomenon, and the expression trend was consistent with that depicted in Figure 6B. The effect of LCBP on the interaction between PKCα and Nox4/ Nox2 was further observed, and the results of the immunoprecipitation experiment (Figure 6D) indicated that PKC and Nox4 established interaction to form a PKC- Nox4 complex, and PKC and Nox2 established interaction to form a PKC- Nox2 complex. The expression trend observed in the results is consistent with the finding presented in Figures 6B,C, indicating that long-term exercise fatigue stimulates the production of both PKC-Nox2 and PKC-Nox4 complexes, which may mediate the production of exercise-induced ROS. Dietary supplementation with LCBP weakened the production of athletic PKC-NOX2 and PKC-NOX4 complexes and reduced ROS production.

### LCBP Regulates the AMPK-PGC1-α-NRF1-TFAM Signal Axis Pathway of Mitochondrial Biosynthesis in Skeletal Muscle

**Figure 7:**
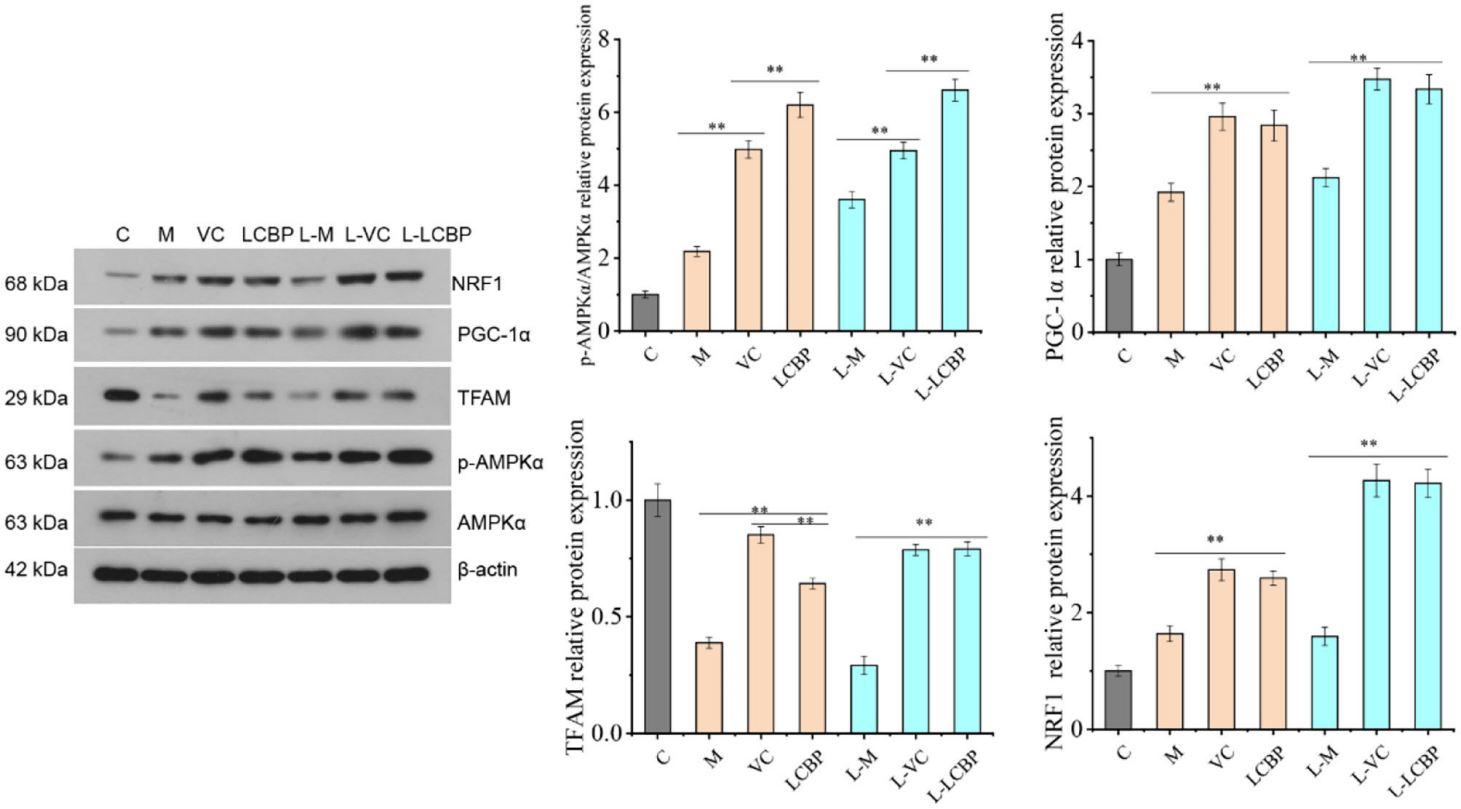
Effects of LCBP on mitochondrial biosynthesis-related protein expression in skeletal muscle of long-term exercise-fatigued mice detected via western blotting. M, VC and LCBP groups were treated at 25°C; L-M, L-VC and L-LCBP groups were treated at −5°C; **p < 0.01.

Performance of long-term exercise was found to significantly enhance the expression of AMPK phosphorylated protein, PGC1-α, and NRF1 protein (*p* < 0.01), thereby contributing to the improvement observed in performance (Figure 7). Dietary supplementation with LCBP significantly enhanced this effect (*p* < 0.01). Compared with the M and L-M groups, the expression of AMPK phosphorylated protein was upregulated by 184.4 and 83.4%, and the expression of PGC1-α was upregulated by 47.7 and 57.0%, respectively, in the LCBP group. Dietary supplementation with either LCBP or VC significantly enhanced TFAM protein expression (*p* < 0.01). Therefore, it can be inferred that LCBP can activate the AMPK-PGC1-α -NRF1-TFAM signal axis in skeletal muscle, can improve skeletal mitochondrial biosynthesis, and can reduce fatigue, thus improving exercise endurance.

### LCBP Promotes the Proliferation of Skeletal Muscle Cells Through the miRNA-133a/IGF-1/PI3K/Akt/mTOR Pathway

**Figure 8:**
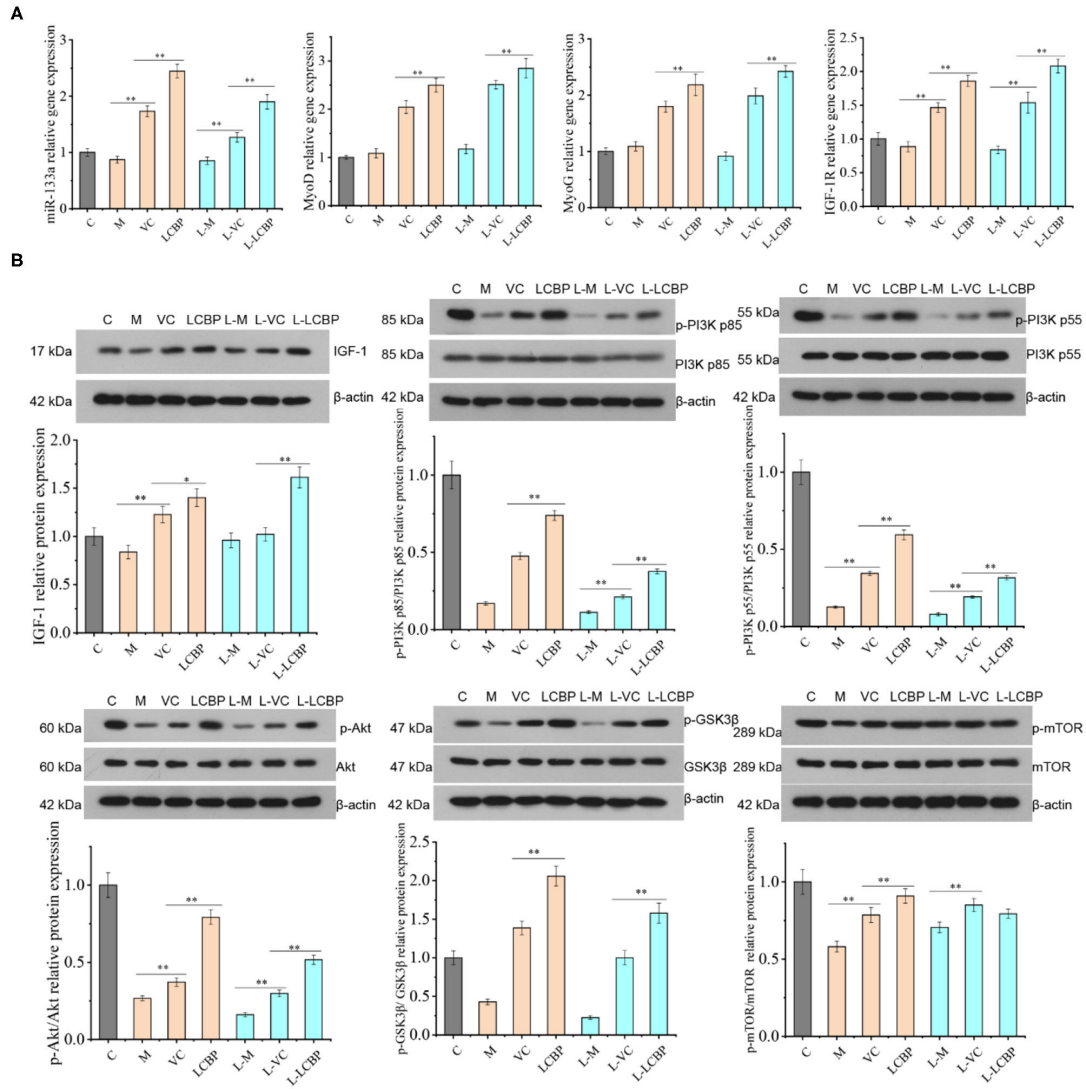
LCBP regulates the expression of proliferation-related gene and protein in skeletal muscle of chronically exercise-fatigued mice. **(A)** Real-time PCR was performed for gene expression analysis; **(B)** protein expression was detected via western blotting. M, VC and LCBP groups were treated at 25°C; L-M, L-VC and L-LCBP groups were treated at −5°C; **p < 0.01, *p < 0.05.

Long-term exercise fatigue can lead to alterations in the gene expression of mi RNA-133a, MyoD, MyoG, and IGF-1R; however, no significant difference was observed as compared with the inactive group in this study (*p* > 0.05) (Figure 8A). Compared with the model group, significant upregulation was observed in the LCBP and VC groups in terms of the expression of the four genes at 25 and −5°C (*p* < 0.01). Moreover, the upregulation of expression was more significant in the low temperature group. These data suggest that dietary LCBP supplementation can significantly aid regulation of the miRNA-133a/MyoD/MyoG/IGF-1R pathway. The effect of LCBP on the expression of proliferation-related proteins in skeletal muscle is presented in Figure 8B. The expression of the phosphorylated proteins IGF1 and GSK3β was significantly downregulated by long-term exercise fatigue *(p* < 0.01). Dietary LCBP supplementation significantly upregulated the expression of these two proteins at 25 and −5°C (*p* < 0.01) to higher levels than that observed in the inactive group (*p* < 0.01). Similarly, the phosphorylation of PI3Kp85, PI3Kp55, Akt, and mTOR was significantly decreased by fatigue associated with long-term exercise (*p* < 0.01). Dietary LCBP supplementation significantly reversed this phenomenon, even under low temperatures (*p* < 0.01), with the achievement of better results than those observed in the VC group. These results suggest that LCBP can activate the AKT/mTOR and AKT/GSK-3β signaling pathways, promote the proliferation of skeletal muscle cells, and reduce the injury of skeletal muscle that results from long-term exercise fatigue.

### The Monomer Component of LCBP Interacts With AMPKα

**Figure 9:**
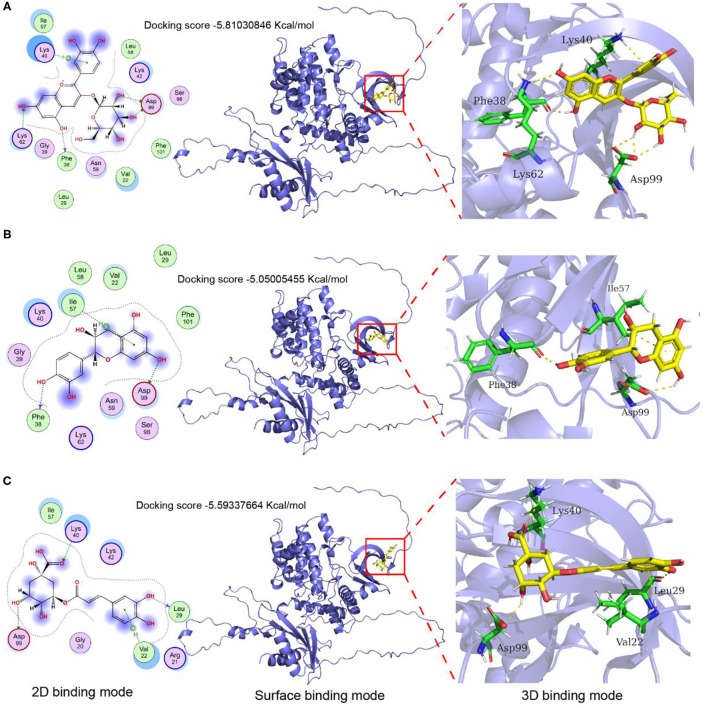
Molecular docking and protein-ligand interactions. **(A)** Cyanidin-3- glucoside binding mode with AMPKα; **(B)** Catechin binding mode with AMPKα; **(C)** Chlorogenic acid binding mode with AMPKα; The 3D structure of cyanidin 3-O-glucoside, catechin, and chlorogenic acid are shown in yellow, the structure of nearby residues is shown in green, the skeleton of the receptor protein is shown in light blue cartoon, and hydrogen bond is represented by the yellow dotted line.

The AMPK is a key molecule that regulates energy metabolism. To further verify the effective role of the major components in LCBP, the effects of cyanidin-3-glucoside, catechin, and chlorogenic acid on AMPKα were investigated using computer-aided molecular docking simulation (Figure 9). The binding pocket of cyanidin-3-glucoside and AMPKα exhibits an appropriate spatial complementarity (Figure 9A). The oxygen atoms (O3, O5) of cyanidin-3-glucoside form hydrogen bond interaction with the donor oxygen atoms (OD2, OD1) on residue Asp99. The oxygen atom on the hydroxyl group of cyanidin-3-glucoside acts as the donor to form hydrogen bond interactions with the oxygen atoms on residues Phe38 and Lys63. Cyanidin-3-glucoside forms pi-cation interactions with nitrogen atoms on residues Lys40. The combined pocket of catechin and AMPKα forms a suitable spatial complement (Figure 9B). As the donor, oxygen atoms on catechin hydroxyl group interact with the oxygen atoms on Asp99 and Glu898 to form hydrogen bonds. The oxygen atom on the hydroxyl group of catechin acts as the donor and forms hydrogen bond interaction with the nitrogen atom on the residue Phe38, and the benzene ring on catechin forms pi-H interaction with the carbon atom on the residue Ile57. The combination of chlorogenic acid and AMPKα pockets forms another suitable spatial complement (Figure 9C). The oxygen atoms on the hydroxyl group of chlorogenic acid serve as the donor to form hydrogen bond interaction with the oxygen atoms on the residues Asp99 and Leu29. The oxygen atom on the carbonyl of chlorogenic acid acts as the acceptor to form hydrogen bond interaction with the nitrogen atom on the residue Lys40, and the benzene ring on the chlorogenic acid forms pi-H interaction with the nitrogen atom on the residue Val22. The binding energies of cyanidin-3-glucoside, catechin, and chlorogenic acid to AMPKα were −5.81030846, −5.05005455, and −5.59337664 Kcal/mol, respectively. The binding energies of cyanidin-3-glucoside are close to−6, indicating that the binding affinity between ligands and proteins is substantially strong. These results demonstrate that cyanidin-3-glucoside, catechin, and chlorogenic acid could all react with AMPKα, suggesting that these three components, especially cyanidin-3-glucoside may play some important roles in contributing to the anti-fatigue activity of LCBP.

## Discussion

The performance of long-term high-intensity exercise can lead to oxidative stress and inflammation and ultimately result in fatigue. Fatigue is mainly reflected by a decrease in endurance. This study showed that dietary supplementation with LCBP could significantly prolong the running time of mice, improve endurance, and delay fatigue. Muscle glycogen and liver glycogen are direct energy sources used in movement. Under both aerobic and anaerobic conditions, glycogen undergoes rapid decomposition to produce ATP, which is an important indicator of exercise tolerance. The excessive consumption of glycogen by muscle and accumulation of lactic acid in the body results in fatigue (26).

Hb is generally considered to be one of the major factors that can improve endurance by delivering oxygen to tissues (27). Decreases in the Hb level can be considered as a biochemical indicator to help determine whether rats experience fatigue following subjection to exercise. Blood LA and serum BUN levels are also commonly used to evaluate fatigue. Strenuous exercise can result in insufficient oxygen supply or hypoxia in the tissues, leading to anaerobic glycolysis, and the accumulation of lactic acid, the product of oxidation, can directly lead to the occurrence of fatigue. Excessive exercise results in the catabolism of proteins and amino acids, resulting in an increase in serum BUN levels. The BUN content therefore generally increases in response to exercise (21). LDH and CK have always been regarded as sensitivity indices for evaluating muscle injuries. Under normal circumstances, a considerable proportion of CK is present in muscle cells, and an increase in CK levels in blood generally indicates the occurrence of muscle injury (complete or active) (25). Such injuries can lead to a significant increase in the damage to muscle cell membranes, increasing the permeability of the membranes and allowing LDH and CK to leak out of the muscle cells. This study showed that LCBP significantly prolonged the time taken by exercise-subjected mice (exercising on treadmills) to experience fatigue, and the levels of muscle glycogen and liver glycogen in the exercise-subjected groups were higher than those in the model group (*p* < 0.01), indicating that LCBP could provide sufficient energy for the body to relieve or to counter fatigue by increasing the glycogen content. This observation corroborates the results obtained in previous studies (28). However, we also found that the glucose content in LCBP and VC group did not change significantly after exhaustive exercise. This can be attributed to the longer exercise time and increased glucose consumption compared to the model group. Studies have established that exhaustive exercise has no significant effect on blood glucose level in normal rats (29). It has also been reported that exercise has no considerable effect on blood glucose levels within 1 h (30). The levels of muscle glycogen, liver glycogen, and muscle ATP decreased in the exhaustive exercise group, but increased in the nutrition supplemented group (31). Again, this also is consistent with our results.

LCBP significantly increased the levels of Hb, and decreased the levels of LDH, LA, BUN, and CK. These results suggest that the anti-fatigue effect of LCBP may be related to changes in hemoglobin, LDH, LA, and BUN concentrations. The water-soluble pectin found in okra stems (450 mg/kg diet) can prolong the swimming time of mice before exhaustion and reduce the levels of LA and BUN in serum (32), findings that are consistent with the results of this study. The green tea polyphenol EGCG has also been found to prolong the exhaustive swimming time, and decreased blood LA, serum BUN, serum CK, and MDA levels, while increasing the liver and muscle glycogen content, findings which were consistent with the results of this study. Meanwhile, the SOD, CAT, and GSH-Px activities increased (33). Long-term supplementation with a peptide derived from *Trichiurus lepturus* has been found to prolong the exhaustion swimming time of mice, to relieve fatigue, and to improve performance. The contents of liver glycogen, muscle glycogen, metabolites (BLA, BUN), CK, and other fatigue indices were significantly improved, along with the levels of antioxidants GSH-Px, SOD, CAT activity, and MDA content (31). It is suggested that increasing the amount of antioxidants within the body can help improve the fatigue associated with exercise significantly.

Long-term fatigue destroys the balance of the oxidation/antioxidant systems in the body, and the production and accumulation of ROS can lead to oxidative stress (34, 35). Antioxidant enzymes are important in preventing oxidative damage. SOD catalyzes the conversion of superoxide to hydrogen peroxide and oxygen, while GSH-Px aids the removal of hydroxyl radicals. SOD and GSH-Px also inhibit lipid peroxidation by reducing the production of MDA, thus conferring protection to cell structures from damage and reducing fatigue (36). Studies have shown that the consumption of considerable amounts of antioxidants is positively associated with a reduction in exercise-induced muscle damage (37). The oxidative damage induced by exercise fatigue can be prevented by optimizing nutrition, especially by increasing the dietary antioxidant content (38). Salidroside, acting as an exogenous antioxidant, can effectively prevent and delay oxidative damage, reduce MDA levels, enhance the activities of the antioxidant enzymes SOD, CAT and GSH-Px, while maintaining its own antioxidant activity and stabilizing Ca^2+^ homeostasis in cells (39). Amarkand extract is rich in polyphenols and can significantly extend swimming endurance time by mitigating oxidative stress and by improving various injuries related to fatigue, leading to an increase in the aerobic metabolism of glucose and endurance (40). LCBP can increase the levels of SOD, GSH-Px, and CAT in serum, and reduce MDA and ROS levels, improving the total antioxidant capacity of the body and reducing oxidative stress. Studies investigating the expression regulation of oxidative stress-related proteins (NQO1 and HO-1) have also indicated the occurrence of this phenomenon. These findings suggest that a reduction in oxidative stress may be associated with an enhanced LCBP performance.

In addition to oxidative stress, the increased secretion of inflammatory factors further reduces exercise capacity (41). The induced nitric oxide synthase/nitric oxide (iNOS/NO) signaling pathway and inflammatory factors play an important role in the pathogenesis of exercise fatigue. Presently, it is generally believed that the performance of high-intensity exercise can activate iNOS in endothelial and skeletal muscles, leading to an increase in the production of NO. The excessive production of NO during inflammation reduces the ability of muscles to contract, leading to a reduction in exercise ability (42). Chronic exercise fatigue increases the expression of iNOS and proinflammatory cytokines (IL-1β, IL-6, and TNF-α). Studies suggest that Mongolian warm acupuncture can help relieve the fatigue that results from chronic exhaustive swimming, and that this may be related to a decrease in the inflammatory response and the expression of iNOS (43), The results of this study indicate that LCBP decreases the expression of iNOS and the production of NO in skeletal muscles of mice following subjection to treadmill exercise, contributing to the improvement in exercise performance. Additionally, LCBP supplementation can significantly reduce the inflammatory response that is induced by long-term exercise fatigue. Studies have shown that supplementation with lactic acid bacteria can improve muscle and energy imbalances and other injuries that are associated with triathlon training by improving oxidative stress and inflammatory responses (44). The consumption of ginseng extract in water has been found to improve energy metabolic abnormalities, oxidative stress, lipid peroxidation, and inflammatory reactions, and exerts a strong anti-exercise-induced fatigue effect (45). The results of this study indicate that LCBP supplementation can alleviate long-term exercise fatigue, which is closely related to the reduction of oxidative stress and inflammatory responses.

Exercise-induced oxidative stress is one of the main causes of apoptosis in skeletal muscle cells (46). The performance of intense exercise significantly increases the uptake of oxygen, aids production of ROS, and causes extensive DNA damage, leading to the infliction of damage on skeletal muscle fibers, which can lead to aging and death. As mitochondria is considered a source and a target of ROS, the production of mitochondrial reactive oxygen species can induce an increase in mitochondrial permeability, opening of conversion pores, reduction in ATP production, and upregulation of the release of Cyt C to activate pathways that lead to mitochondrial death (47). Mitochondrial apoptosis is considered one of the most common forms of programmed cell death. The Bcl-2 family proteins are involved in this process; this includes the pro-apoptotic proteins Bax and Bak, along with the anti-apoptotic proteins Bcl-2, with bcl-2 and Bax regarded as the most important regulatory factors in the bcl-2 family. Bax has been found to induce mitochondrial permeability and the release of Cyt C (48). The released Cyt C from mitochondria into the cytoplasm directly establishes interaction with Apaf-1 in the cytoplasm, causing Apaf-1 to undergo conformational changes and revelation of its cystease recruitment domain, leading to the formation of ATP-dependent macromolecular complexes in the apoptotic complex. Apoptotic cells activate Caspase-9 and Caspase-3 downstream (49). Studies have shown that Anwulignan significantly upregulates the expression of the antioxidant and anti-apoptotic regulatory proteins NRF2 and Bcl2, significantly improving exercise tolerance (50). Salidroside inhibits the production of ROS and NO, thus regulating the ratio of Bcl-2 to Bax and reducing the release of Cyt C, which in turn inhibits the activation of Caspase-3, Caspase-6, and Caspase-9 to ultimately inhibit apoptosis (51). These results were consistent with our findings, indicating that LCBP could significantly upregulate the expression of the Cyt C protein in the mitochondria (*p* < 0.01) and inhibit the release of the Cyt C protein into the cytoplasm. These results suggest that LCBP can provide protection to skeletal muscles and increase exercise performance by reducing ROS levels and the mitochondrial apoptosis pathways.

ROS has been considered the main cause of oxidative stress, inflammation, and apoptosis in skeletal muscle, and thus we further studied ROS production. The sources of ROS include both mitochondria and NOX. Nox2 is considered the main non-mitochondrial enzyme system that is involved in the generation of ROS (52, 53). Nox2, its regulatory subunit, and Nox4 can be found in muscle fibers and the sarcoplasmic reticulum. The fatigue that results from exercise promotes the production of ROS in skeletal muscles through the PKC-NOX2 pathway. PKCα was found to upregulate Nox4 expression (54) and one-off exercise activated the NOX2-mediated generation of ROS in skeletal muscle, providing direct evidence of the role of the Nox production pathway in ROS production, indicating the direct consequences of exercise (55) We therefore determined the expression of the different types of Nox that are known to aid the production of ROS during exercise. LCBP was found to significantly downregulate the protein expression of Nox2 and Nox4 in skeletal muscle. Further study indicated that the co-immunoprecipitation of PKCα and Nox4 led to the formation of PKCα-Nox2 and PKCα-Nox4 complexes in skeletal muscles after the performance of long-term exercise, indicating the establishment of an interaction between the two. Exercise fatigue stimulates an increase in the production of this complex, mediating the production of exercise-induced ROS. The production of the weakened complex may further mediate the inhibition of ROS generation during exercise. These results suggest that the anti-fatigue effect of LCBP can help reduce ROS production in skeletal muscle by regulating the PKCα-Nox2/ Nox4 pathway.

The AMPK/PGC1-α pathway is deemed one of the main pathways that promotes mitochondrial synthesis. Adenosine-activated protein kinase (AMPK) is regarded as the master switch of energy metabolism within the body, and phosphorylation activates the synthesis of mitochondria (56). PGC-1α can help stimulate mitochondrial biosynthesis by binding nuclear respiratory factor 1 (NRF-1), the peroxide proliferator activator receptor PPARs, and muscle cell enhancer factor 2 (57). Increasing the oxidative phosphorylation ability of skeletal muscle cells can help enhance the anti-fatigue effect. This is closely related to an increase in the levels of mitochondrial protein and its components, and the promotion of mitochondrial biosynthesis. The overexpression of PGC-1α in skeletal muscle can lead to an increase the number of mitochondria, thus increasing the oxidative phosphorylation capacity of mitochondria in tissue (25). Additionally, PGC-1 transcription is also stimulated by the external environment. Previous studies have shown that exposure to low temperatures can induce the increased expression of PGC-1α in muscle tissues (58), a finding which is consistent with the finding obtained in this study. Studies have also shown that a peptide derived from the sea cucumber can inhibit oxidative stress state and can enhance the mitochondrial function in mice by regulating the NRF2/ARE and AMPK/PGC-1α signaling pathways, thus playing an anti-fatigue role (59). Through mechanism studies, it was found that citrus tangeretin increased the phosphorylation of LKB1 kinase Ser428 through energy regulation, and then activated the mitochondrial biogenesis AMPK-PGC1α-NRF1-TFAM signaling pathway, which played an active role in regulating mitochondrial biogenesis and delayed the occurrence of fatigue (60). Lycopene activates the protein expression of skeletal muscle metabolic regulators (AMPK, NRF1, and PGC-1α) and promotes mitochondrial biosynthesis, thereby enhancing exercise endurance (61). These results suggest that LCBP can improve physical performance, and the occurrence of antagonistic fatigue is closely related to the activation of the skeletal muscle AMPK-PGC1α-NRF1-TFAM signal axis and the enhancement of mitochondrial biogenesis in skeletal muscle.

The proliferation of skeletal muscle cells is crucial for the recovery of muscle that has been injured as a result of long-term exercise fatigue. Studies have found that the upstream region of miRNA-133a is regulated by the myogenic regulatory factors MyoD and MyoG, while the downstream region is involved in regulating the proliferation and differentiation of myoblasts, inducing muscle growth by regulating IGF-1R expression and acting on the PI3K/AKT signaling pathway (62). Supplementation with guanidinoacetic acid can induce the activation of the AKT/mTOR/S6K signaling pathway through miR133a-3p, leading to the promotion of myoblast differentiation and the growth of skeletal muscle (63). The PI3K/Akt pathway is deemed a key pathway in IGF-1 activation. Akt promotes the activation of glycogen synthase kinase 3β (GSK3β) and mTOR, while the phosphorylation of GSK3β reduces the phosphorylation level of eukaryotic initiation factor 2 at Serine 535, facilitating translation initiation and protein synthesis (64). Exercise results in muscle enlargement and improved muscle regeneration, and, surprisingly, inhibits the Akt-MTOR pathway of muscle satellite cells (65). This observation is consistent with our findings. Long-term exercise increases performance but inhibits the Akt-mTOR pathway, while LCBP significantly activates the PI3K/Akt/GSK3β/mTOR pathway. These results suggest that the anti-fatigue effect of LCBP can be achieved by promoting skeletal muscle cell proliferation through the miRNA-133A/IGF-1/PI3K/Akt/mTOR pathway.

In addition, cyanidin-3-glucoside, catechin, and chlorogenic acid, the primary components of LCBP, may play major roles in improving exercise-induced fatigue in LCBP. Cyanidin-3-glucoside enhances the performance during exercise by increasing lactate metabolism, and upregulating the phosphorylation of AMPK (66). Previous report demonstrate that catechins involved in liver ammonia metabolism substantially improves endurance performance in mice (67). Currently, there are not many other studies reporting a single component responsible for fatigue improvement due to the lack of a suitable cellular model. Therefore, we used the computer-aided molecular docking simulation to demonstrate the binding of three monomers to AMPKα. Therefore, the anti-fatigue effect of LCBP can be attributed to cyanidin-3-glucoside, catechin, and chlorogenic acid, which is mainly the result of the single or synergistic action of its three monomer components.

Moreover, studies have shown that supplementing the diet with antioxidants, such as vitamins C and E, can improve athletic performance (68). In this study, VC was used as a positive control to compare the effect and mechanism of LCBP in alleviating exercise fatigue in mice. The results showed that VC significantly increased the exhaustive exercise time of mice compared with the M group (*p* < 0.05). It has been confirmed that VC can relieve exercise fatigue and improve exercise performance, and LCBP and VC have similar mechanisms. However, different studies have shown conflicting results whether oral administration of VC decreases muscle mitochondrial biogenesis (69). This may be related to different doses of VC and exercise training methods.

## Conclusions

LCBP increased energy storage (in the form of liver glycogen and muscle glycogen), reduced the accumulation of metabolic products (BUN and LA), reduced oxidative stress and inflammatory responses, inhibited apoptosis in the skeletal muscle cells, promoted cell proliferation, reduced the muscle injury associated with long-term exercise fatigue, decreased fatigue, and improved sports performance. The main mechanisms involved were ROS reduction and the induction of the mitochondrial apoptosis pathway, which conferred protection to skeletal muscle. The production of ROS in skeletal muscle is reduced by regulation of the PKCα-Nox2/ Nox4 pathway and activation of the AMPK-PGC1α -NRF1-TFAM signal axis in skeletal muscle can improve the mitochondrial biogenesis in skeletal muscle. Activation of the miRNA-133a/ IGF-1/PI3K/Akt/mTOR pathway promotes skeletal muscle cell proliferation. Although exercising in a cold environment (-5°C) was found to exacerbate injuries, the fatigue relief associated with LCBP supplementation was not affected. In addition, we confirmed that the mechanism of VC as an antioxidant is similar to that of LCBP in alleviating exercise fatigue. Therefore, LCBP is a promising nutritional agent that can be considered for relieving exercise fatigue. Additionally, the pathological status (H&E staining) of the small intestine showed that long-term fatigue and exercise also caused intestinal inflammation, which might result from oxidative stress and increased inflammation; however, further study should be conducted to ascertain whether this could be related to the presence of specific intestinal microbes.

## Data Availability Statement

The original contributions presented in the study are included in the article/Supplementary Material, further inquiries can be directed to the corresponding authors.

## Ethics Statement

The animal study was reviewed and approved by Ethics Committee of Hebei Normal University of Science and Technology.

## Author Contributions

SL and DS: conceptualization, supervision, and funding acquisition. SL and FM: methodology and writing—review and editing. DZ and JZ: investigation and data curation. SG: formal analysis. SL: writing—original draft. XC: critical revision of the manuscript. All authors contributed to the article and approved the submitted version.

## Funding

This work was funded by the Science and Technology Support Program of Hebei in China (Nos. 19975708D and 215A7103D).

## Conflict of Interest

The authors declare that the research was conducted in the absence of any commercial or financial relationships that could be construed as a potential conflict of interest.

## Publisher's Note

All claims expressed in this article are solely those of the authors and do not necessarily represent those of their affiliated organizations, or those of the publisher, the editors and the reviewers. Any product that may be evaluated in this article, or claim that may be made by its manufacturer, is not guaranteed or endorsed by the publisher.
