# Supplementary figures and images for: Lonicera caerulea Berry Polyphenols Extract Alleviates Exercise Fatigue in Mice by Reducing Oxidative Stress, Inflammation, Skeletal Muscle Cell Apoptosis, and by Increasing Cell Proliferation (part 1 of 2)
Source: Front Nutr. 2022 Mar 9;9:853225. doi: 10.3389/fnut.2022.853225 (PMC8959458; doi:10.3389/fnut.2022.853225)

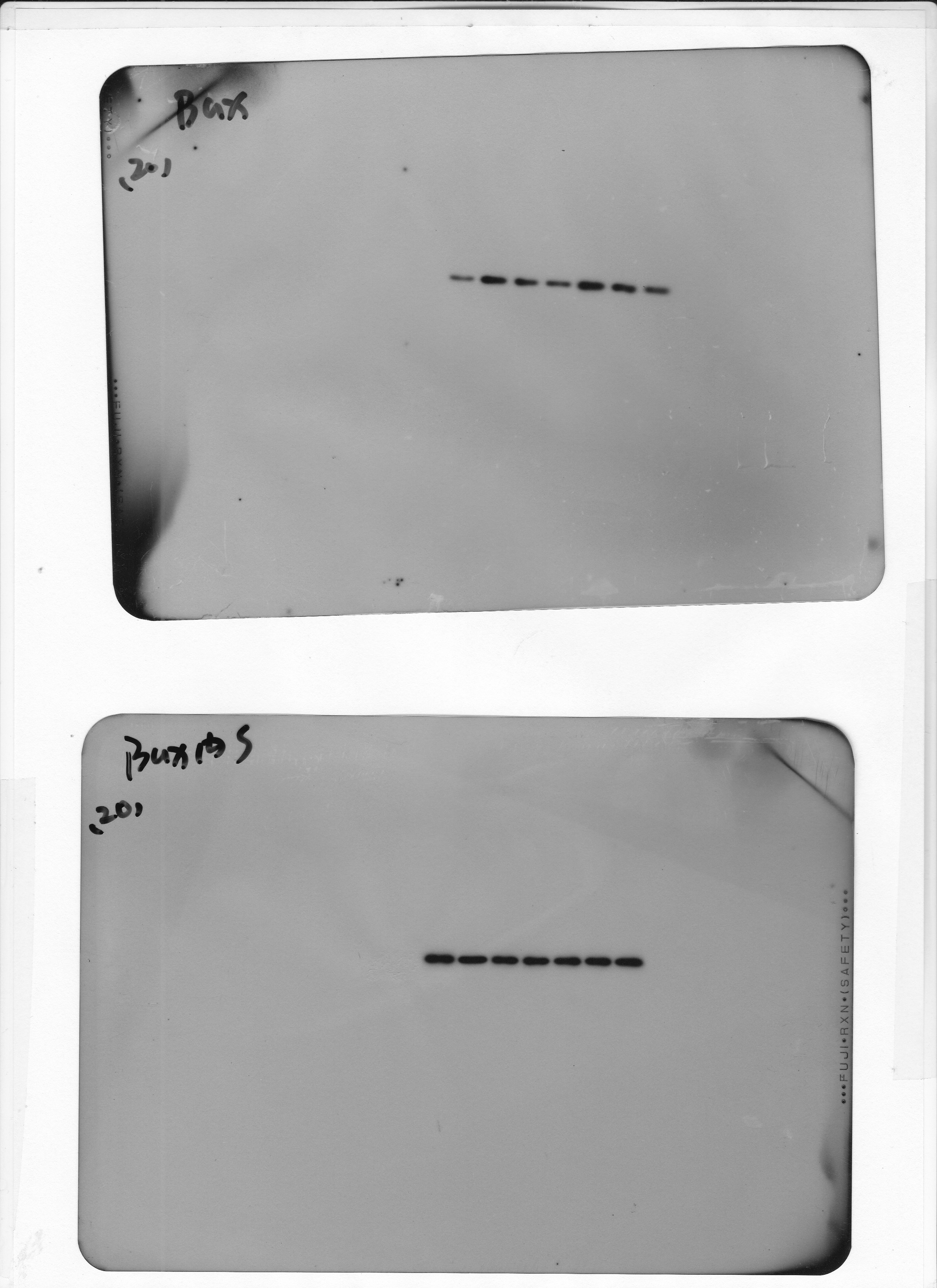

Supplement: Supplementary file 1 [file Data_Sheet_1.ZIP › Western blot figure/Bax.jpg]

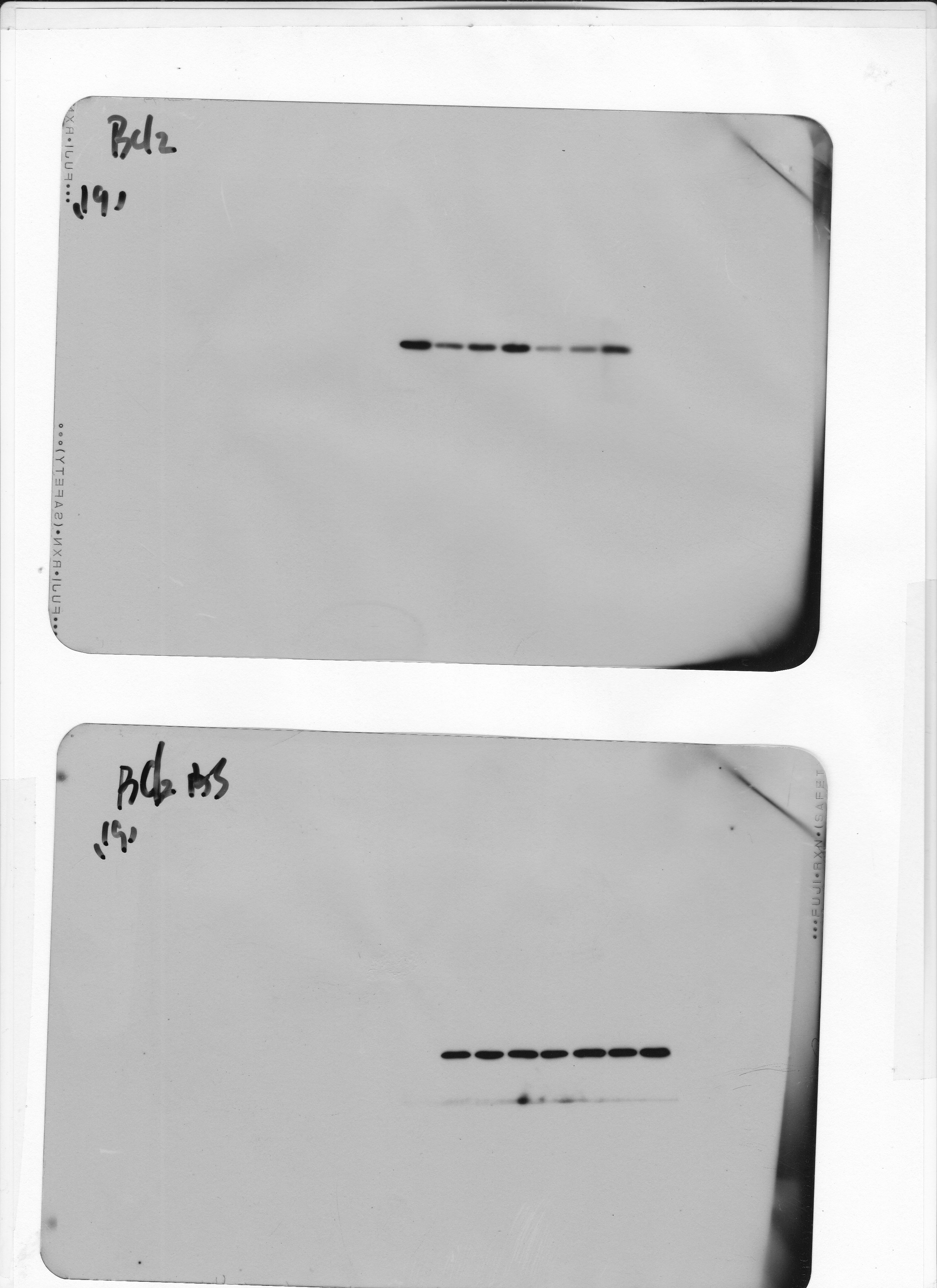

Supplement: Supplementary file 1 [file Data_Sheet_1.ZIP › Western blot figure/Bcl2.jpg]

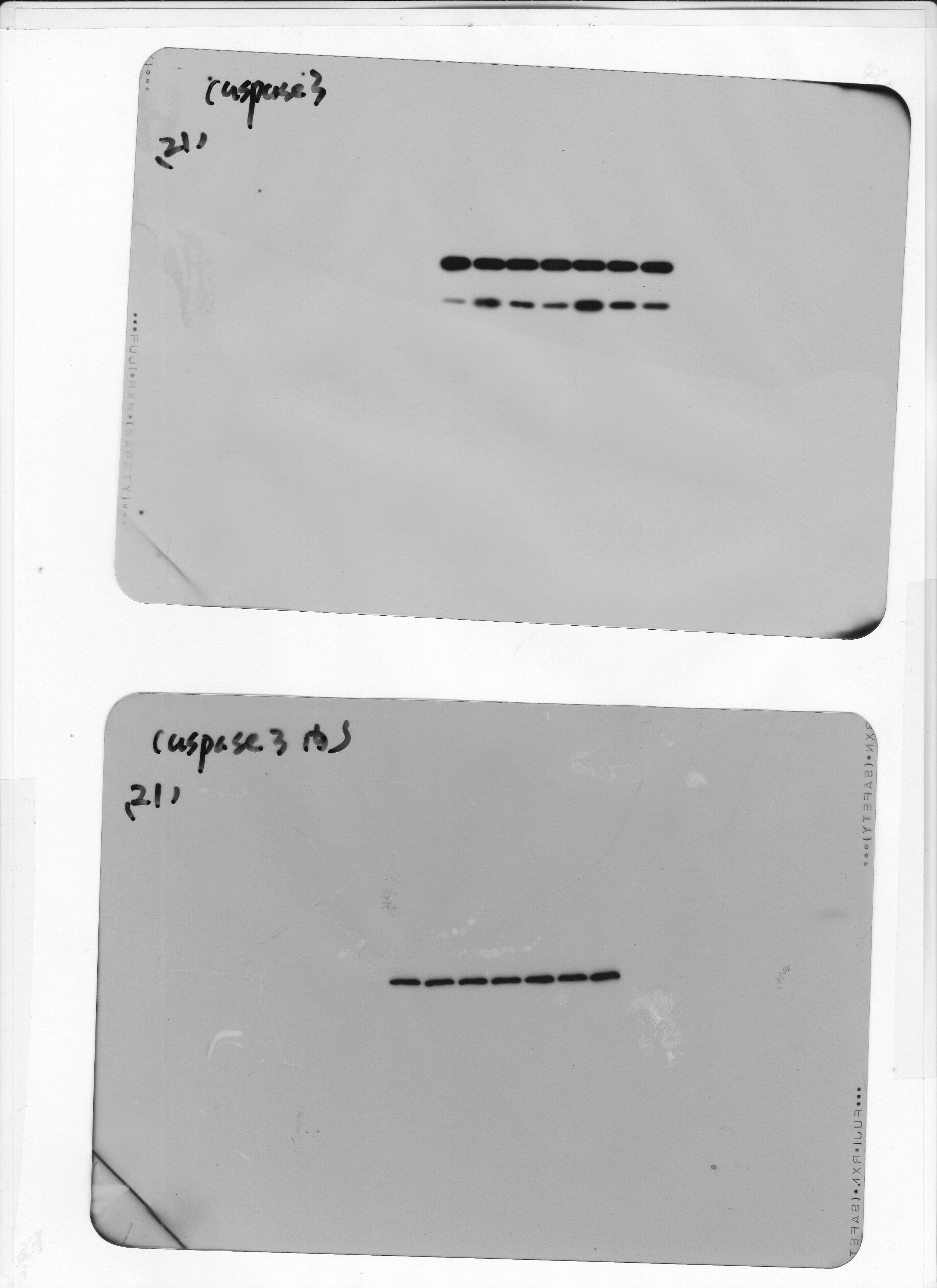

Supplement: Supplementary file 1 [file Data_Sheet_1.ZIP › Western blot figure/Caspase3.jpg]

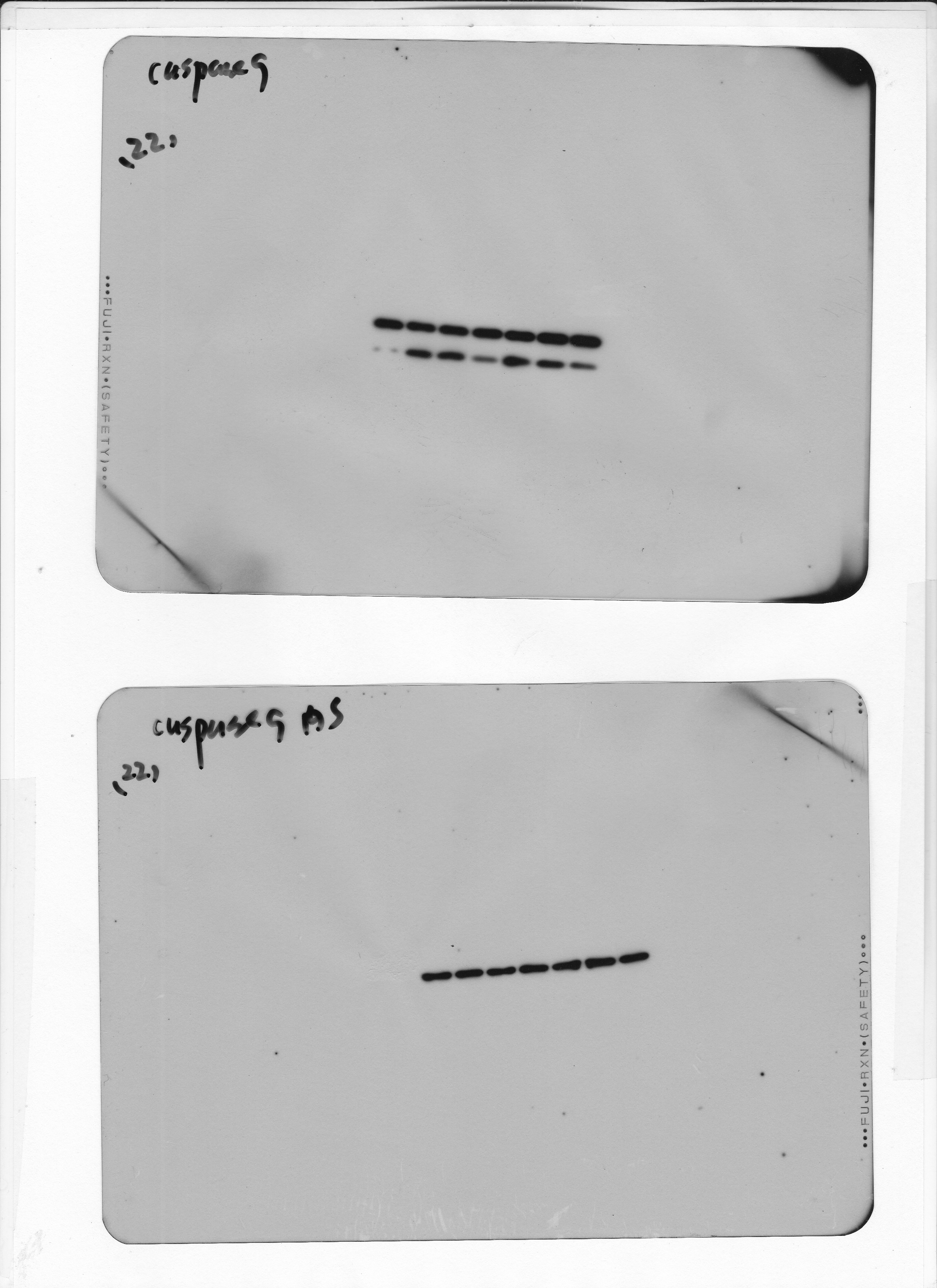

Supplement: Supplementary file 1 [file Data_Sheet_1.ZIP › Western blot figure/Caspase9.jpg]

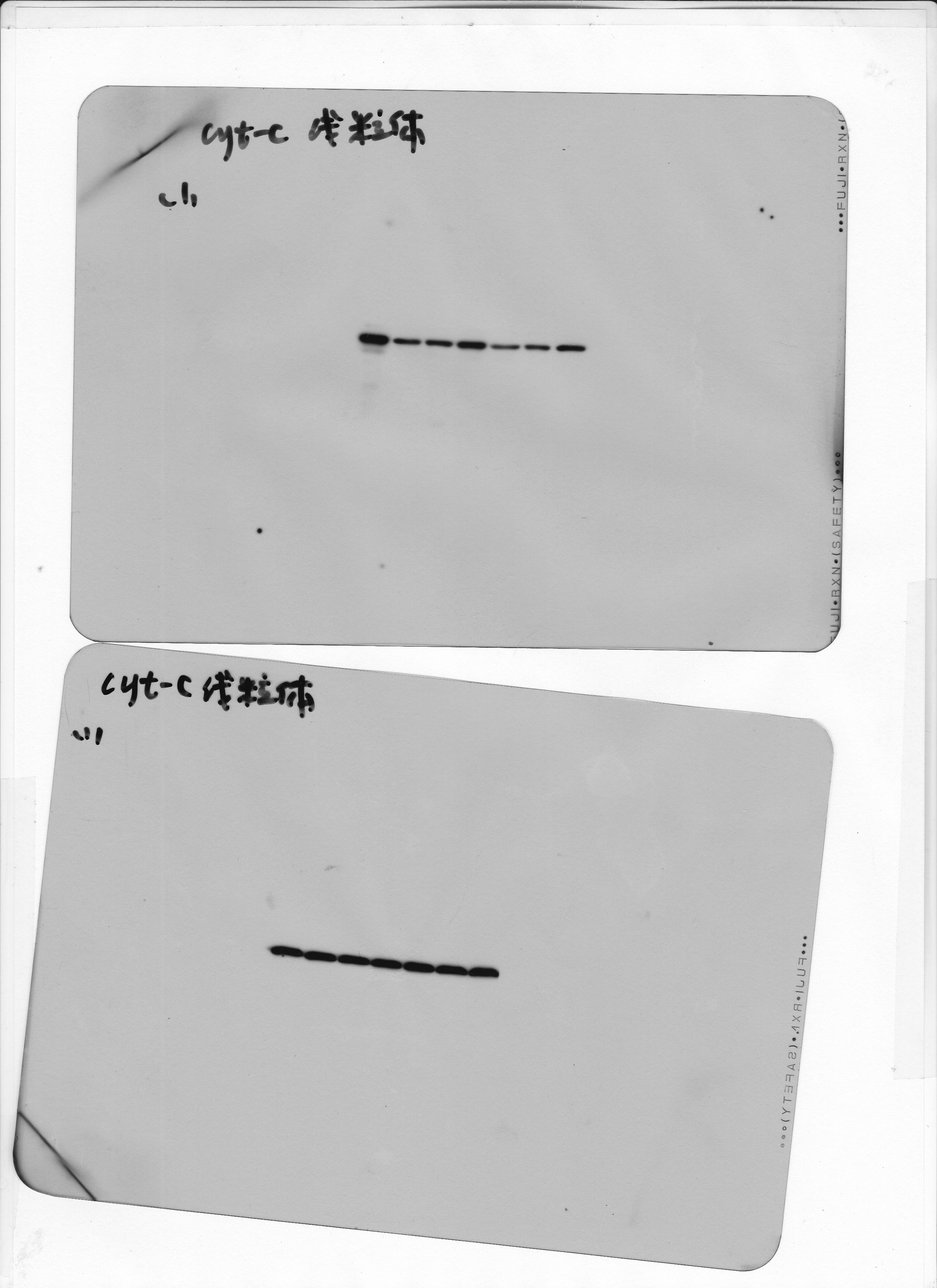

Supplement: Supplementary file 1 [file Data_Sheet_1.ZIP › Western blot figure/cytc.jpg]

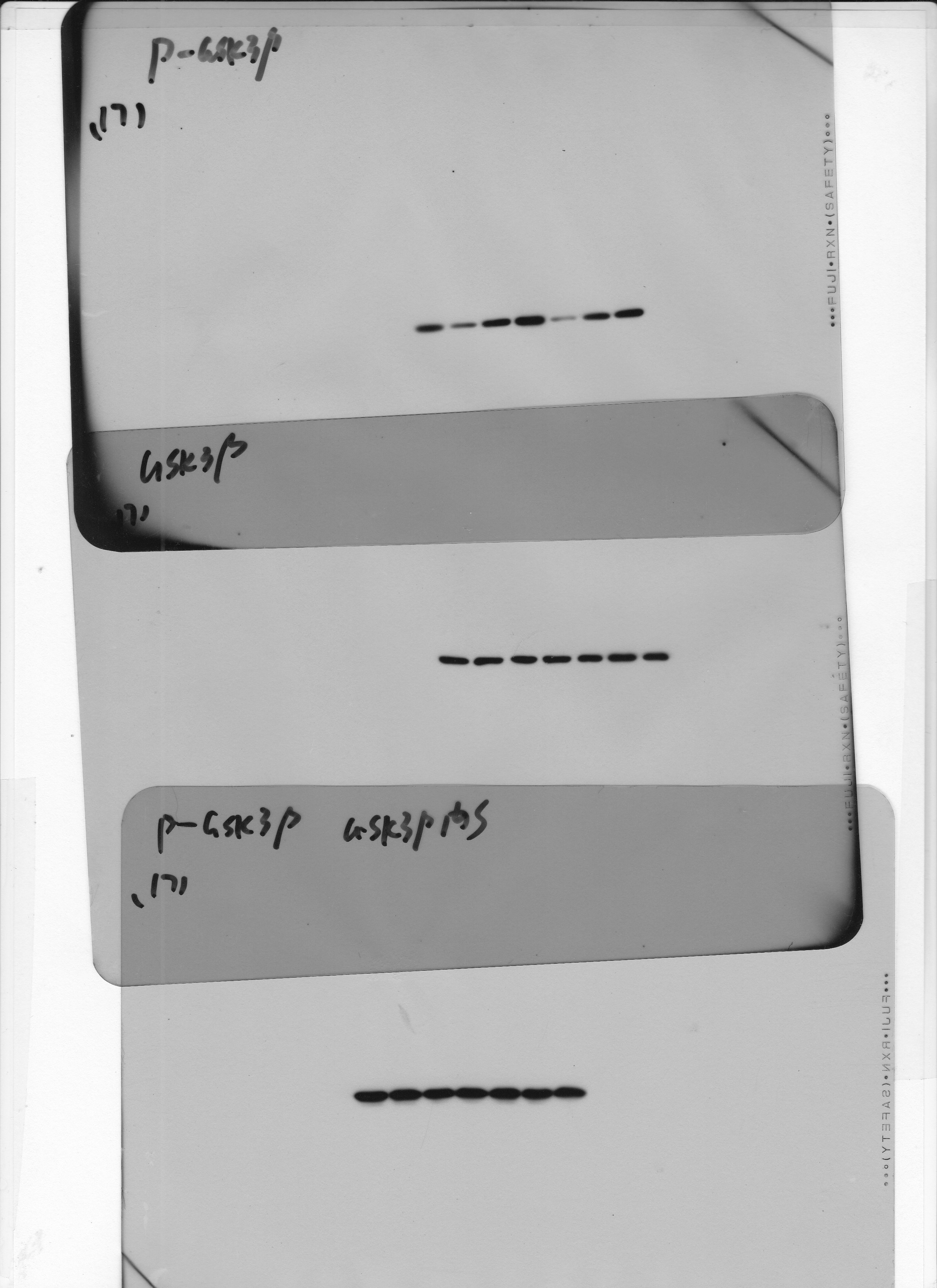

Supplement: Supplementary file 1 [file Data_Sheet_1.ZIP › Western blot figure/GSK3β.jpg]

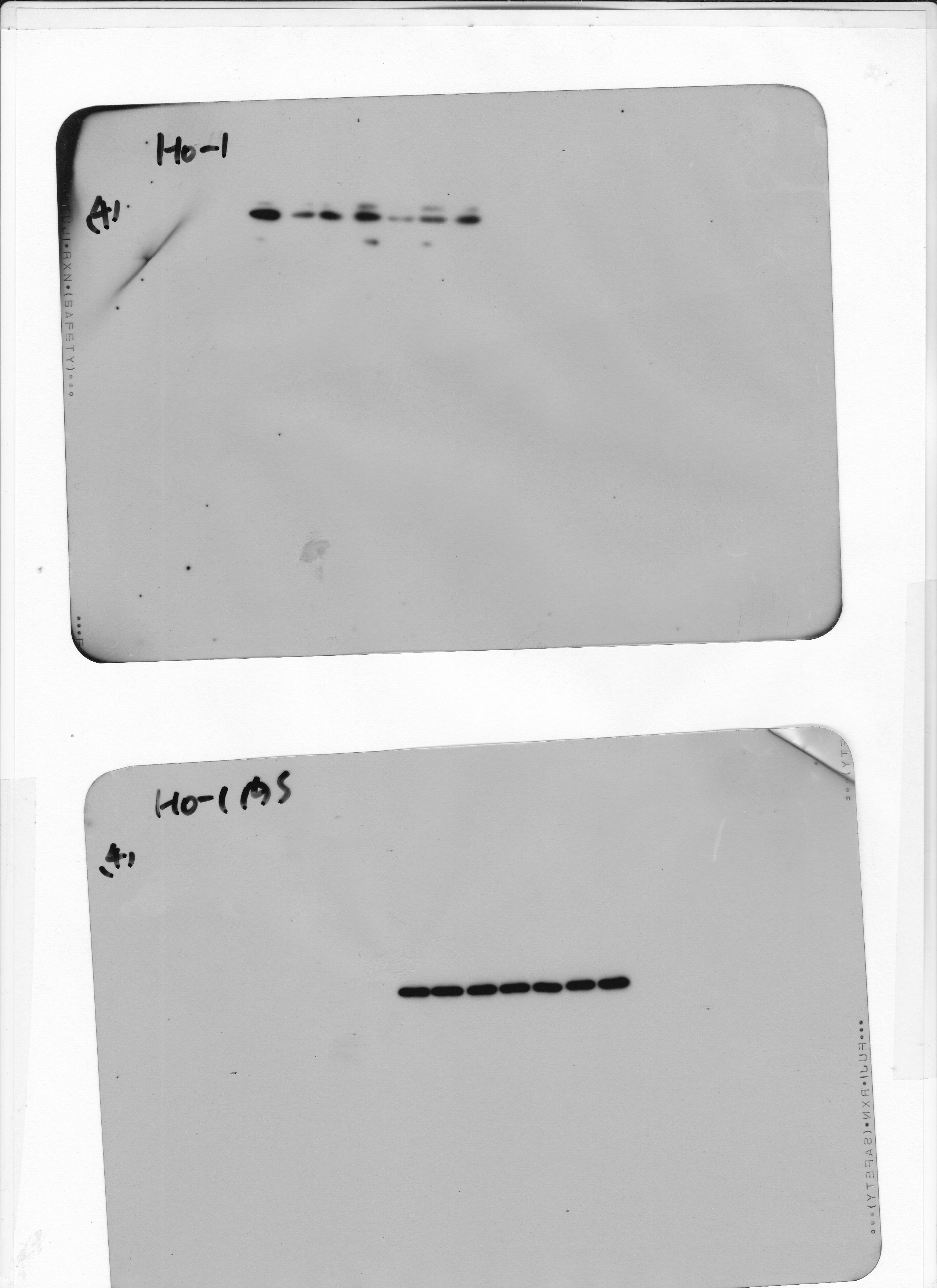

Supplement: Supplementary file 1 [file Data_Sheet_1.ZIP › Western blot figure/HO-1.jpg]

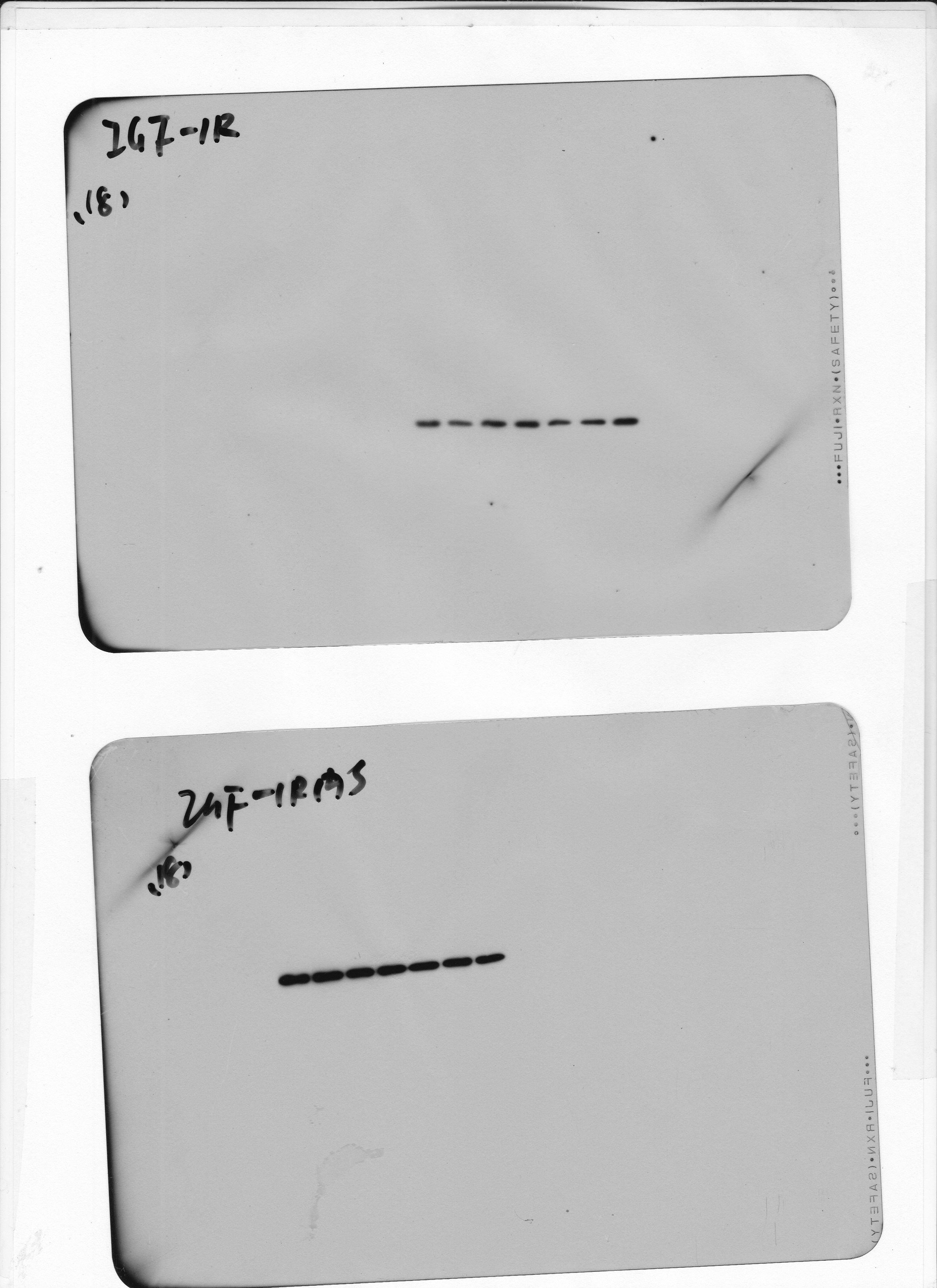

Supplement: Supplementary file 1 [file Data_Sheet_1.ZIP › Western blot figure/IGF-1.jpg]

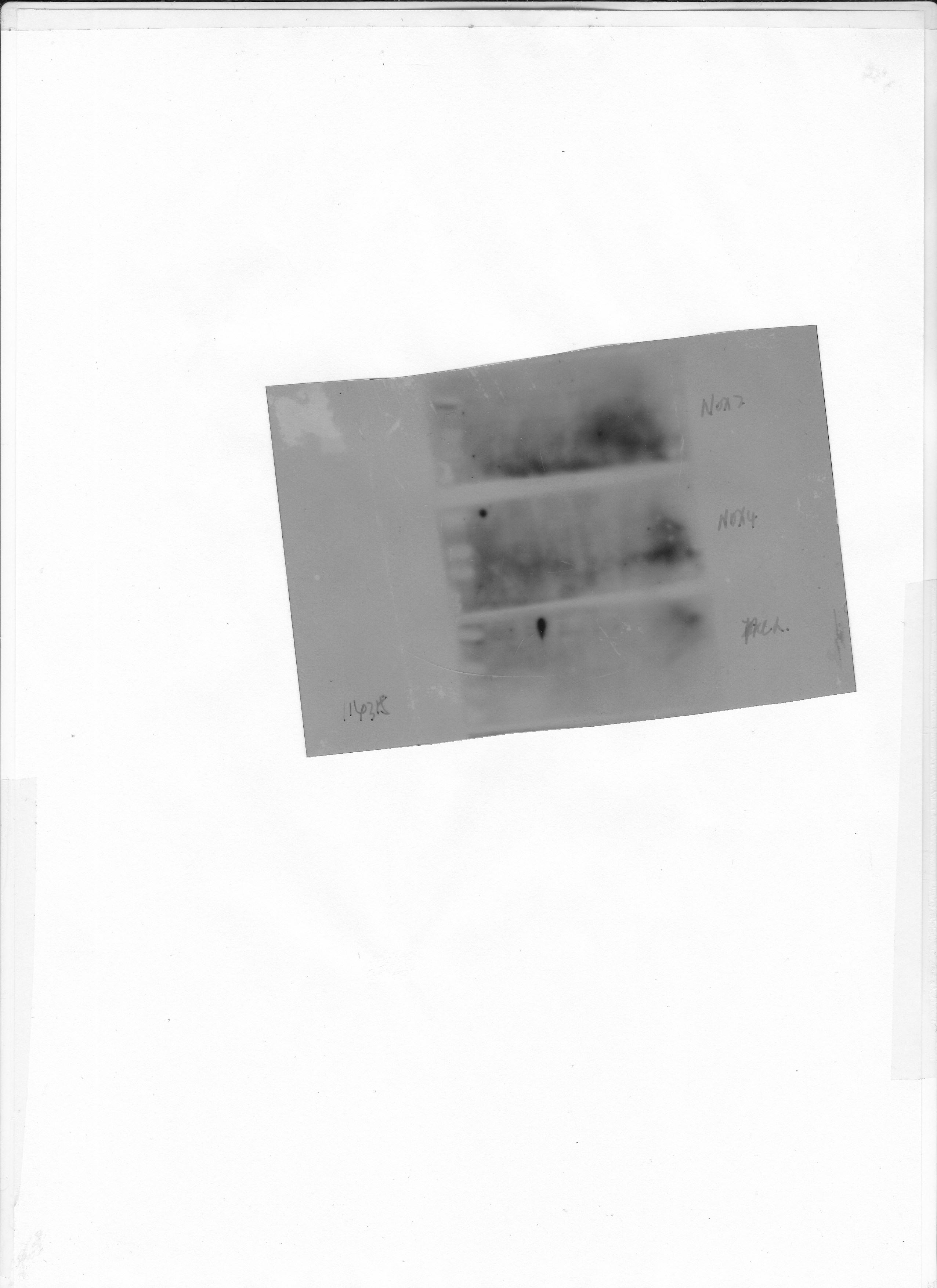

Supplement: Supplementary file 1 [file Data_Sheet_1.ZIP › Western blot figure/IgG.jpg]

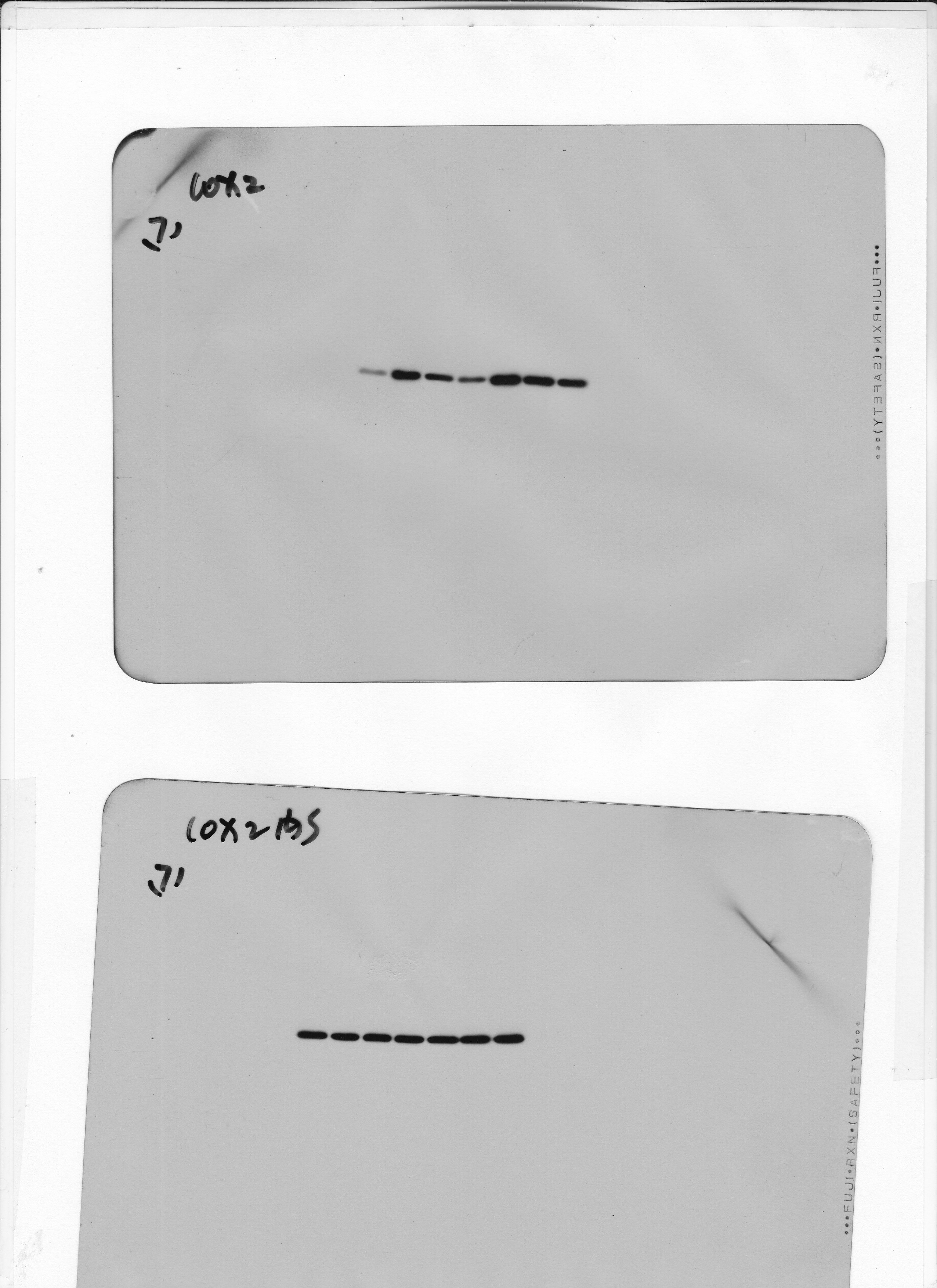

Supplement: Supplementary file 1 [file Data_Sheet_1.ZIP › Western blot figure/INOS.jpg]

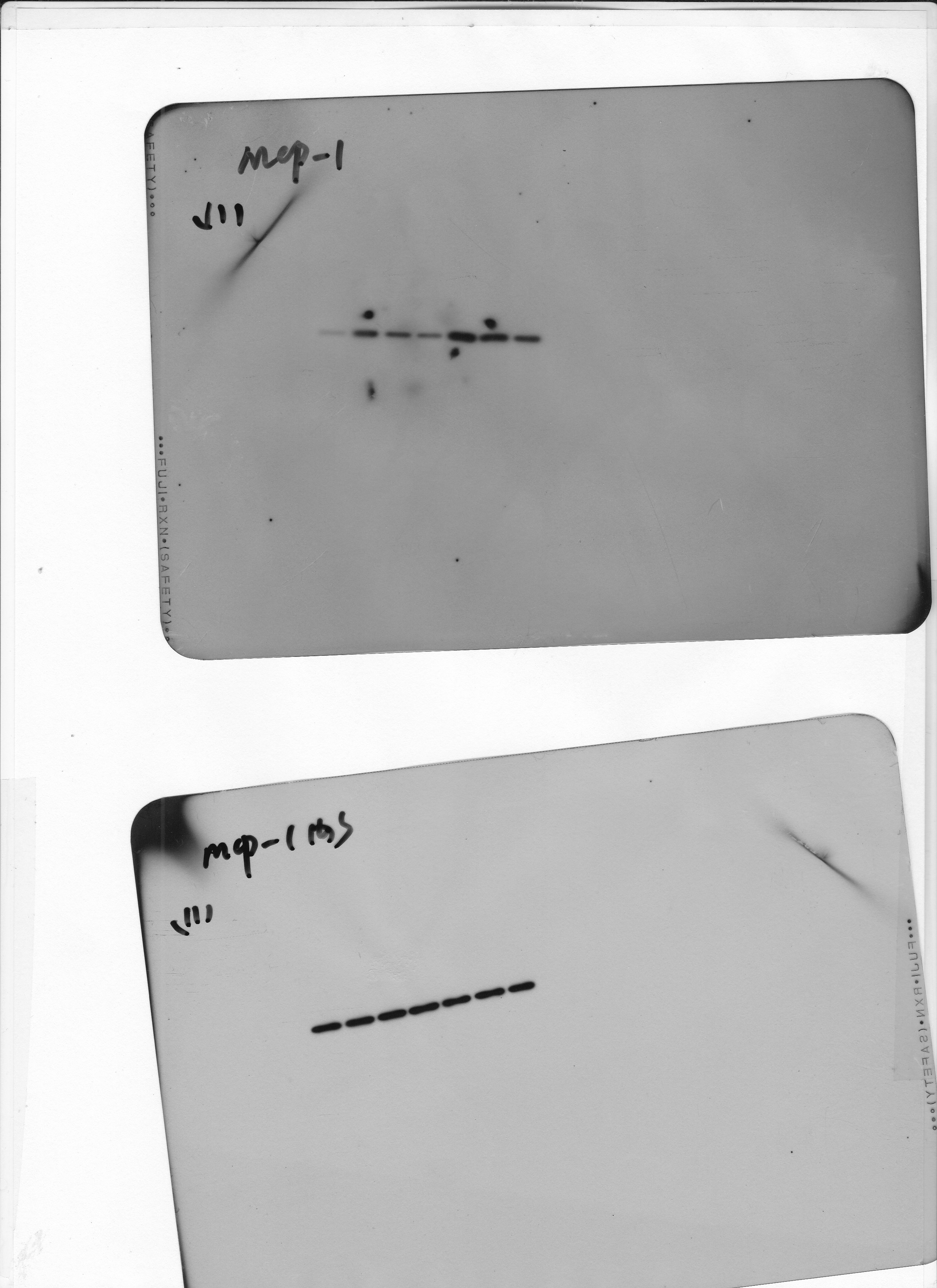

Supplement: Supplementary file 1 [file Data_Sheet_1.ZIP › Western blot figure/MCP-1.jpg]

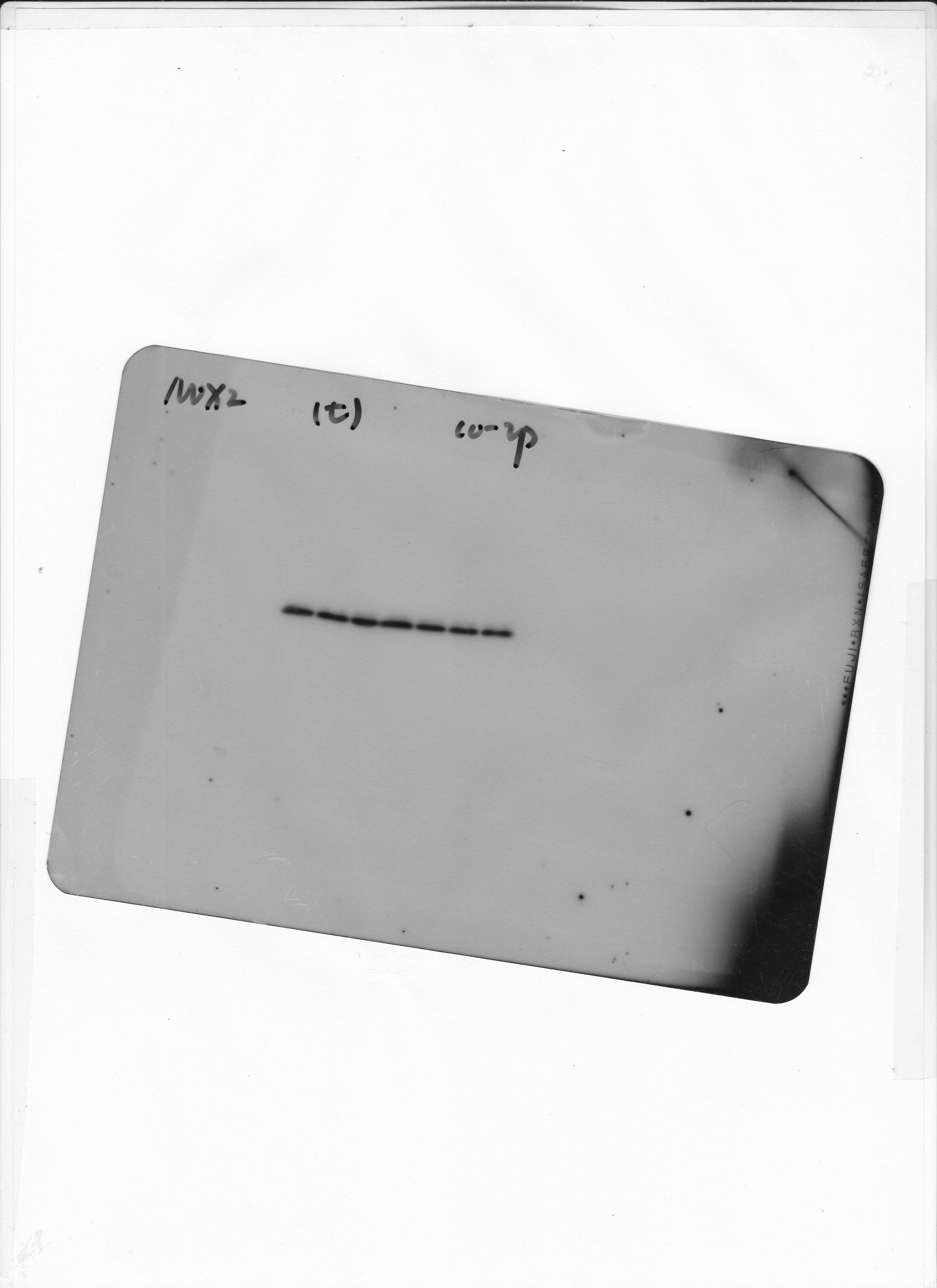

Supplement: Supplementary file 1 [file Data_Sheet_1.ZIP › Western blot figure/Nox2 (2).jpg]

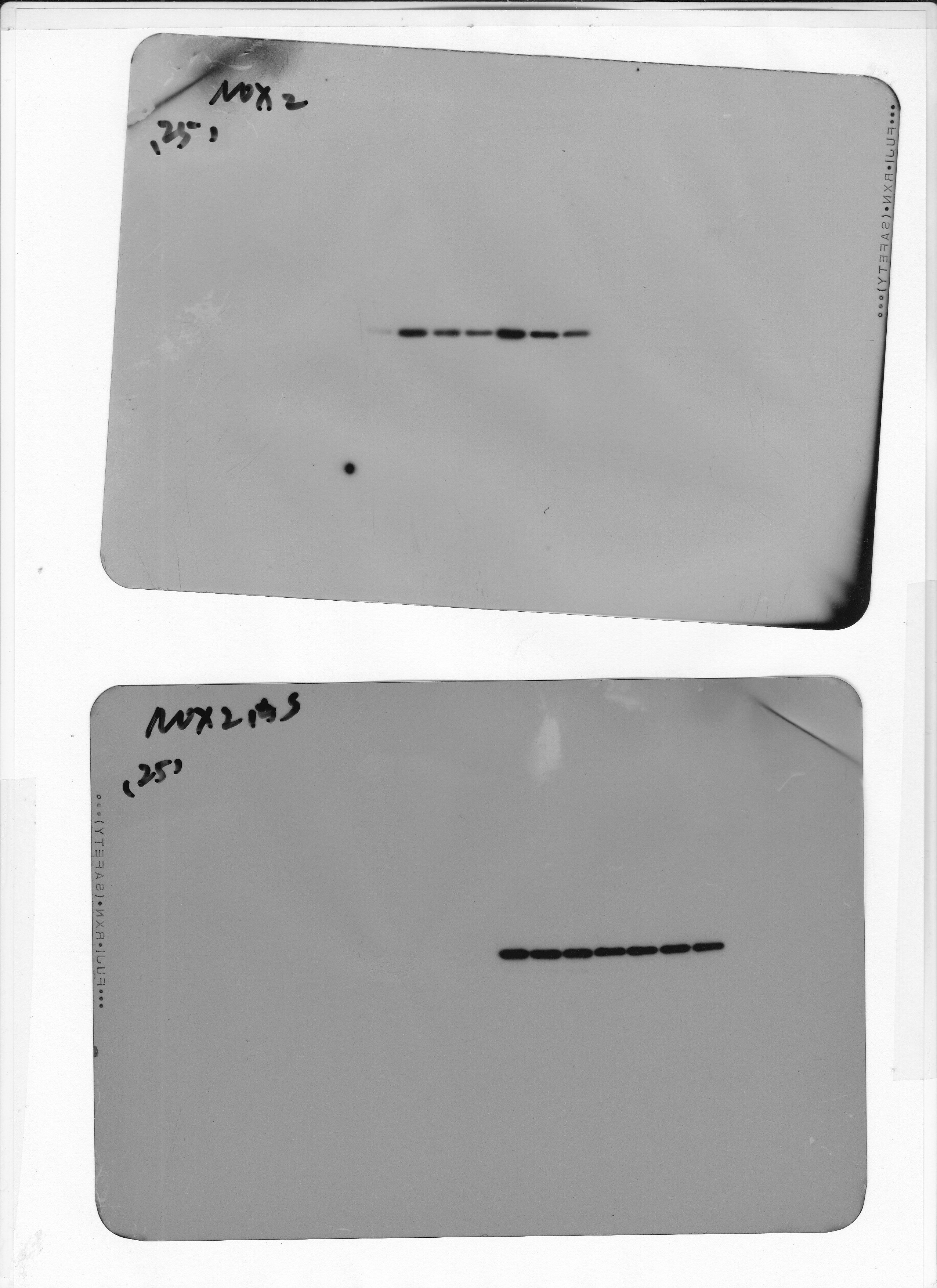

Supplement: Supplementary file 1 [file Data_Sheet_1.ZIP › Western blot figure/Nox2.jpg]

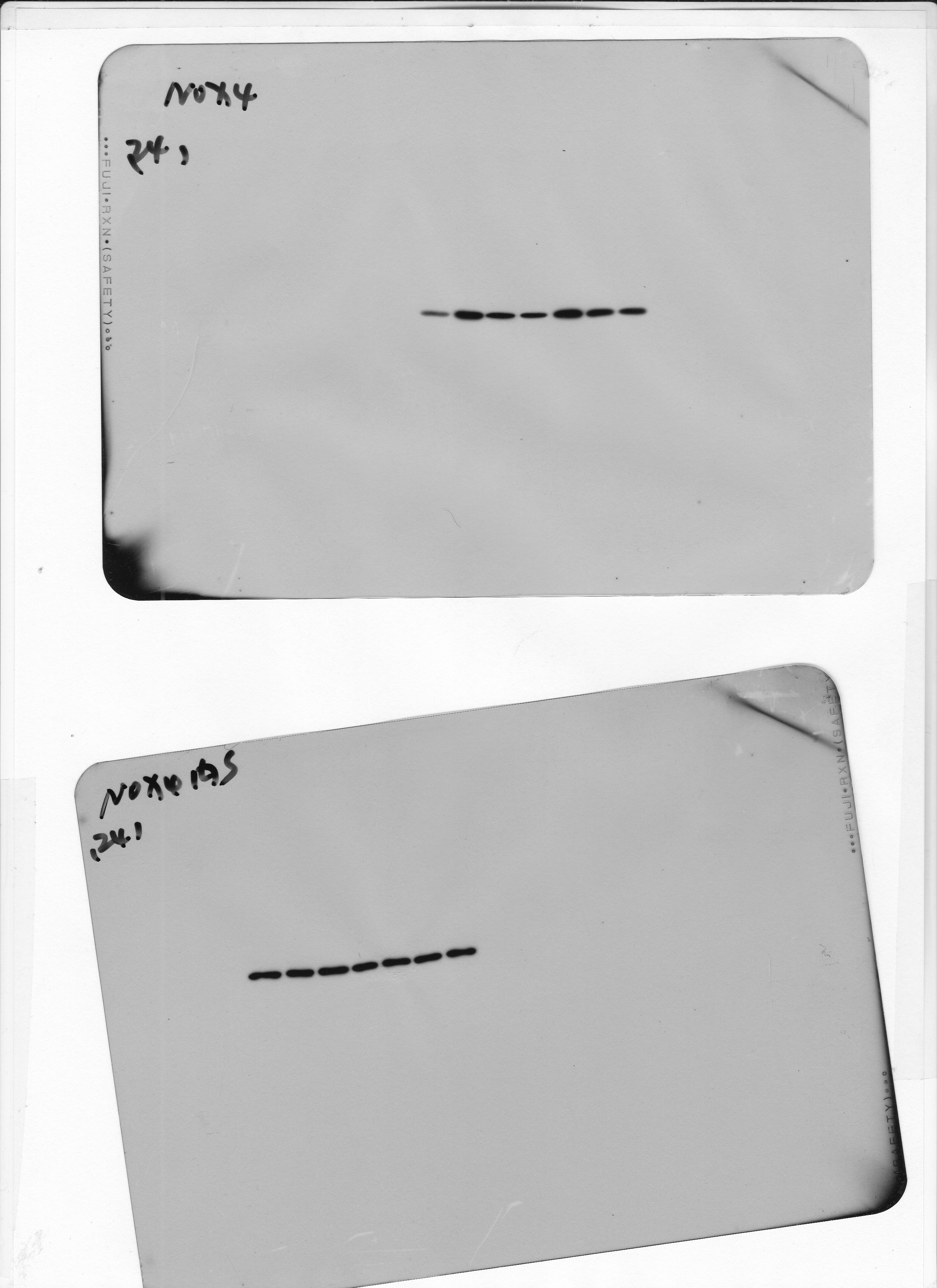

Supplement: Supplementary file 1 [file Data_Sheet_1.ZIP › Western blot figure/Nox4.jpg]

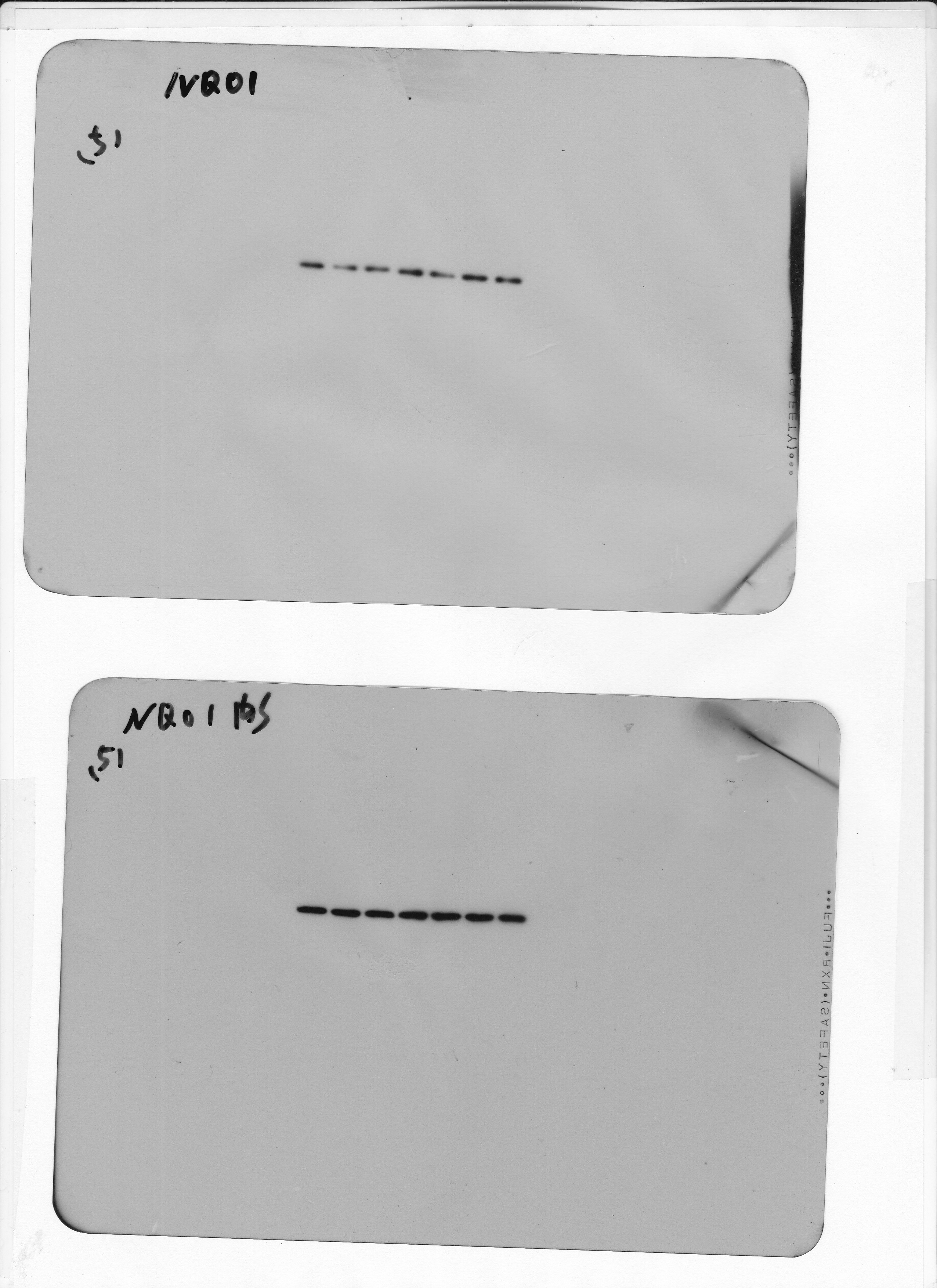

Supplement: Supplementary file 1 [file Data_Sheet_1.ZIP › Western blot figure/NQO1.jpg]

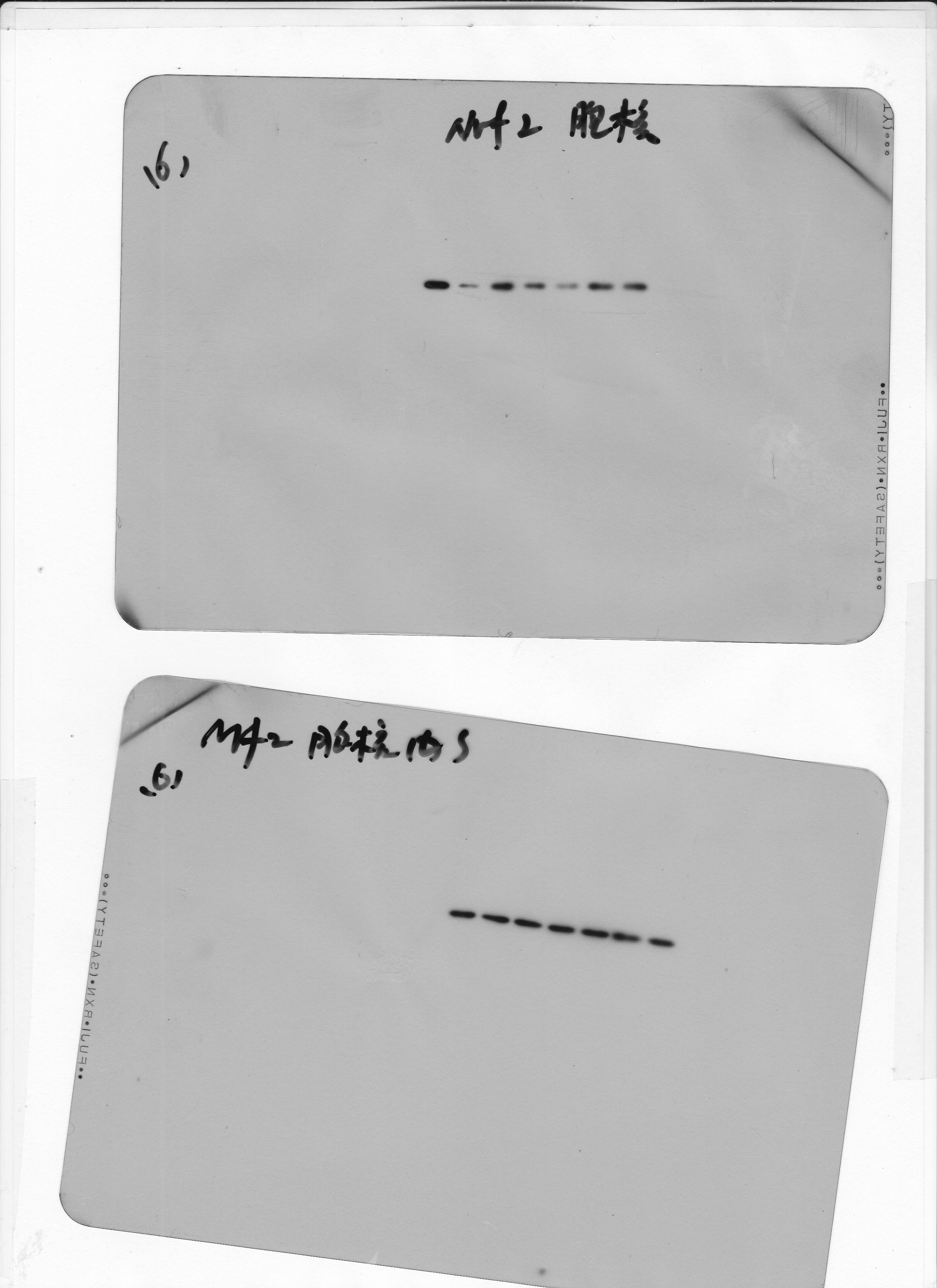

Supplement: Supplementary file 1 [file Data_Sheet_1.ZIP › Western blot figure/nrf2.jpg]

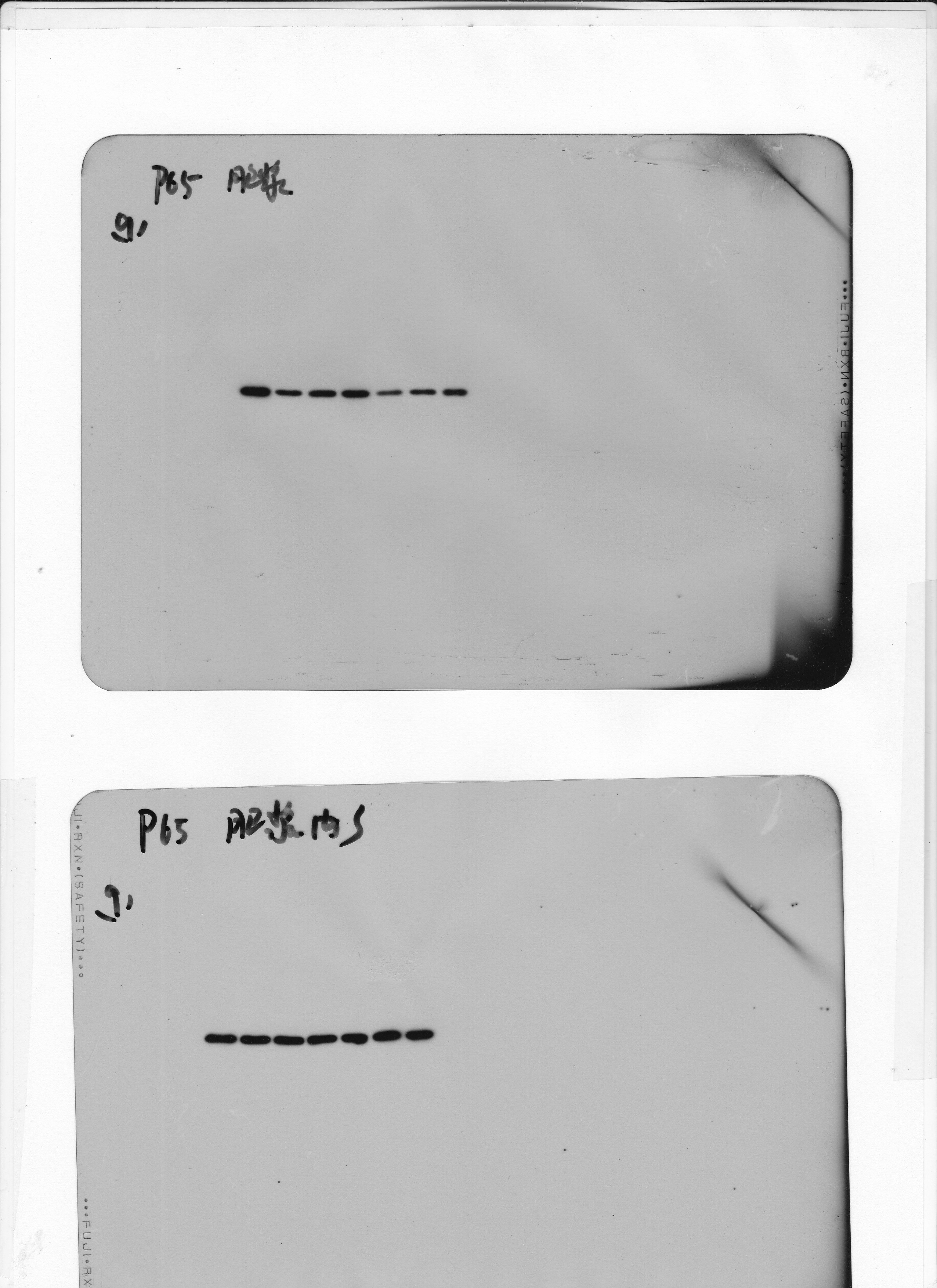

Supplement: Supplementary file 1 [file Data_Sheet_1.ZIP › Western blot figure/p65-2.jpg]

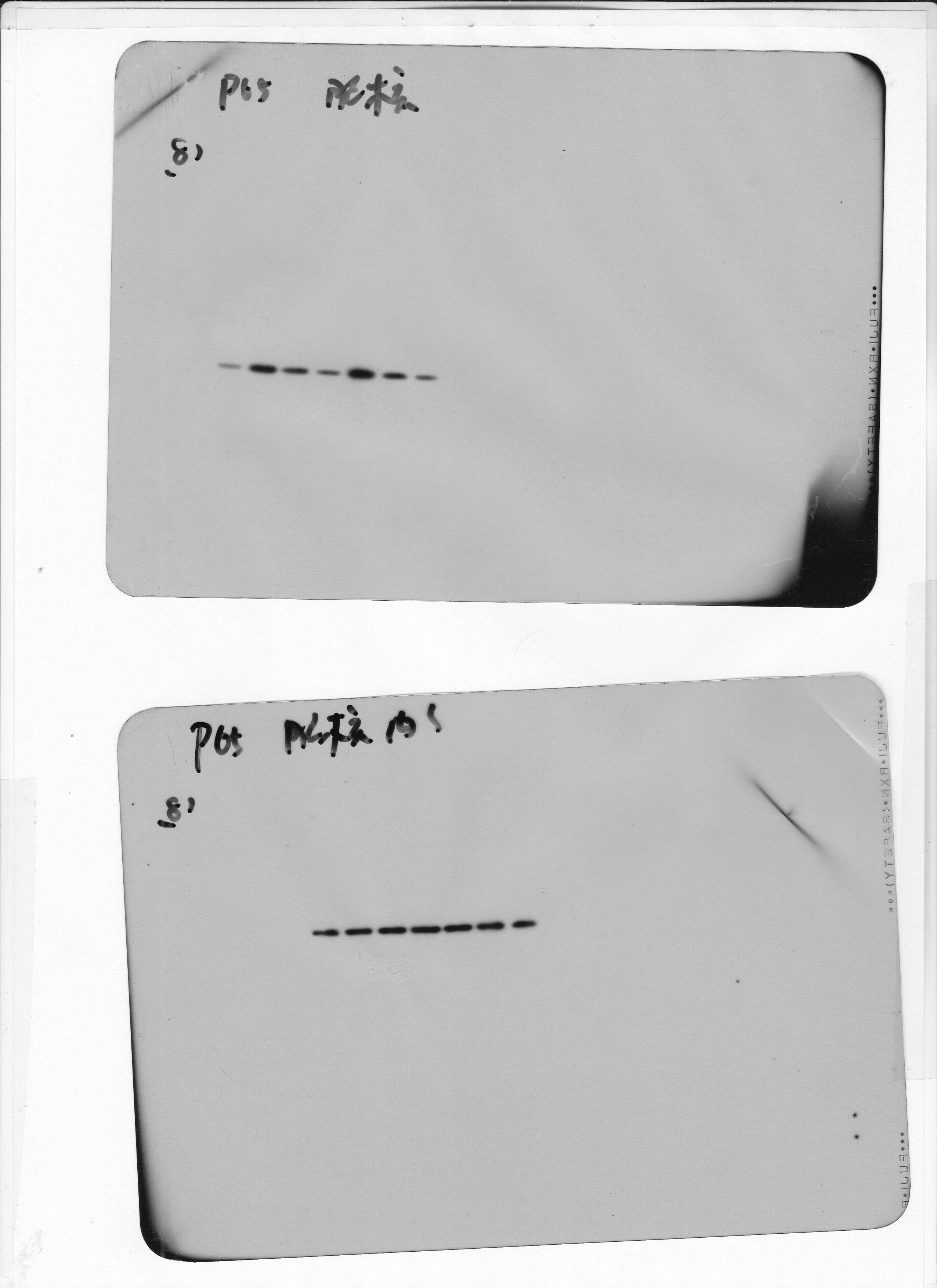

Supplement: Supplementary file 1 [file Data_Sheet_1.ZIP › Western blot figure/p65.jpg]

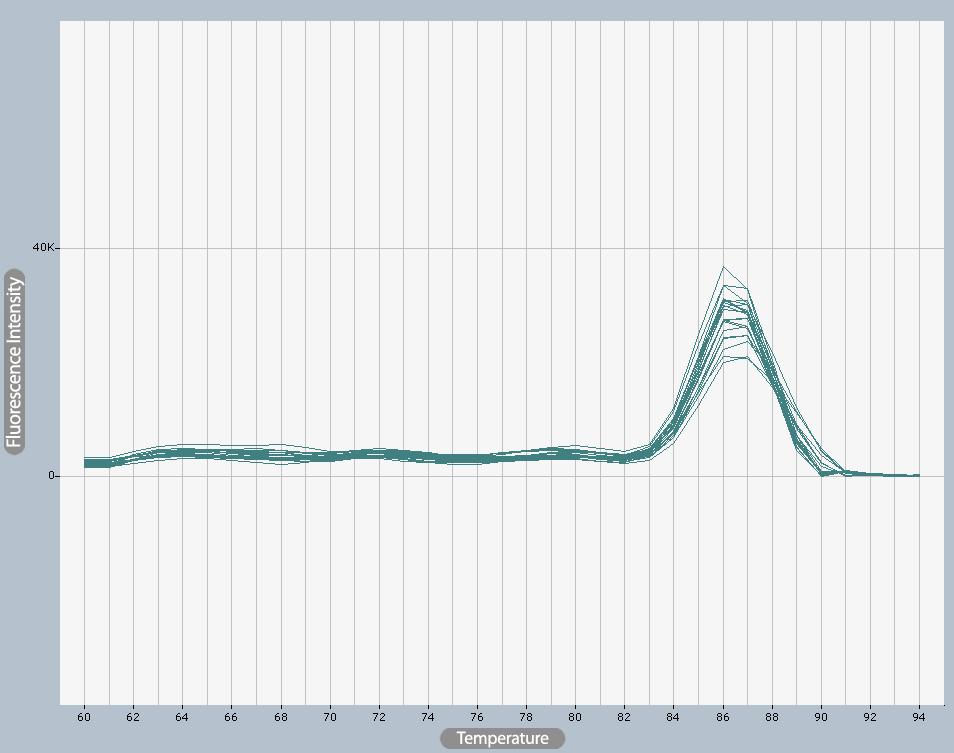

Supplement: Supplementary file 2 [file Data_Sheet_2.ZIP › data/IGF-1R/IGF-1R melting.jpg]

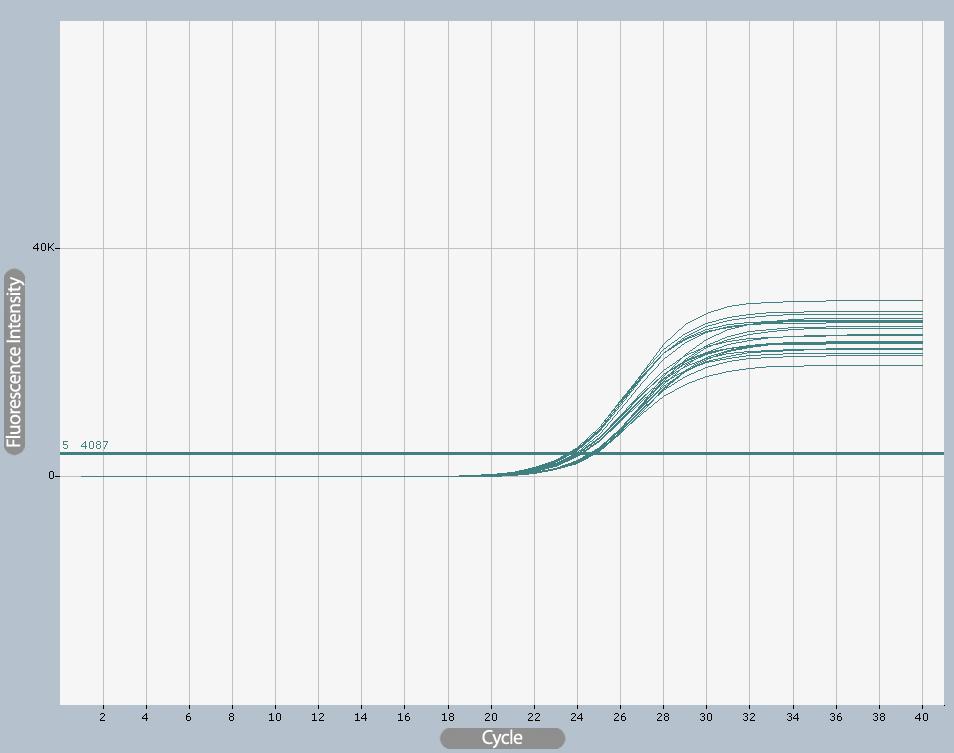

Supplement: Supplementary file 2 [file Data_Sheet_2.ZIP › data/IGF-1R/IGF-1R.jpg]

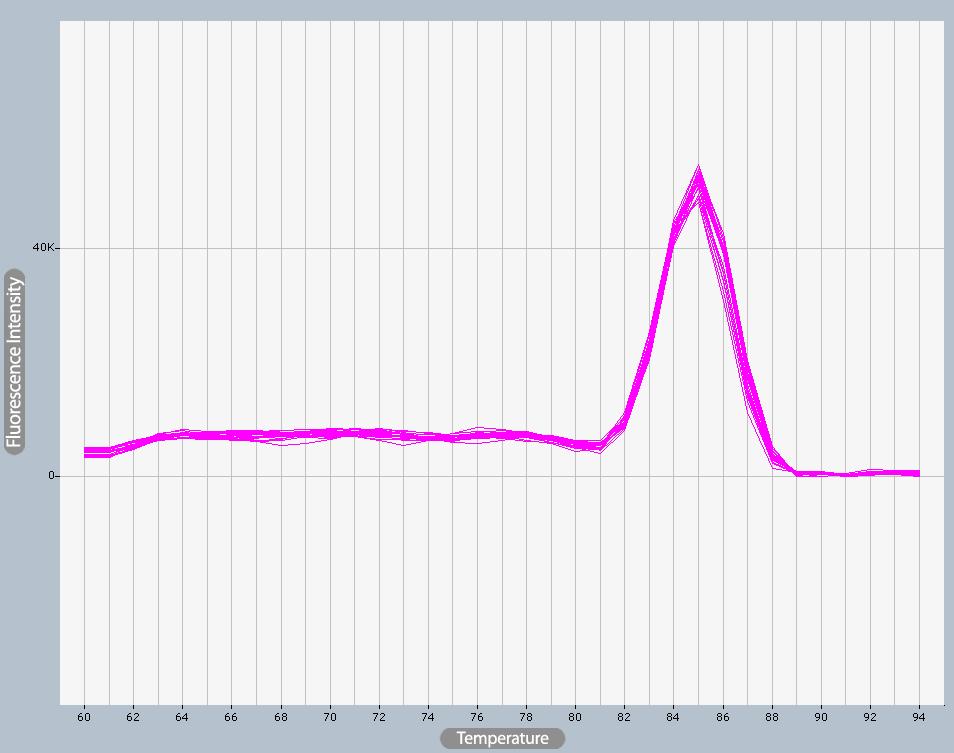

Supplement: Supplementary file 2 [file Data_Sheet_2.ZIP › data/IGF-1R/β-actin melting.jpg]

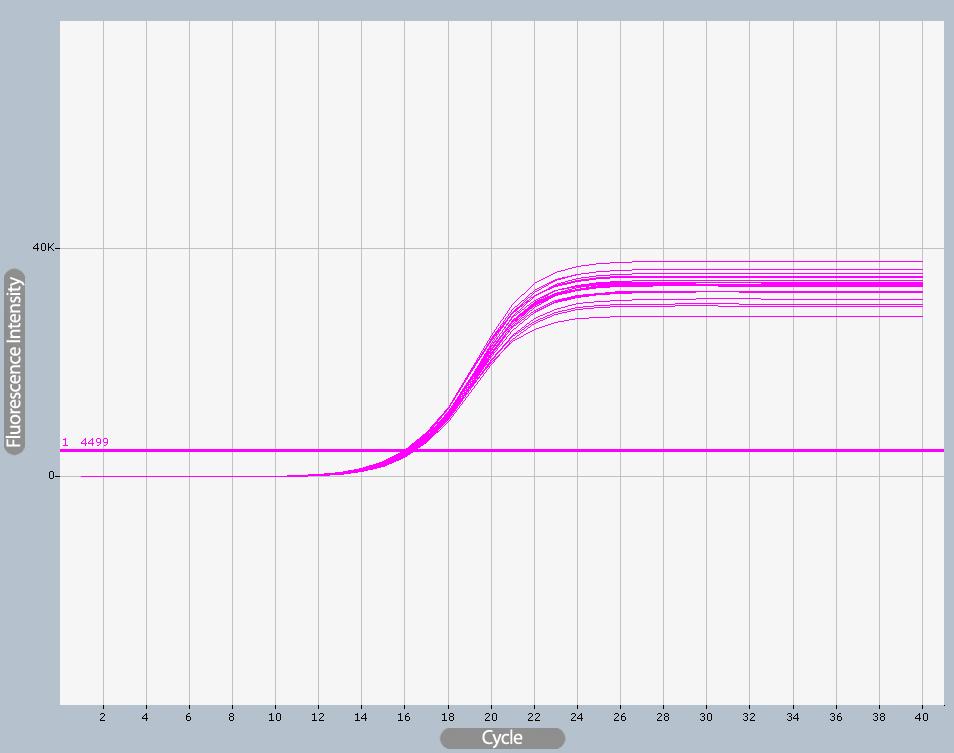

Supplement: Supplementary file 2 [file Data_Sheet_2.ZIP › data/IGF-1R/β-actin.jpg]

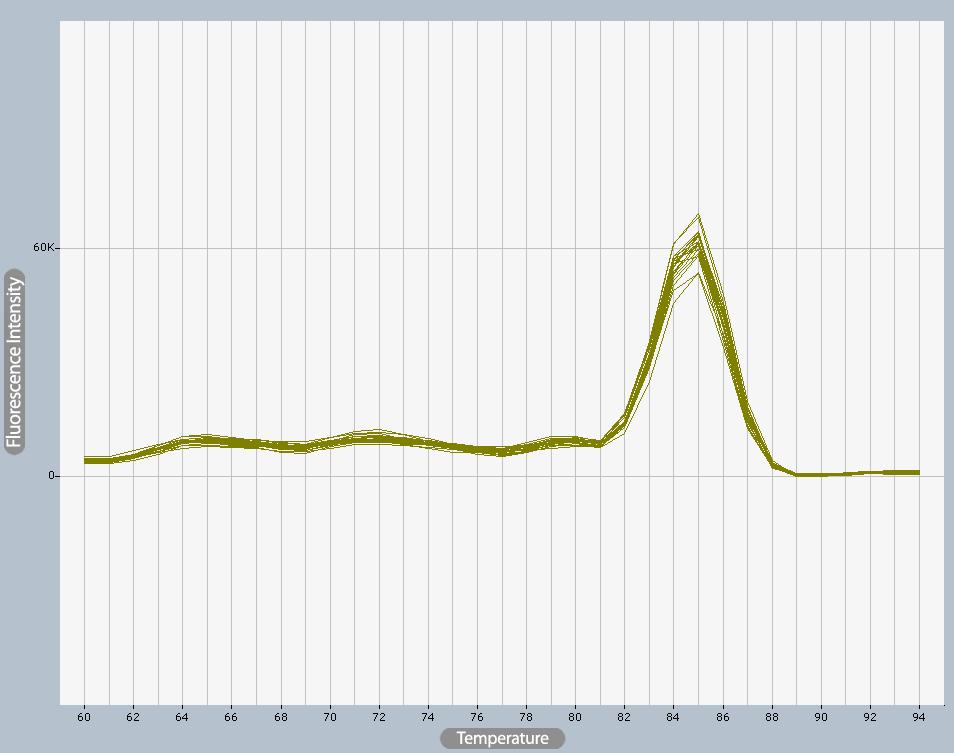

Supplement: Supplementary file 2 [file Data_Sheet_2.ZIP › data/miR-133a-3p/miR-133a-3p melting.jpg]

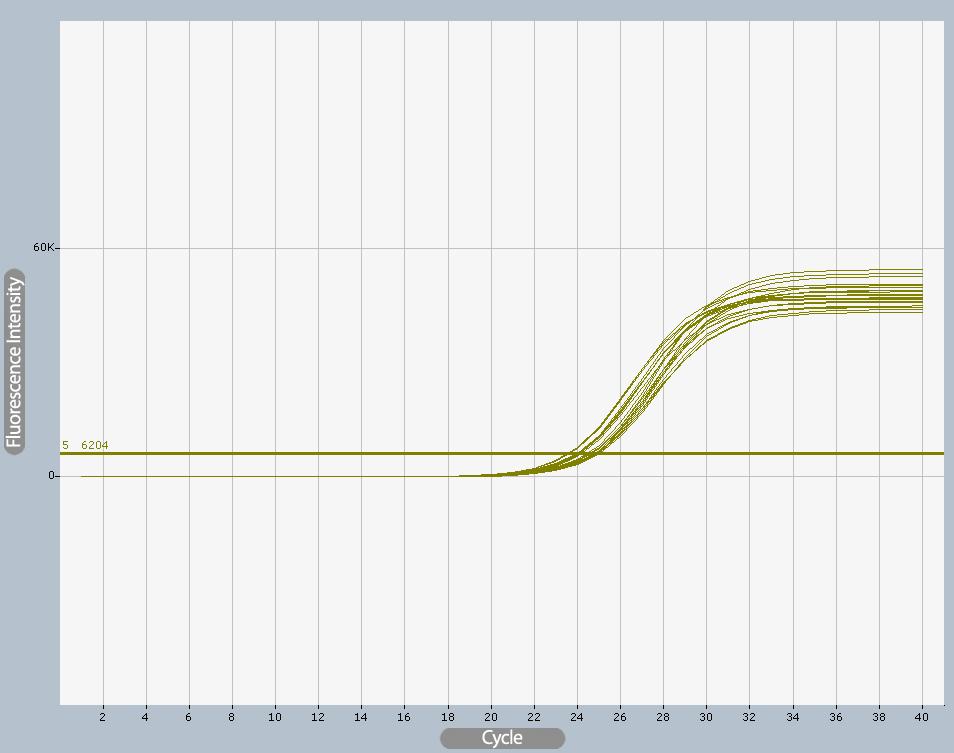

Supplement: Supplementary file 2 [file Data_Sheet_2.ZIP › data/miR-133a-3p/miR-133a-3p.jpg]

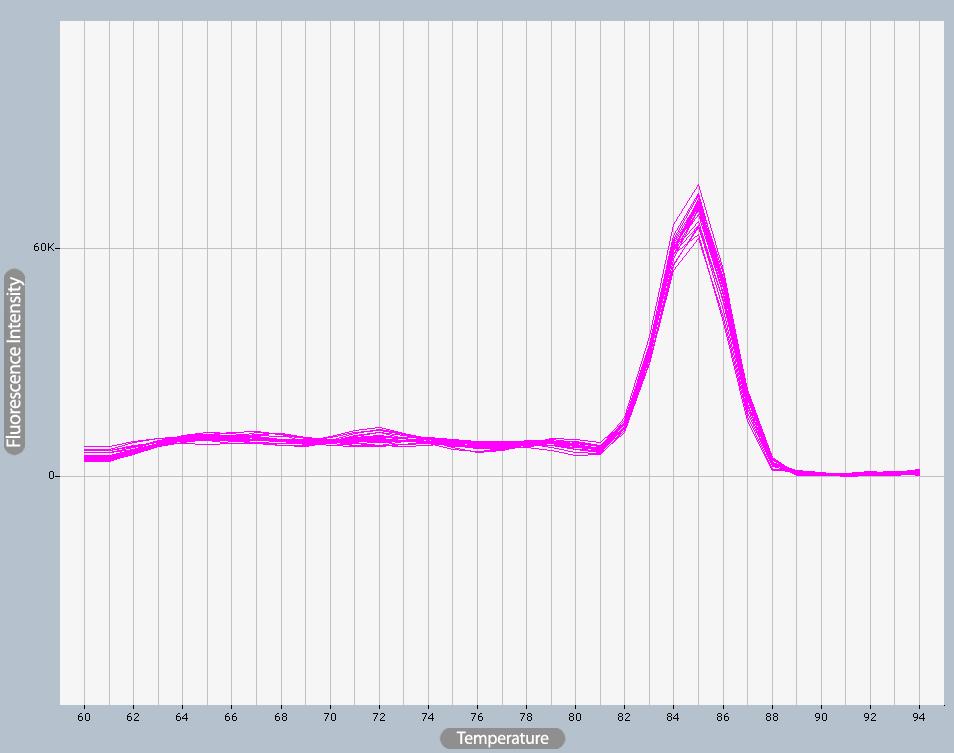

Supplement: Supplementary file 2 [file Data_Sheet_2.ZIP › data/miR-133a-3p/U6 melting.jpg]

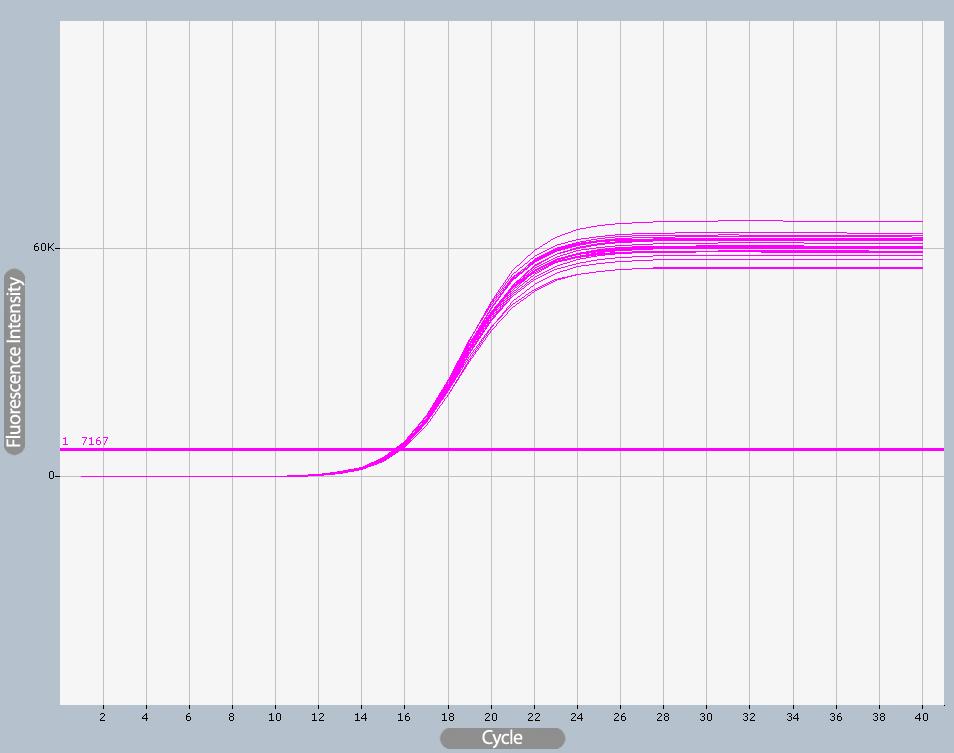

Supplement: Supplementary file 2 [file Data_Sheet_2.ZIP › data/miR-133a-3p/U6.jpg]

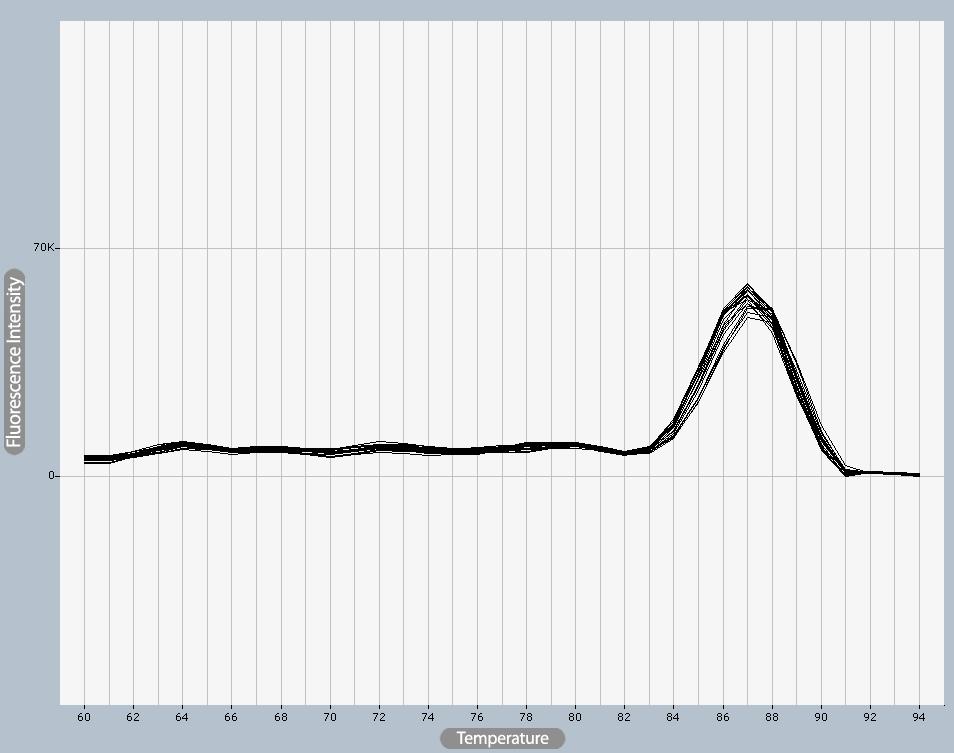

Supplement: Supplementary file 2 [file Data_Sheet_2.ZIP › data/MyoD/MyoD melting.jpg]

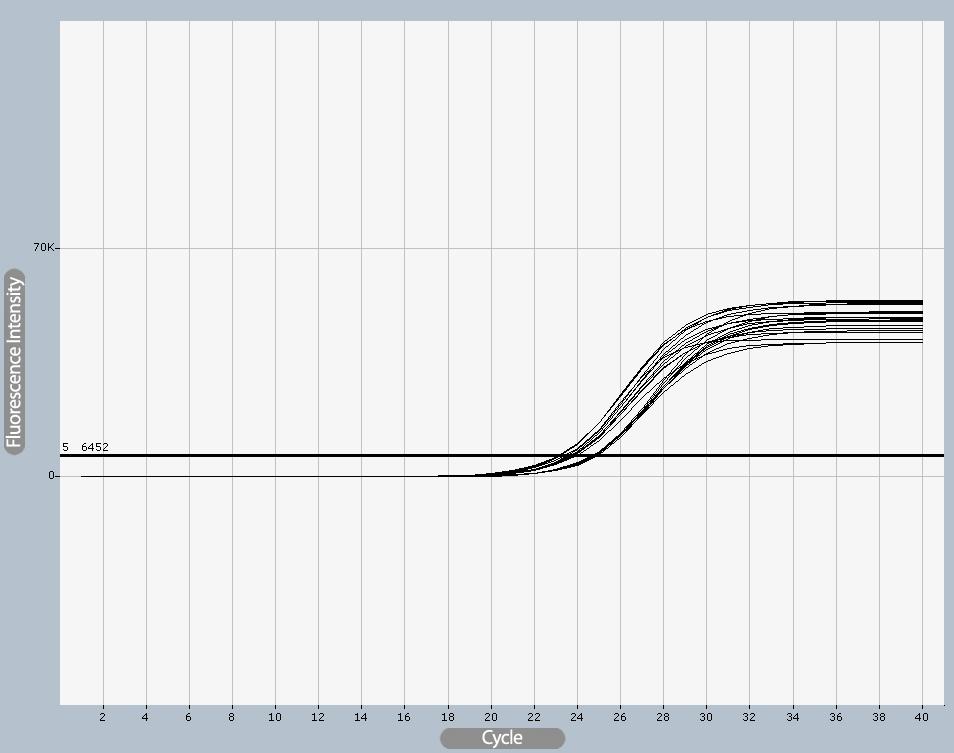

Supplement: Supplementary file 2 [file Data_Sheet_2.ZIP › data/MyoD/MyoD.jpg]

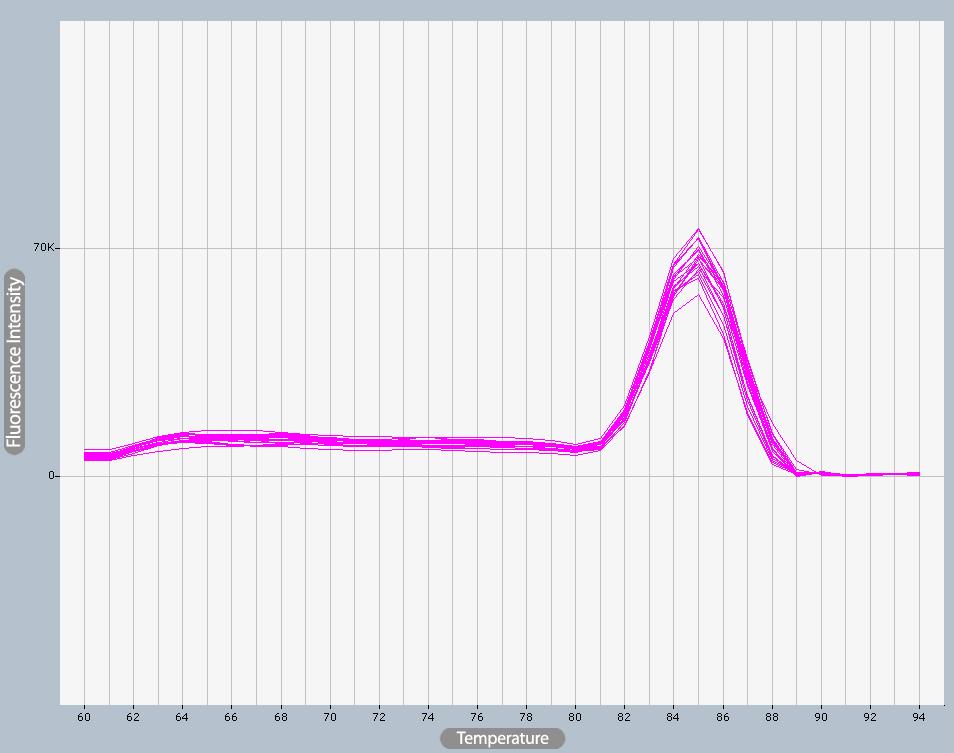

Supplement: Supplementary file 2 [file Data_Sheet_2.ZIP › data/MyoD/β-actin melting.jpg]

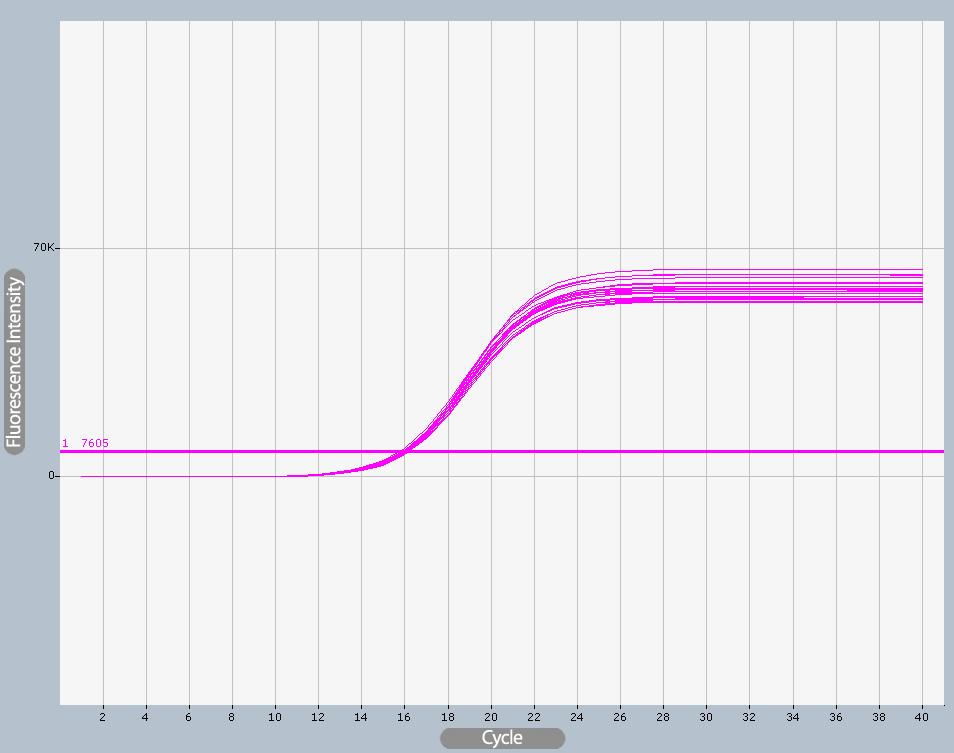

Supplement: Supplementary file 2 [file Data_Sheet_2.ZIP › data/MyoD/β-actin.jpg]

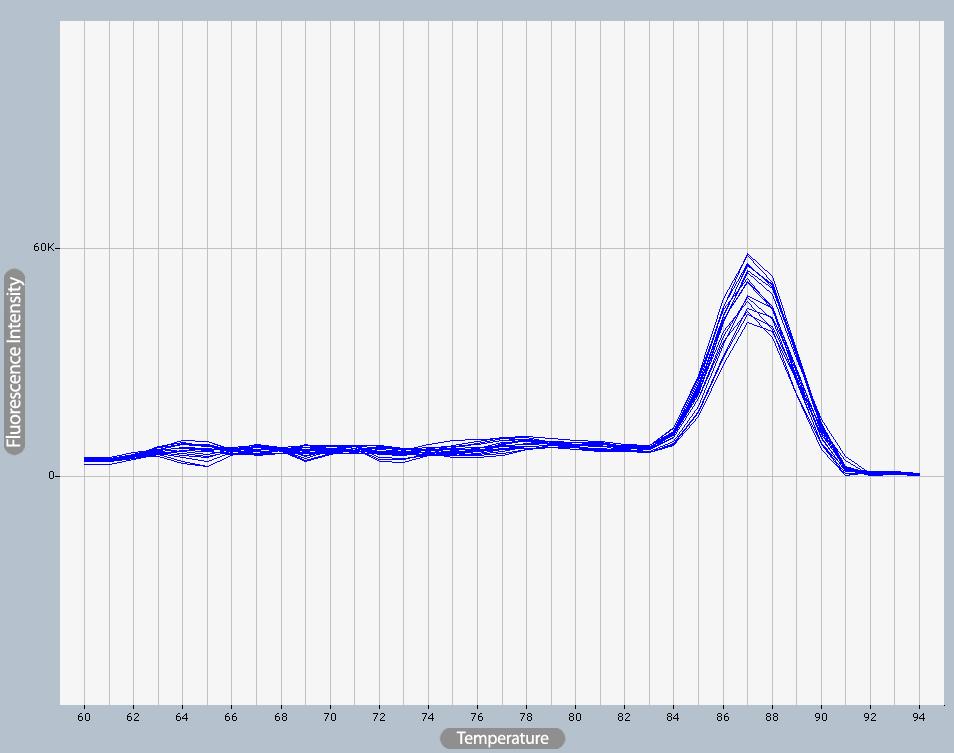

Supplement: Supplementary file 2 [file Data_Sheet_2.ZIP › data/MyoG/MyoG melting.jpg]

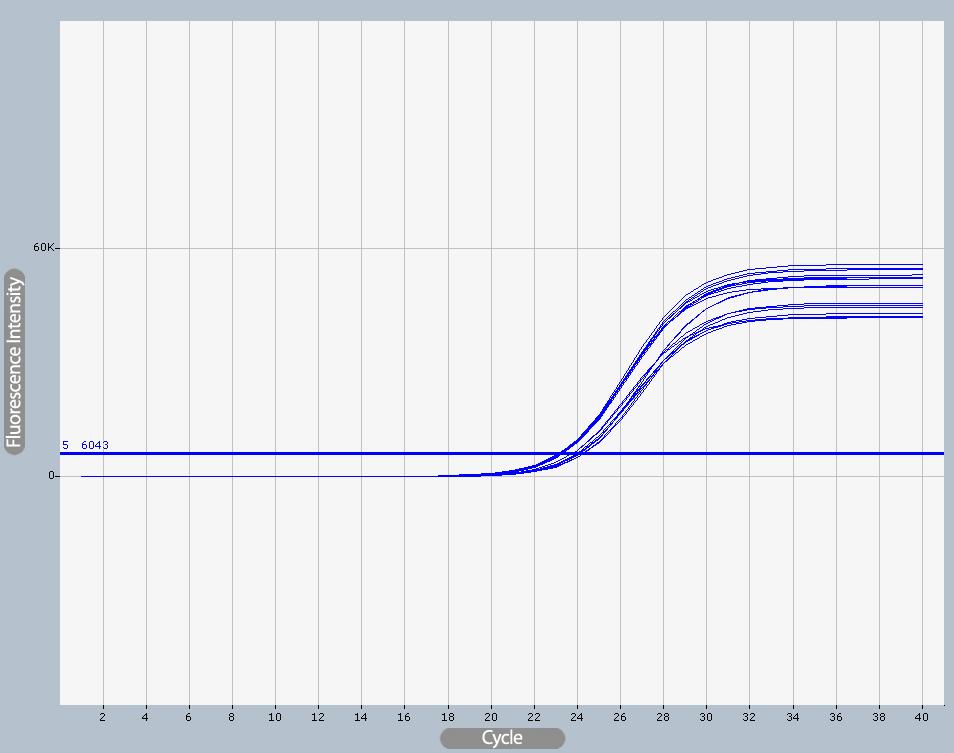

Supplement: Supplementary file 2 [file Data_Sheet_2.ZIP › data/MyoG/MyoG.jpg]

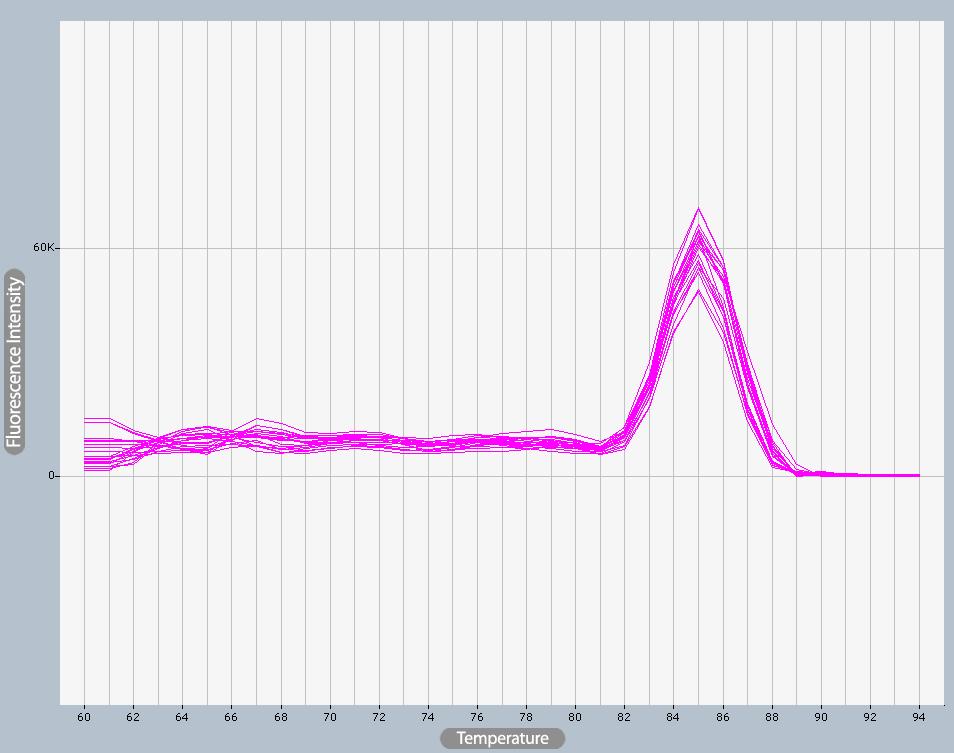

Supplement: Supplementary file 2 [file Data_Sheet_2.ZIP › data/MyoG/β-actin melting.jpg]

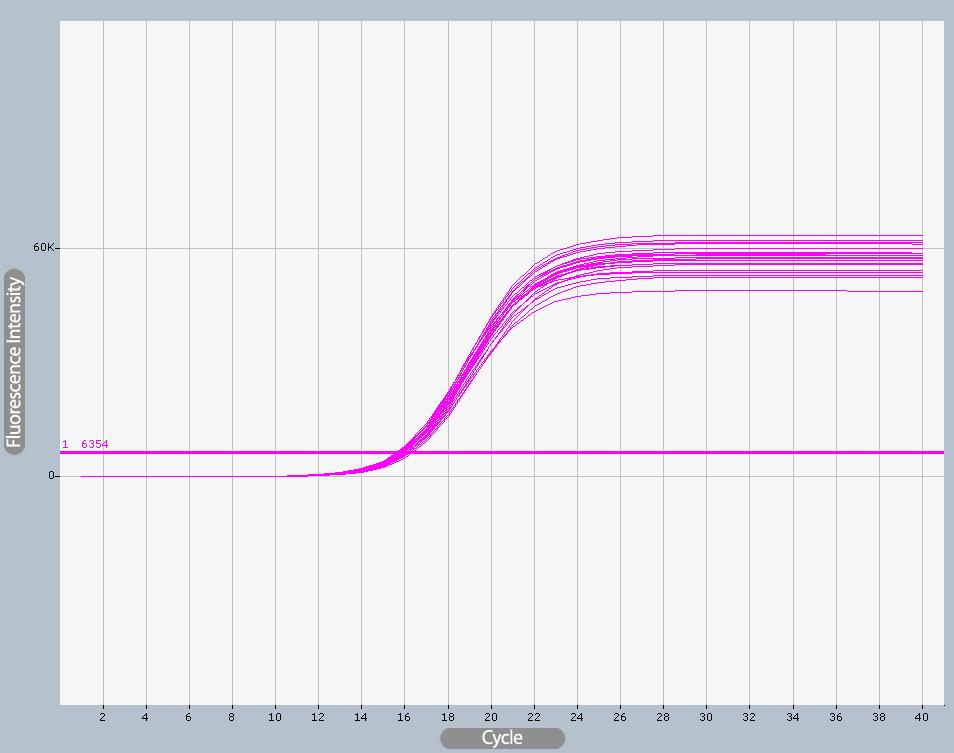

Supplement: Supplementary file 2 [file Data_Sheet_2.ZIP › data/MyoG/β-actin.jpg]

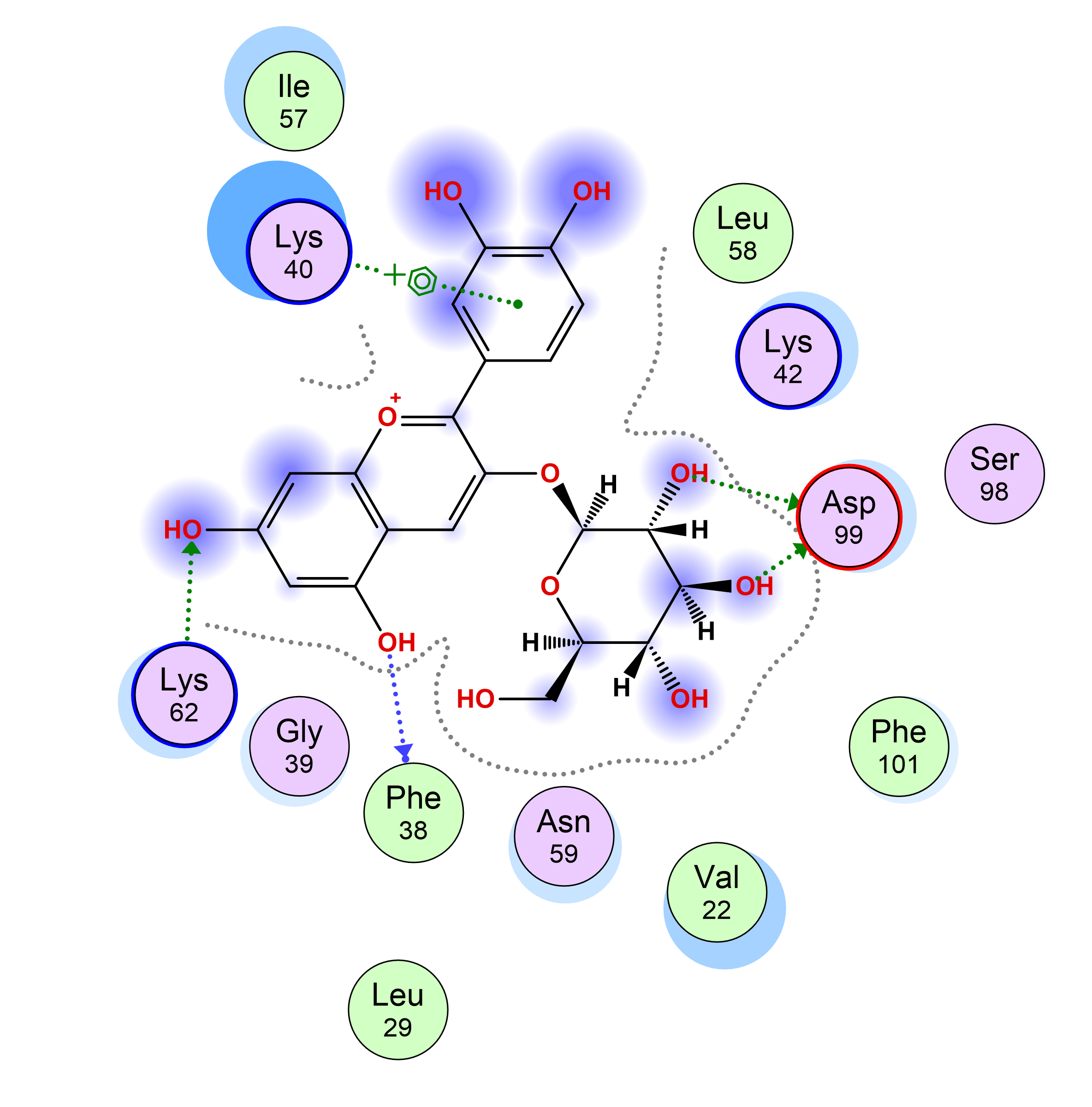

Supplement: Supplementary file 2 [file Data_Sheet_2.ZIP › docking figure/c3og-AF_2D.tif]

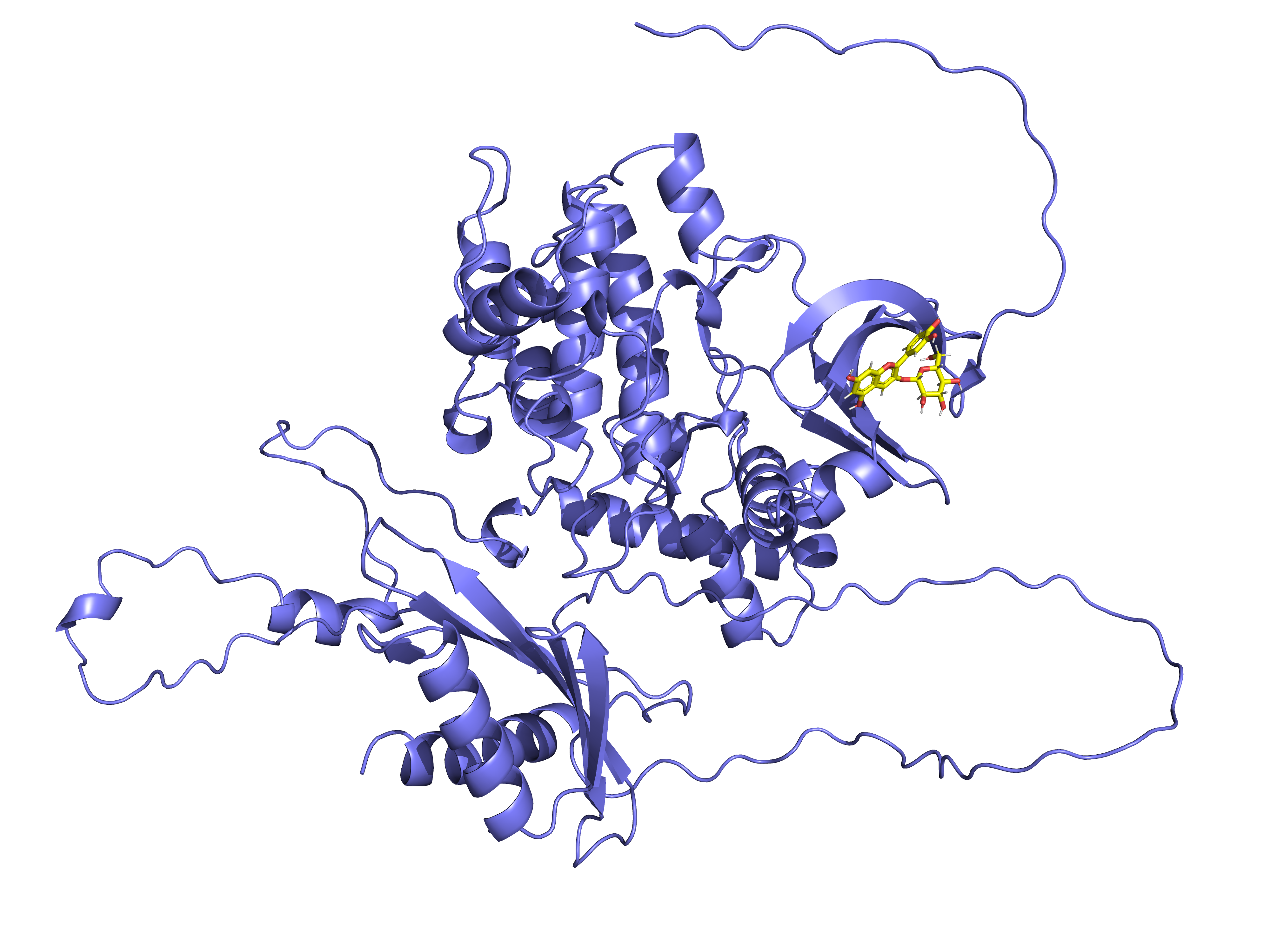

Supplement: Supplementary file 2 [file Data_Sheet_2.ZIP › docking figure/c3og-AF_3D.png]

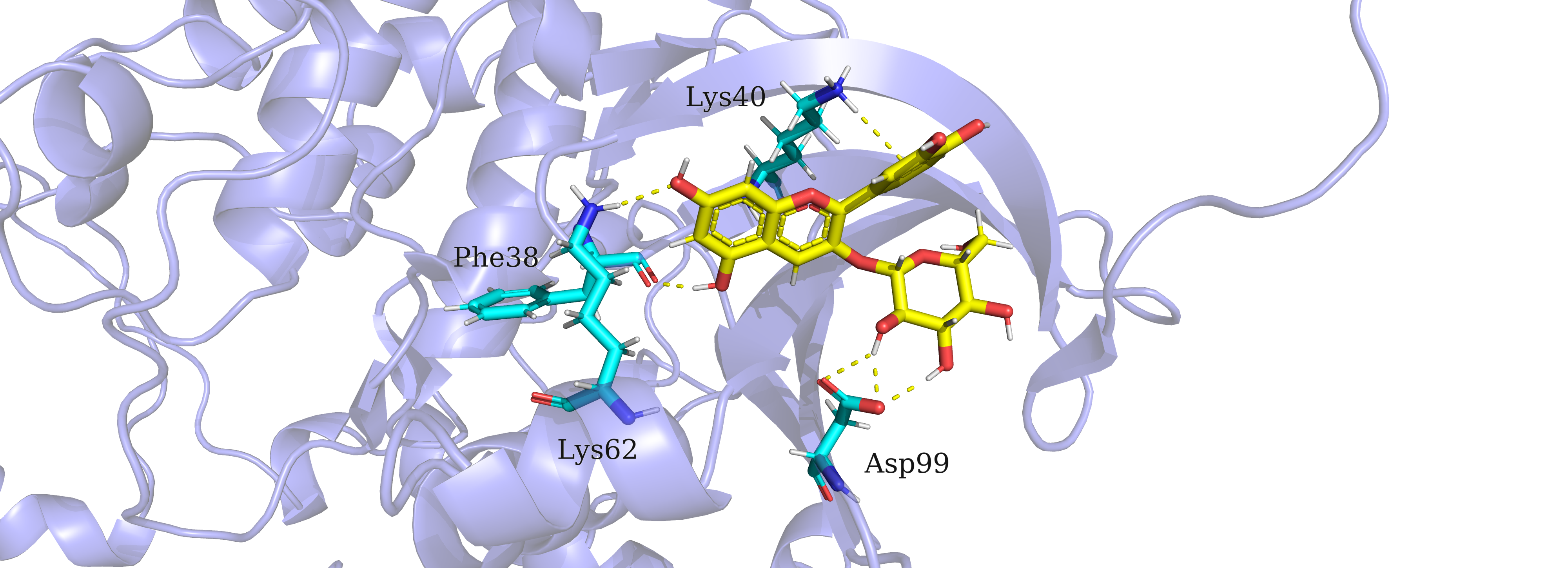

Supplement: Supplementary file 2 [file Data_Sheet_2.ZIP › docking figure/c3og-AF_detail.png]

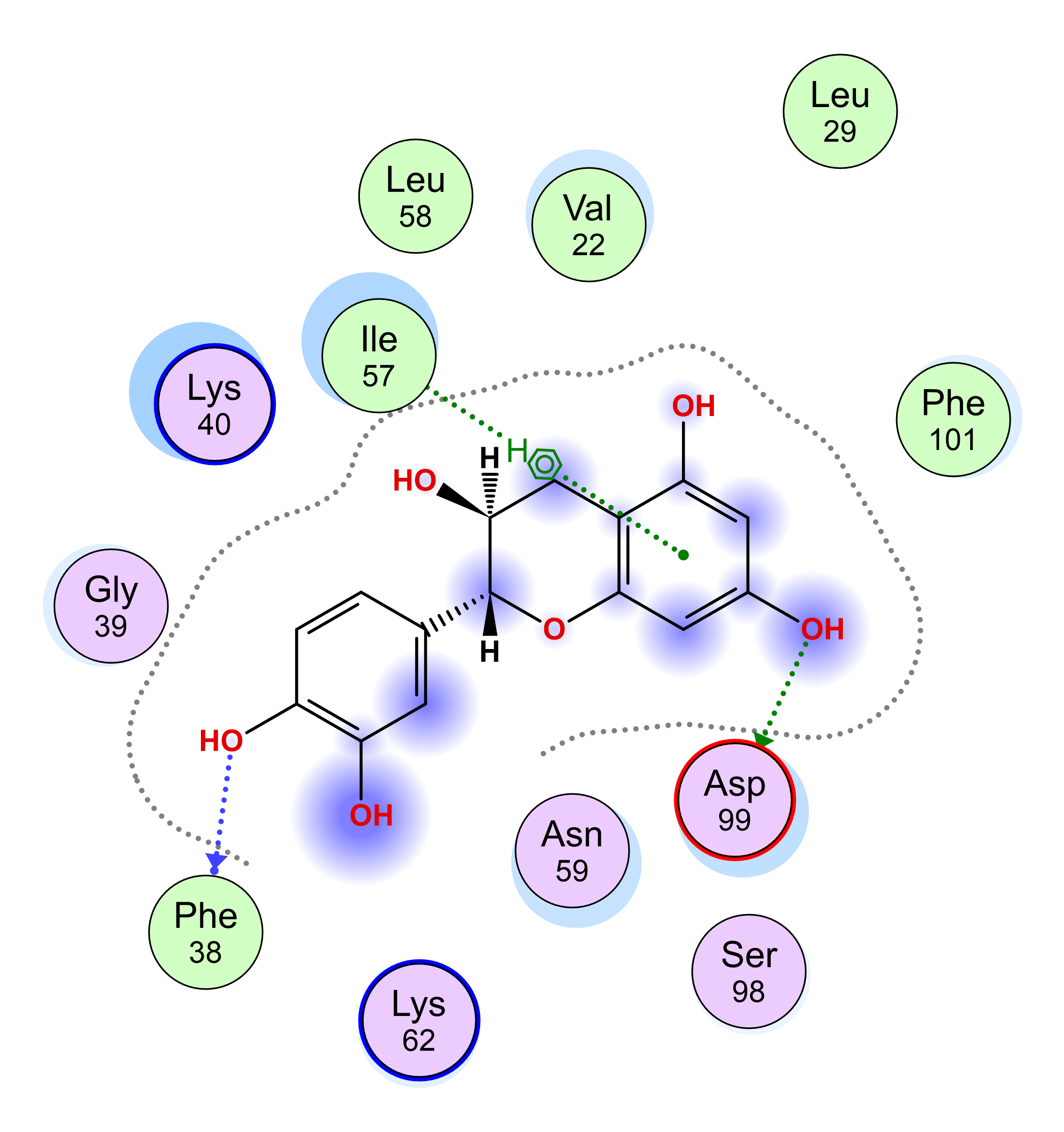

Supplement: Supplementary file 2 [file Data_Sheet_2.ZIP › docking figure/catechin-AF_2D.tif]

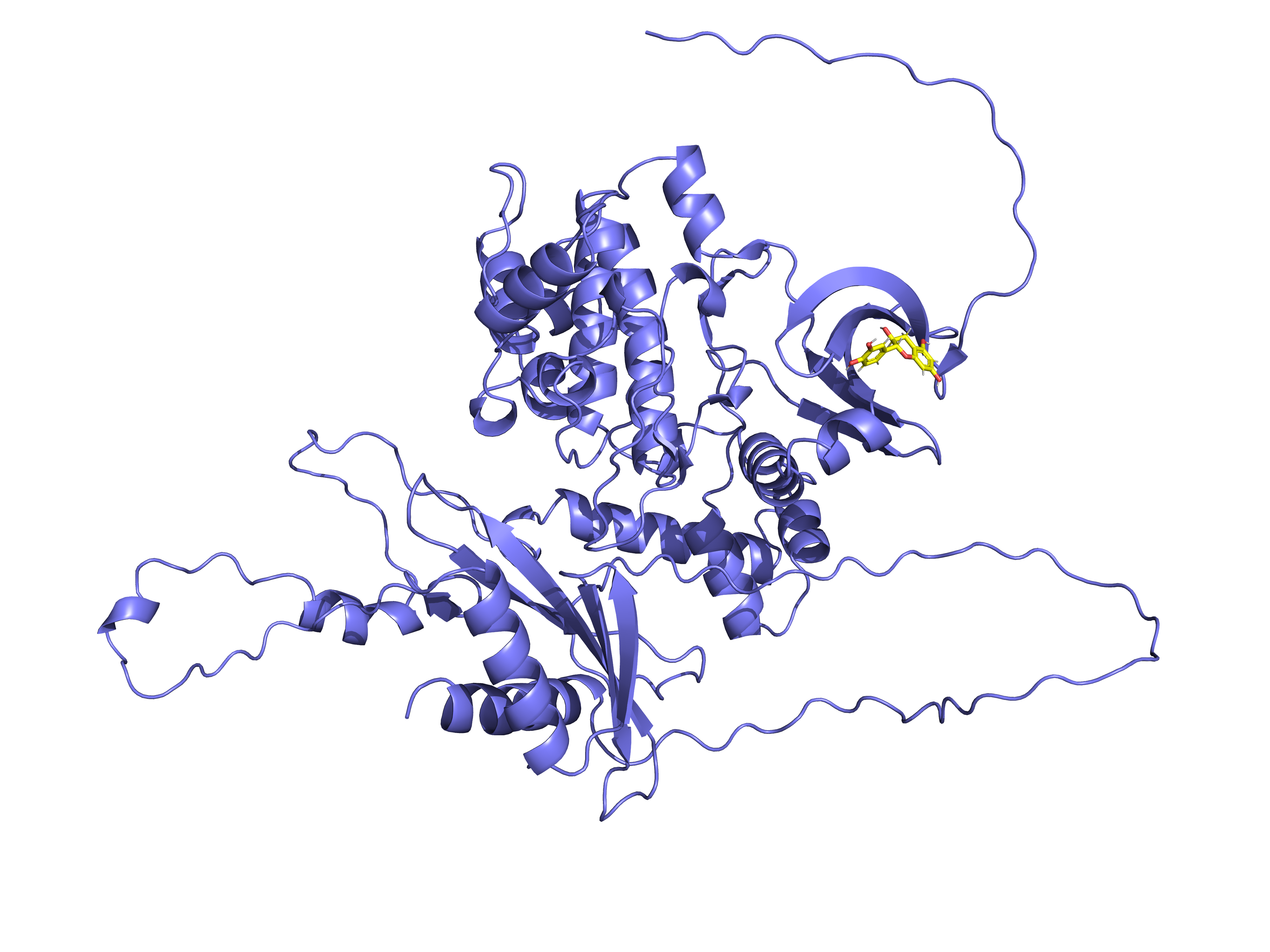

Supplement: Supplementary file 2 [file Data_Sheet_2.ZIP › docking figure/catechin-AF_3D.png]

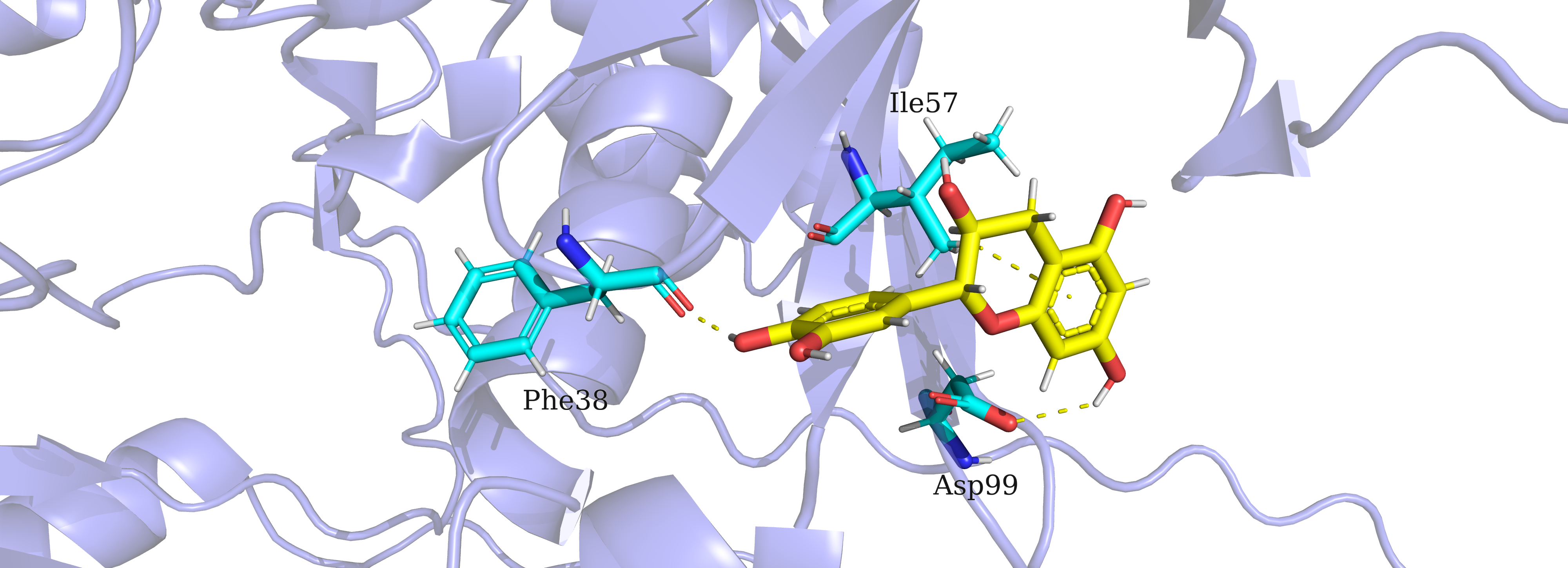

Supplement: Supplementary file 2 [file Data_Sheet_2.ZIP › docking figure/catechin-AF_detail.png]

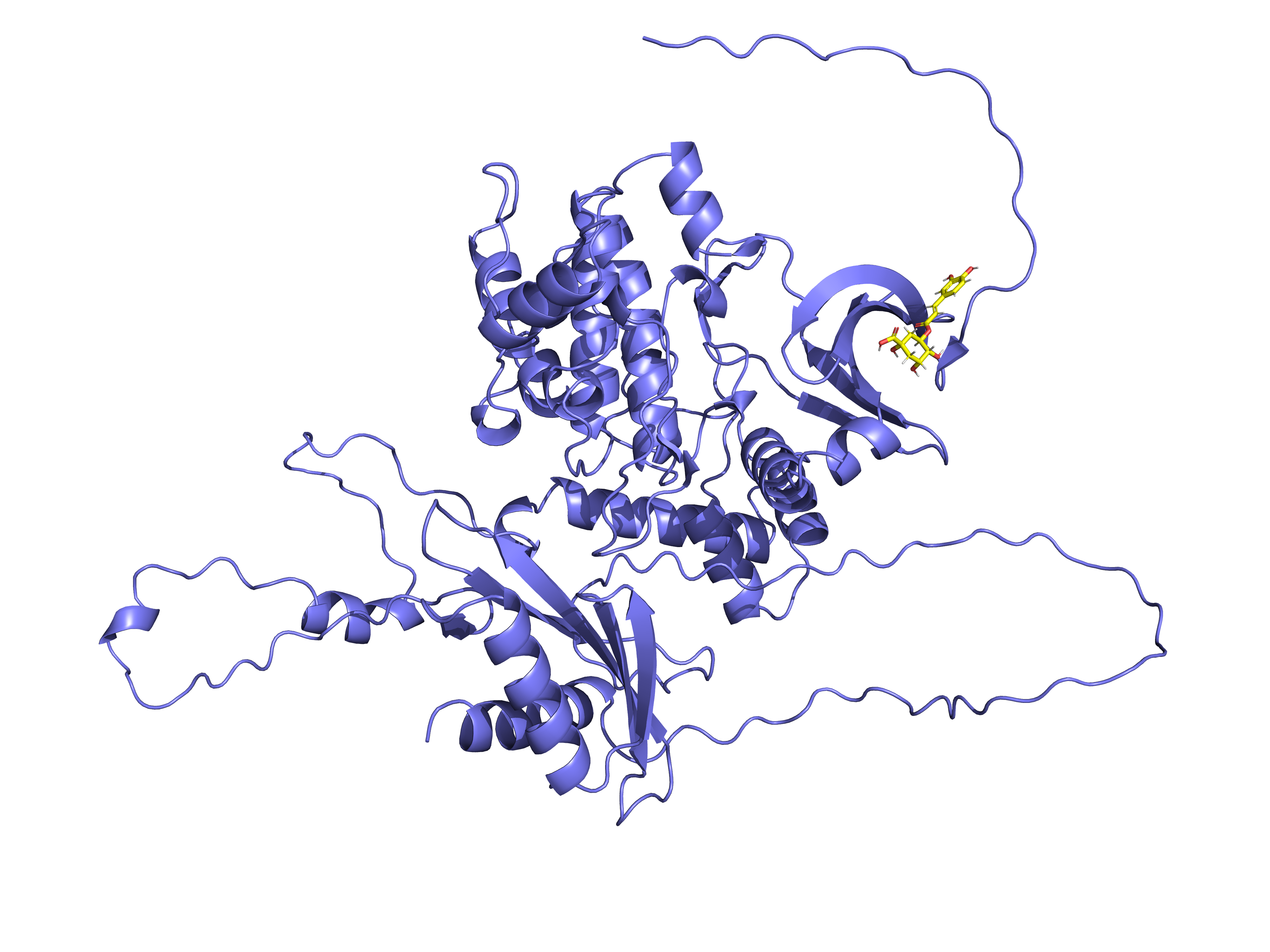

Supplement: Supplementary file 2 [file Data_Sheet_2.ZIP › docking figure/Chlorogenic_acid-AF-3D.png]

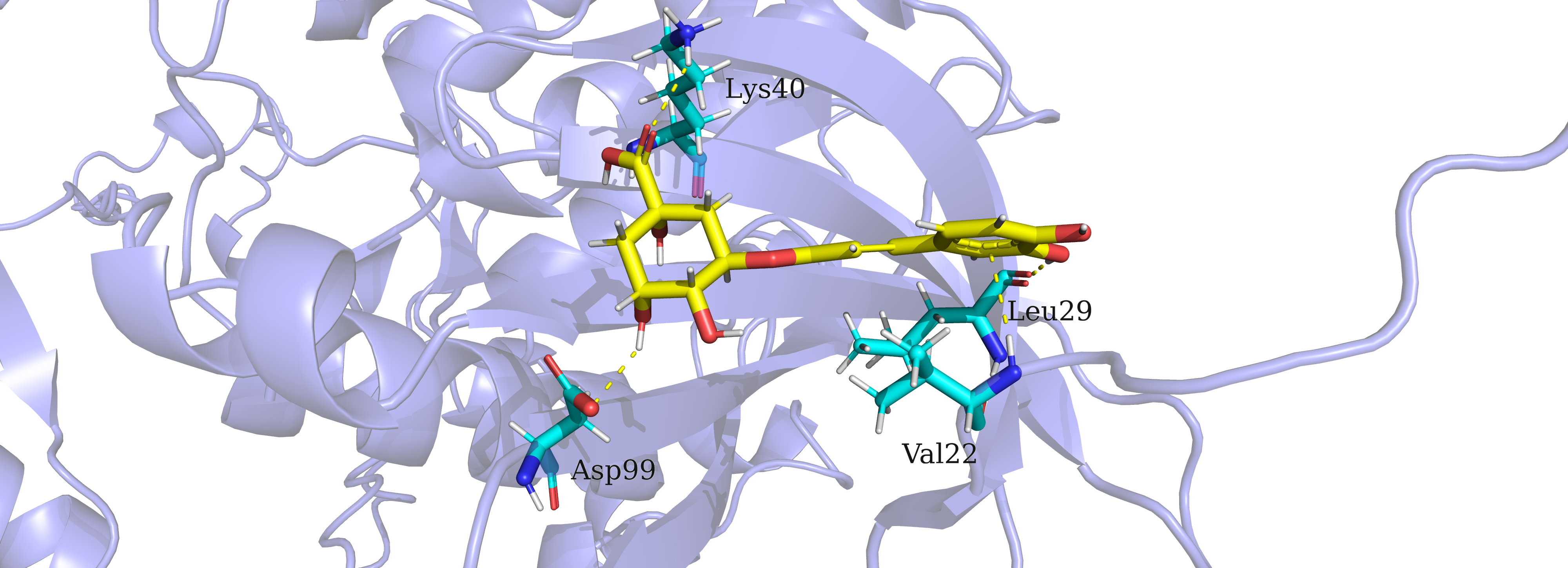

Supplement: Supplementary file 2 [file Data_Sheet_2.ZIP › docking figure/Chlorogenic_acid-AF-detail.png]

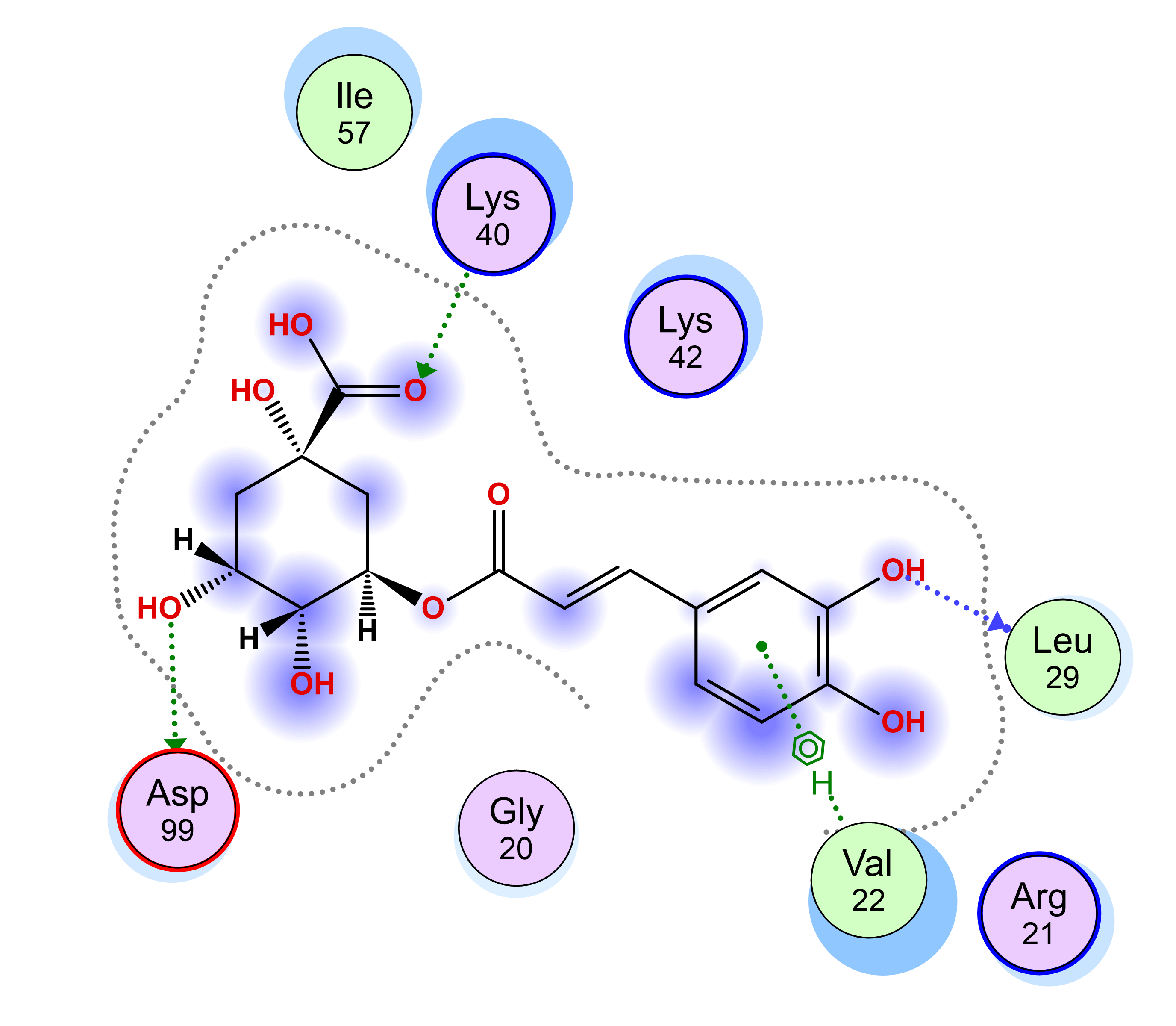

Supplement: Supplementary file 2 [file Data_Sheet_2.ZIP › docking figure/Chlorogenic_acid-AF_2D.tif]

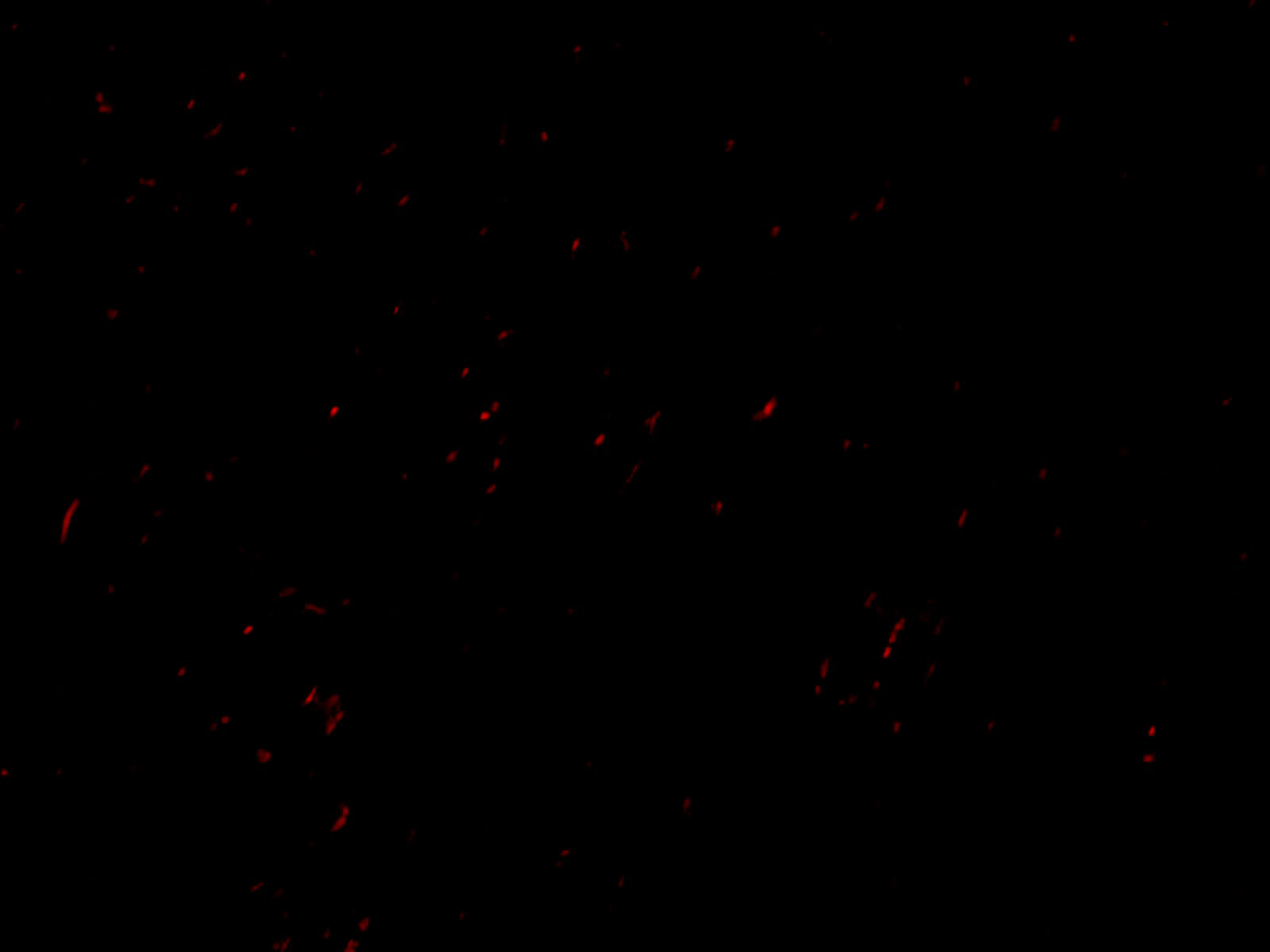

Supplement: Supplementary file 3 [file Data_Sheet_3.ZIP › microscopy images/DHE/C.jpg]

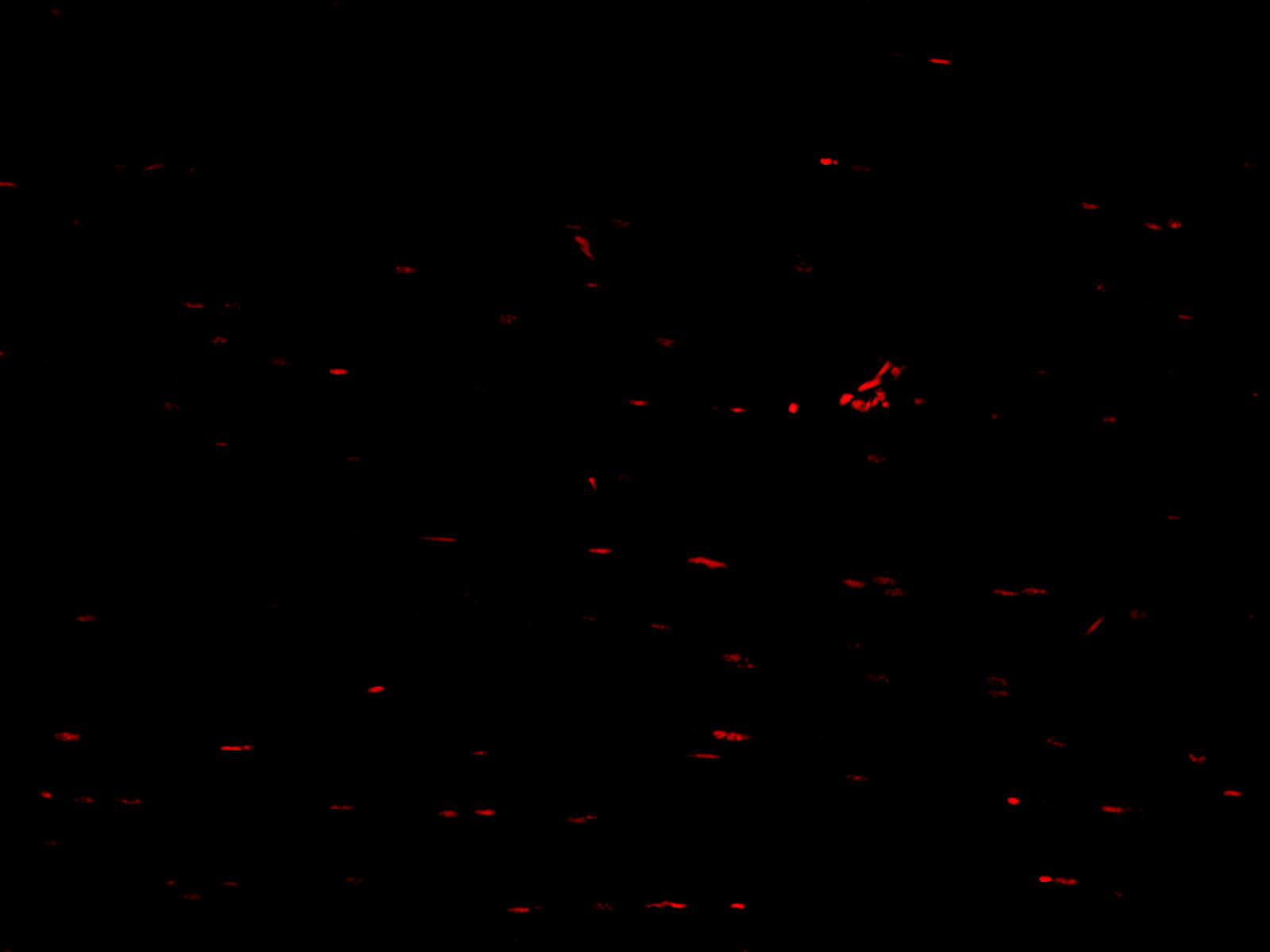

Supplement: Supplementary file 3 [file Data_Sheet_3.ZIP › microscopy images/DHE/L-LCBP.jpg]

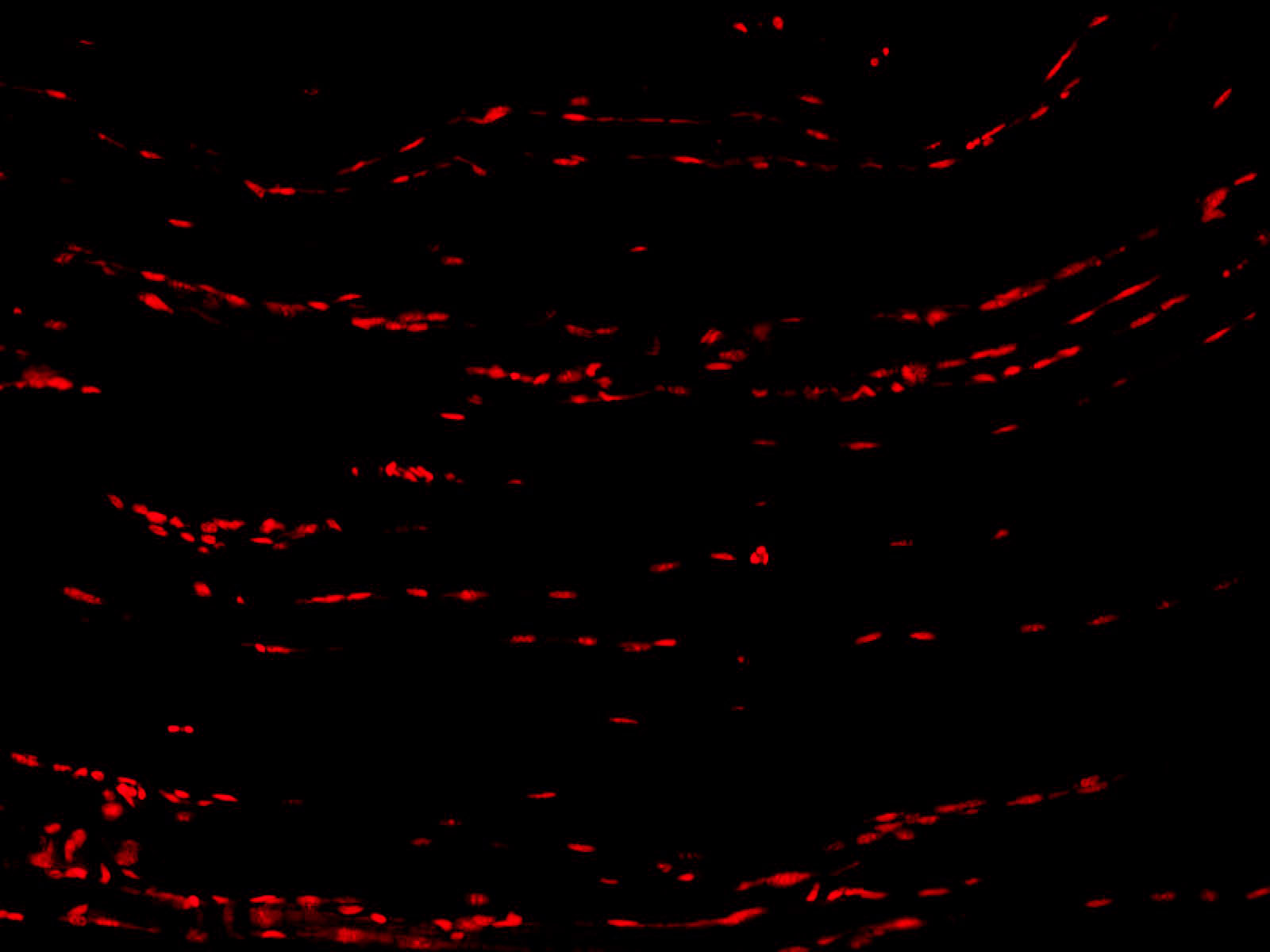

Supplement: Supplementary file 3 [file Data_Sheet_3.ZIP › microscopy images/DHE/L-M.jpg]

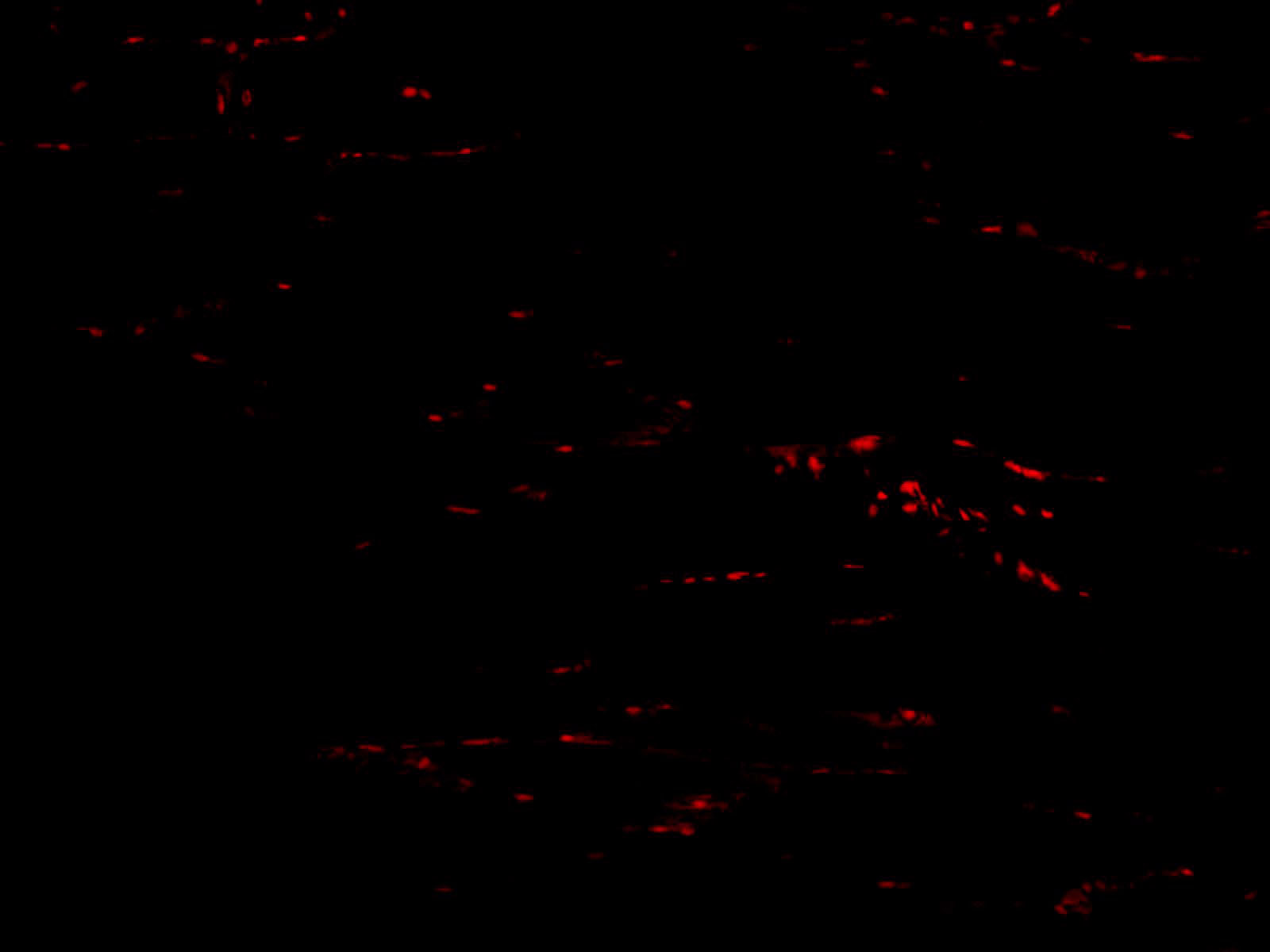

Supplement: Supplementary file 3 [file Data_Sheet_3.ZIP › microscopy images/DHE/L-VC.jpg]

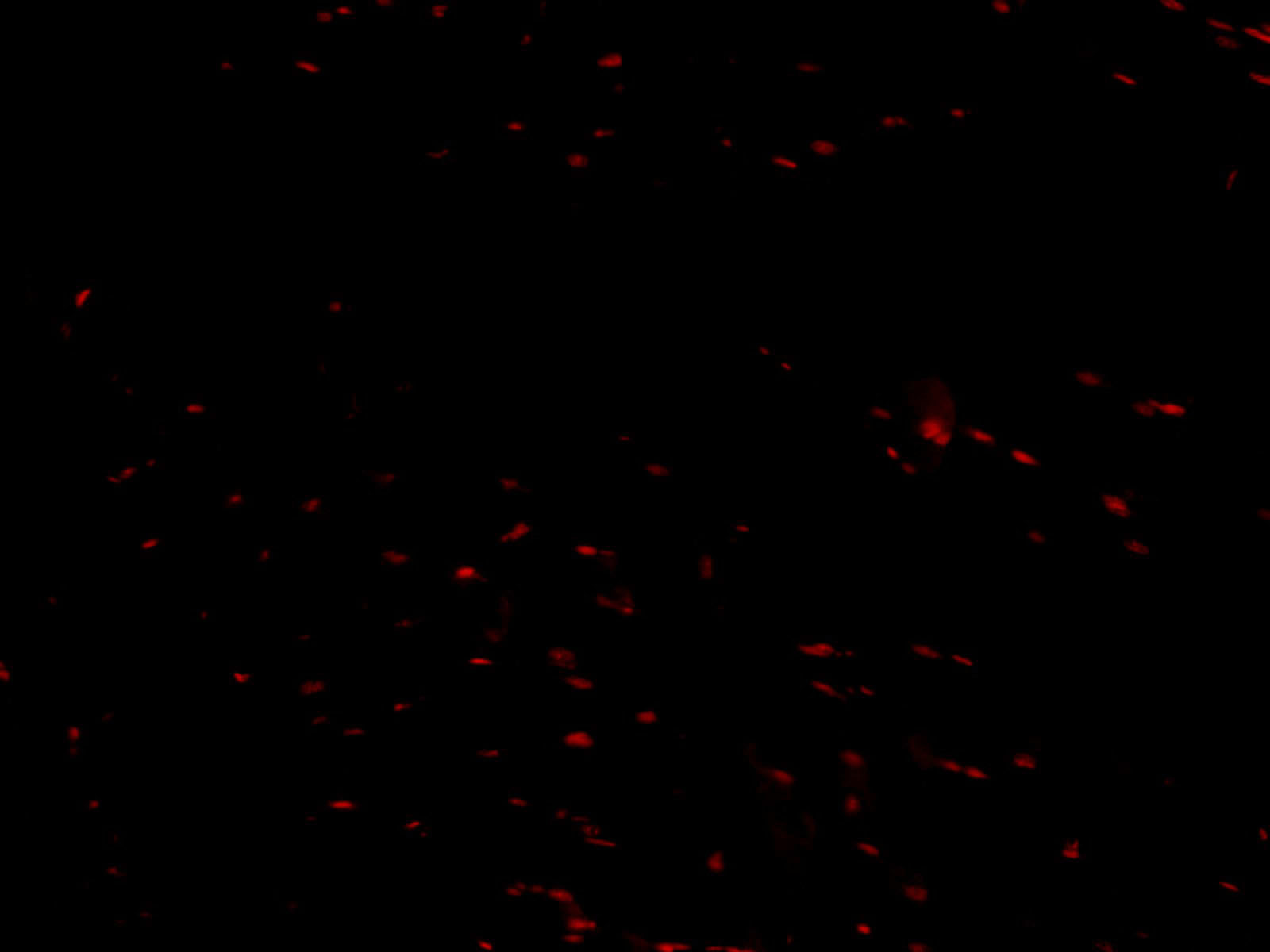

Supplement: Supplementary file 3 [file Data_Sheet_3.ZIP › microscopy images/DHE/LCBP.jpg]

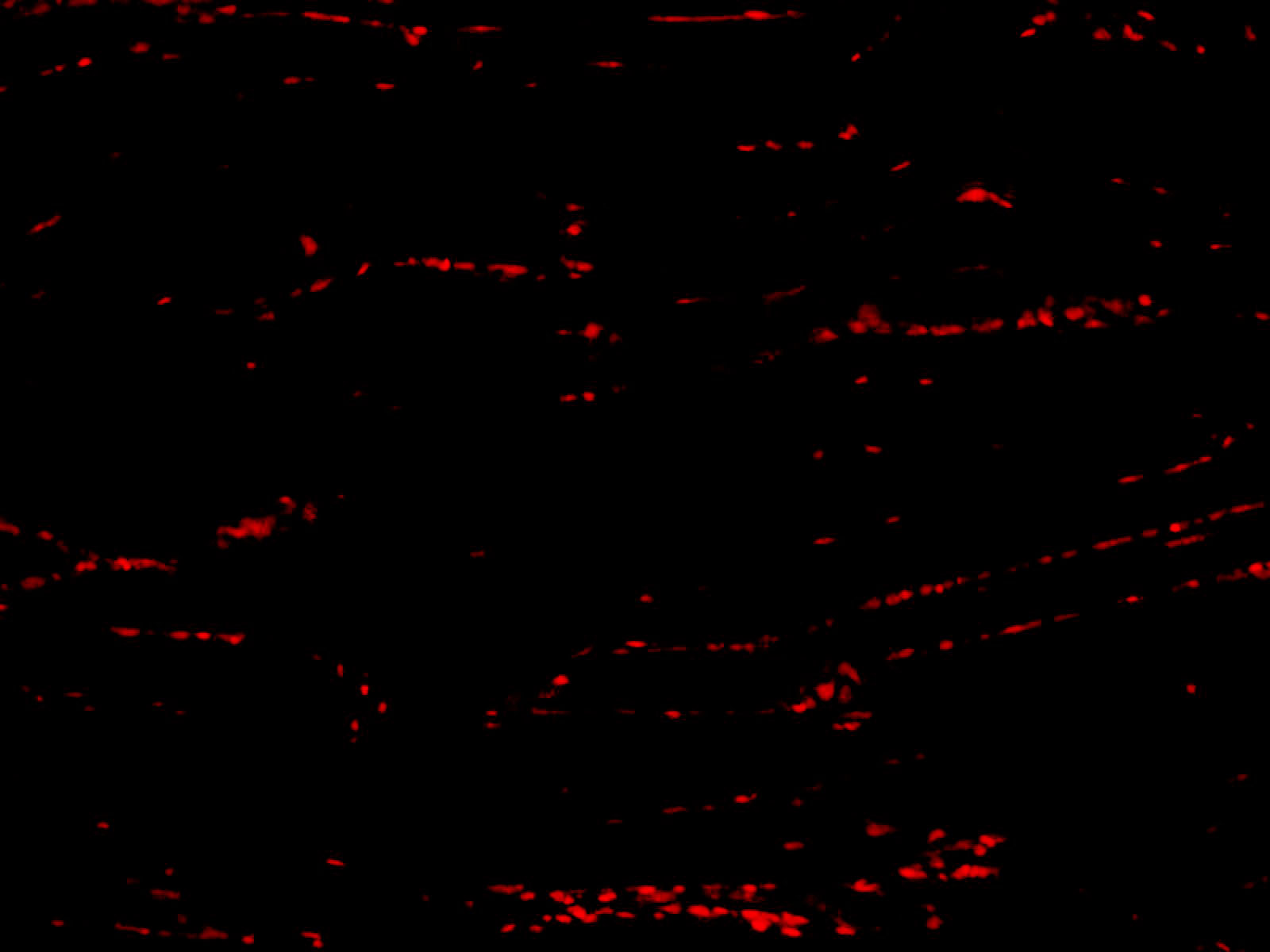

Supplement: Supplementary file 3 [file Data_Sheet_3.ZIP › microscopy images/DHE/M.jpg]

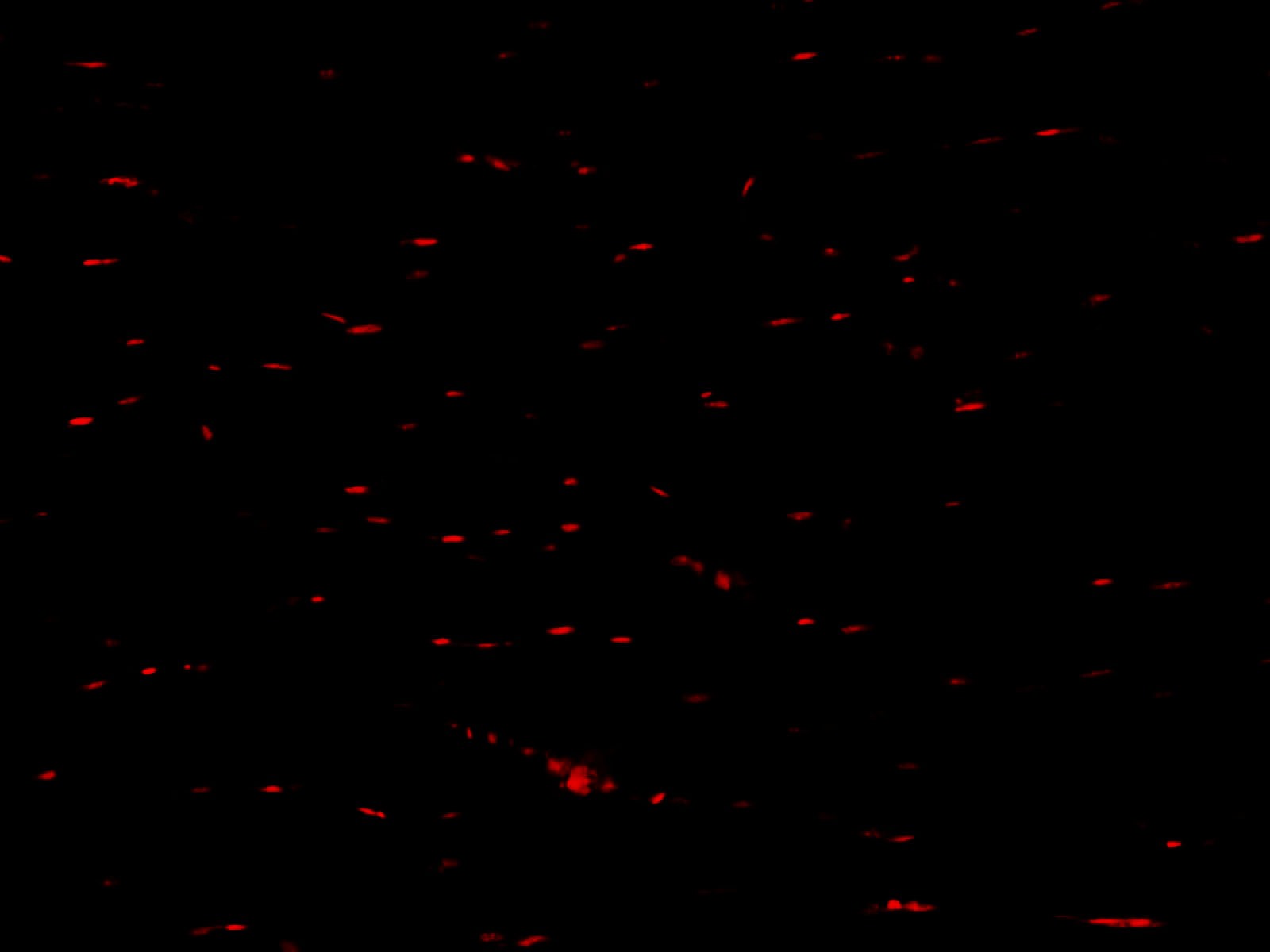

Supplement: Supplementary file 3 [file Data_Sheet_3.ZIP › microscopy images/DHE/VC.jpg]

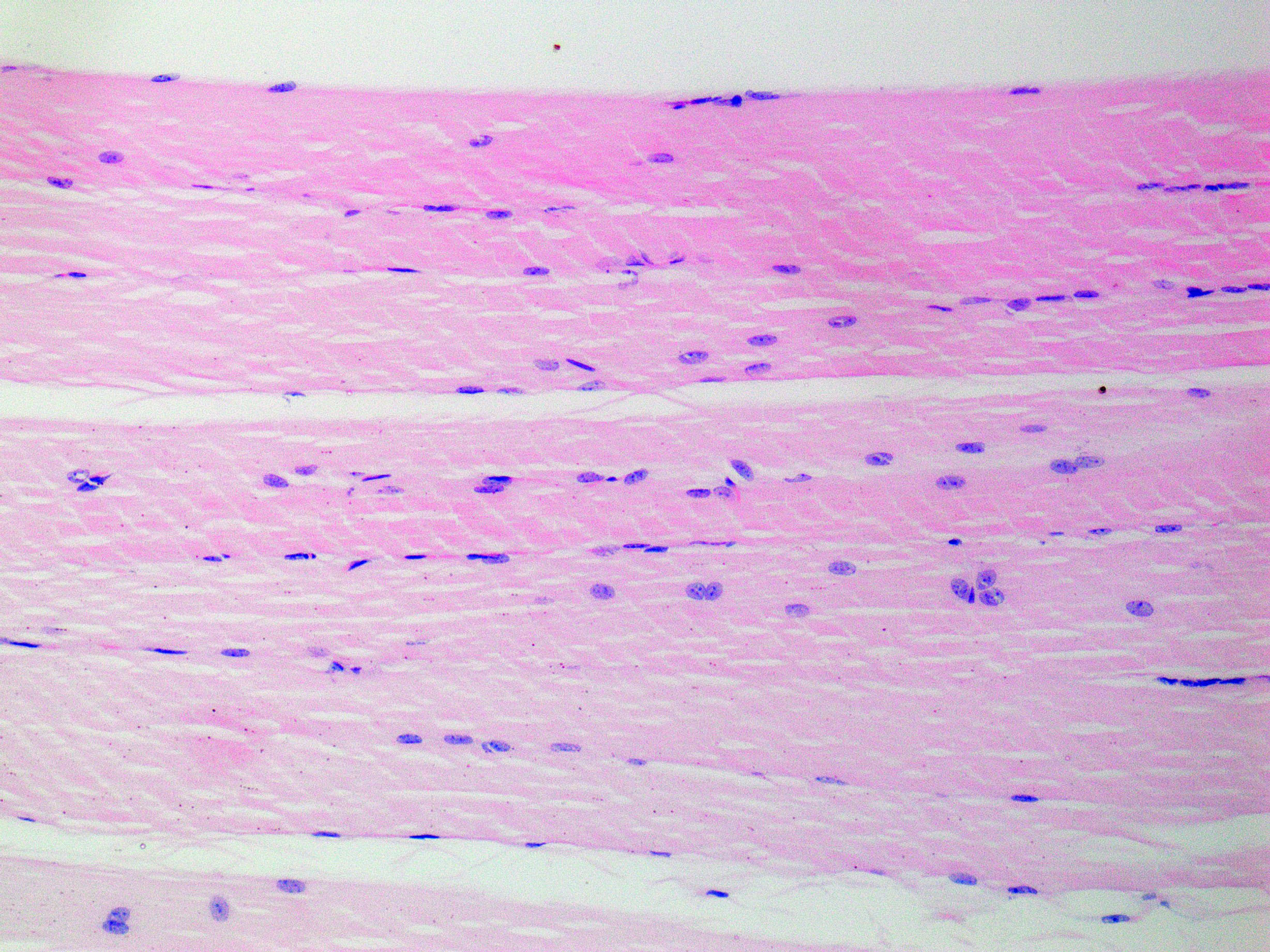

Supplement: Supplementary file 3 [file Data_Sheet_3.ZIP › microscopy images/HE/skeletal muscle/C.jpg]

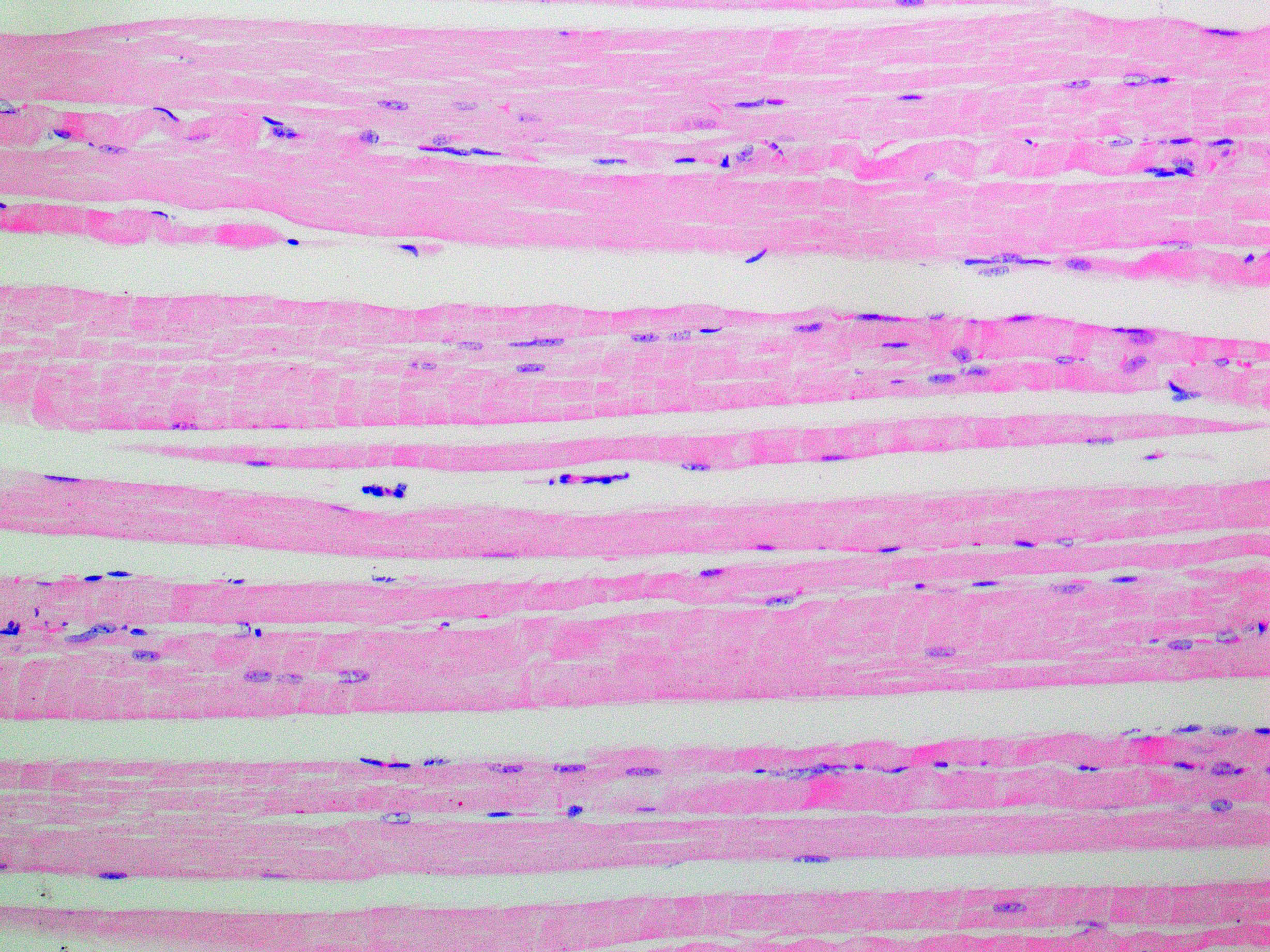

Supplement: Supplementary file 3 [file Data_Sheet_3.ZIP › microscopy images/HE/skeletal muscle/L-LCBP.jpg]

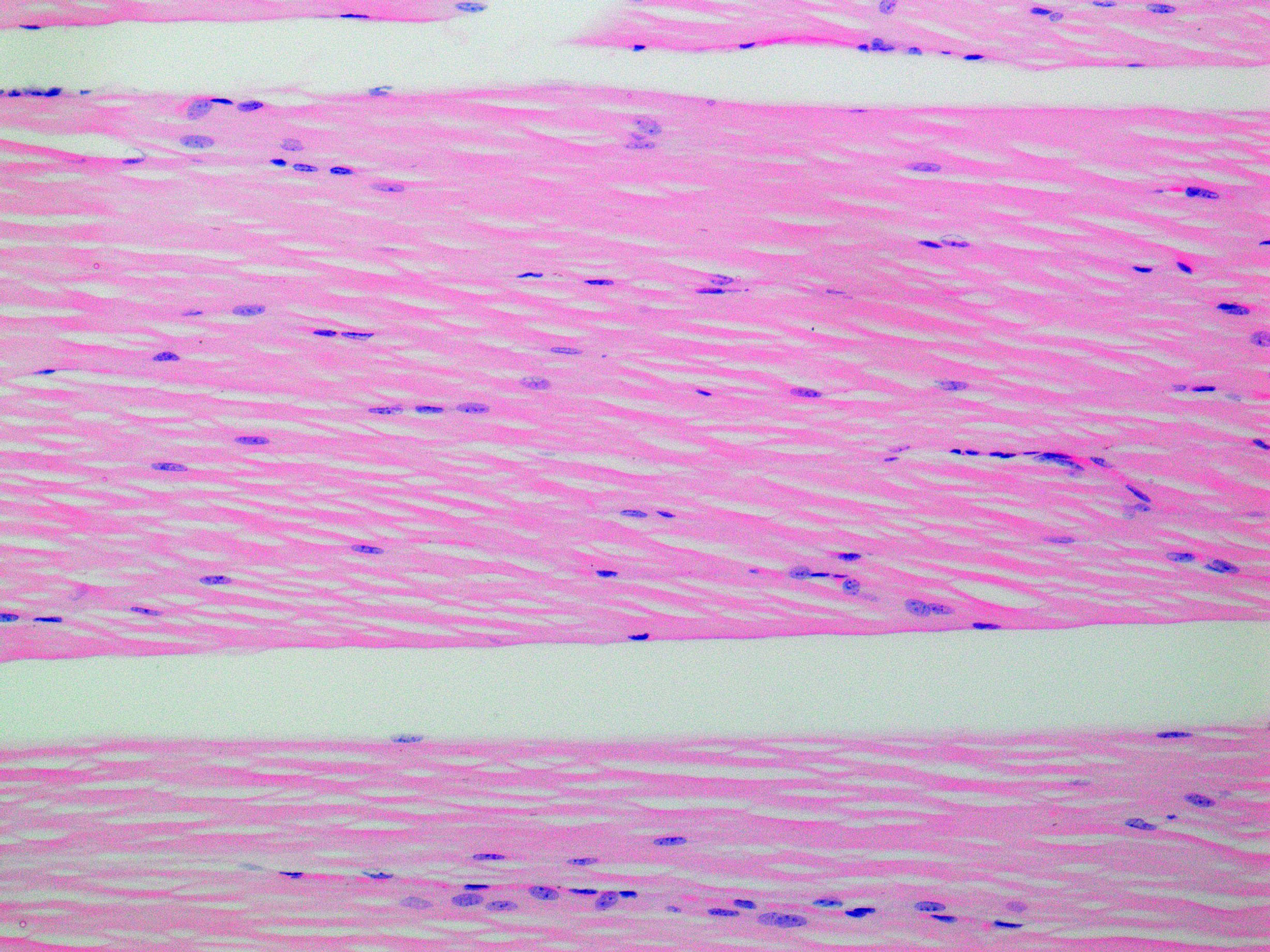

Supplement: Supplementary file 3 [file Data_Sheet_3.ZIP › microscopy images/HE/skeletal muscle/L-M.jpg]

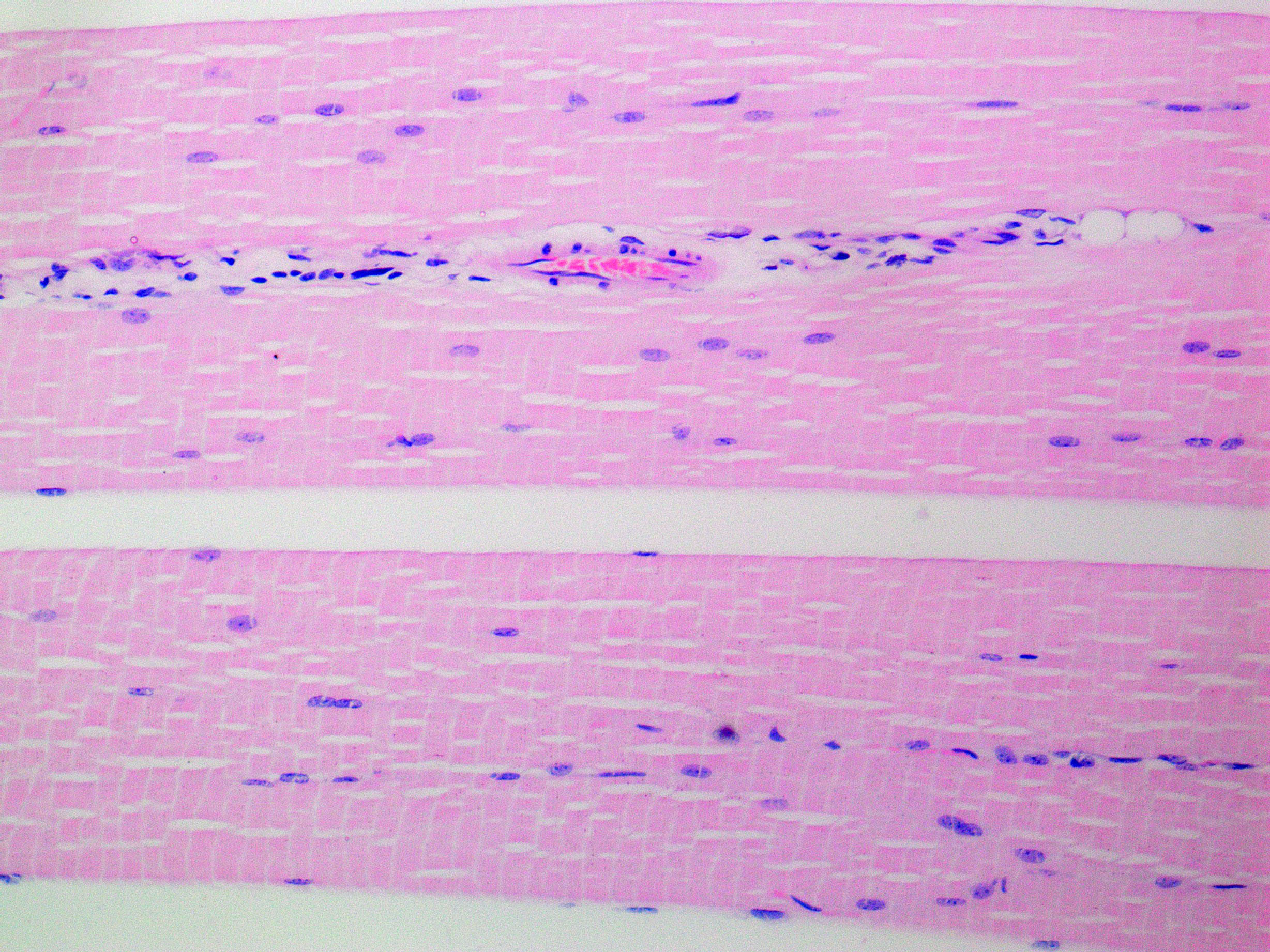

Supplement: Supplementary file 3 [file Data_Sheet_3.ZIP › microscopy images/HE/skeletal muscle/L-VC.jpg]

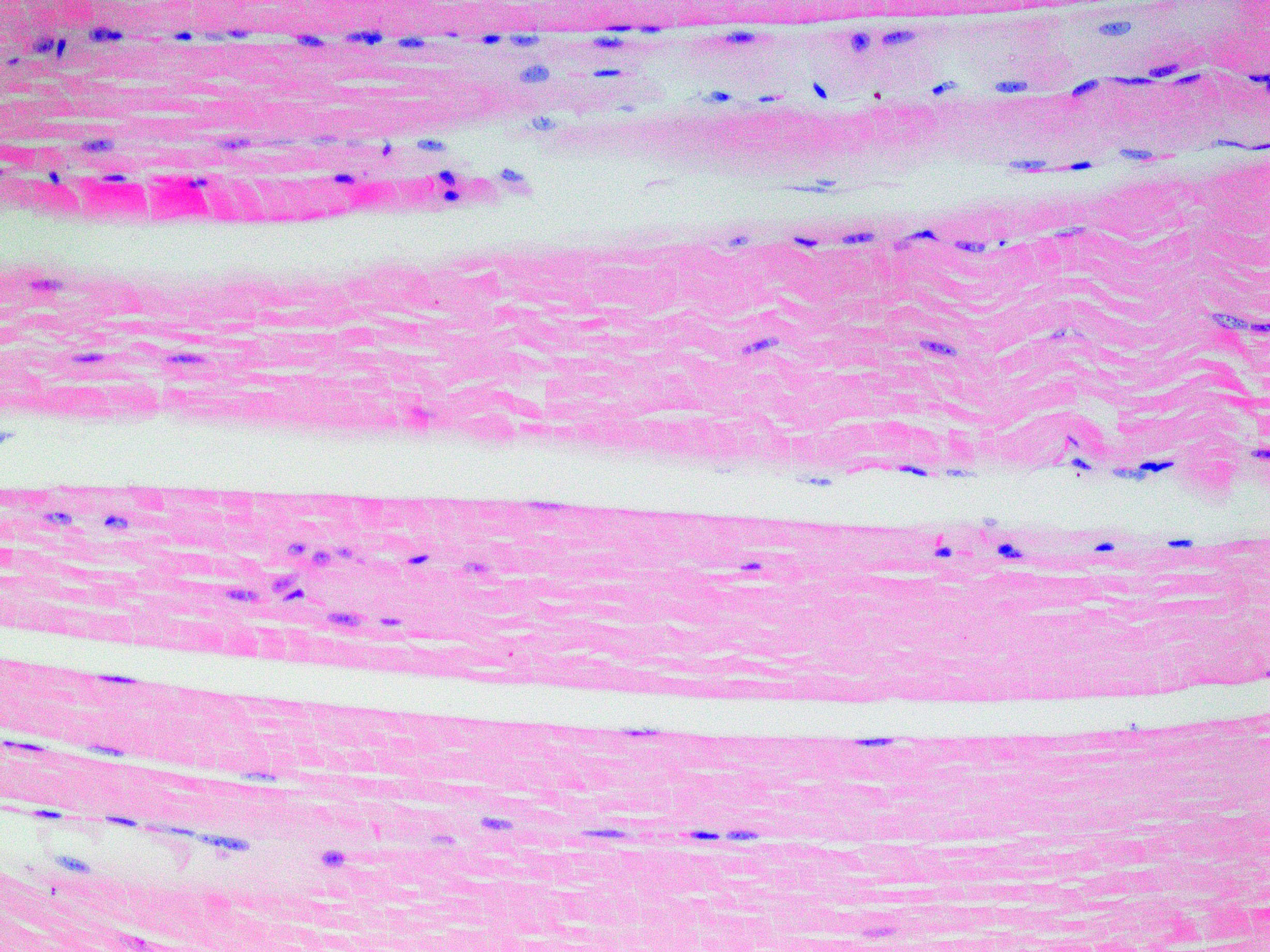

Supplement: Supplementary file 3 [file Data_Sheet_3.ZIP › microscopy images/HE/skeletal muscle/LCBP.jpg]

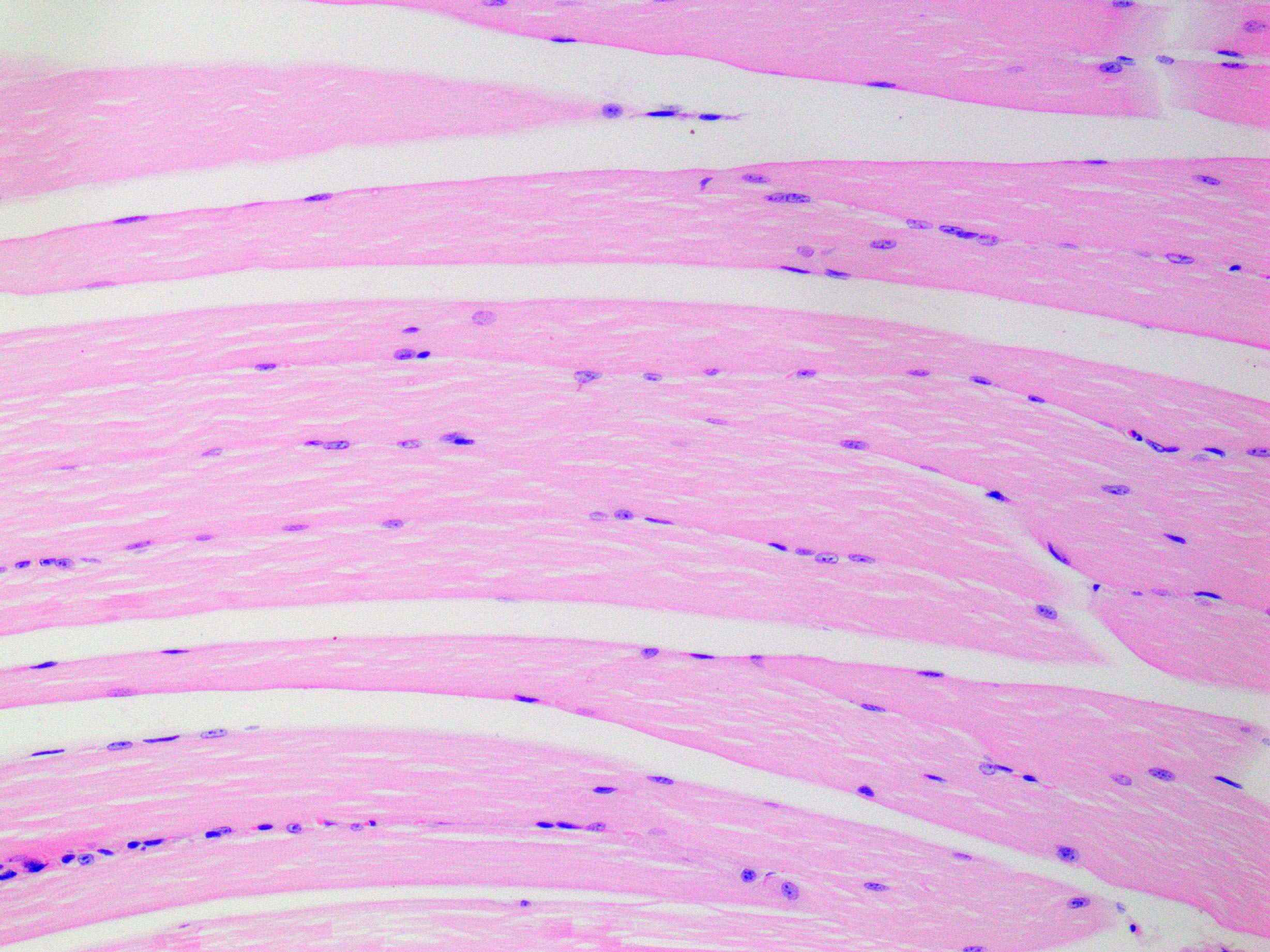

Supplement: Supplementary file 3 [file Data_Sheet_3.ZIP › microscopy images/HE/skeletal muscle/M.jpg]

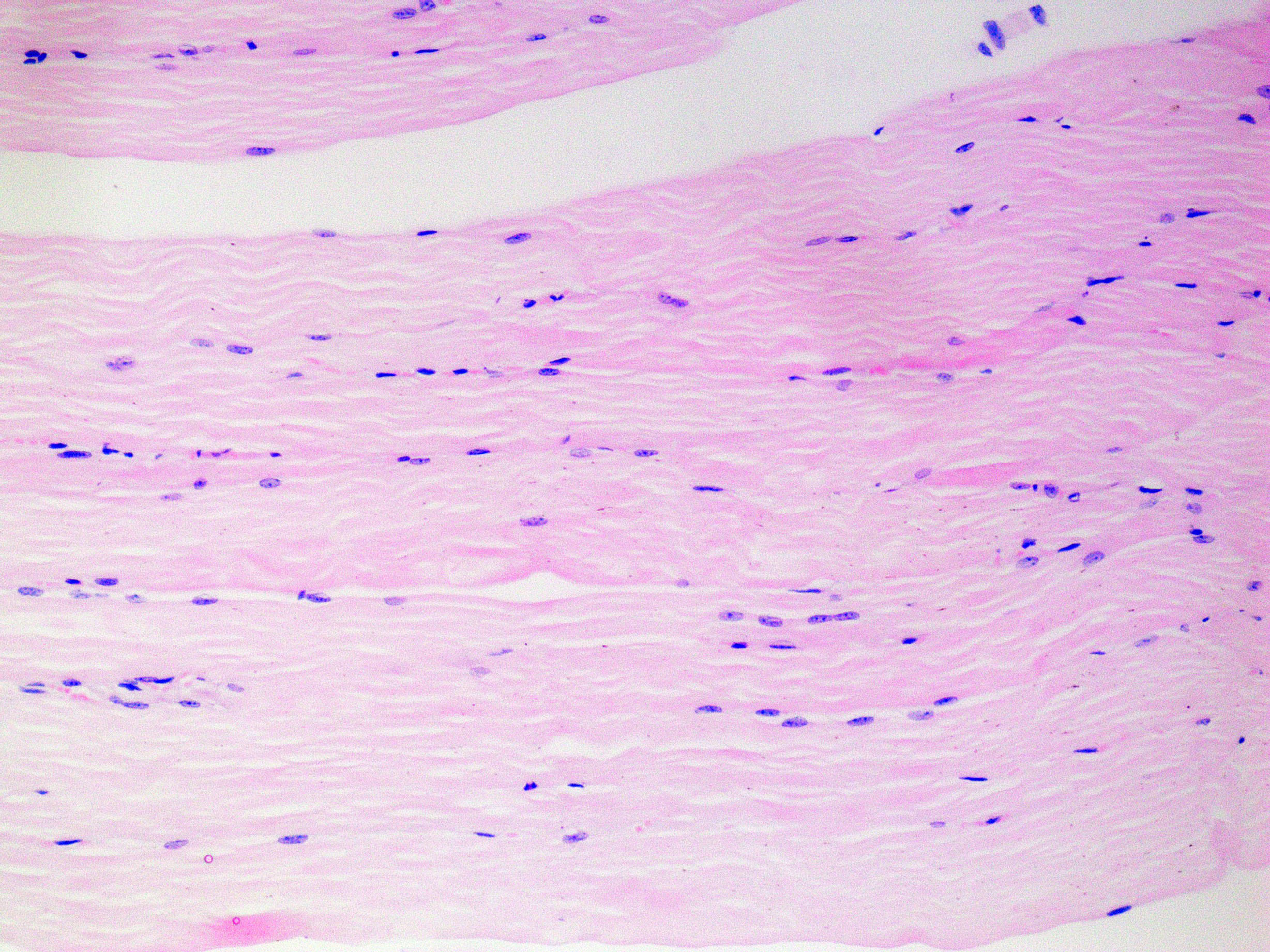

Supplement: Supplementary file 3 [file Data_Sheet_3.ZIP › microscopy images/HE/skeletal muscle/VC.jpg]

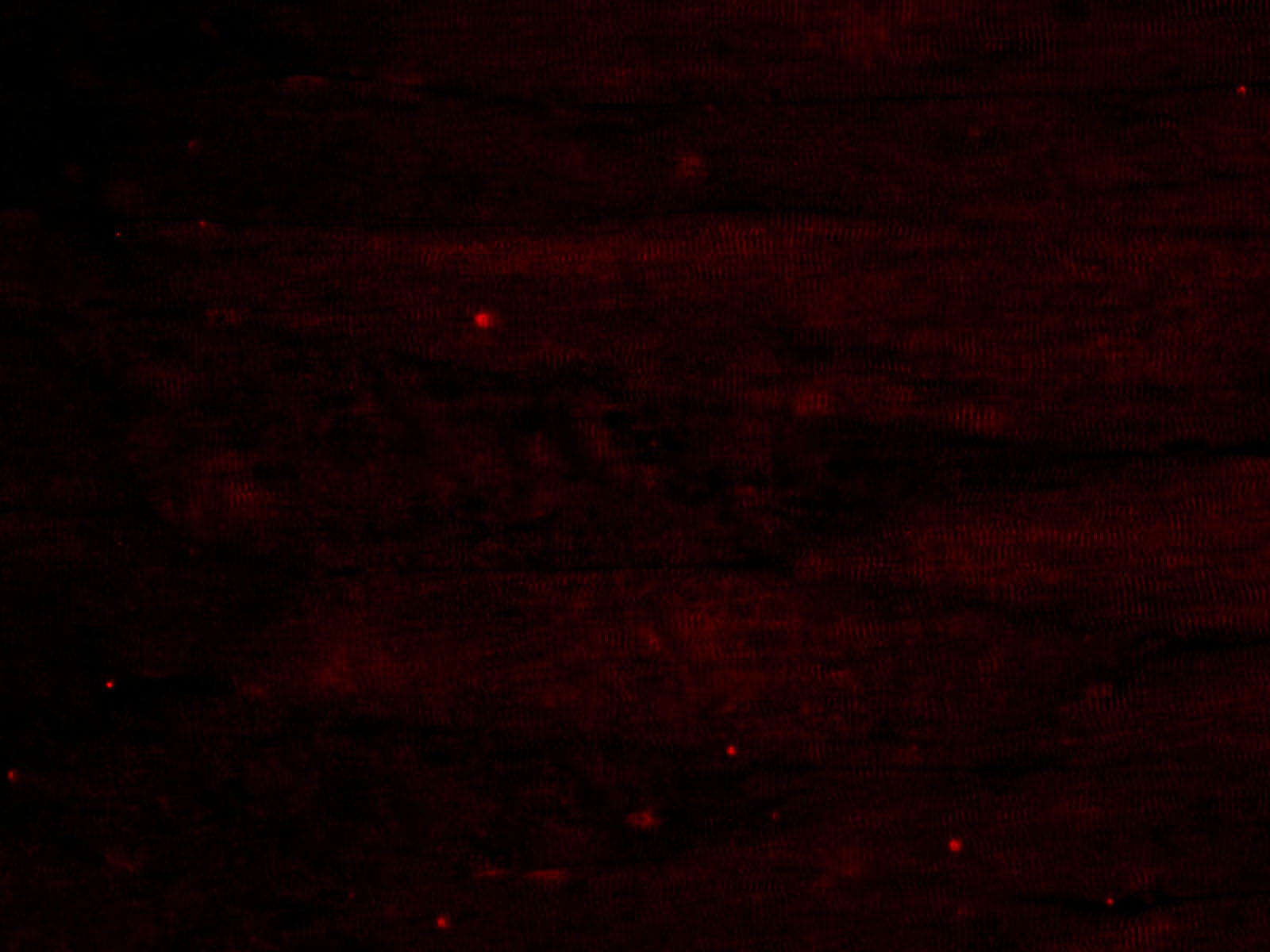

Supplement: Supplementary file 3 [file Data_Sheet_3.ZIP › microscopy images/IF/PKCα(red light)+Nox2(green light)/C (1).jpg]

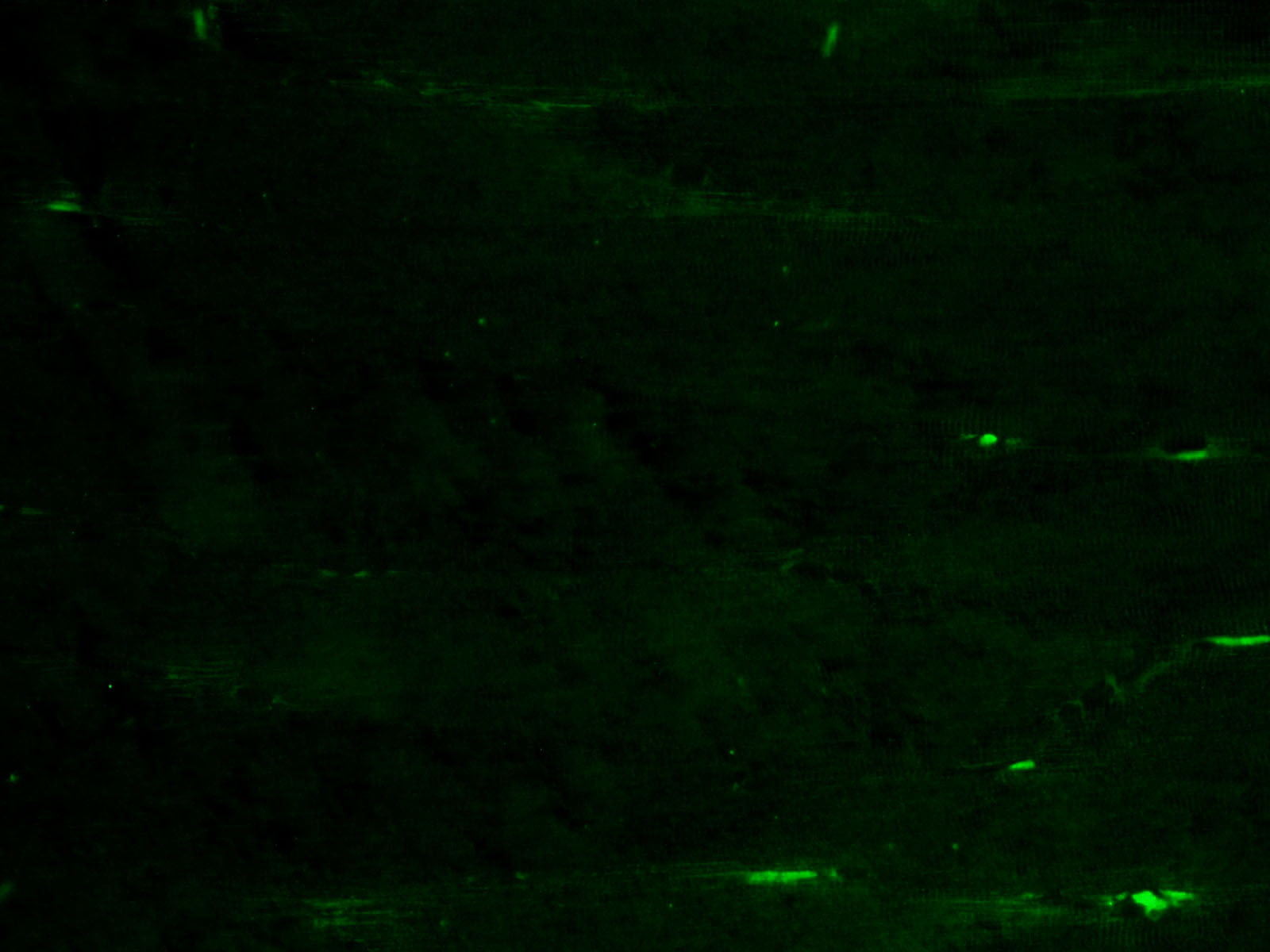

Supplement: Supplementary file 3 [file Data_Sheet_3.ZIP › microscopy images/IF/PKCα(red light)+Nox2(green light)/C (2).jpg]

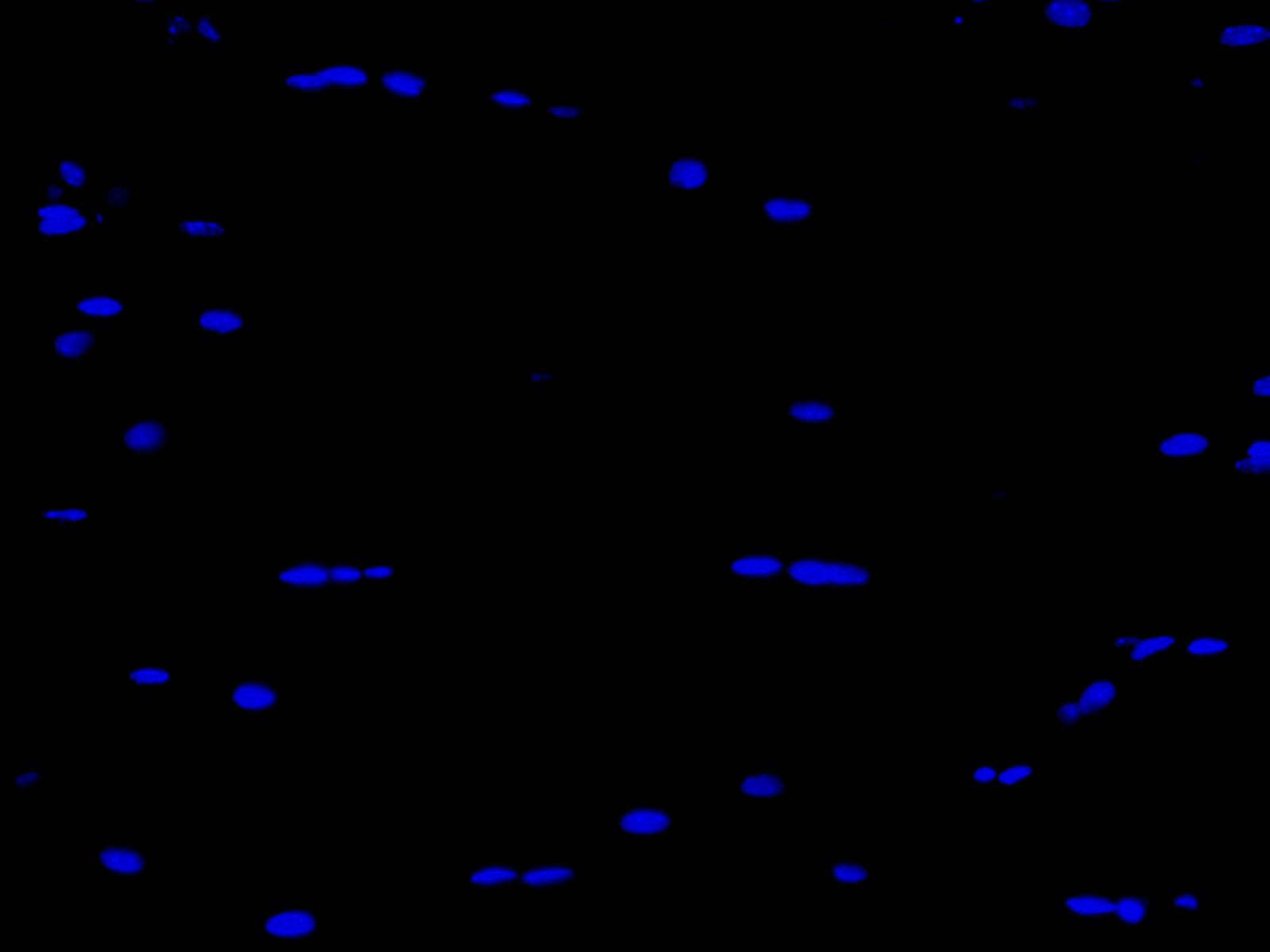

Supplement: Supplementary file 3 [file Data_Sheet_3.ZIP › microscopy images/IF/PKCα(red light)+Nox2(green light)/C (3).jpg]

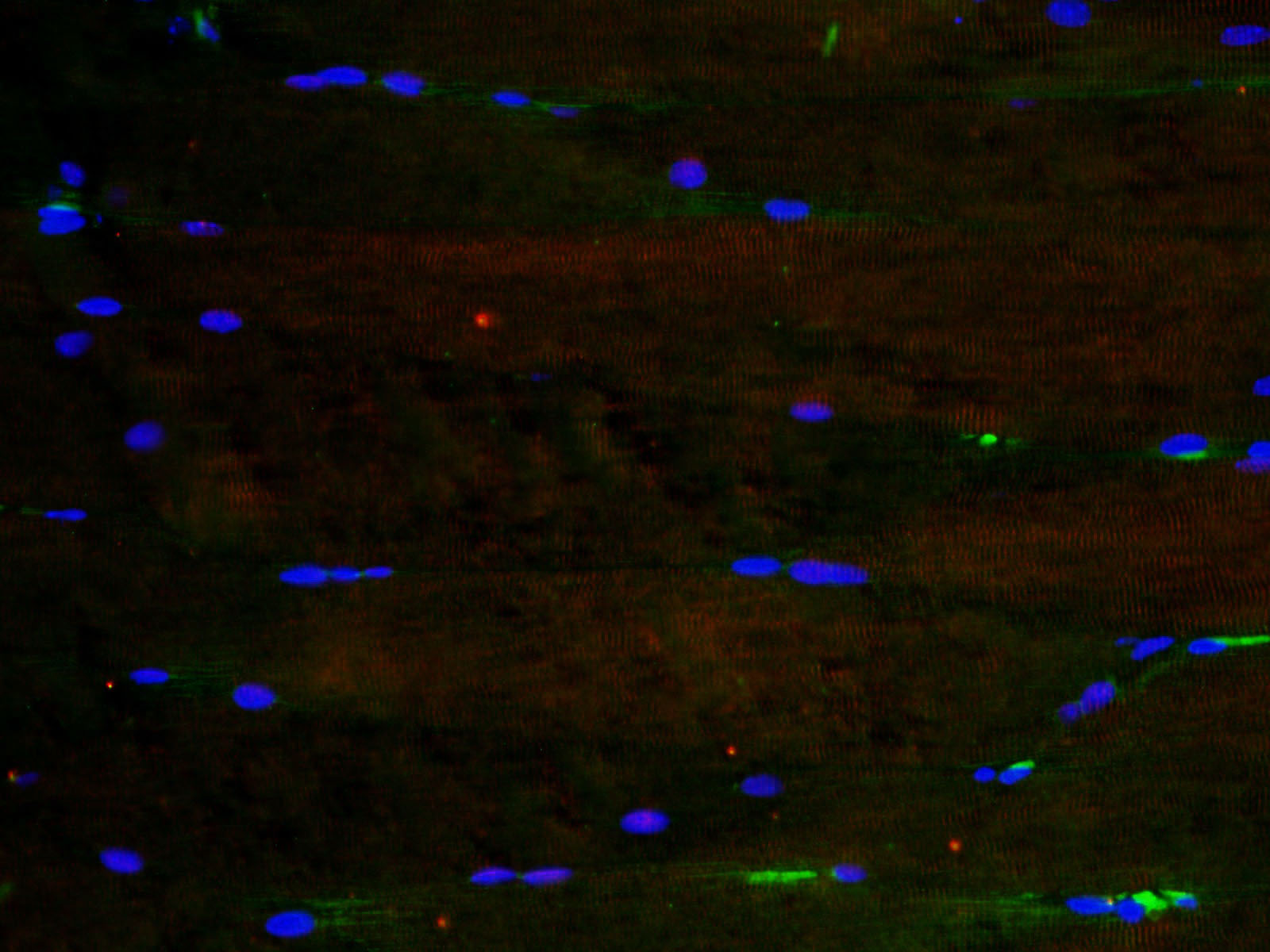

Supplement: Supplementary file 3 [file Data_Sheet_3.ZIP › microscopy images/IF/PKCα(red light)+Nox2(green light)/C (4).jpg]

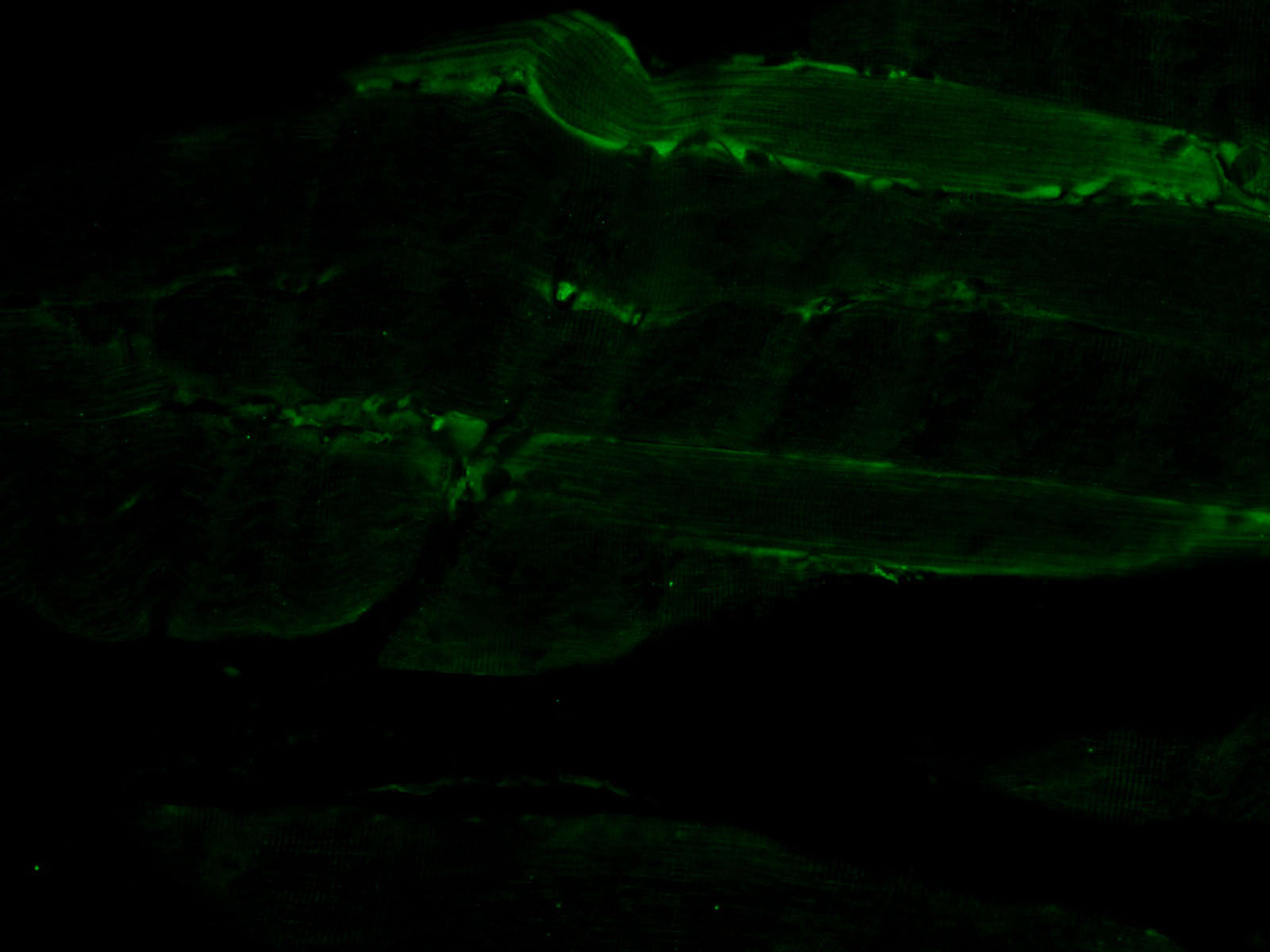

Supplement: Supplementary file 3 [file Data_Sheet_3.ZIP › microscopy images/IF/PKCα(red light)+Nox2(green light)/L-LCBP (2).jpg]

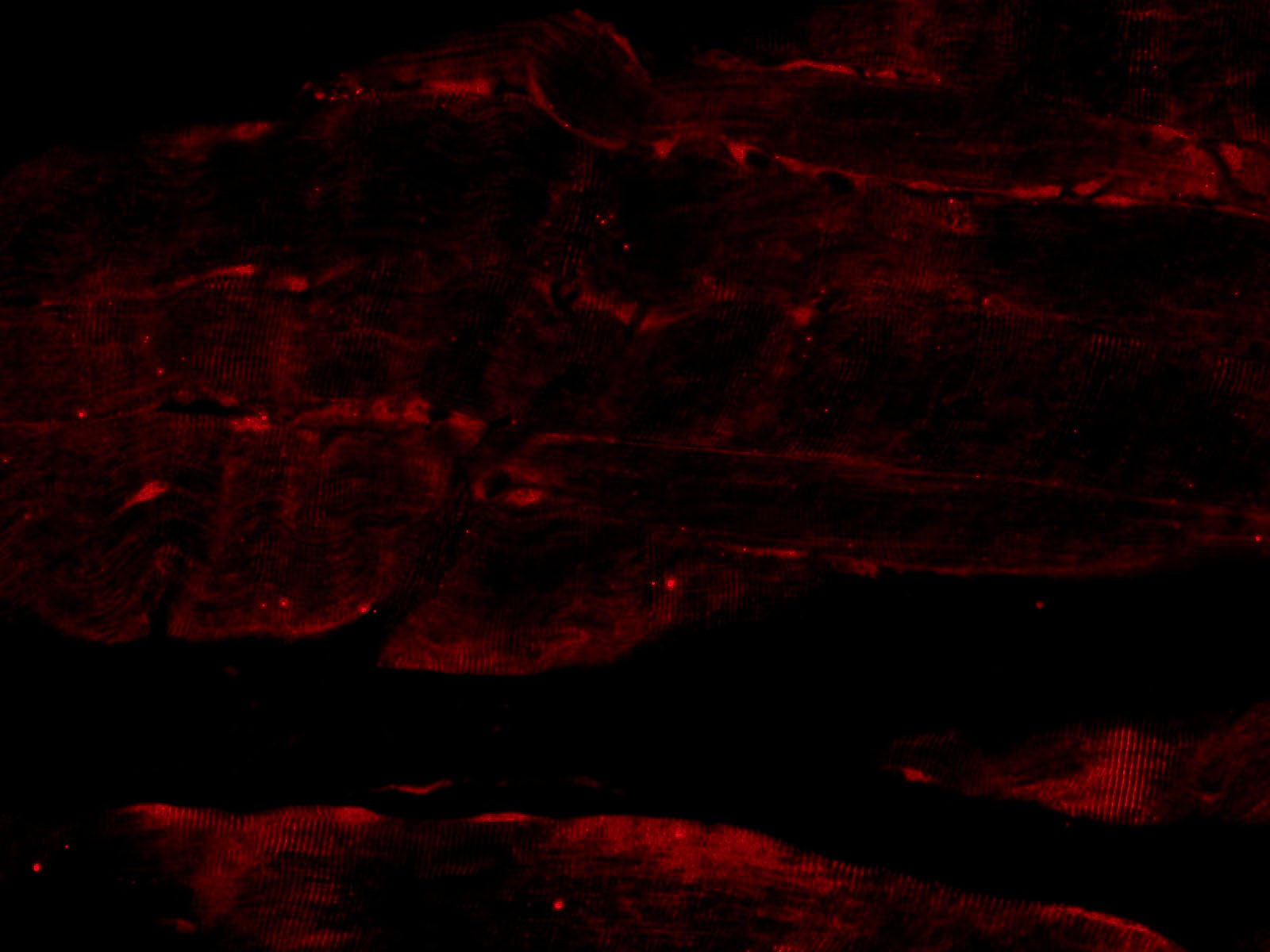

Supplement: Supplementary file 3 [file Data_Sheet_3.ZIP › microscopy images/IF/PKCα(red light)+Nox2(green light)/LCBP (1).jpg]

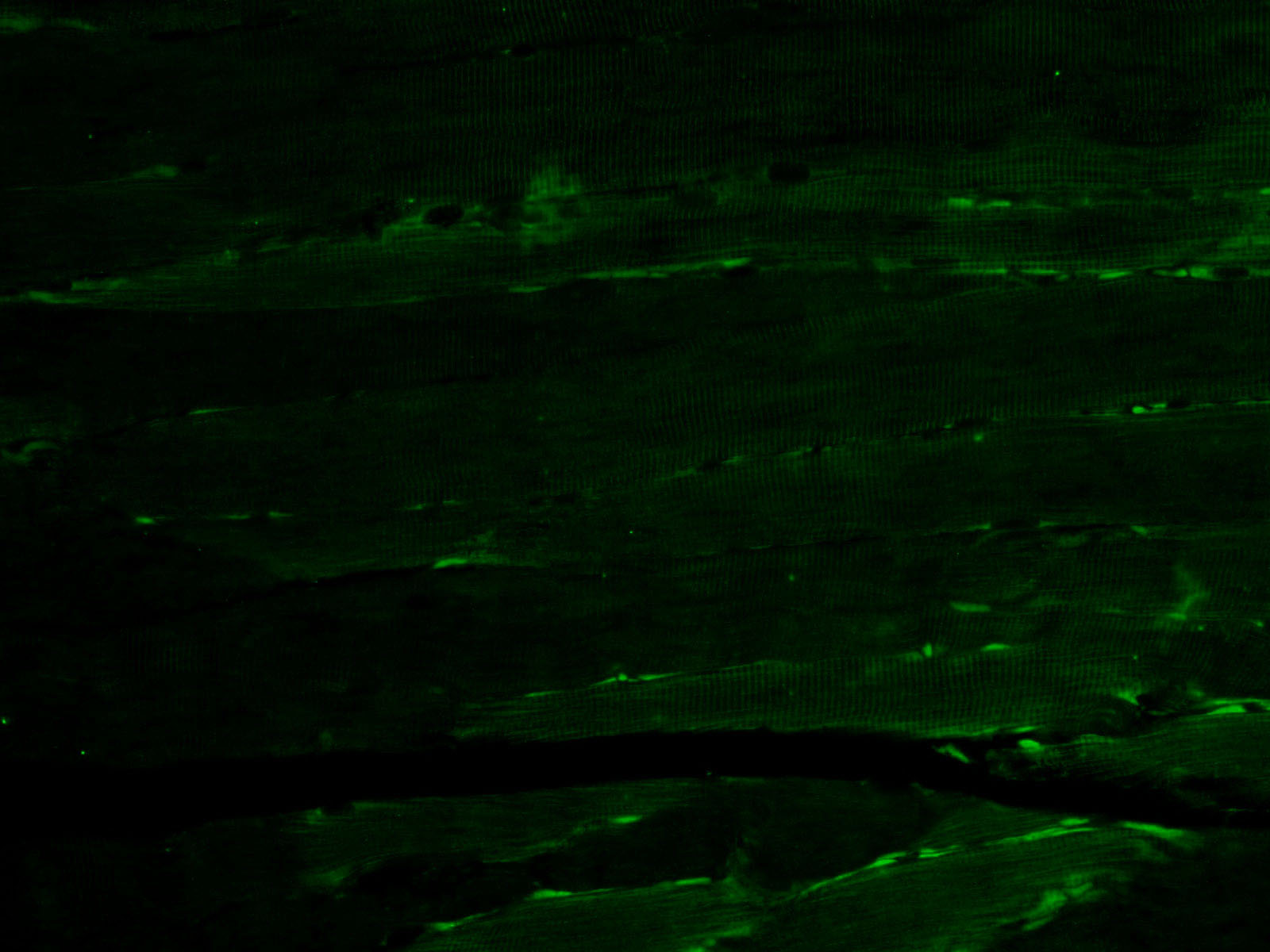

Supplement: Supplementary file 3 [file Data_Sheet_3.ZIP › microscopy images/IF/PKCα(red light)+Nox2(green light)/LCBP (2).jpg]

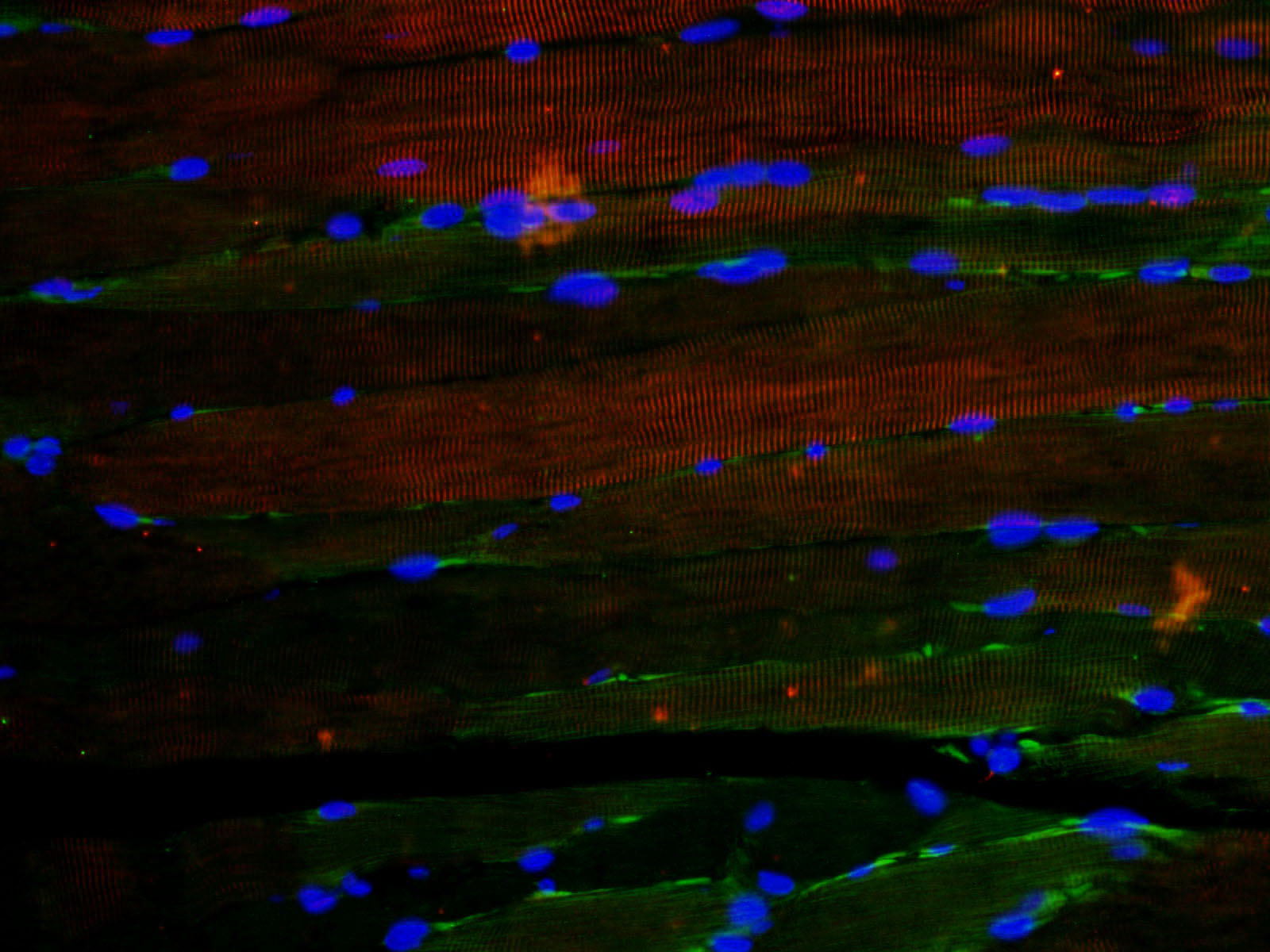

Supplement: Supplementary file 3 [file Data_Sheet_3.ZIP › microscopy images/IF/PKCα(red light)+Nox2(green light)/LCBP (4).jpg]

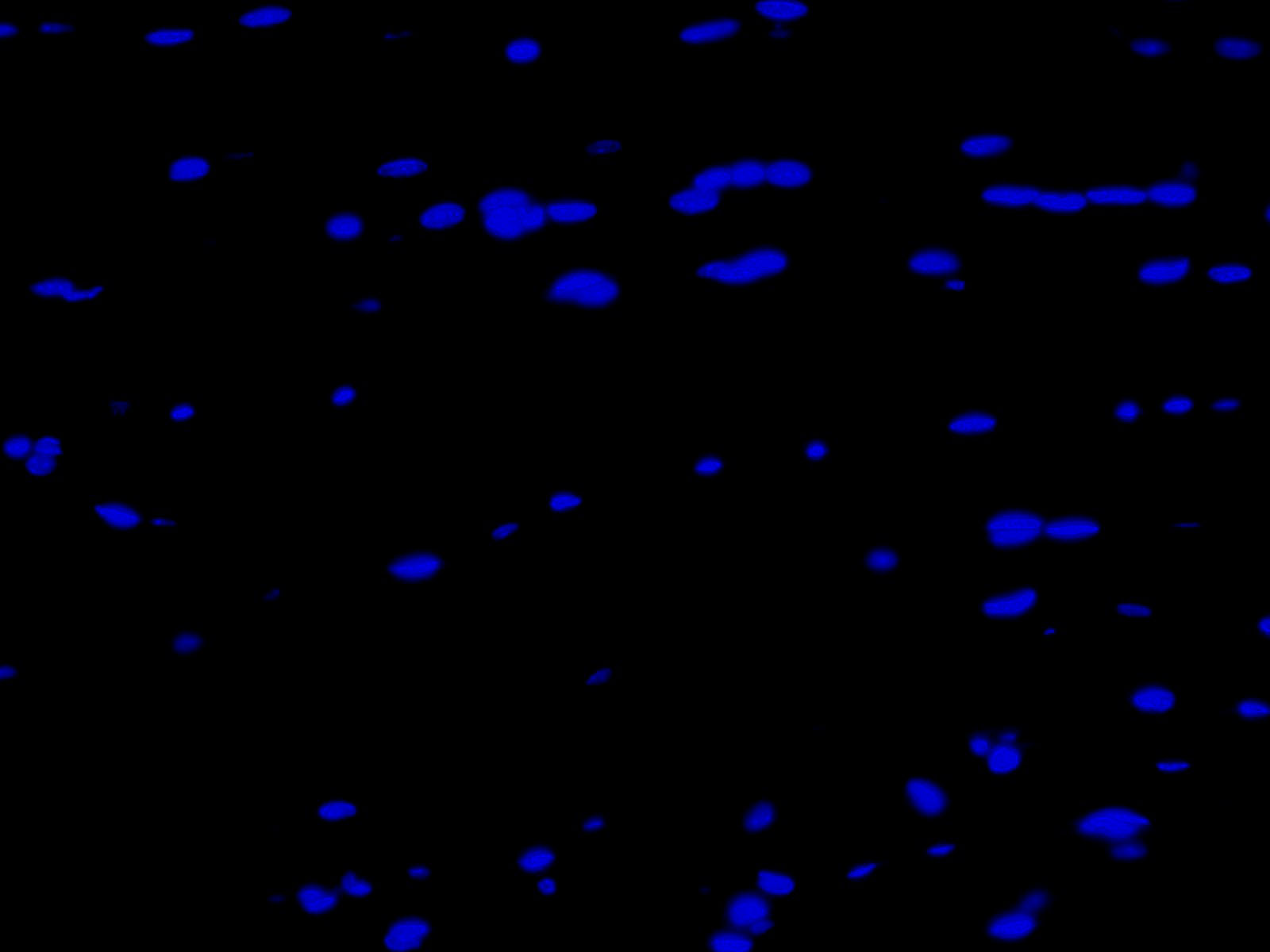

Supplement: Supplementary file 3 [file Data_Sheet_3.ZIP › microscopy images/IF/PKCα(red light)+Nox2(green light)/LCBP(3).jpg]

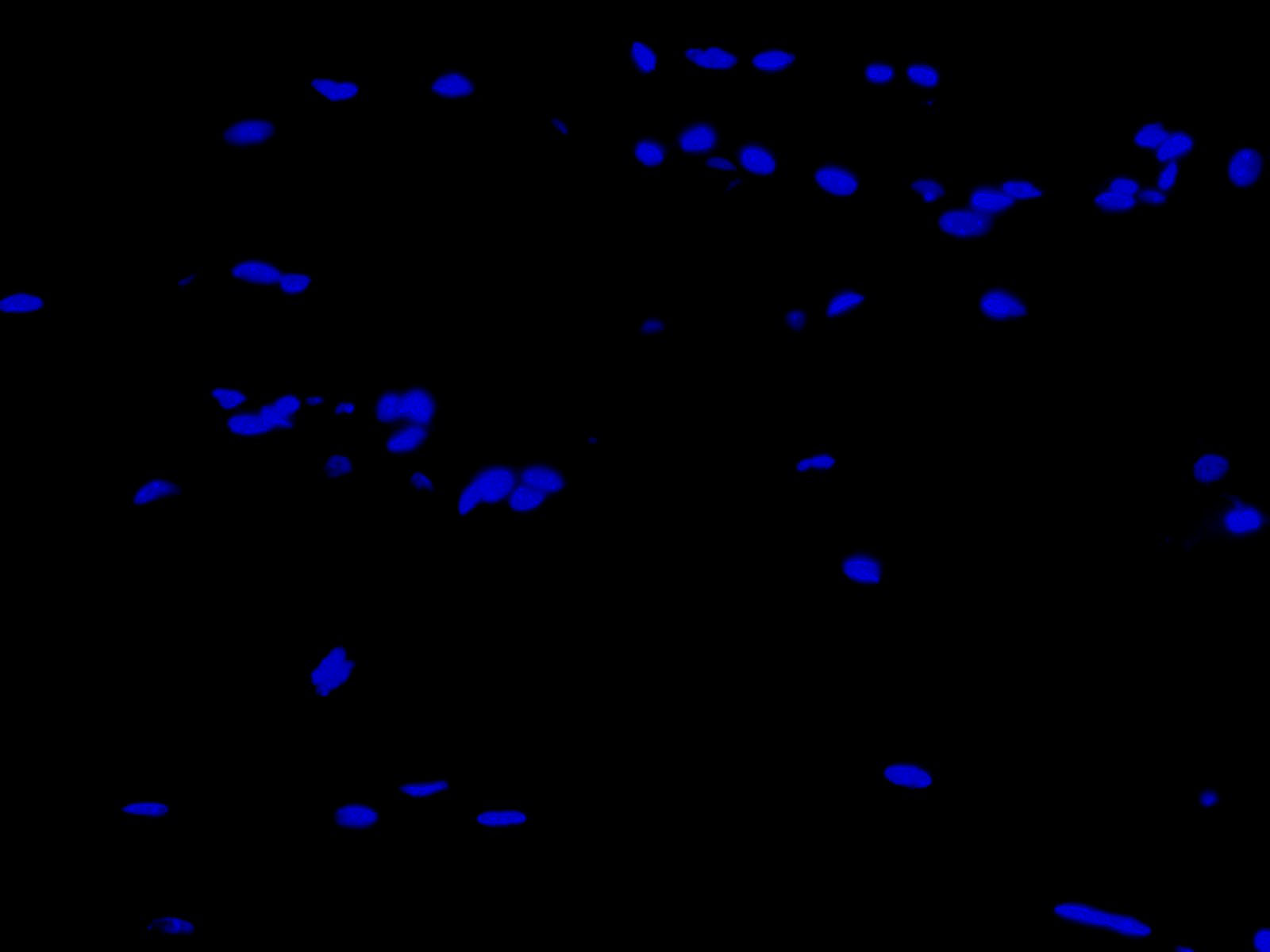

Supplement: Supplementary file 3 [file Data_Sheet_3.ZIP › microscopy images/IF/PKCα(red light)+Nox2(green light)/LLCBP (3).jpg]

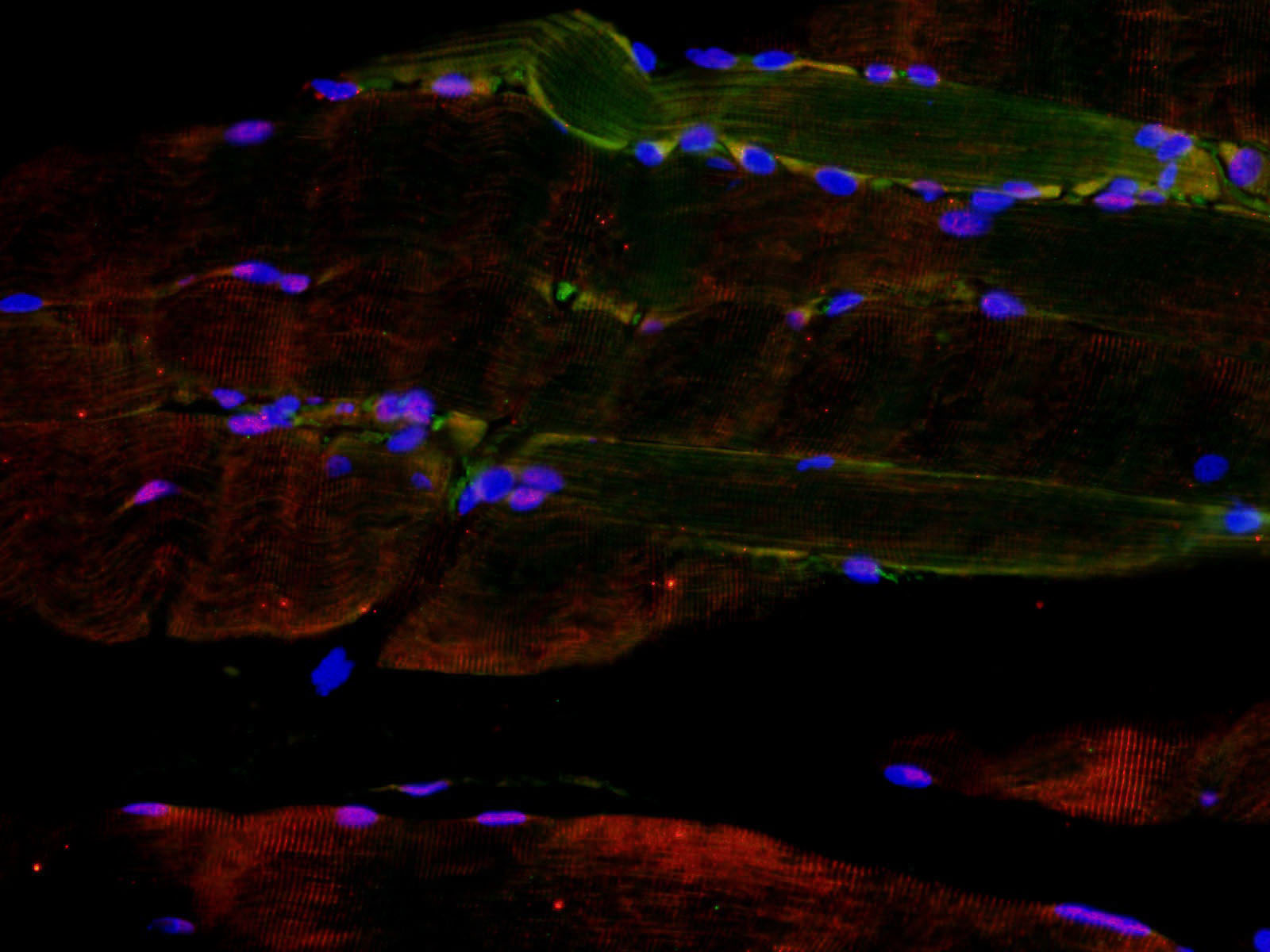

Supplement: Supplementary file 3 [file Data_Sheet_3.ZIP › microscopy images/IF/PKCα(red light)+Nox2(green light)/LLCBP (4).jpg]

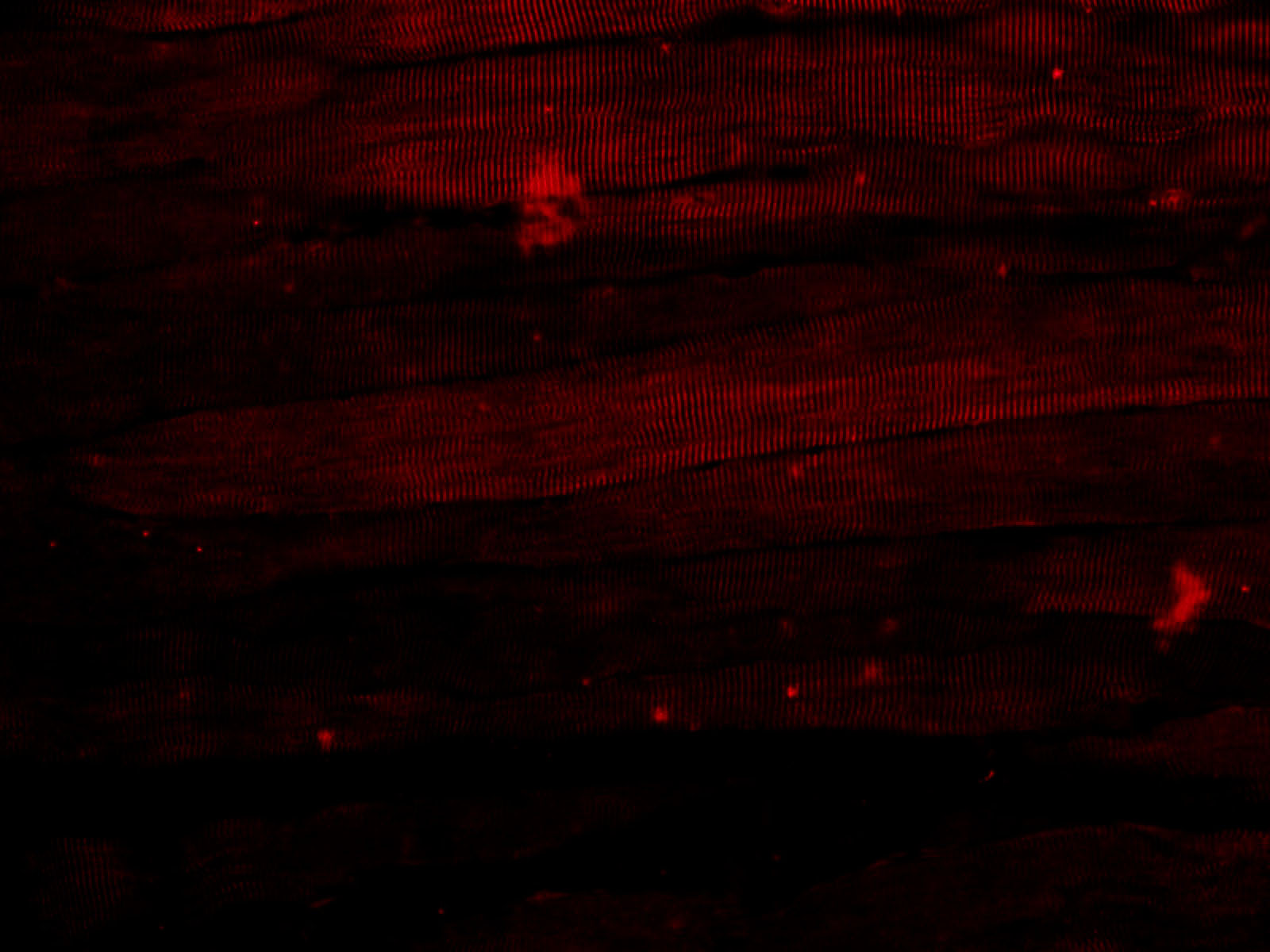

Supplement: Supplementary file 3 [file Data_Sheet_3.ZIP › microscopy images/IF/PKCα(red light)+Nox2(green light)/LLCBP(1).jpg]

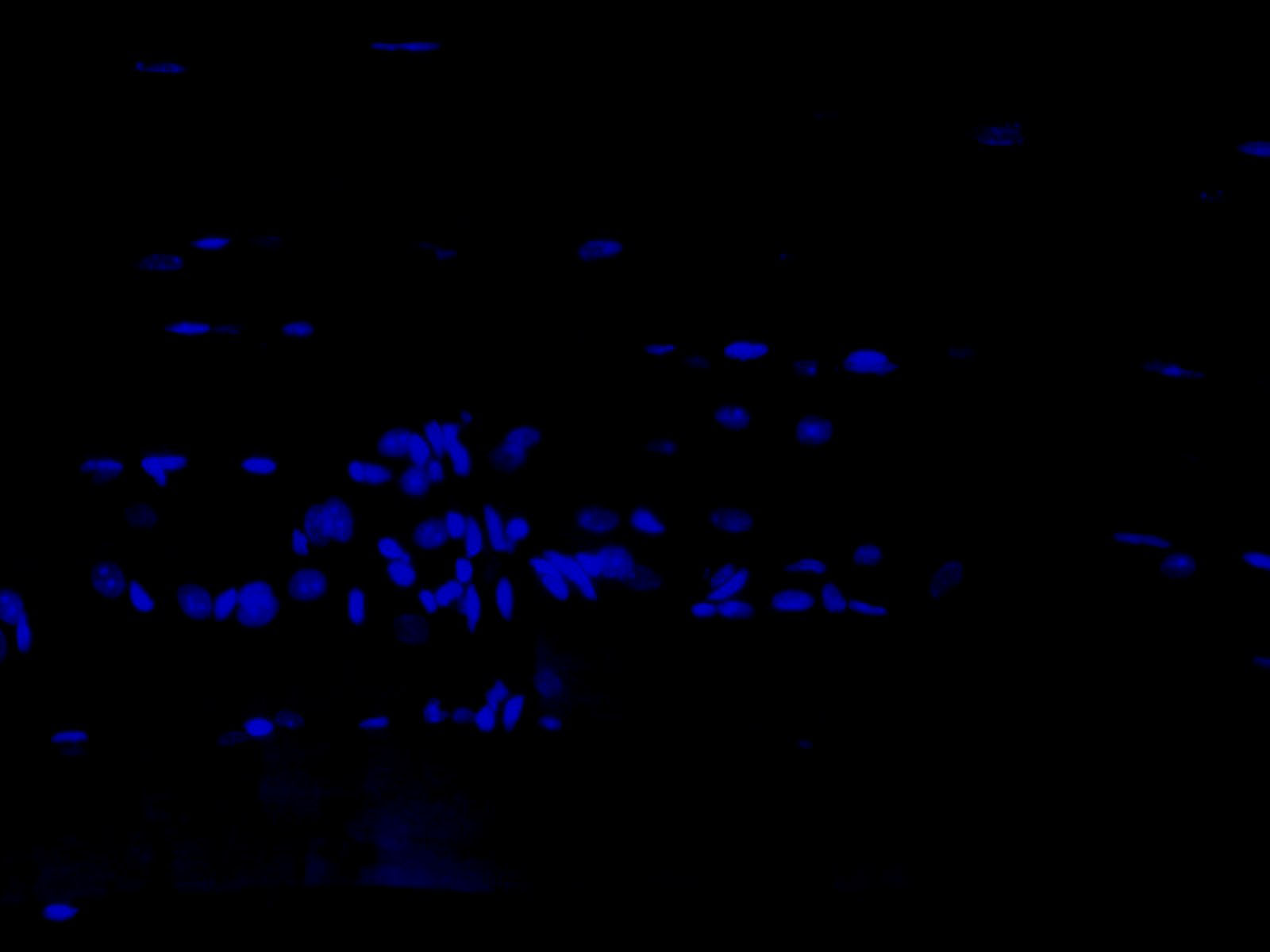

Supplement: Supplementary file 3 [file Data_Sheet_3.ZIP › microscopy images/IF/PKCα(red light)+Nox2(green light)/LM (3).jpg]

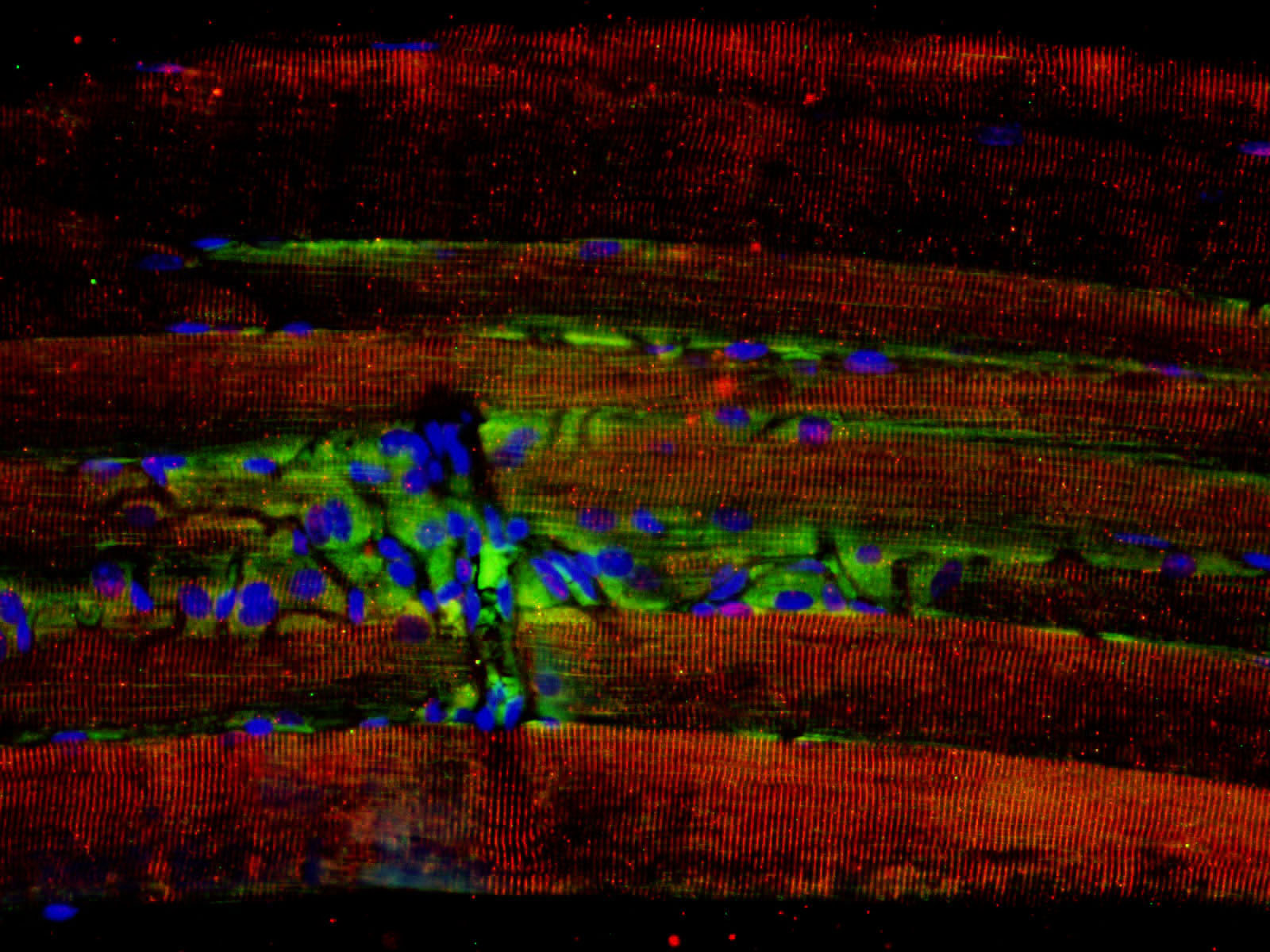

Supplement: Supplementary file 3 [file Data_Sheet_3.ZIP › microscopy images/IF/PKCα(red light)+Nox2(green light)/LM (4).jpg]

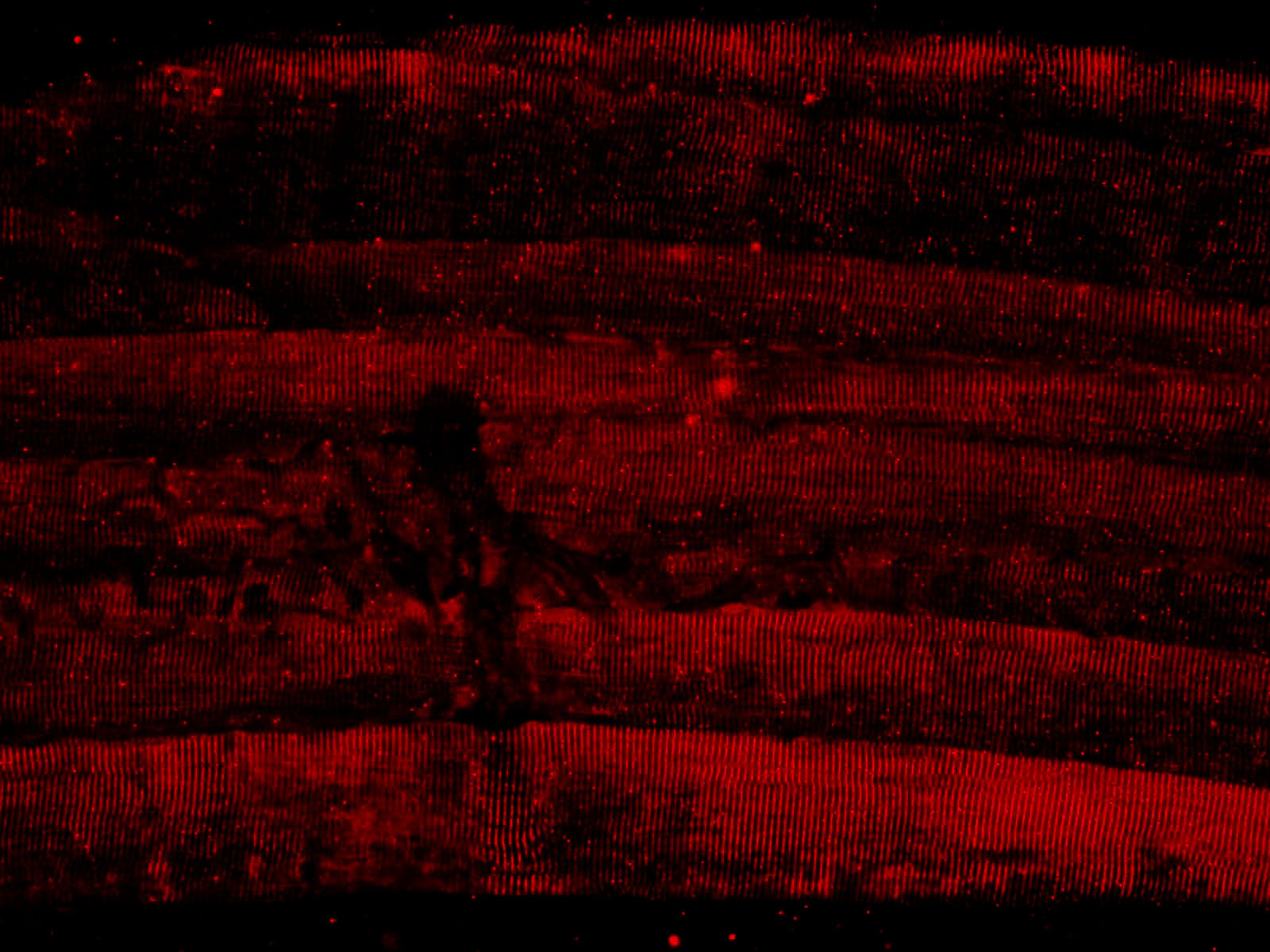

Supplement: Supplementary file 3 [file Data_Sheet_3.ZIP › microscopy images/IF/PKCα(red light)+Nox2(green light)/LM.jpg]

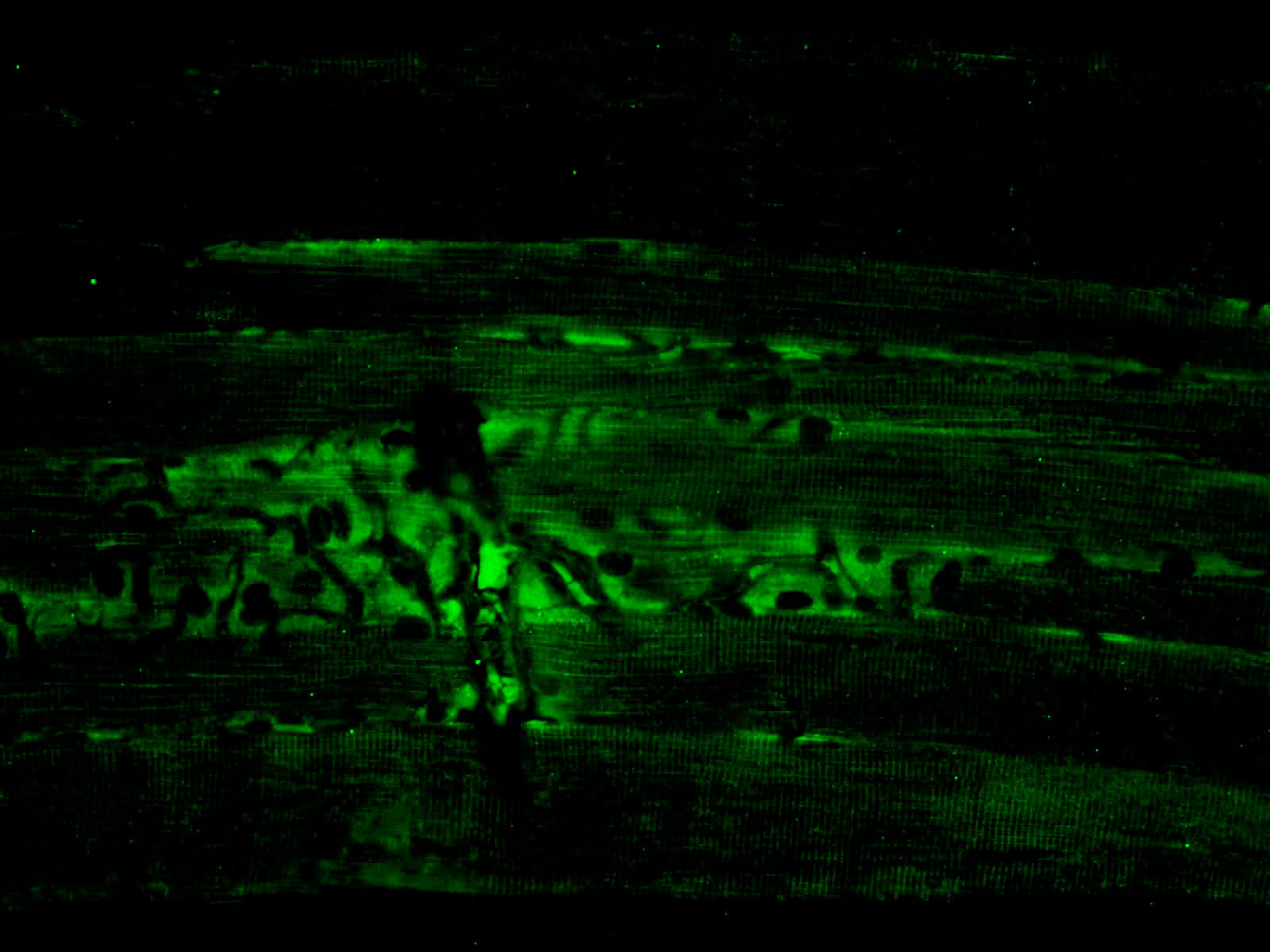

Supplement: Supplementary file 3 [file Data_Sheet_3.ZIP › microscopy images/IF/PKCα(red light)+Nox2(green light)/LM2.jpg]

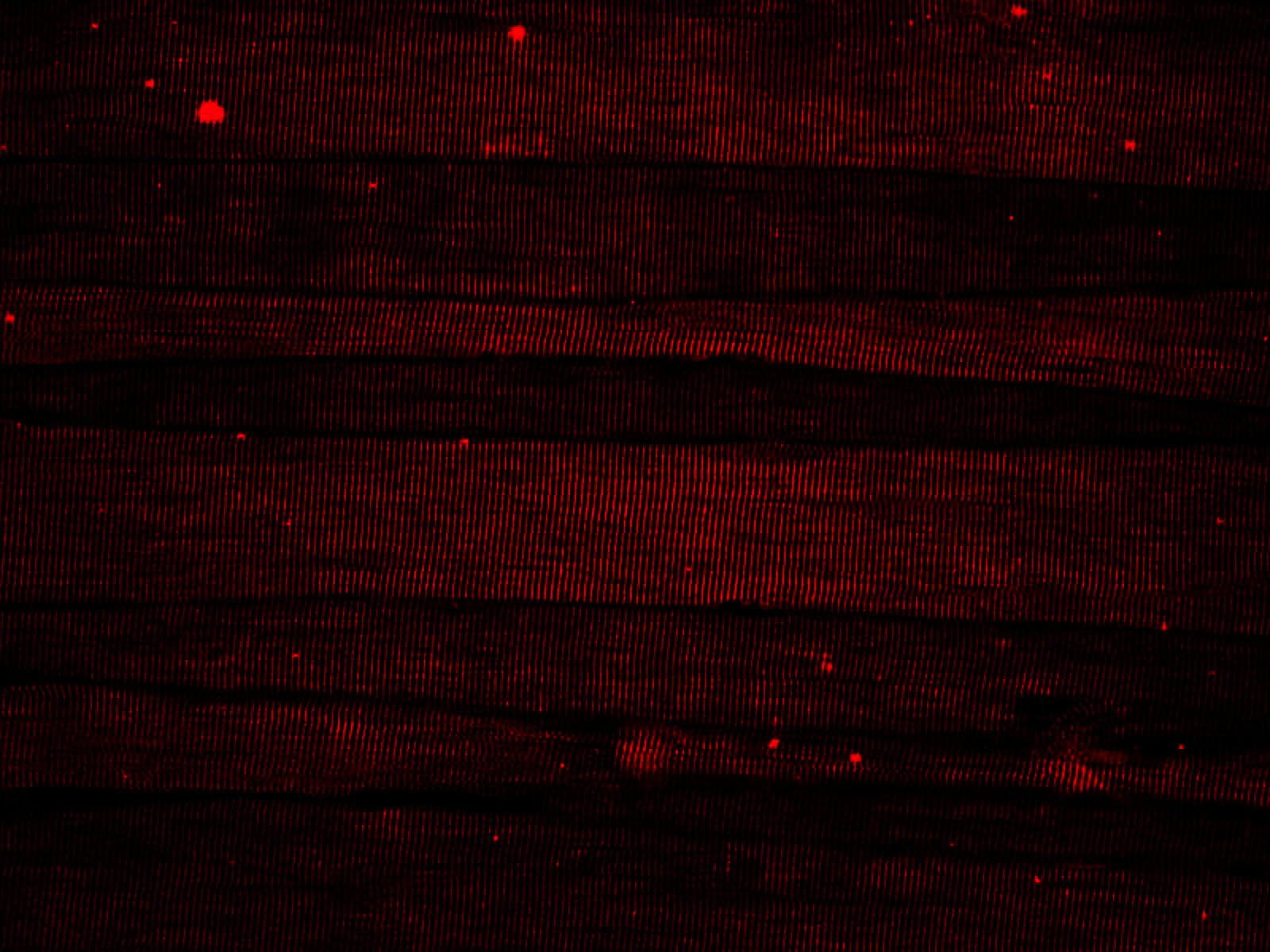

Supplement: Supplementary file 3 [file Data_Sheet_3.ZIP › microscopy images/IF/PKCα(red light)+Nox2(green light)/LVC (1).jpg]

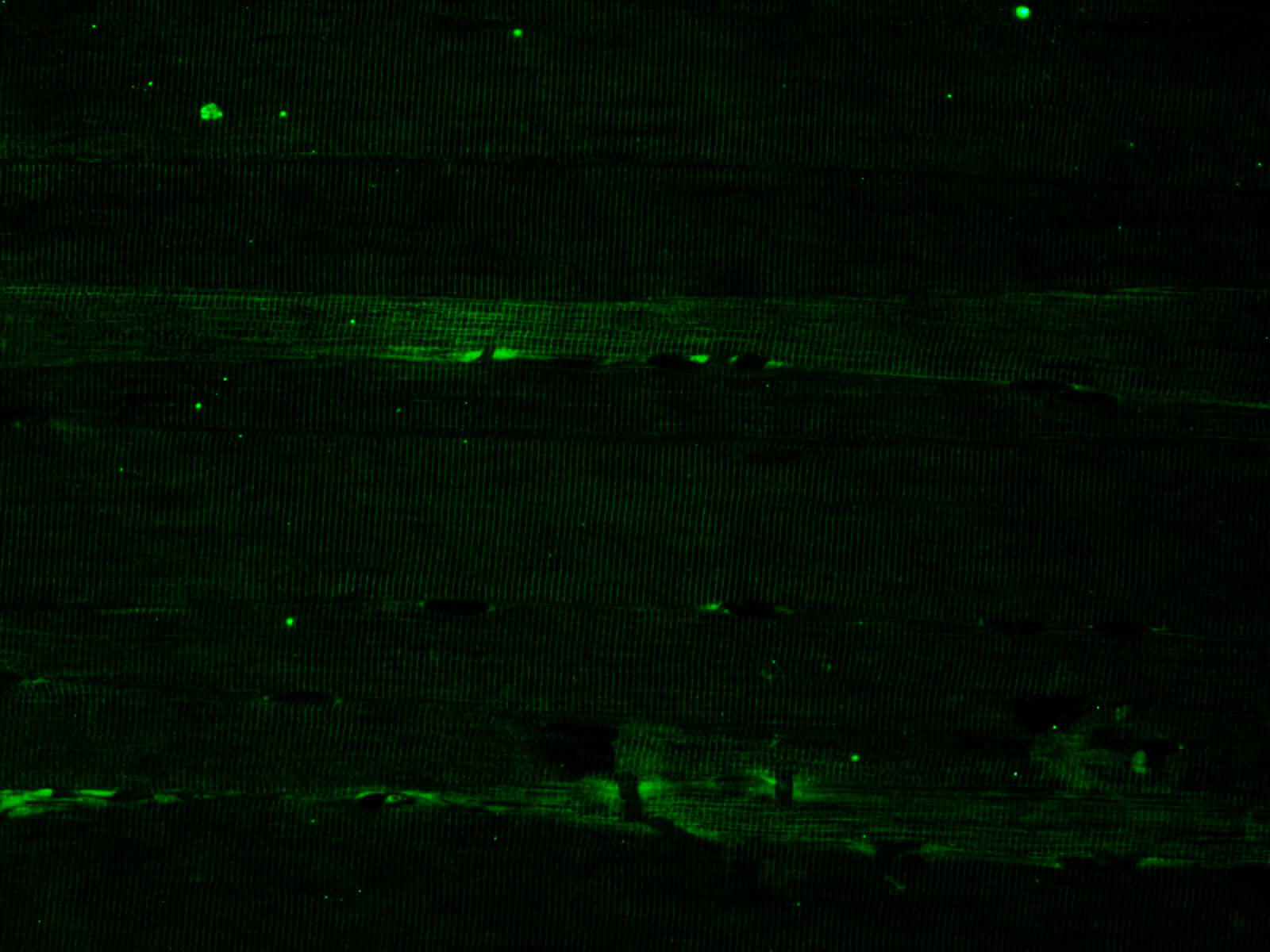

Supplement: Supplementary file 3 [file Data_Sheet_3.ZIP › microscopy images/IF/PKCα(red light)+Nox2(green light)/LVC (2).jpg]

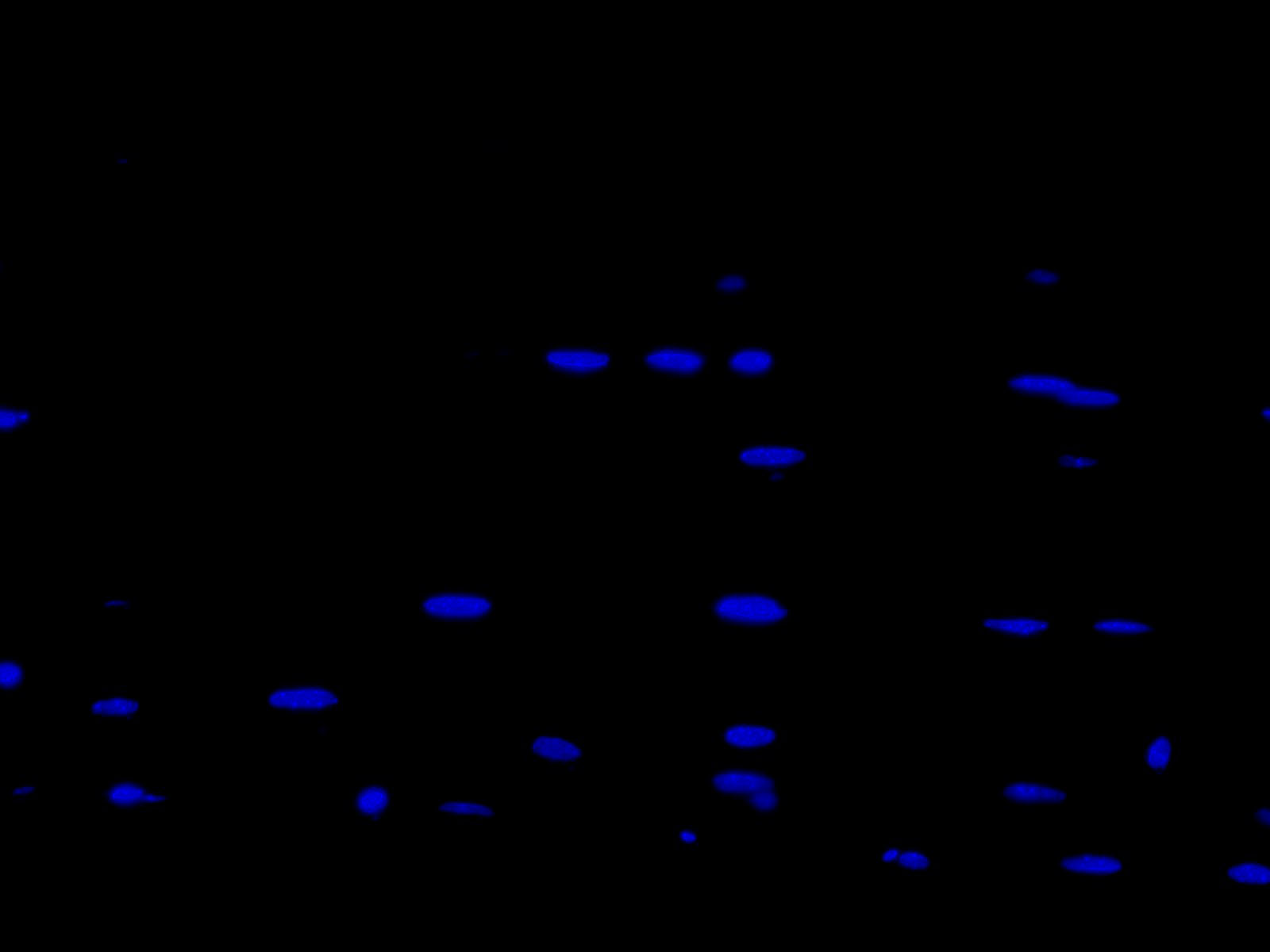

Supplement: Supplementary file 3 [file Data_Sheet_3.ZIP › microscopy images/IF/PKCα(red light)+Nox2(green light)/LVC (3).jpg]

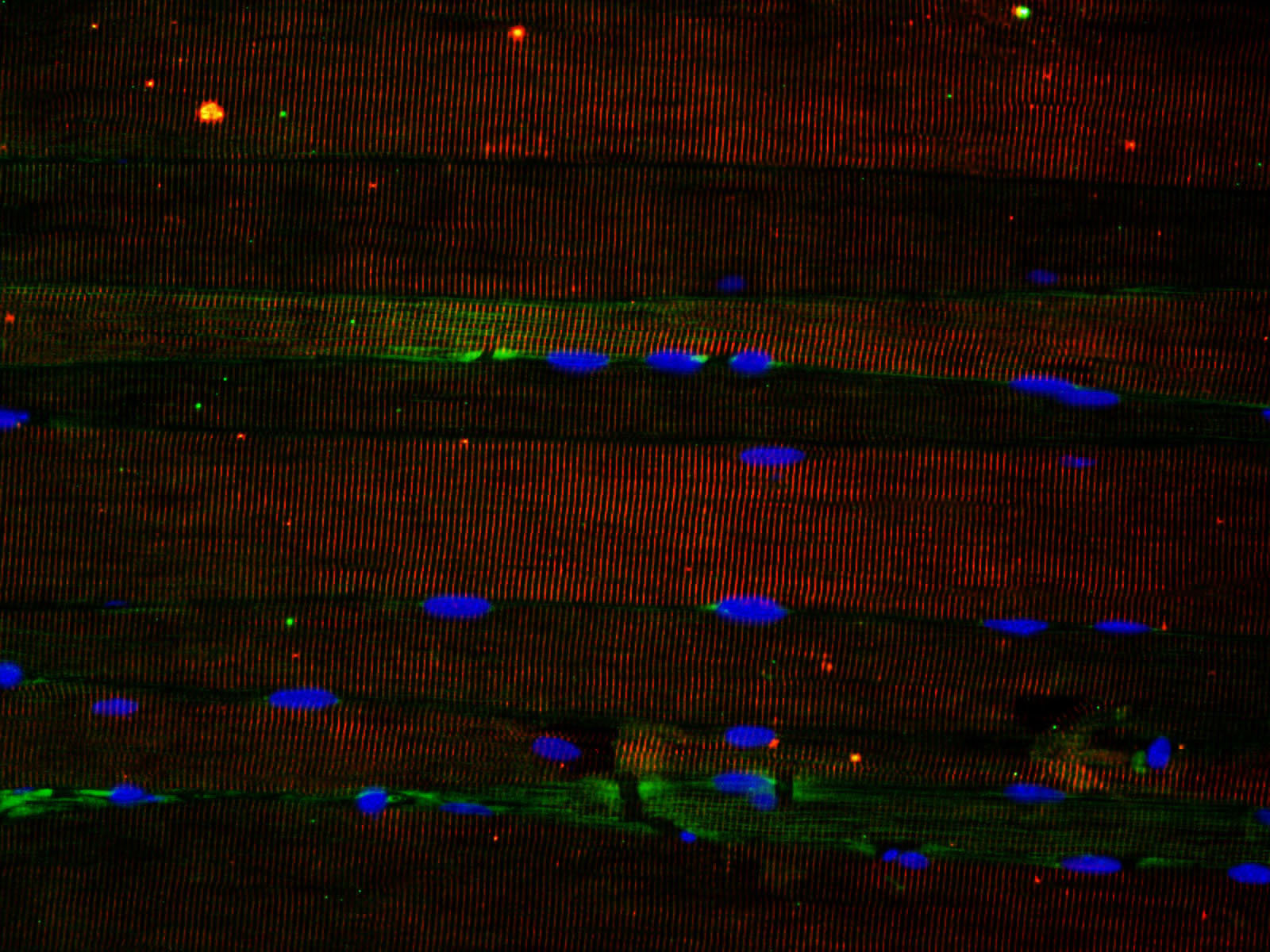

Supplement: Supplementary file 3 [file Data_Sheet_3.ZIP › microscopy images/IF/PKCα(red light)+Nox2(green light)/LVC(4).jpg]

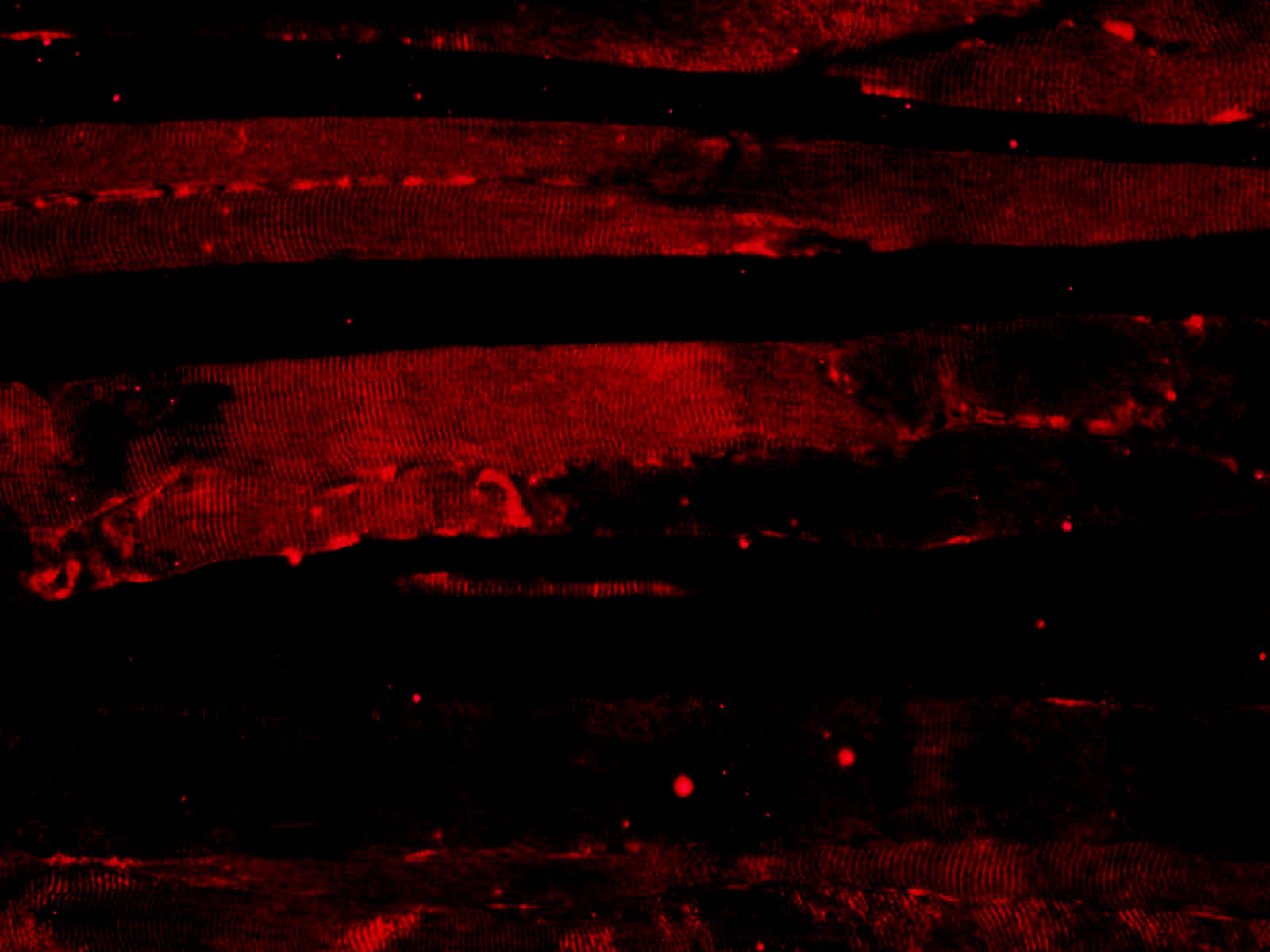

Supplement: Supplementary file 3 [file Data_Sheet_3.ZIP › microscopy images/IF/PKCα(red light)+Nox2(green light)/M (1).jpg]

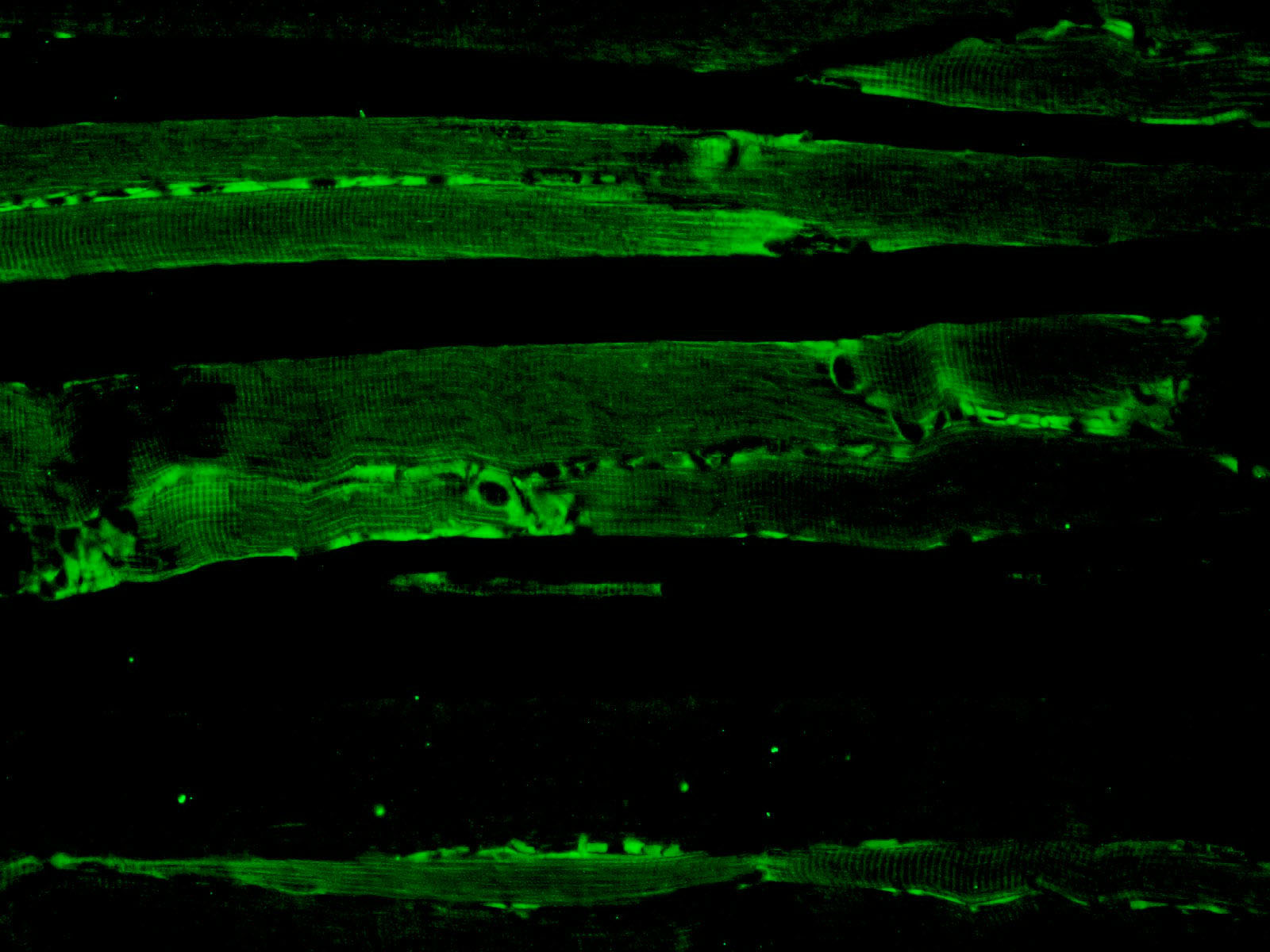

Supplement: Supplementary file 3 [file Data_Sheet_3.ZIP › microscopy images/IF/PKCα(red light)+Nox2(green light)/M (2).jpg]

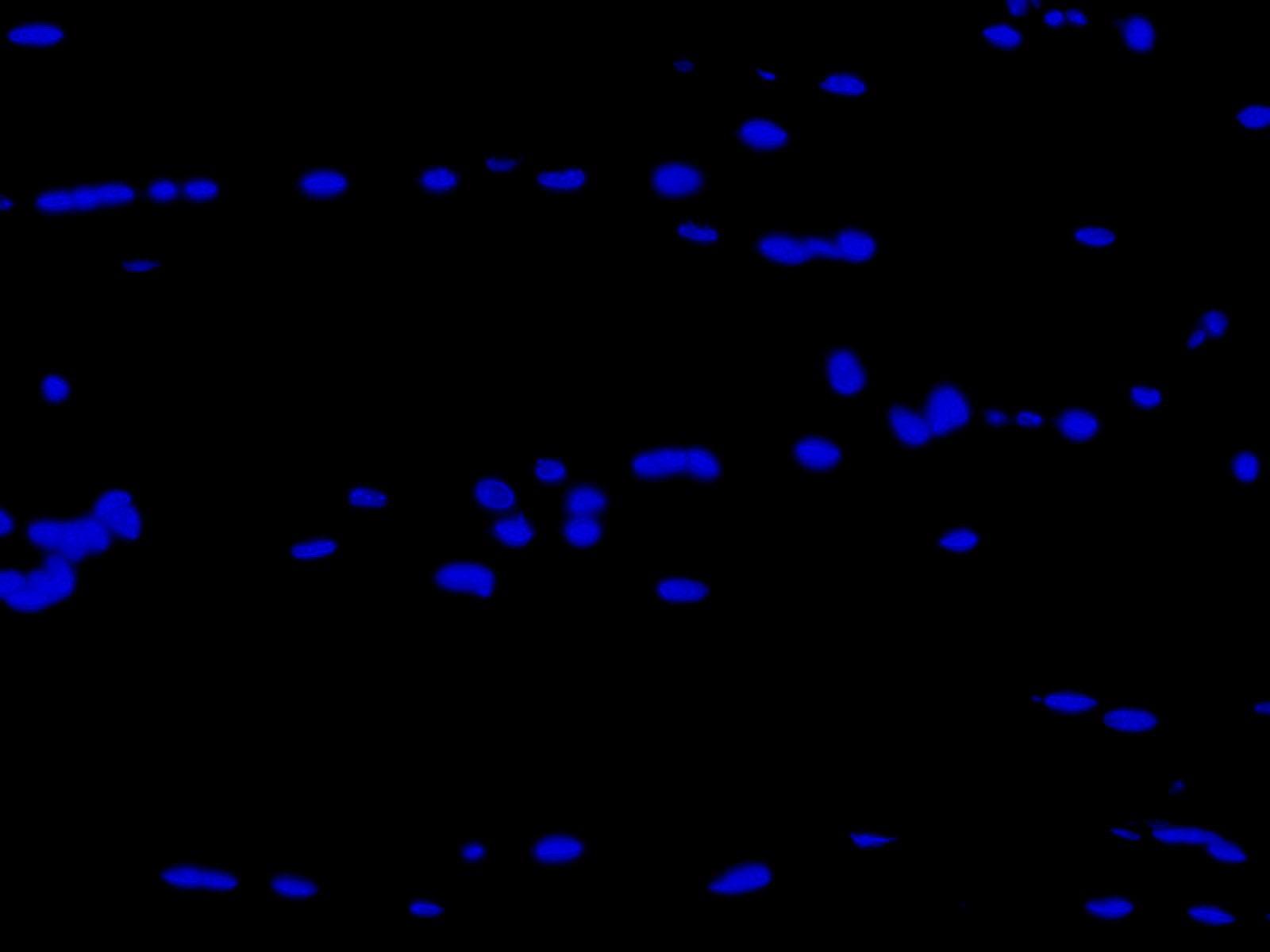

Supplement: Supplementary file 3 [file Data_Sheet_3.ZIP › microscopy images/IF/PKCα(red light)+Nox2(green light)/M (3).jpg]

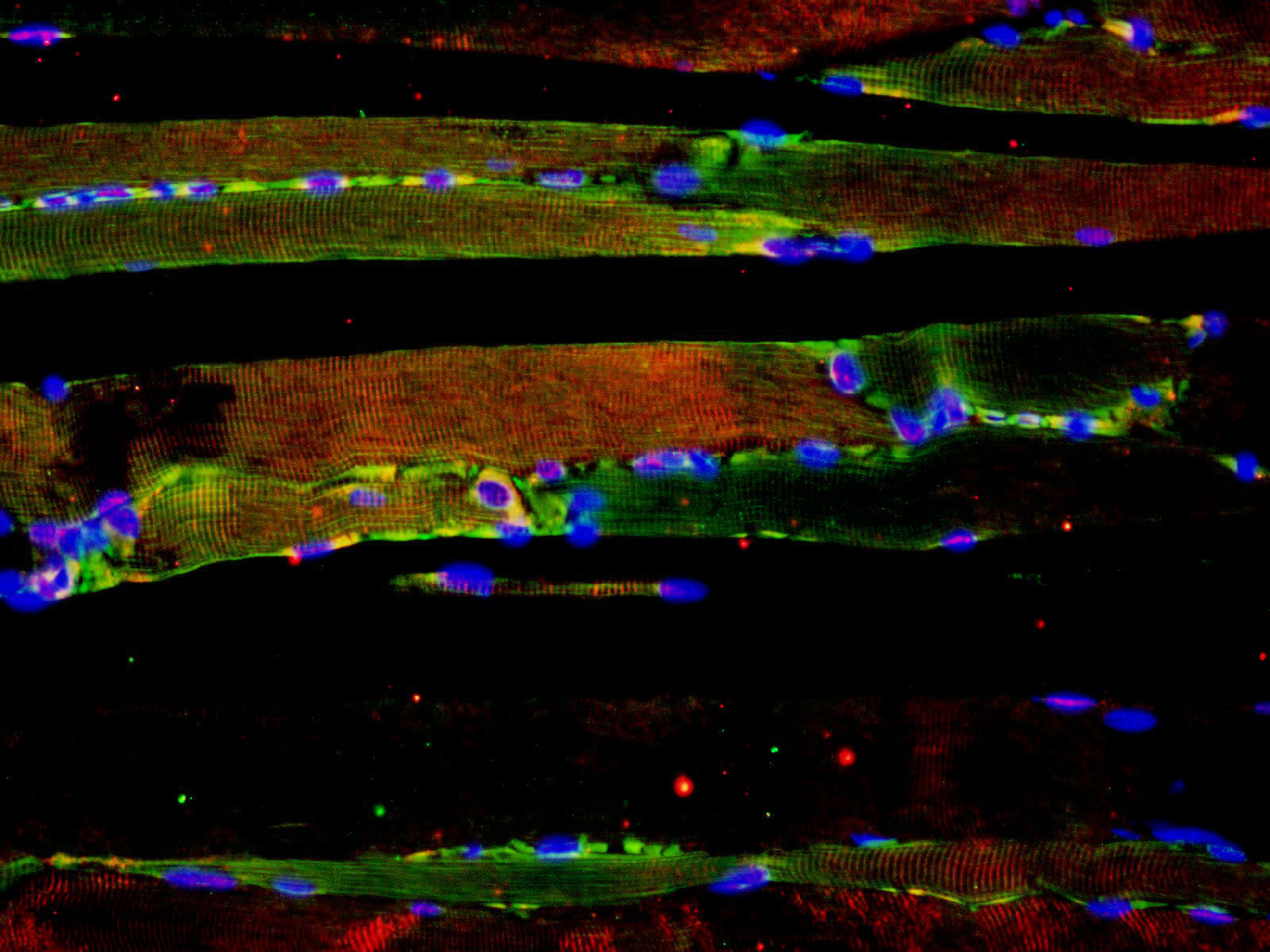

Supplement: Supplementary file 3 [file Data_Sheet_3.ZIP › microscopy images/IF/PKCα(red light)+Nox2(green light)/M (4).jpg]

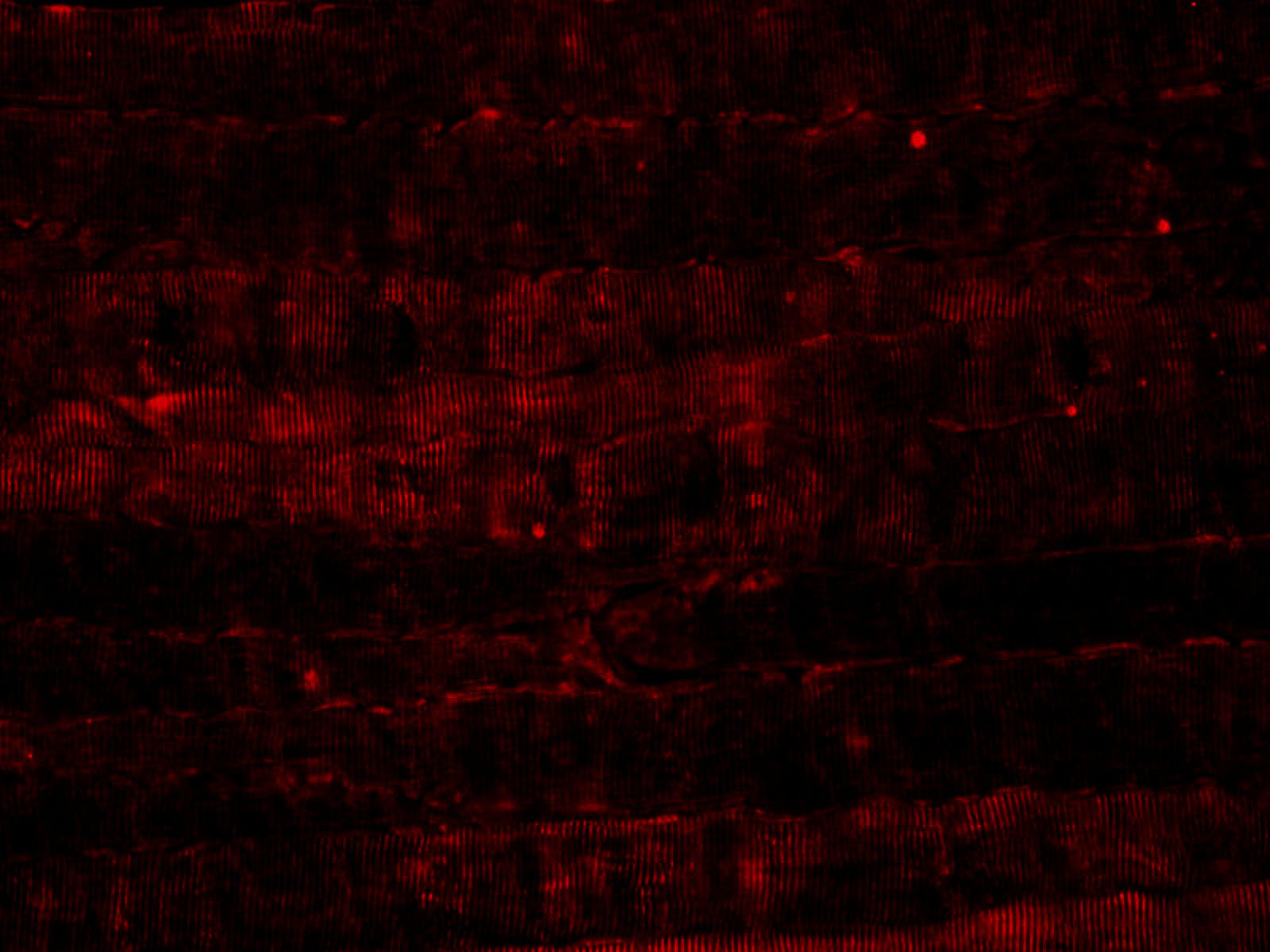

Supplement: Supplementary file 3 [file Data_Sheet_3.ZIP › microscopy images/IF/PKCα(red light)+Nox2(green light)/VC (1).jpg]

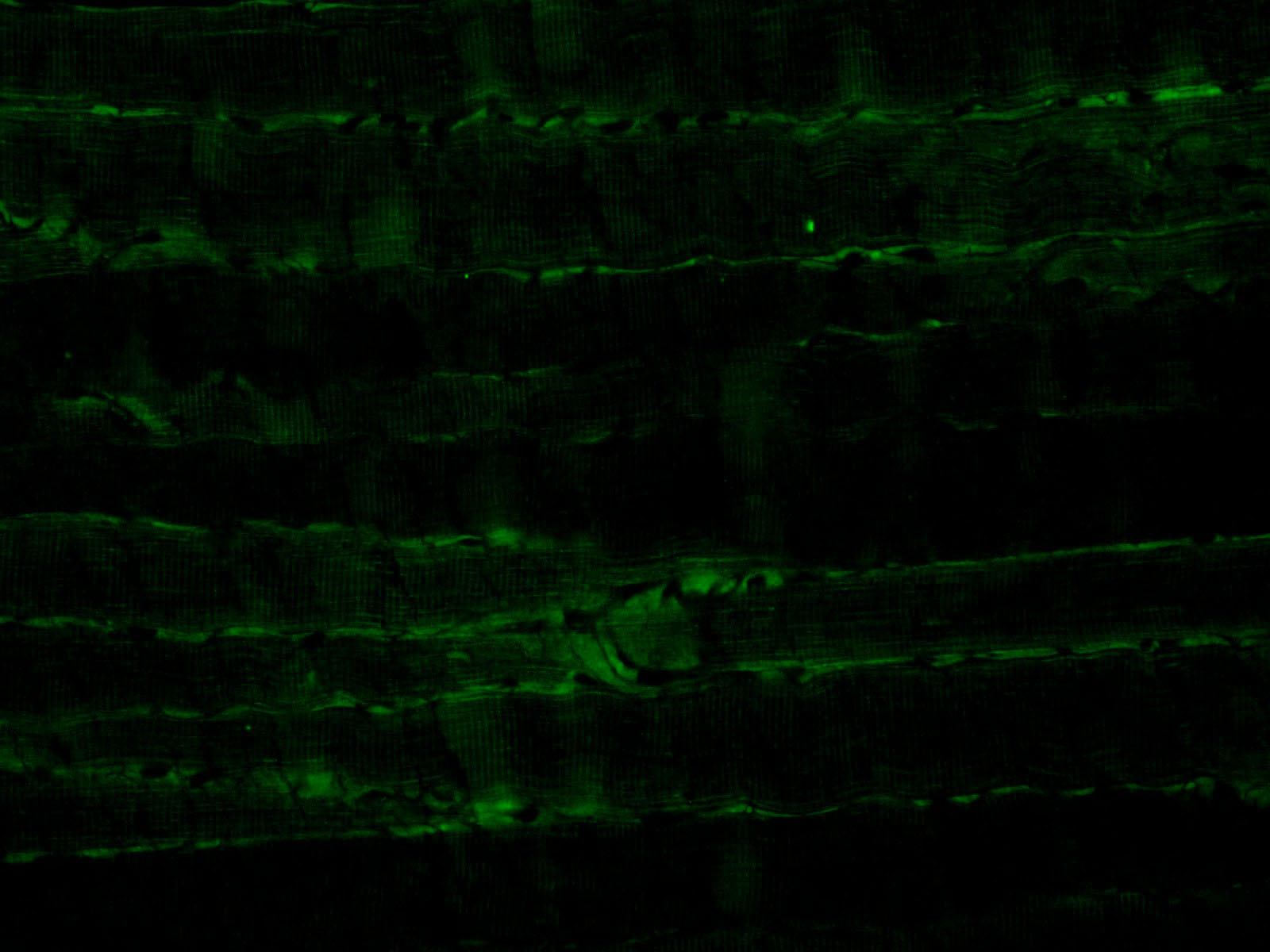

Supplement: Supplementary file 3 [file Data_Sheet_3.ZIP › microscopy images/IF/PKCα(red light)+Nox2(green light)/VC (2).jpg]

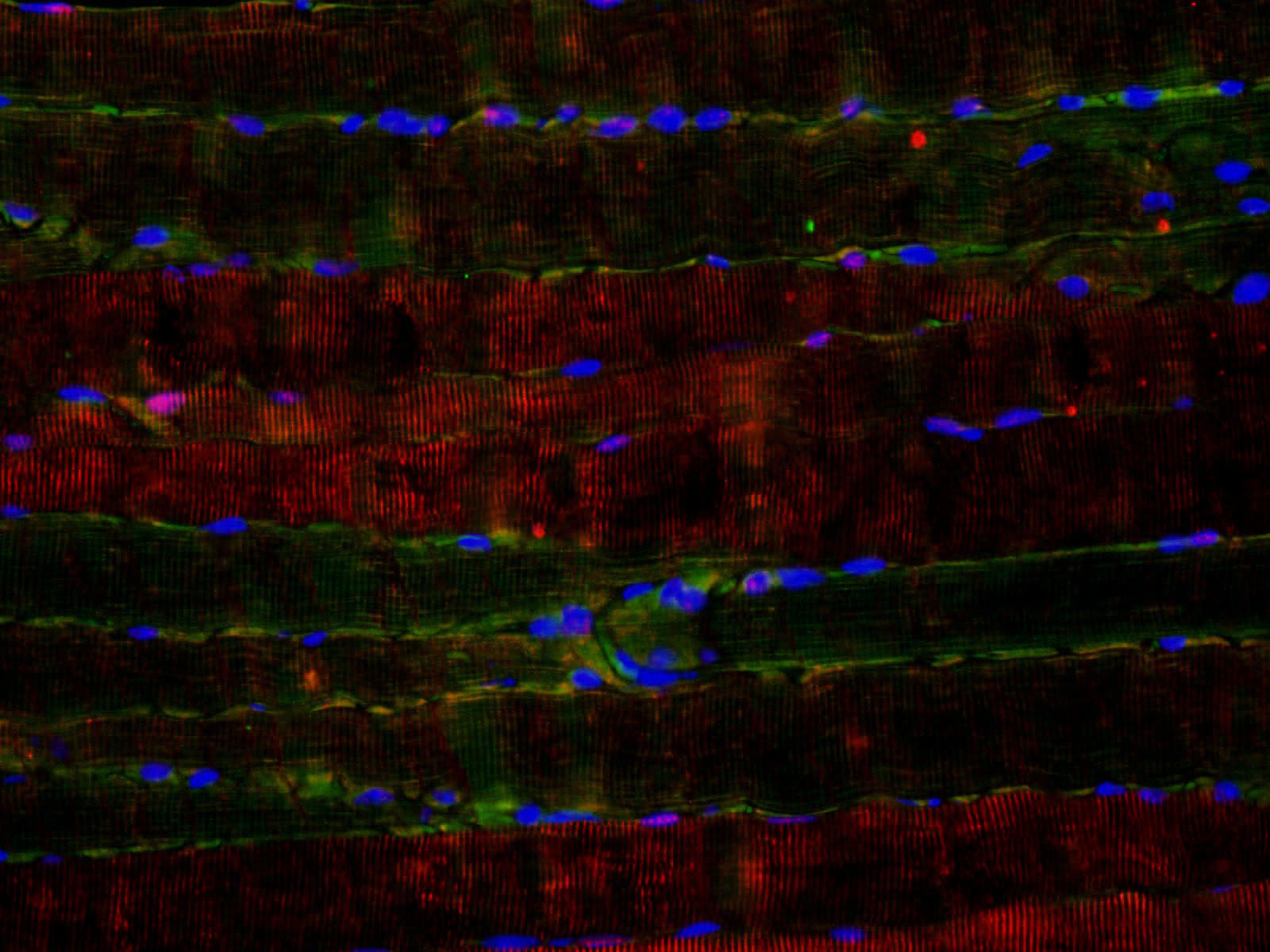

Supplement: Supplementary file 3 [file Data_Sheet_3.ZIP › microscopy images/IF/PKCα(red light)+Nox2(green light)/vc (4).jpg]

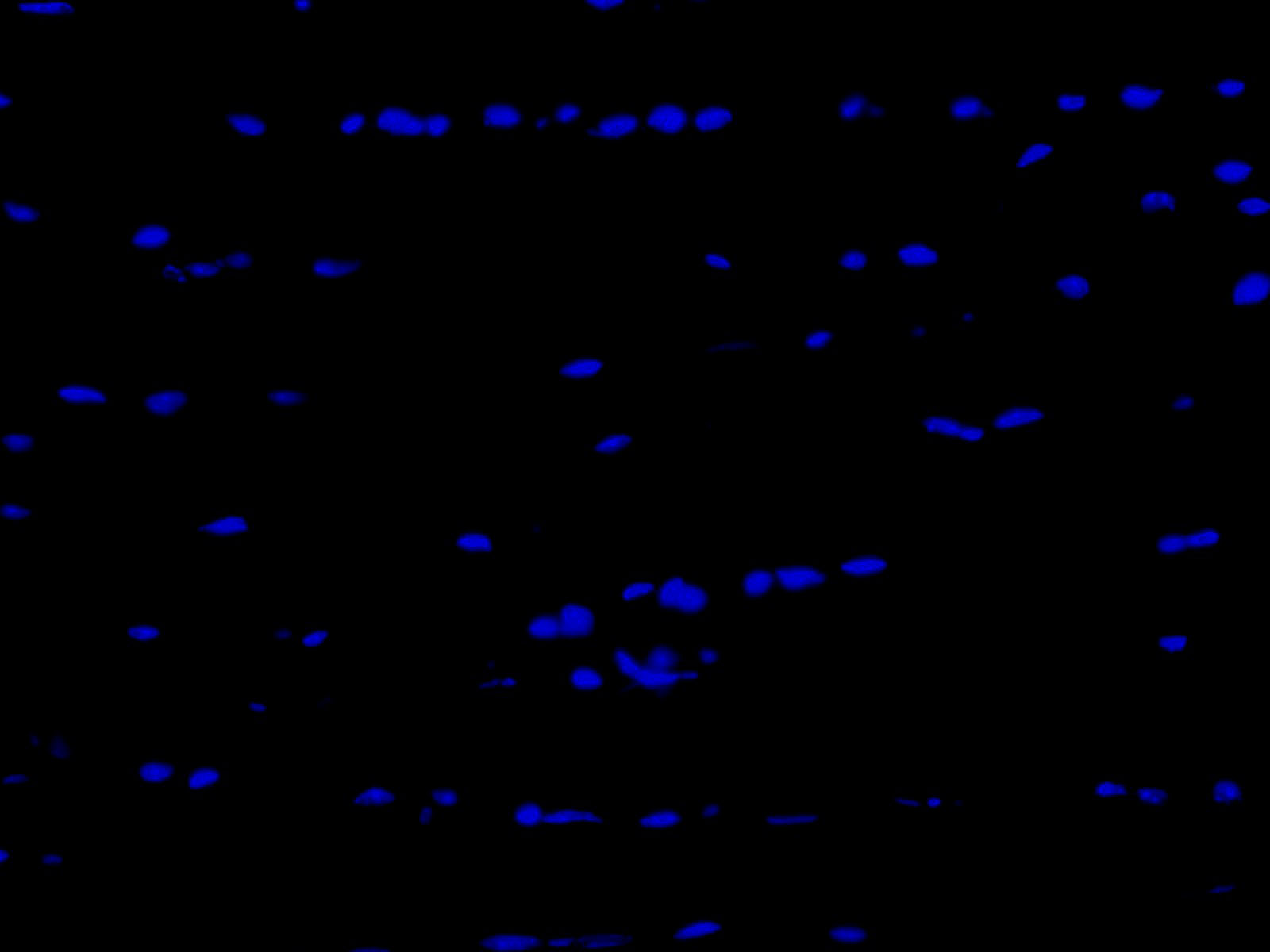

Supplement: Supplementary file 3 [file Data_Sheet_3.ZIP › microscopy images/IF/PKCα(red light)+Nox2(green light)/VC(3).jpg]

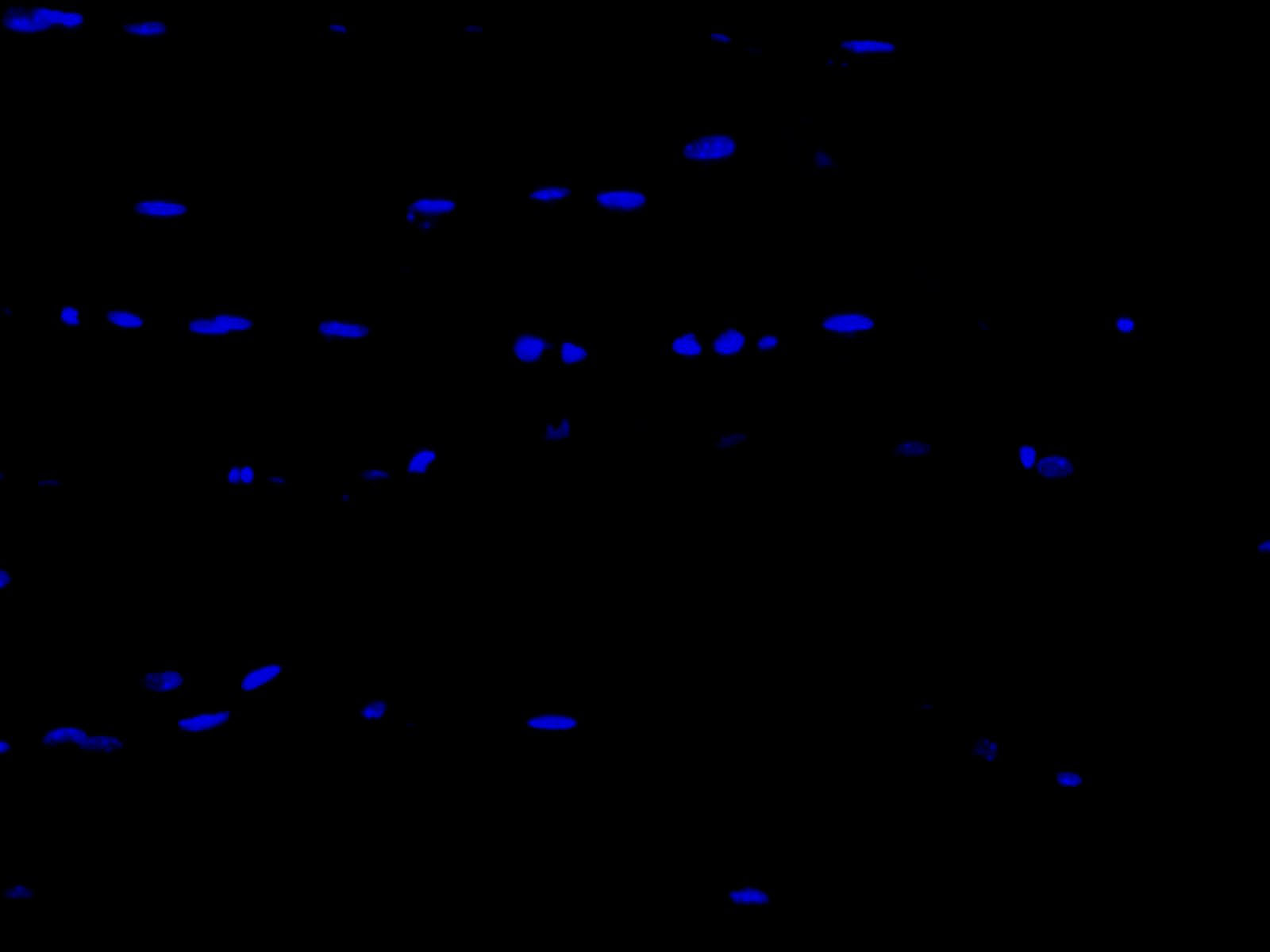

Supplement: Supplementary file 3 [file Data_Sheet_3.ZIP › microscopy images/IF/PKCα(red light)+Nox4(green light)/C (3).jpg]

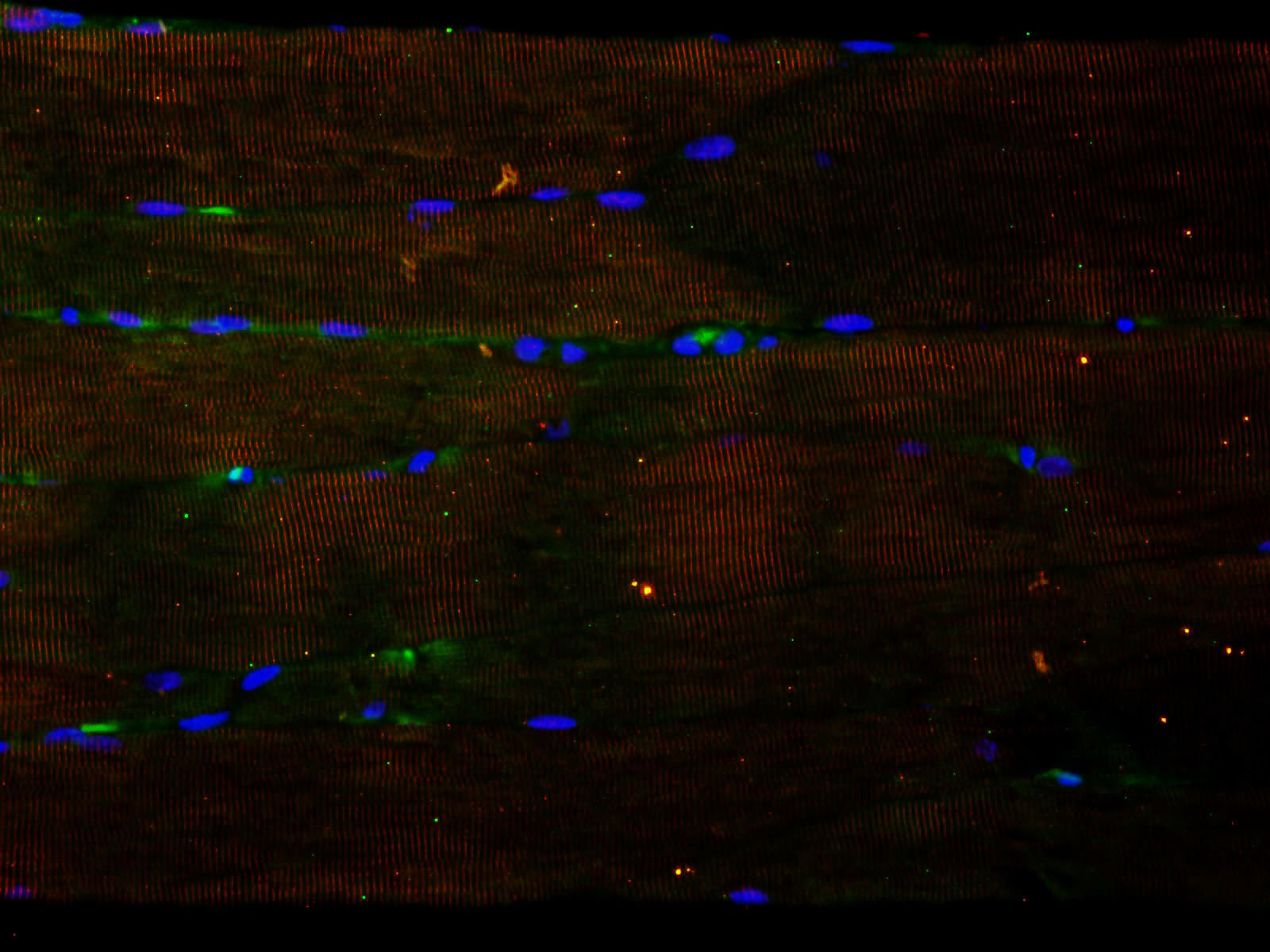

Supplement: Supplementary file 3 [file Data_Sheet_3.ZIP › microscopy images/IF/PKCα(red light)+Nox4(green light)/C (4).jpg]

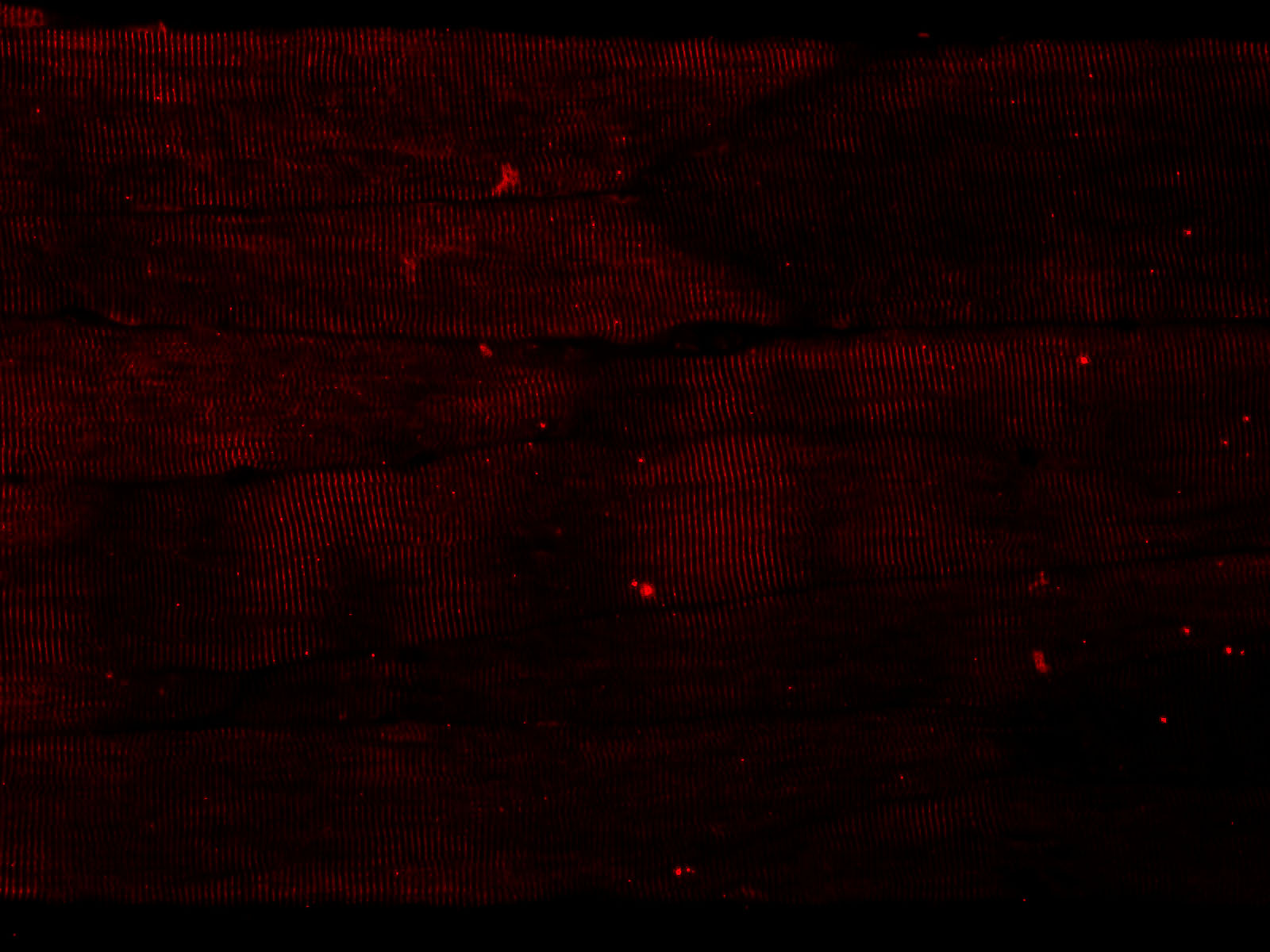

Supplement: Supplementary file 3 [file Data_Sheet_3.ZIP › microscopy images/IF/PKCα(red light)+Nox4(green light)/C(1).jpg]

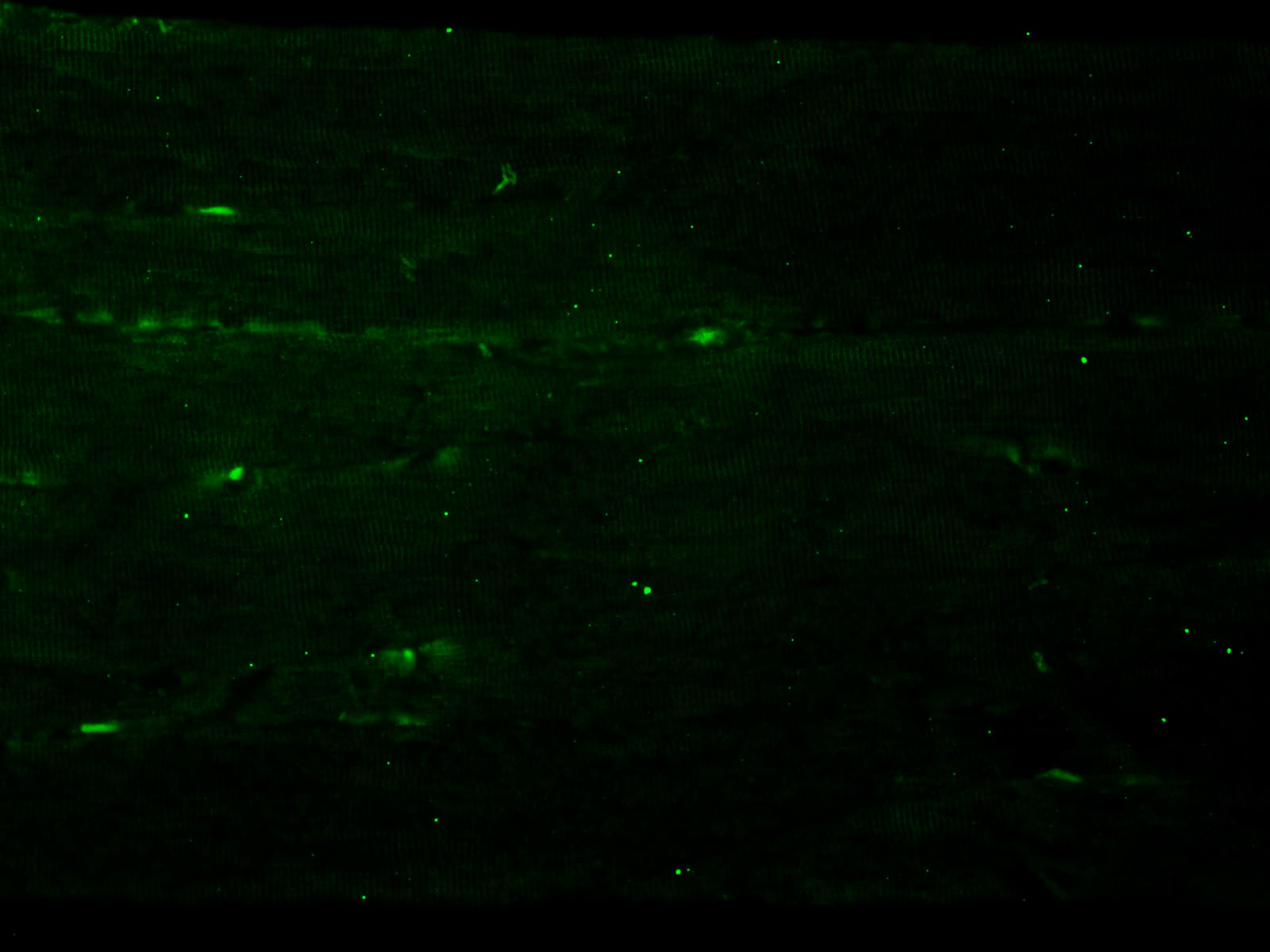

Supplement: Supplementary file 3 [file Data_Sheet_3.ZIP › microscopy images/IF/PKCα(red light)+Nox4(green light)/C(2).jpg]

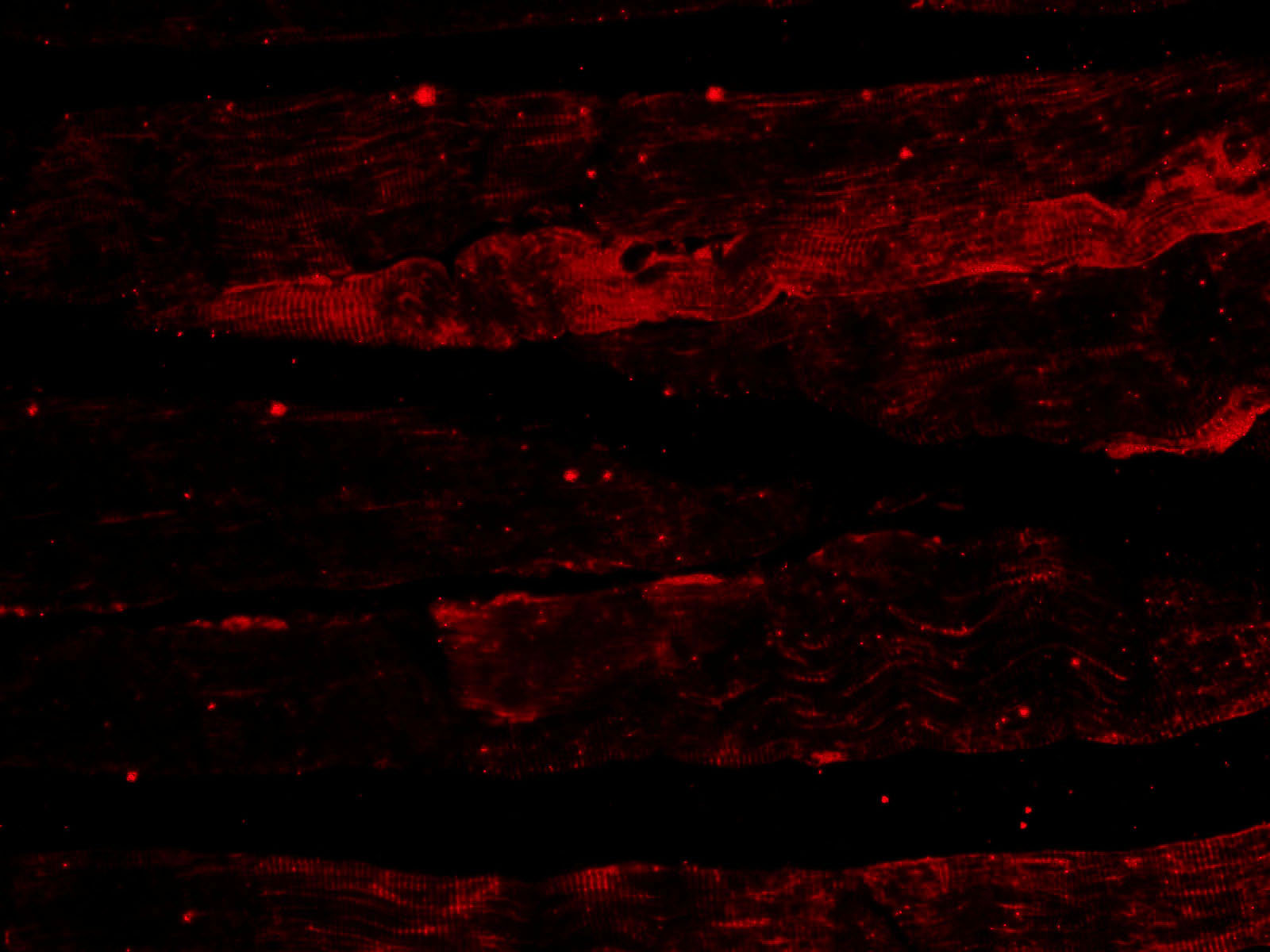

Supplement: Supplementary file 3 [file Data_Sheet_3.ZIP › microscopy images/IF/PKCα(red light)+Nox4(green light)/LCBP (1).jpg]

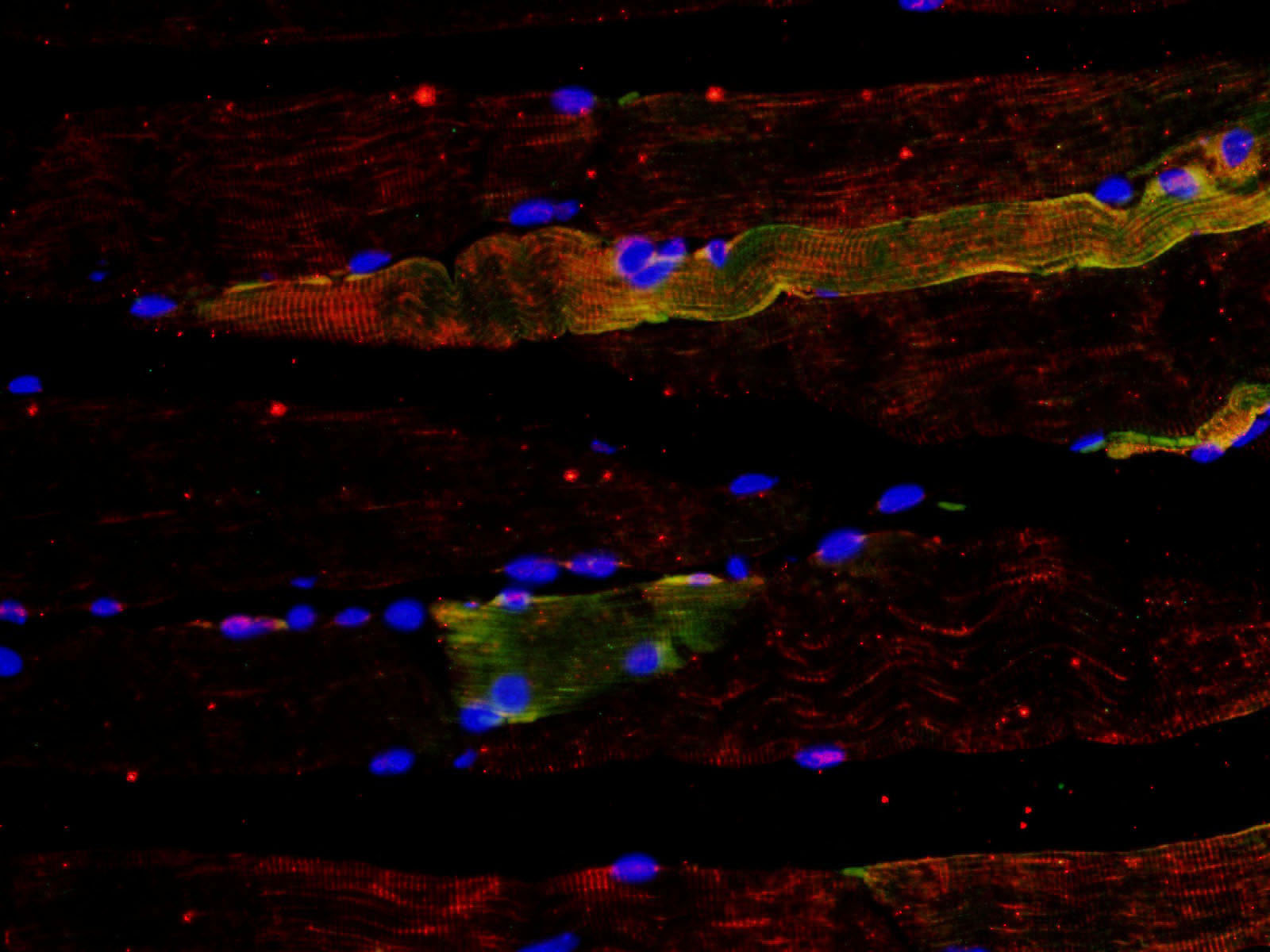

Supplement: Supplementary file 3 [file Data_Sheet_3.ZIP › microscopy images/IF/PKCα(red light)+Nox4(green light)/LCBP (4).jpg]

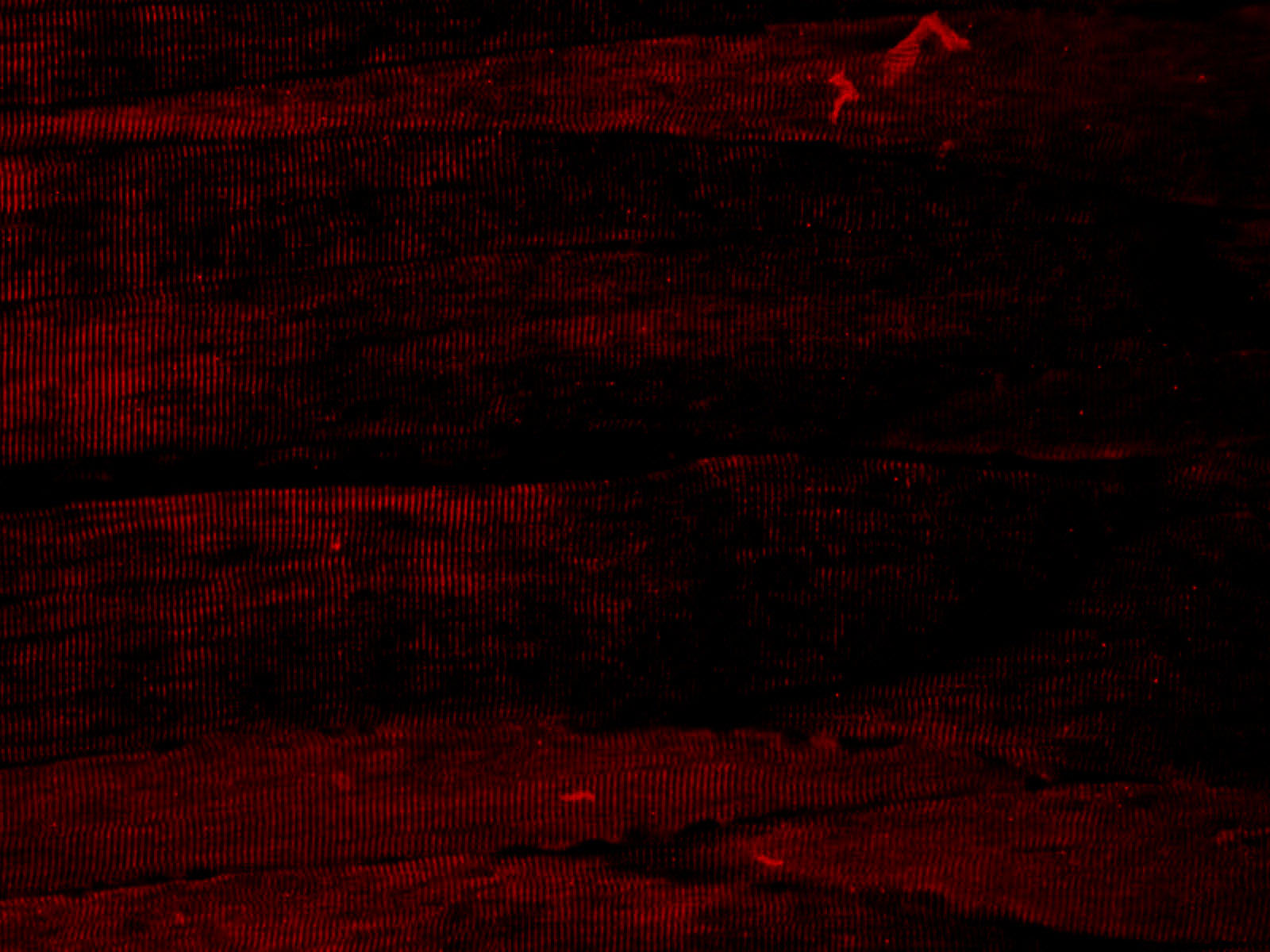

Supplement: Supplementary file 3 [file Data_Sheet_3.ZIP › microscopy images/IF/PKCα(red light)+Nox4(green light)/LLCBP (1).jpg]

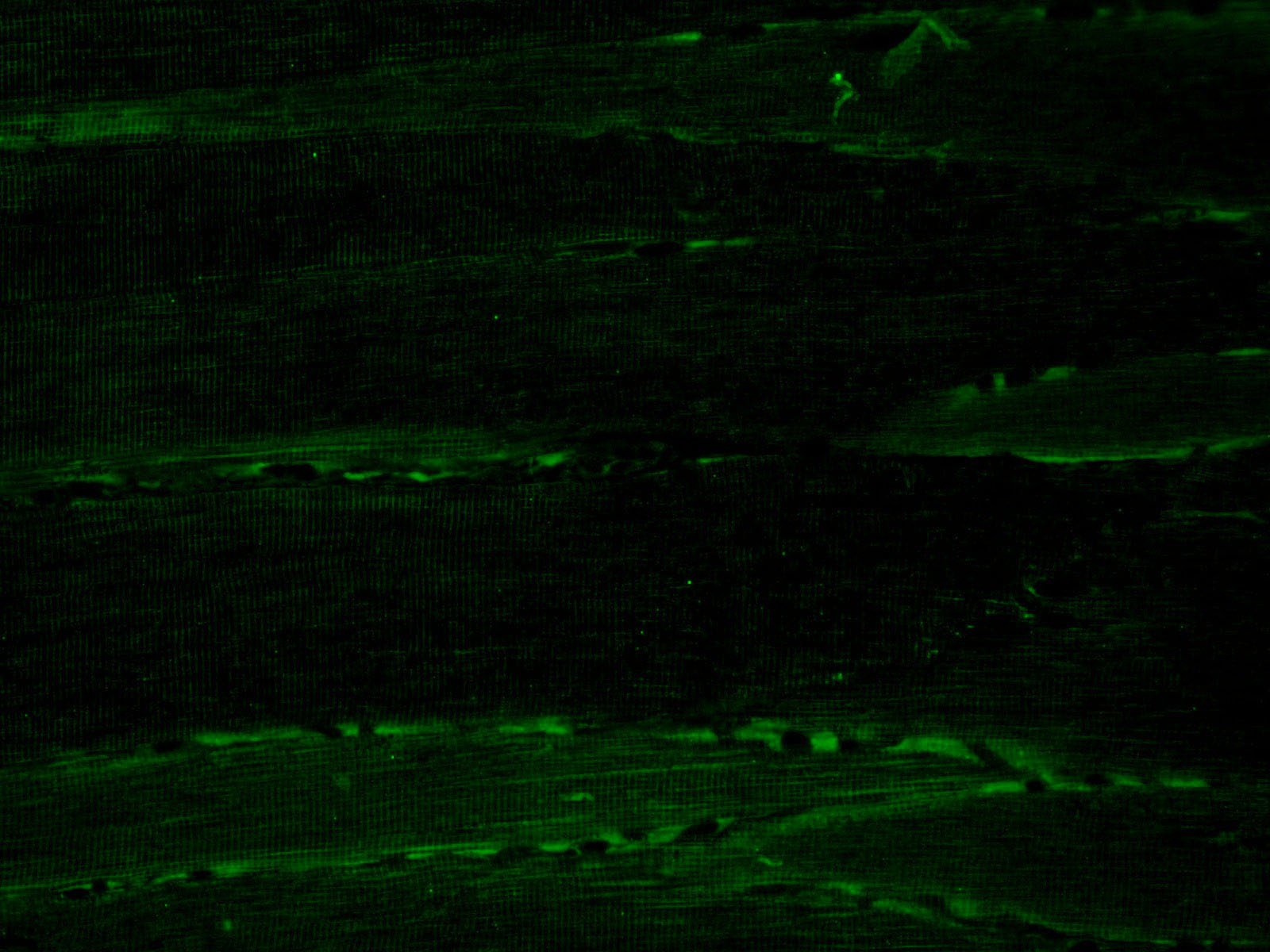

Supplement: Supplementary file 3 [file Data_Sheet_3.ZIP › microscopy images/IF/PKCα(red light)+Nox4(green light)/LLCBP (2).jpg]

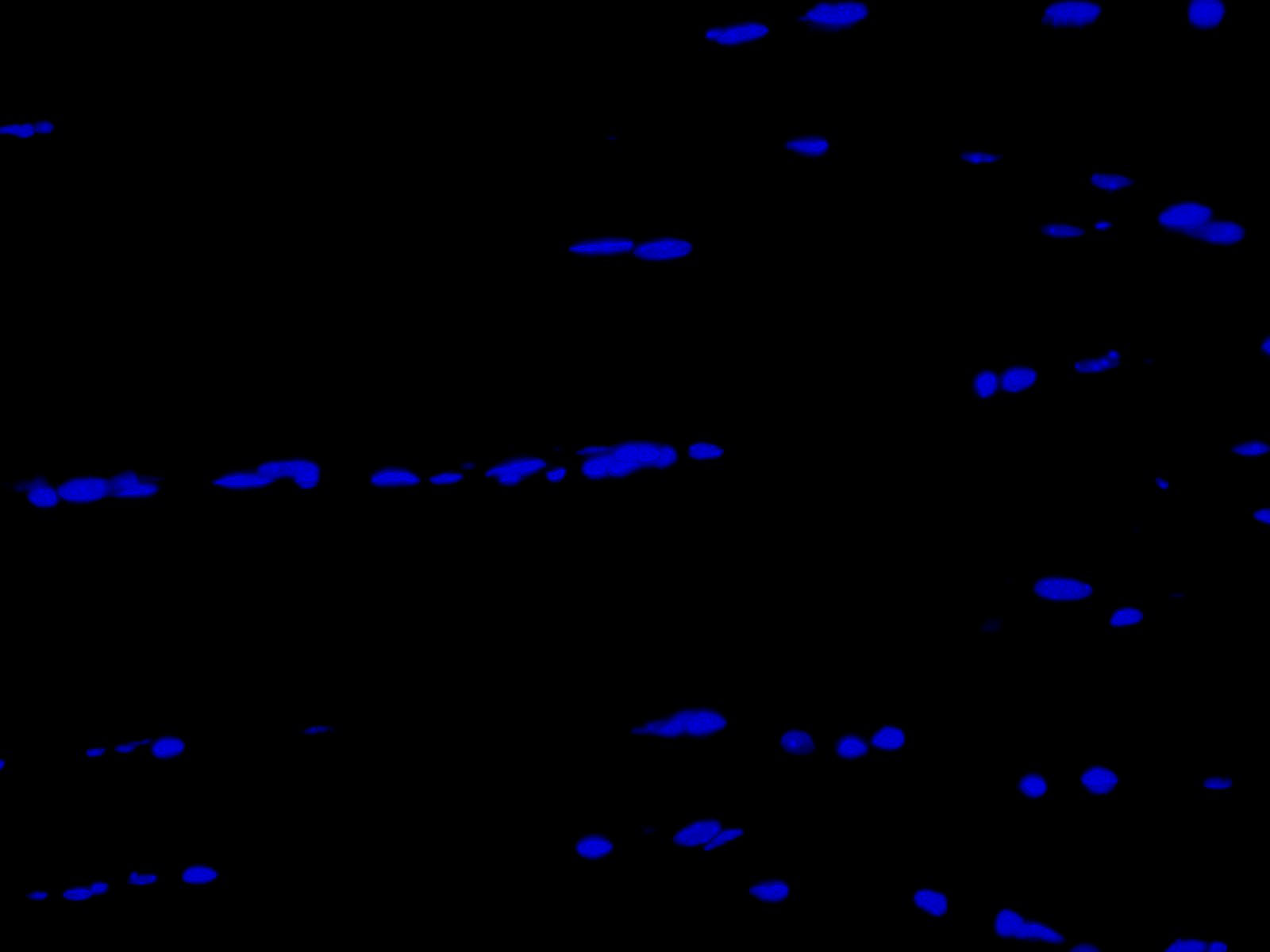

Supplement: Supplementary file 3 [file Data_Sheet_3.ZIP › microscopy images/IF/PKCα(red light)+Nox4(green light)/LLCBP (3).jpg]

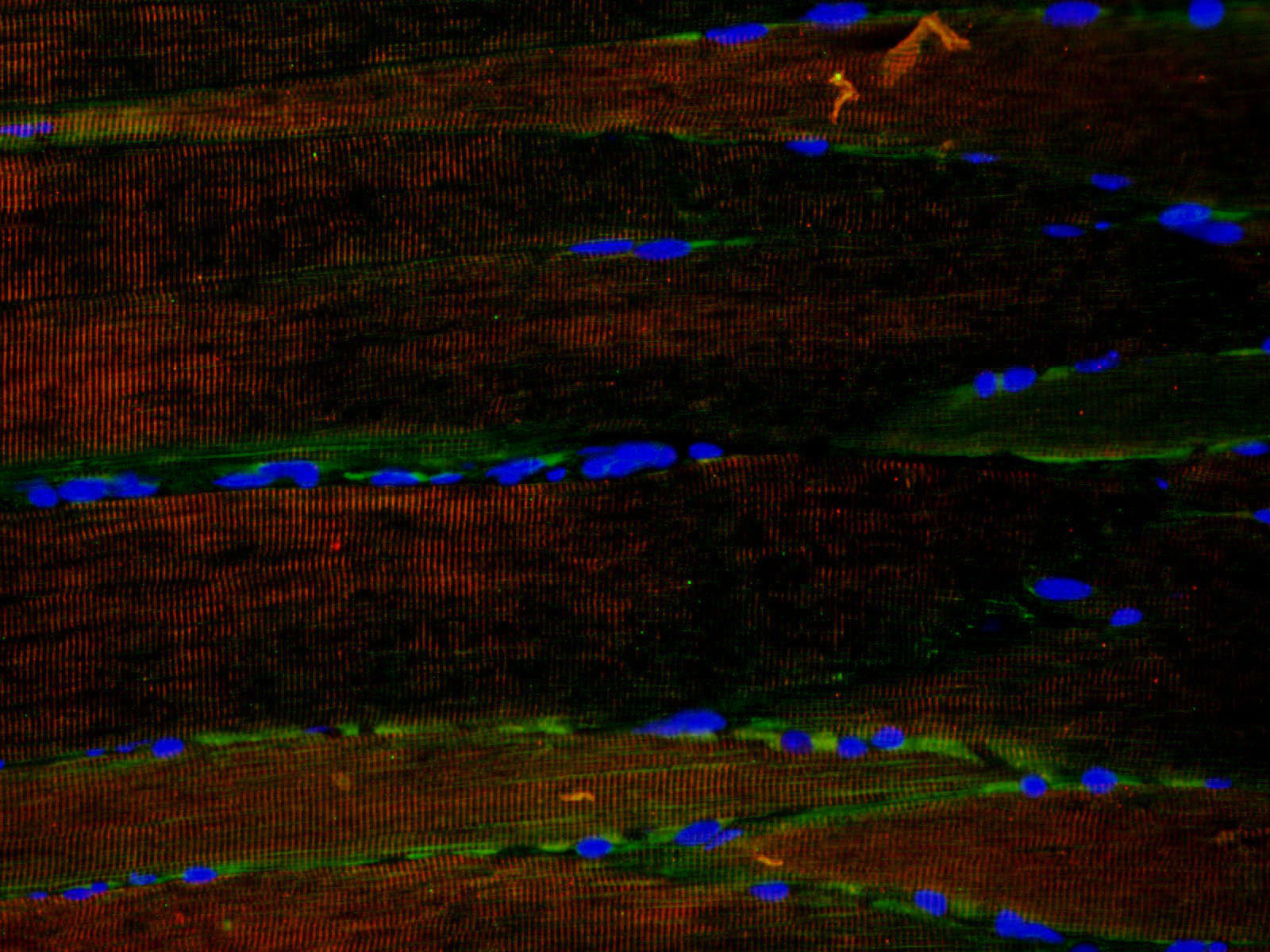

Supplement: Supplementary file 3 [file Data_Sheet_3.ZIP › microscopy images/IF/PKCα(red light)+Nox4(green light)/LLCBP (4).jpg]

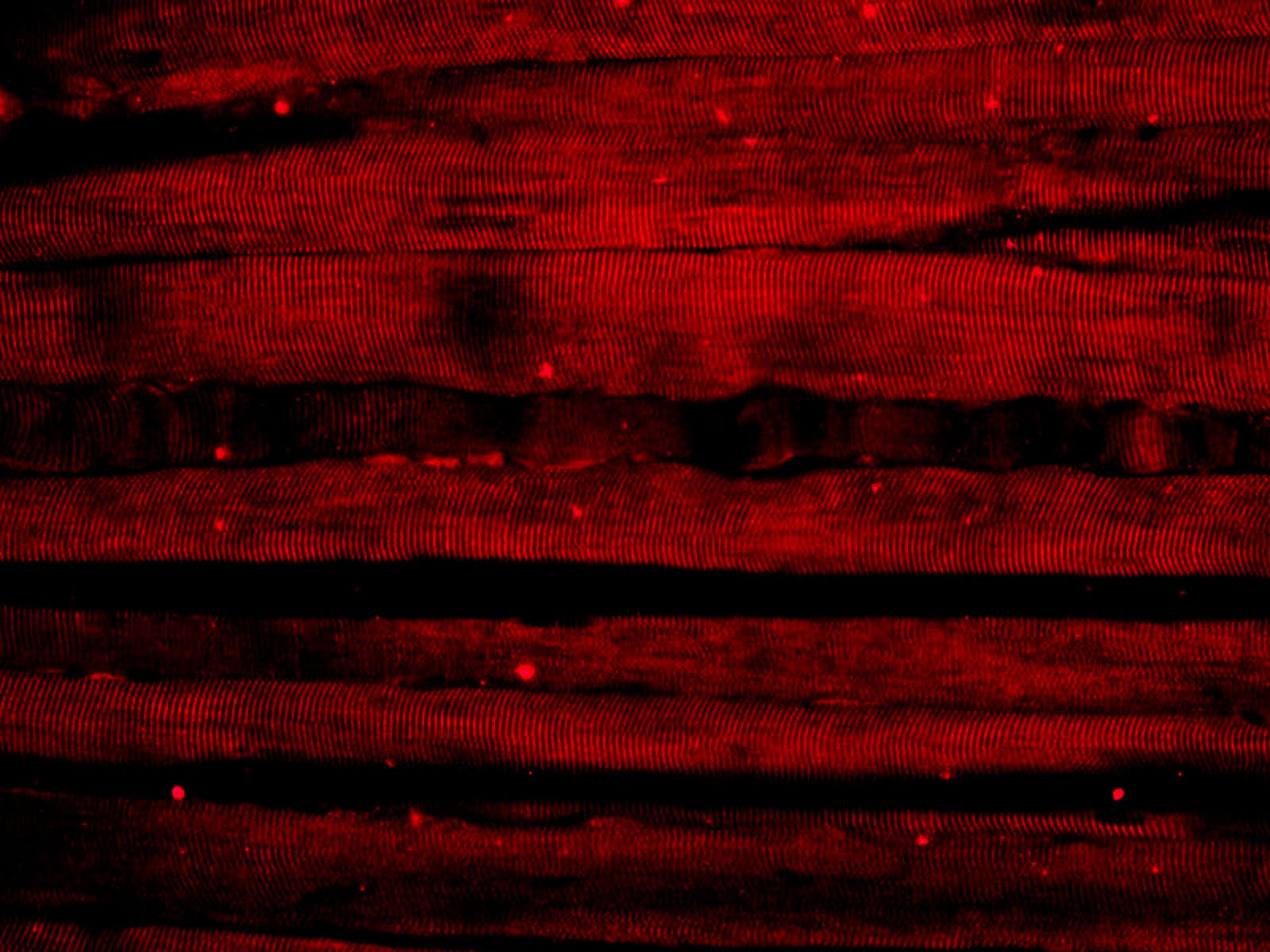

Supplement: Supplementary file 3 [file Data_Sheet_3.ZIP › microscopy images/IF/PKCα(red light)+Nox4(green light)/LM (1).jpg]

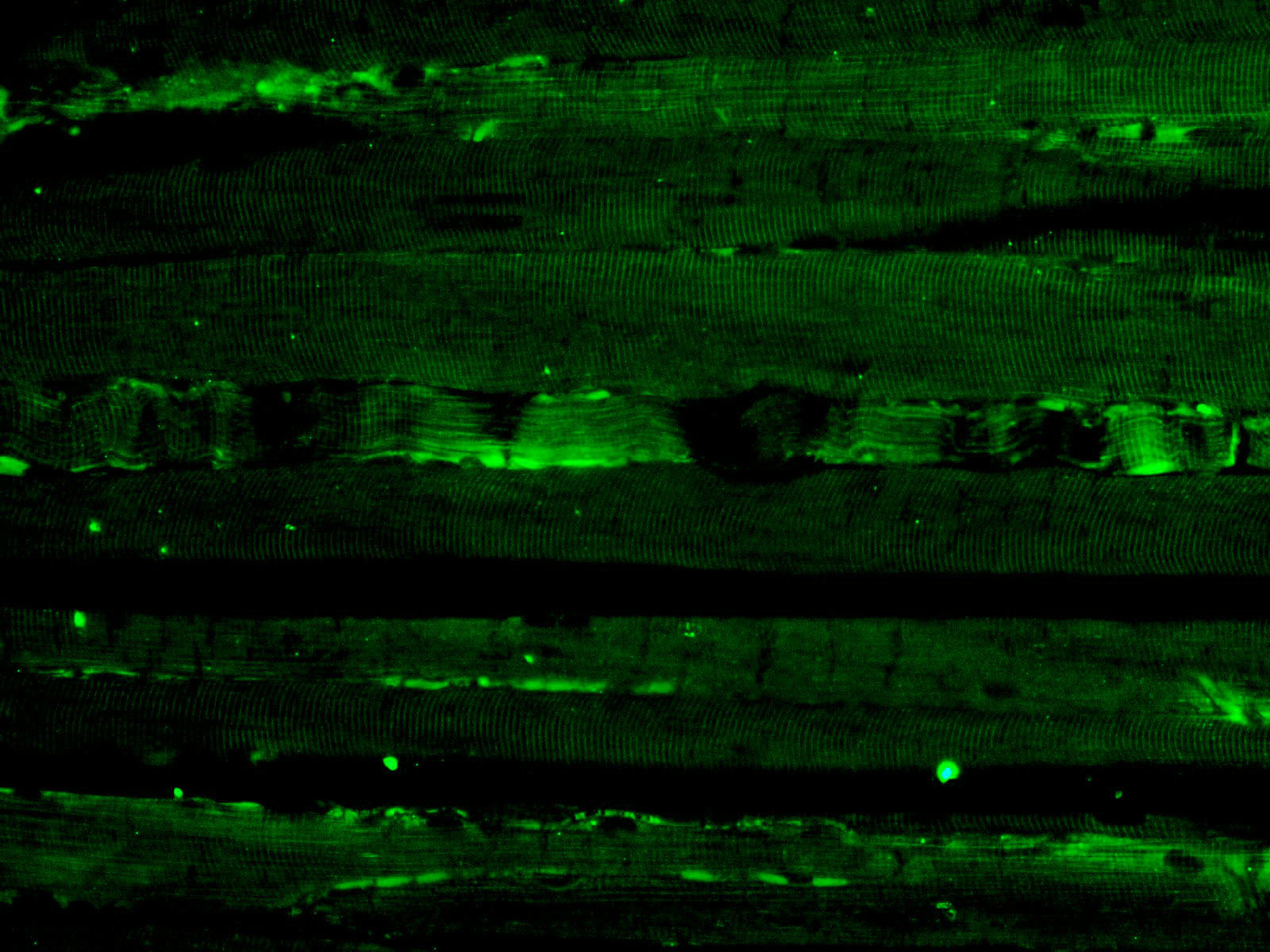

Supplement: Supplementary file 3 [file Data_Sheet_3.ZIP › microscopy images/IF/PKCα(red light)+Nox4(green light)/LM (2).jpg]

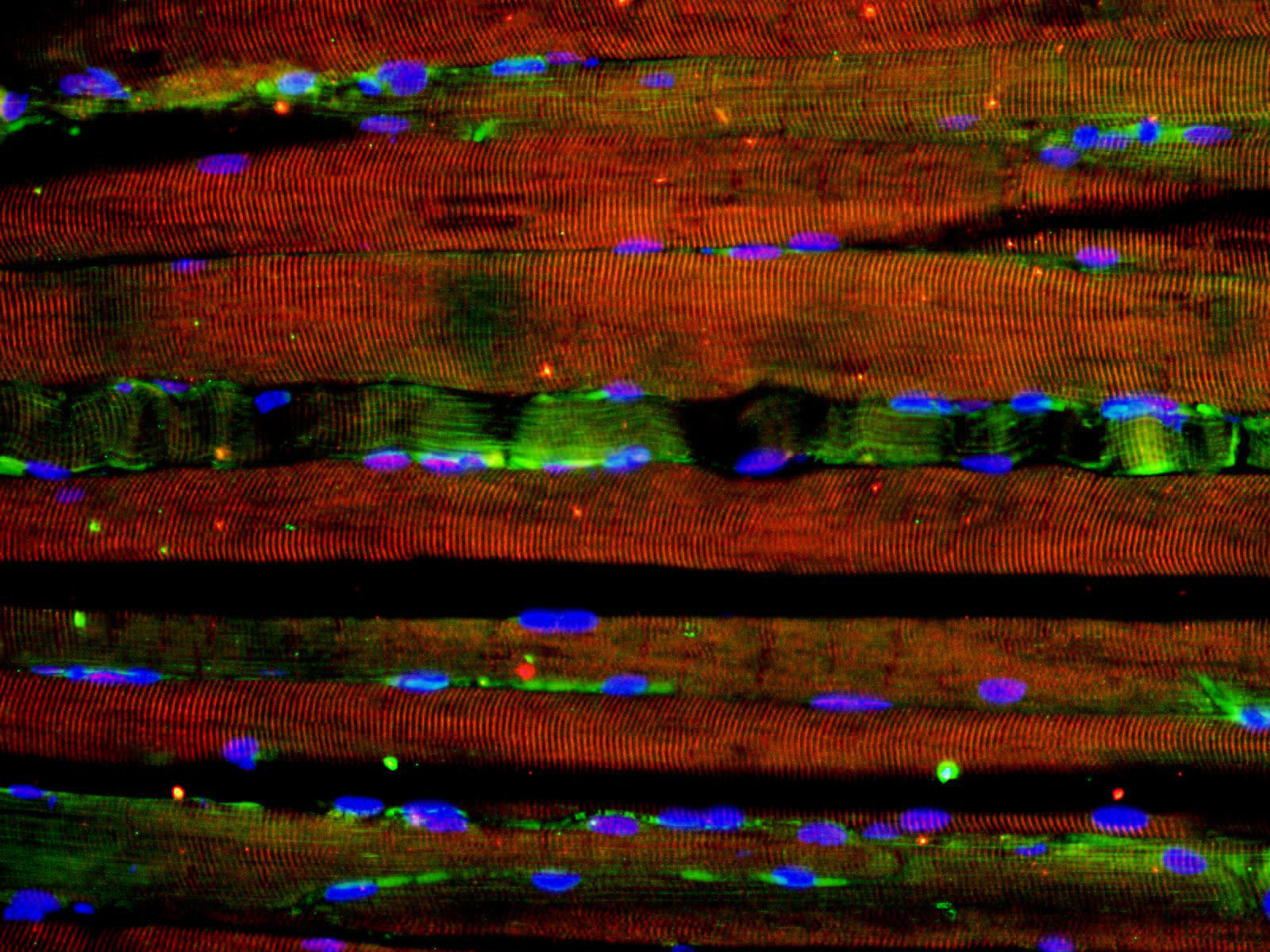

Supplement: Supplementary file 3 [file Data_Sheet_3.ZIP › microscopy images/IF/PKCα(red light)+Nox4(green light)/LM (4).jpg]

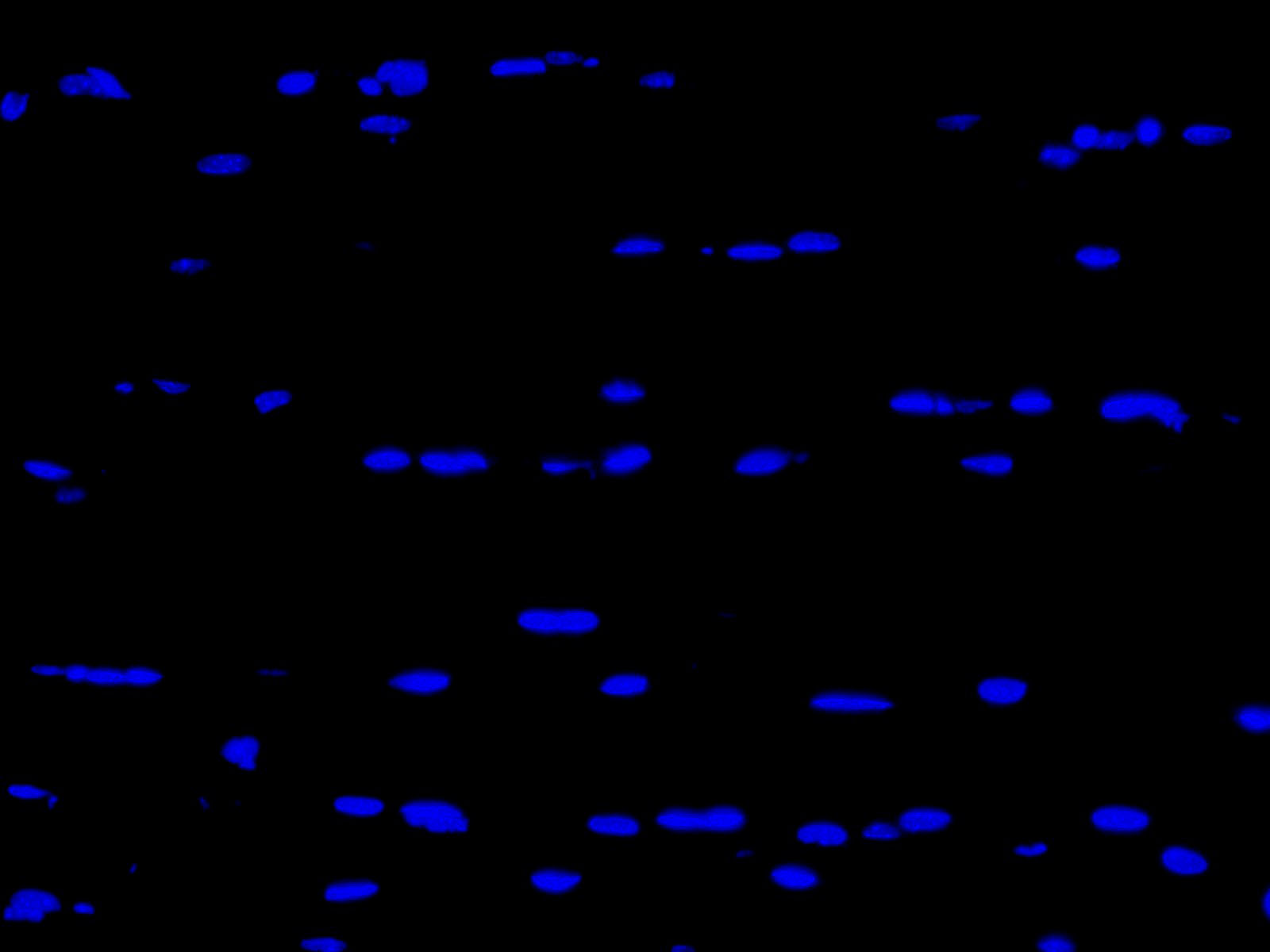

Supplement: Supplementary file 3 [file Data_Sheet_3.ZIP › microscopy images/IF/PKCα(red light)+Nox4(green light)/LM(3).jpg]

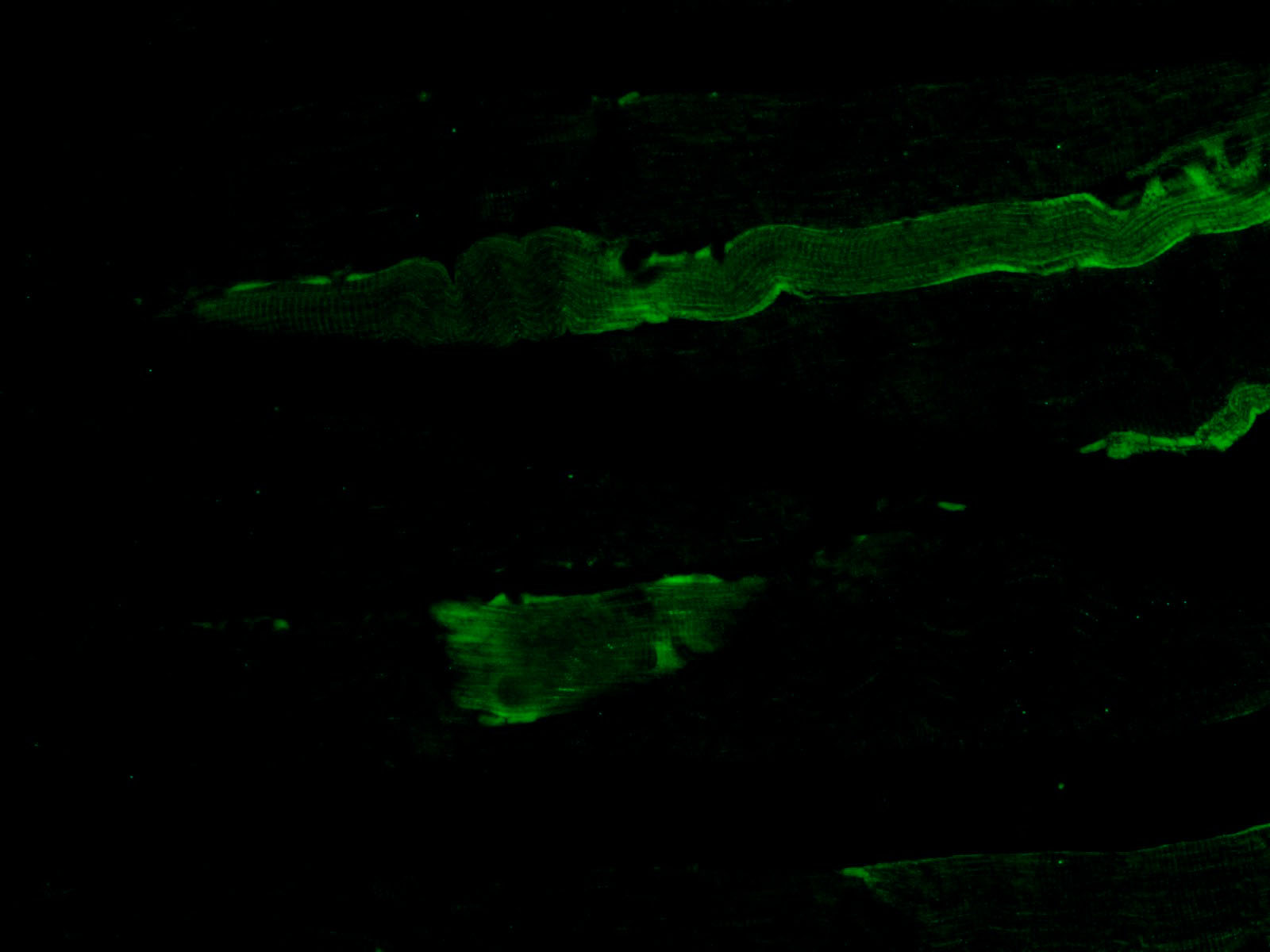

Supplement: Supplementary file 3 [file Data_Sheet_3.ZIP › microscopy images/IF/PKCα(red light)+Nox4(green light)/LVBP (2).jpg]
