# Supplementary figures and images for: Lonicera caerulea Berry Polyphenols Extract Alleviates Exercise Fatigue in Mice by Reducing Oxidative Stress, Inflammation, Skeletal Muscle Cell Apoptosis, and by Increasing Cell Proliferation (part 2 of 2)
Source: Front Nutr. 2022 Mar 9;9:853225. doi: 10.3389/fnut.2022.853225 (PMC8959458; doi:10.3389/fnut.2022.853225)

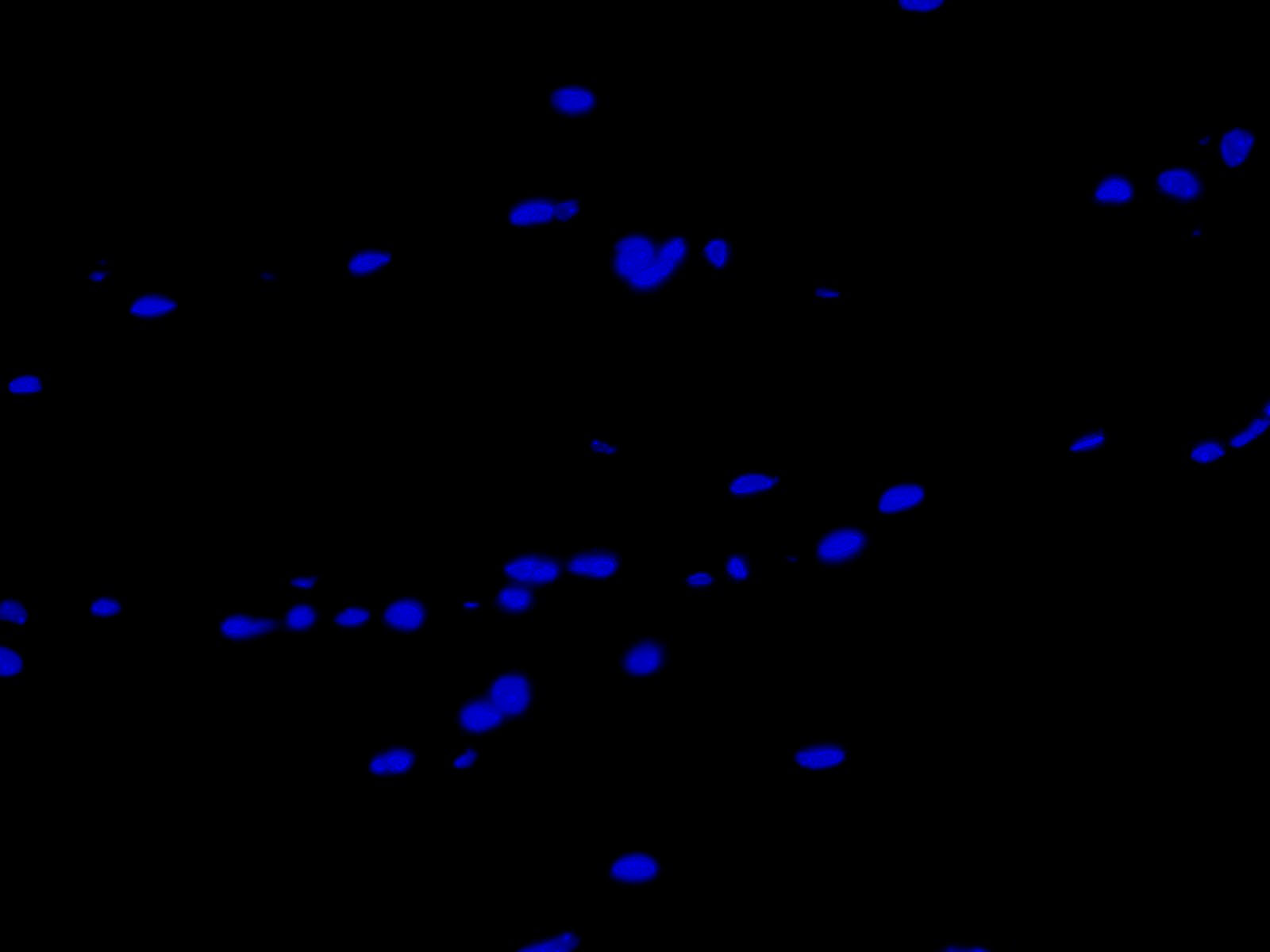

Supplement: Supplementary file 3 [file Data_Sheet_3.ZIP › microscopy images/IF/PKCα(red light)+Nox4(green light)/LVBP(3).jpg]

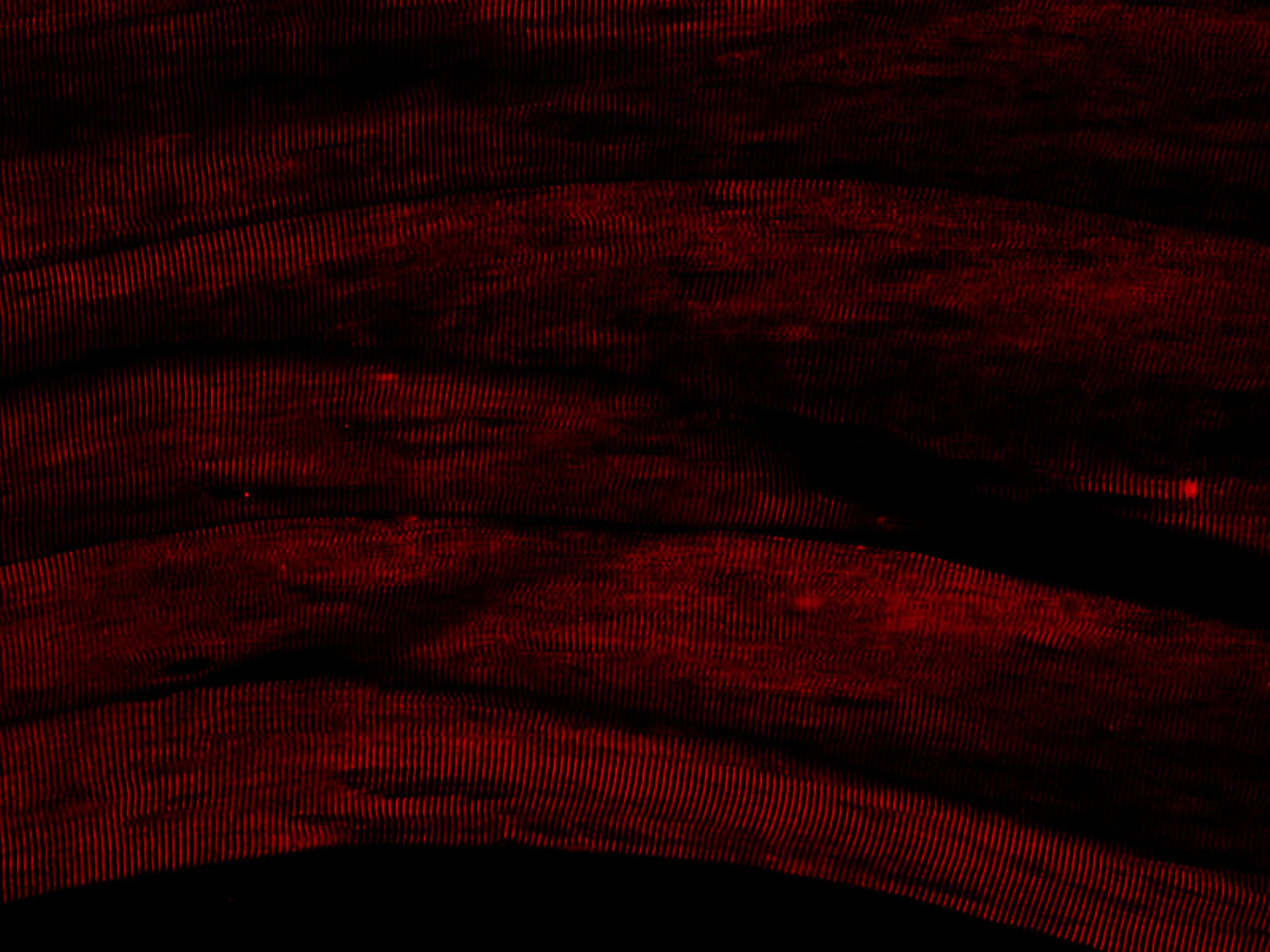

Supplement: Supplementary file 3 [file Data_Sheet_3.ZIP › microscopy images/IF/PKCα(red light)+Nox4(green light)/LVC (1).jpg]

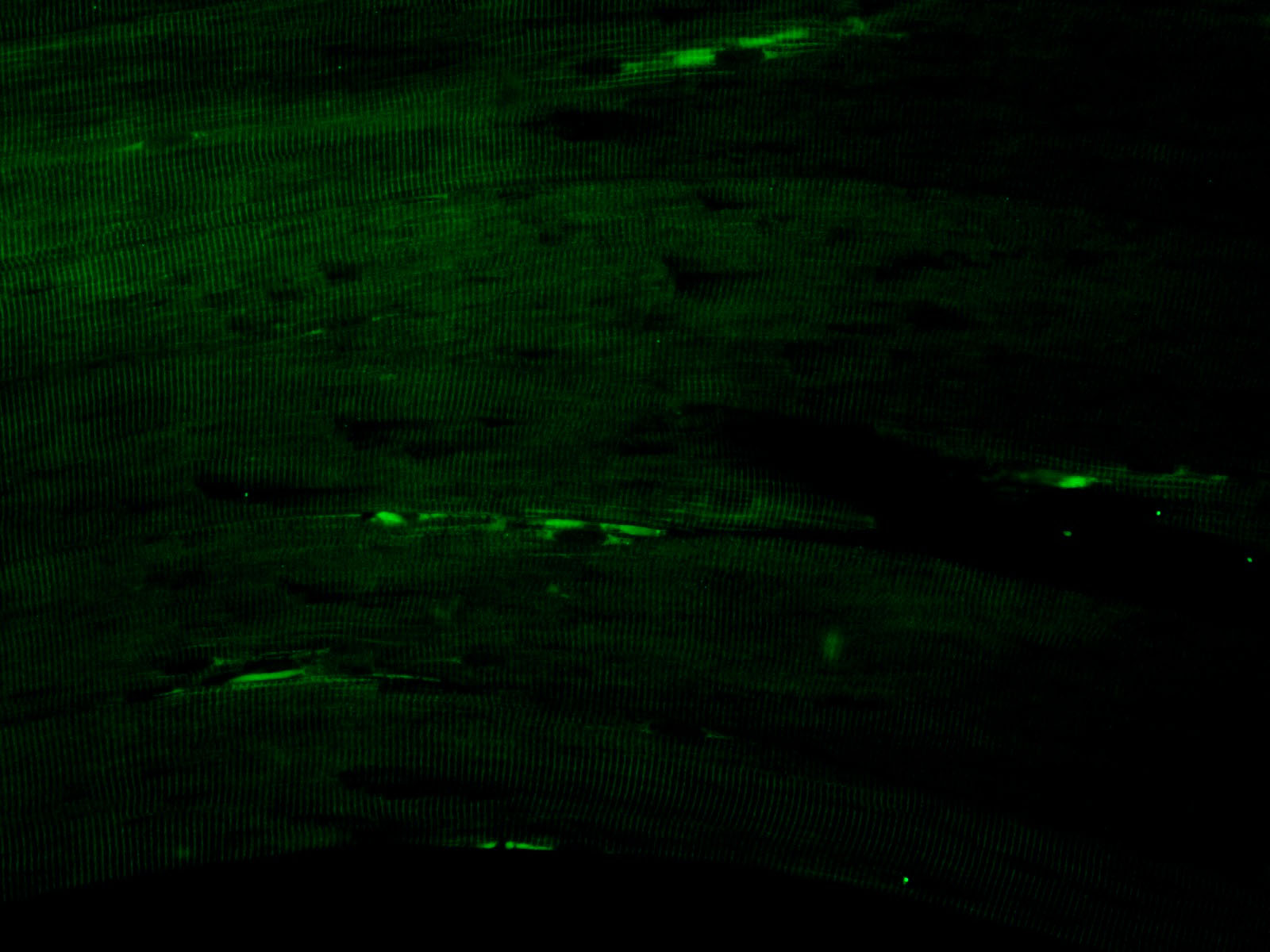

Supplement: Supplementary file 3 [file Data_Sheet_3.ZIP › microscopy images/IF/PKCα(red light)+Nox4(green light)/LVC (2).jpg]

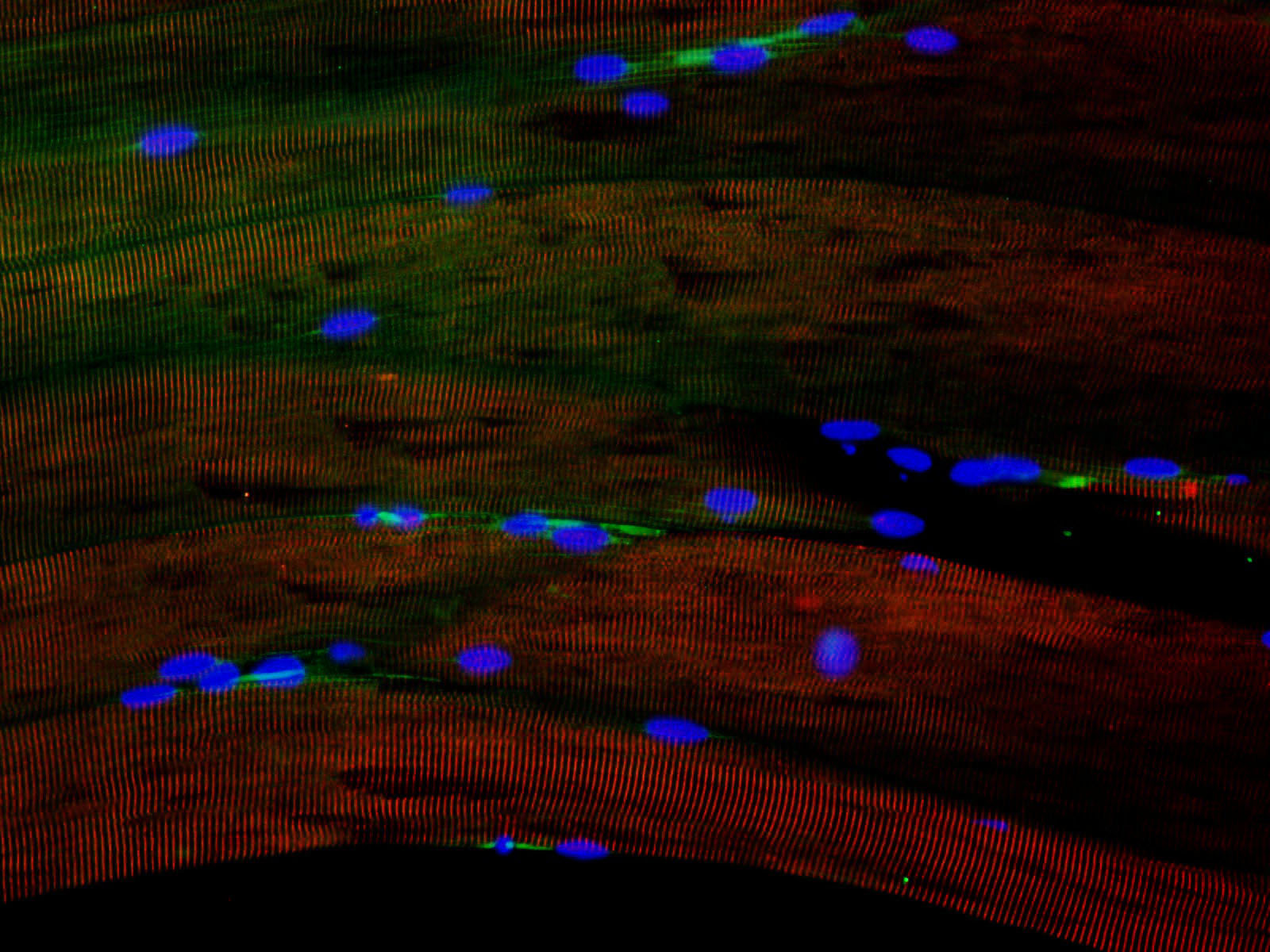

Supplement: Supplementary file 3 [file Data_Sheet_3.ZIP › microscopy images/IF/PKCα(red light)+Nox4(green light)/LVC (4).jpg]

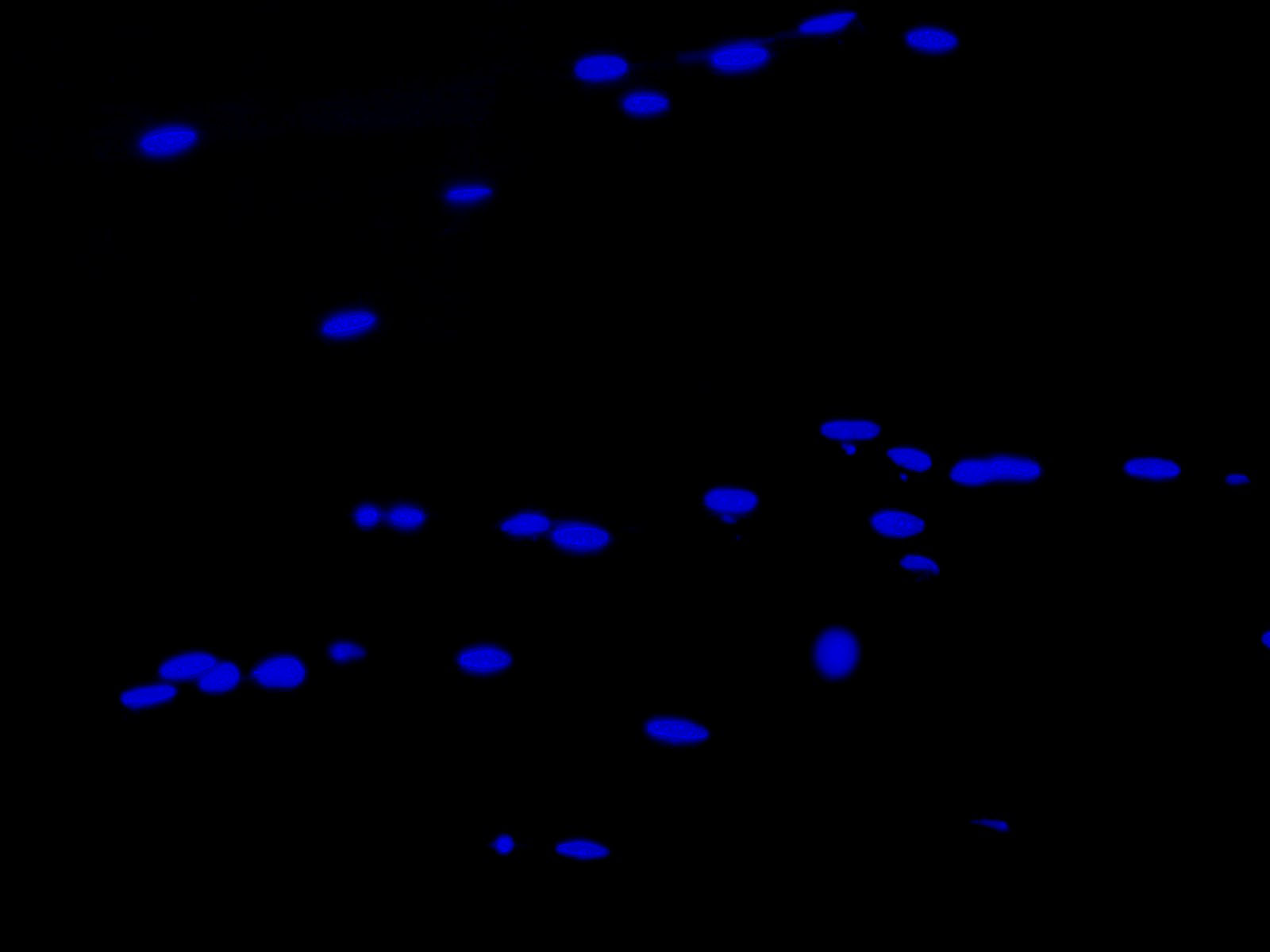

Supplement: Supplementary file 3 [file Data_Sheet_3.ZIP › microscopy images/IF/PKCα(red light)+Nox4(green light)/LVC(3).jpg]

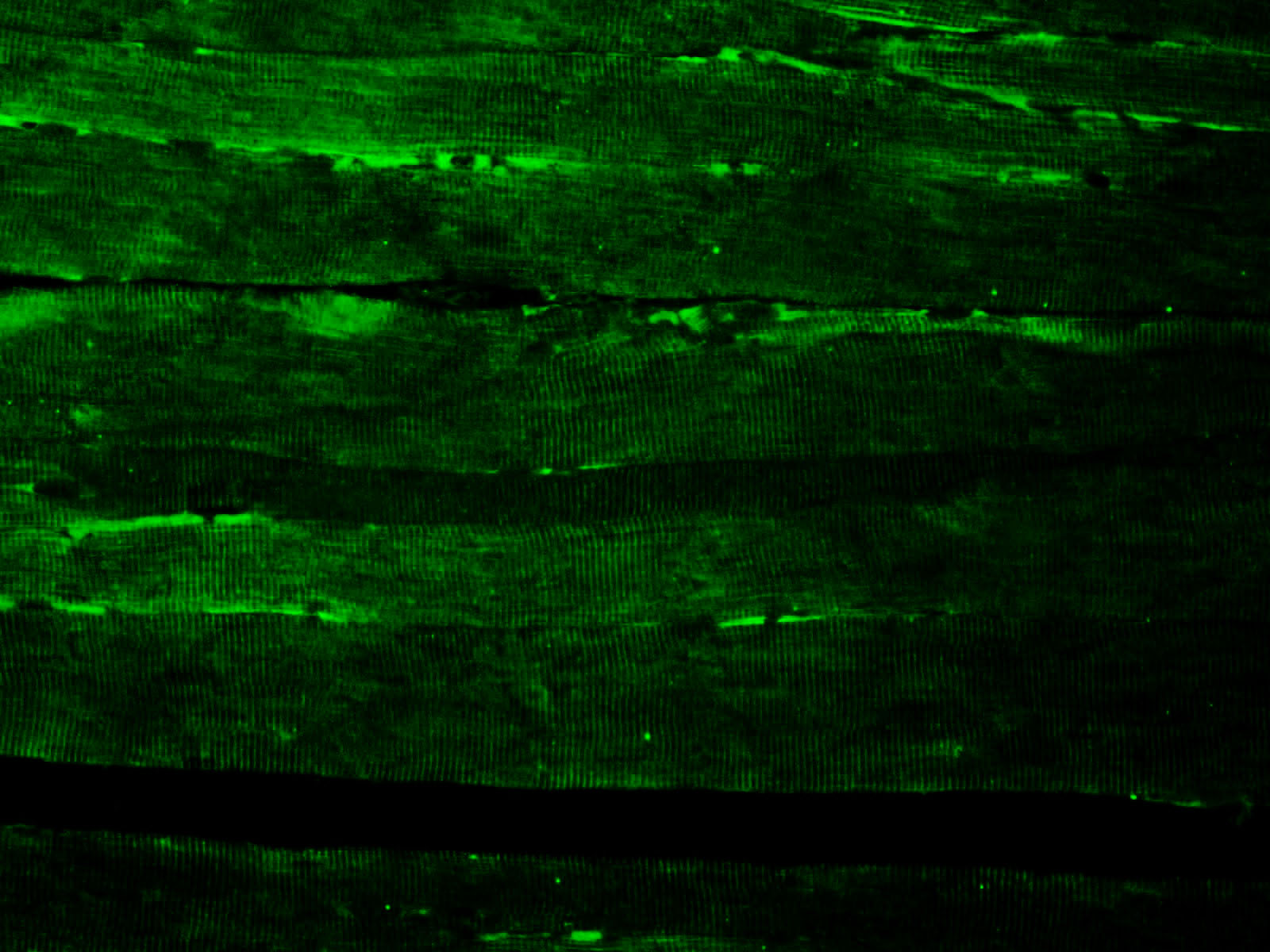

Supplement: Supplementary file 3 [file Data_Sheet_3.ZIP › microscopy images/IF/PKCα(red light)+Nox4(green light)/M (2).jpg]

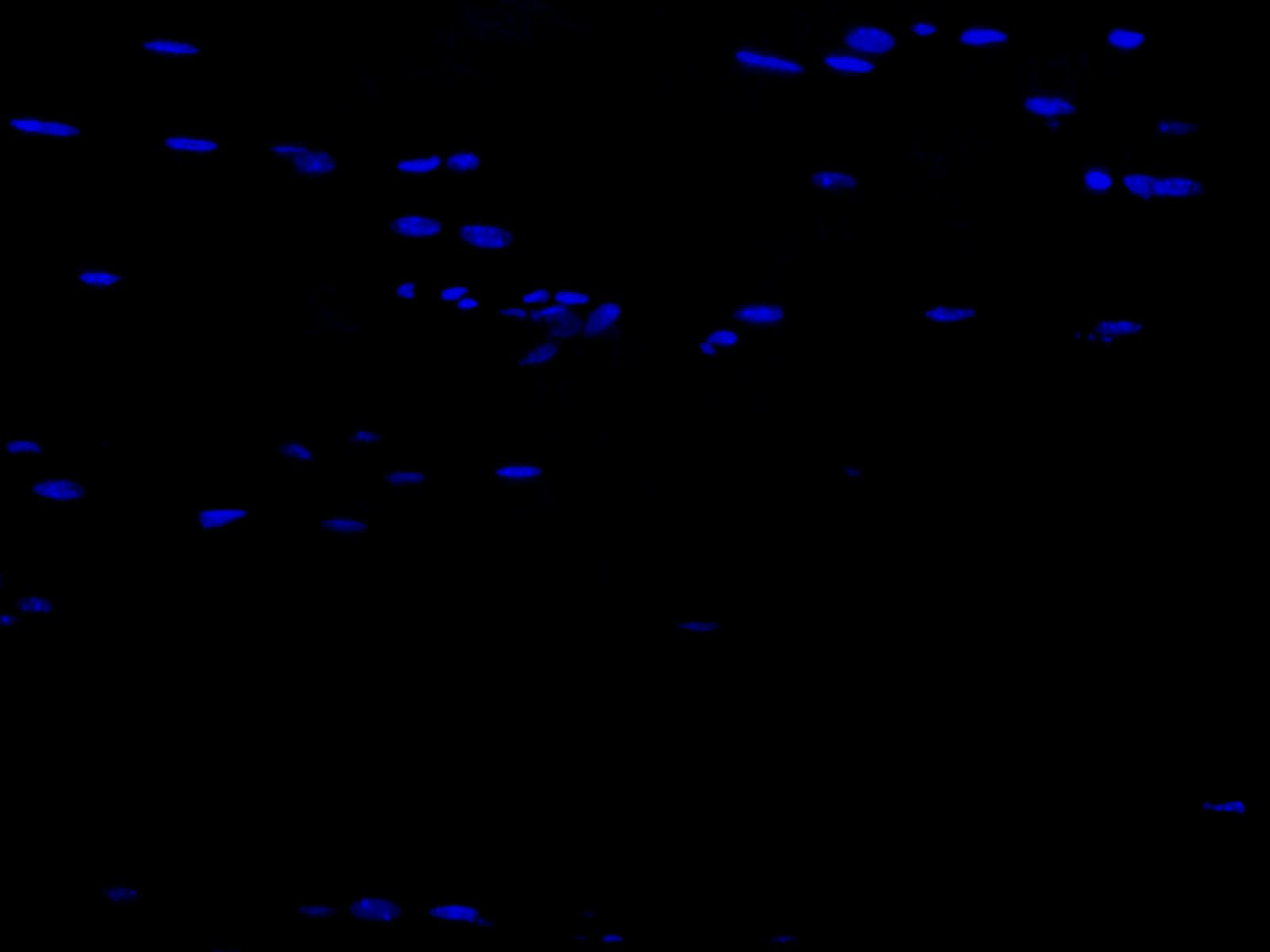

Supplement: Supplementary file 3 [file Data_Sheet_3.ZIP › microscopy images/IF/PKCα(red light)+Nox4(green light)/M (3).jpg]

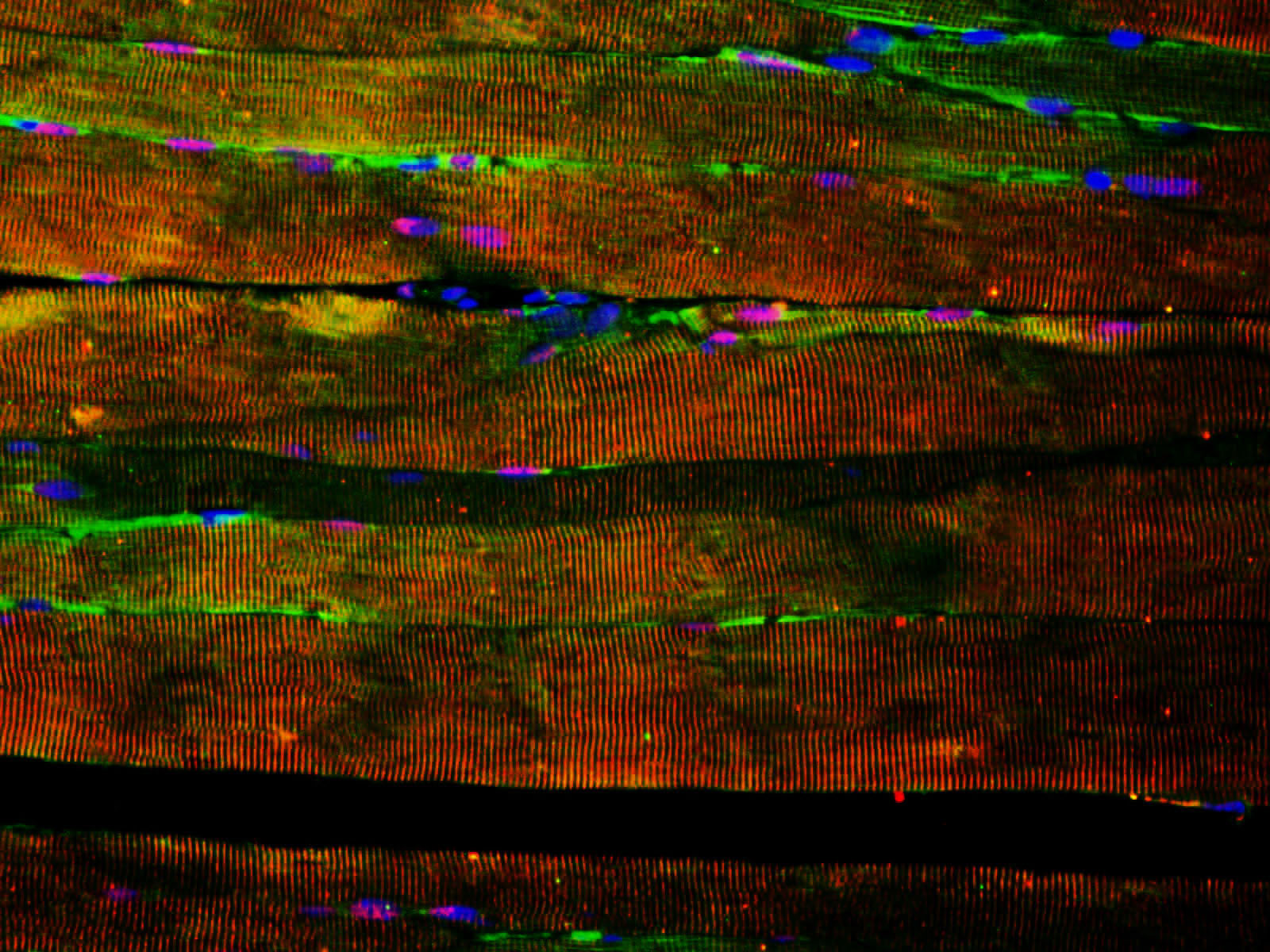

Supplement: Supplementary file 3 [file Data_Sheet_3.ZIP › microscopy images/IF/PKCα(red light)+Nox4(green light)/M (4).jpg]

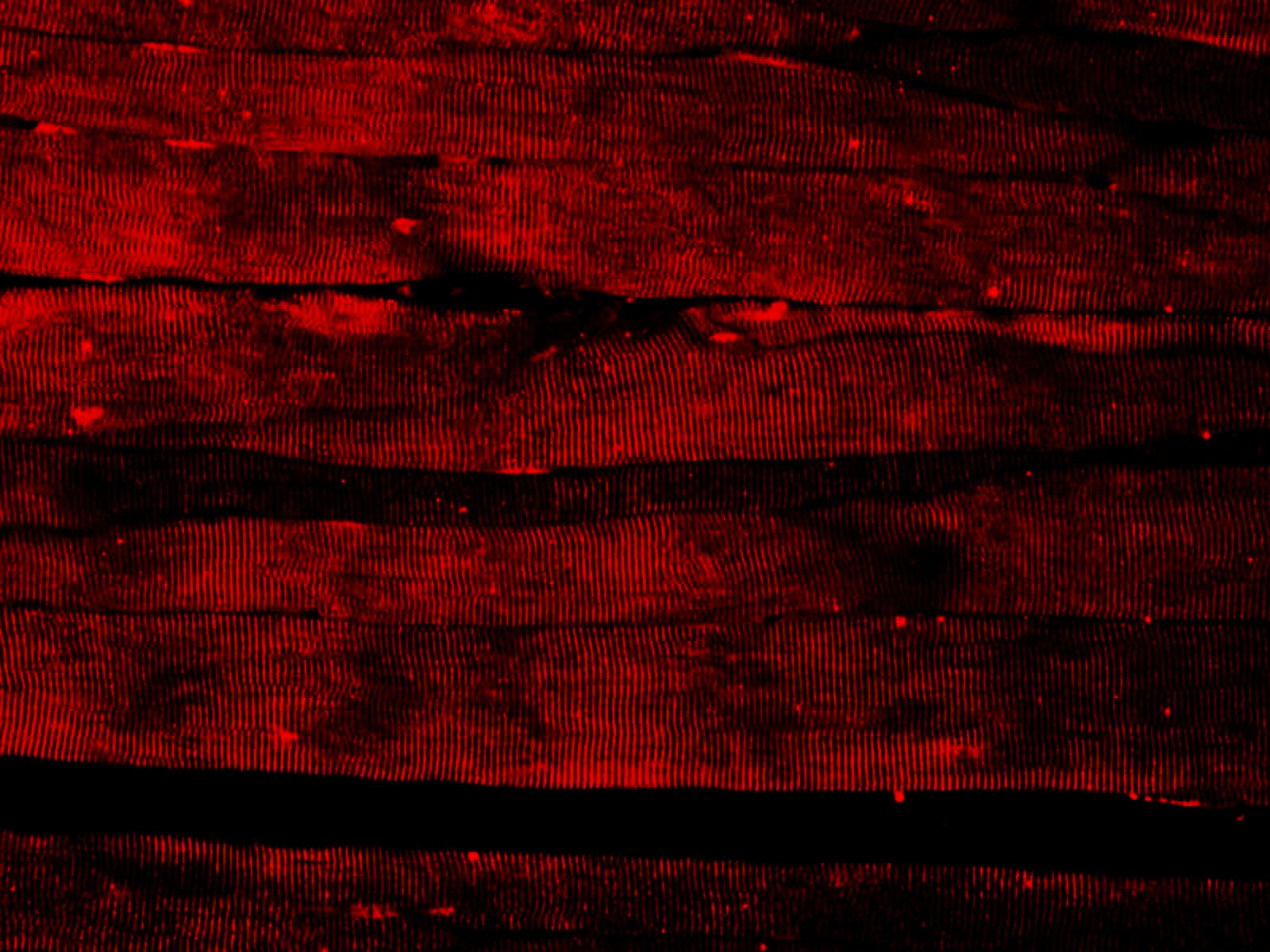

Supplement: Supplementary file 3 [file Data_Sheet_3.ZIP › microscopy images/IF/PKCα(red light)+Nox4(green light)/M(1).jpg]

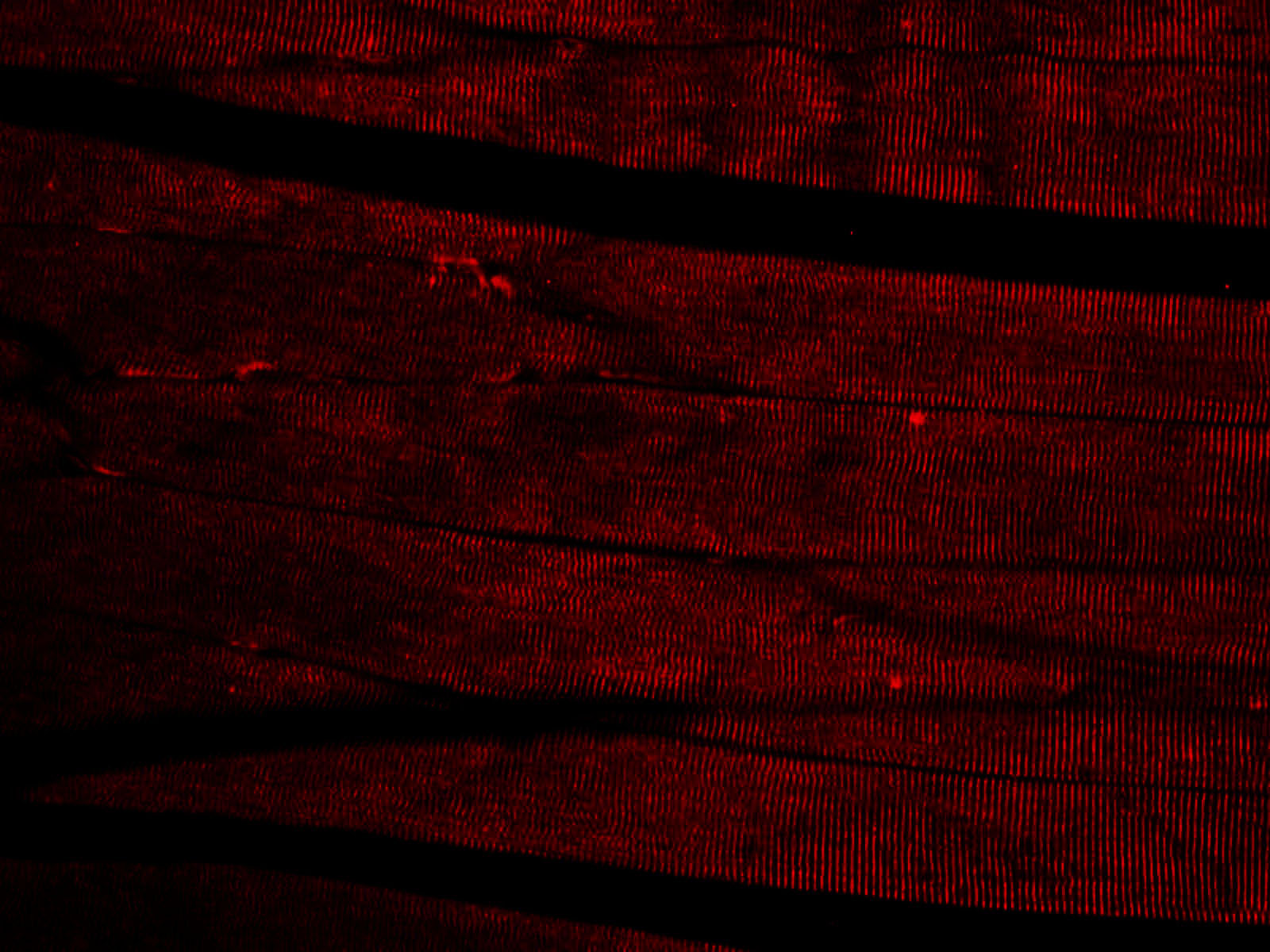

Supplement: Supplementary file 3 [file Data_Sheet_3.ZIP › microscopy images/IF/PKCα(red light)+Nox4(green light)/VC (1).jpg]

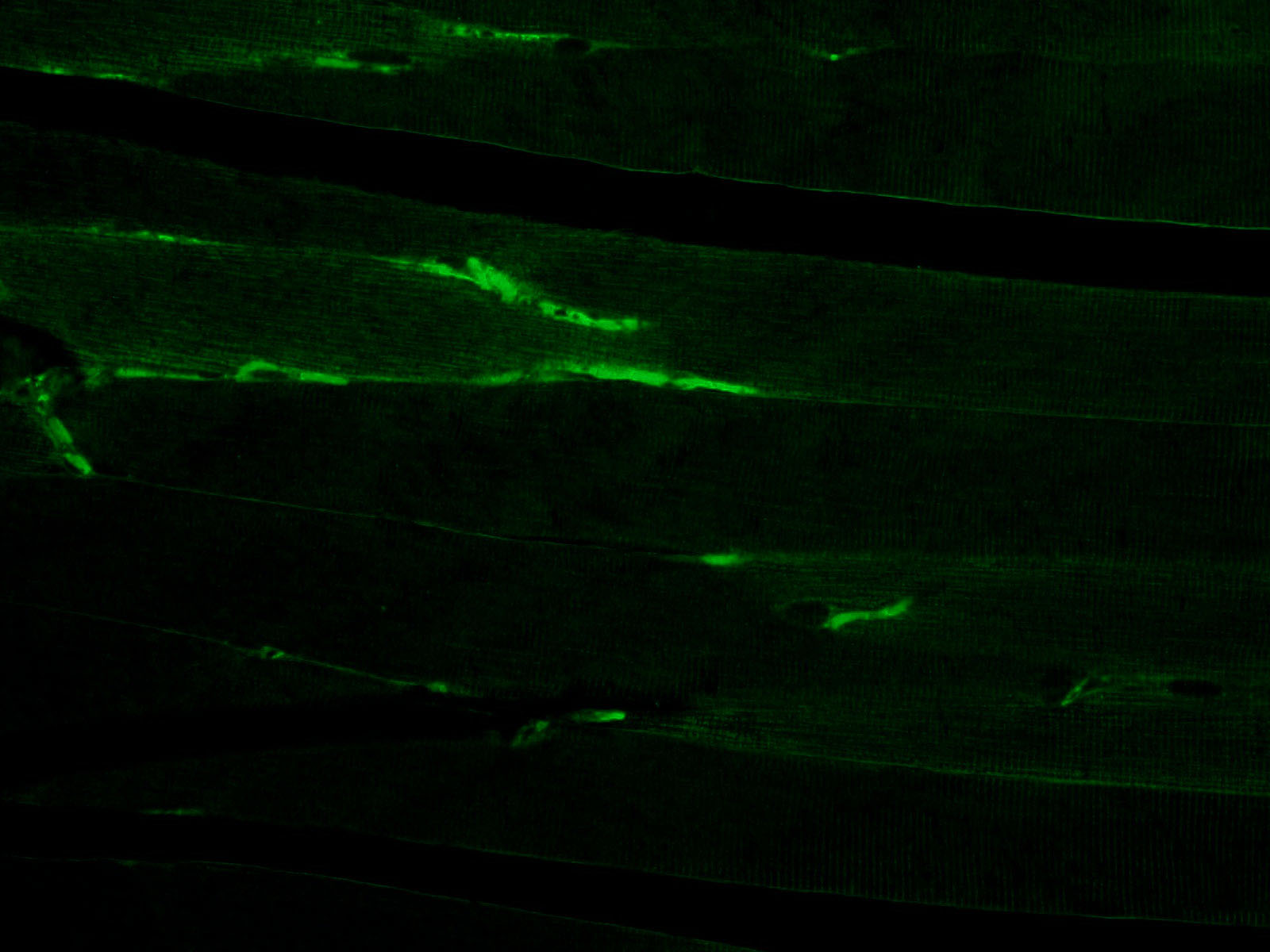

Supplement: Supplementary file 3 [file Data_Sheet_3.ZIP › microscopy images/IF/PKCα(red light)+Nox4(green light)/VC (2).jpg]

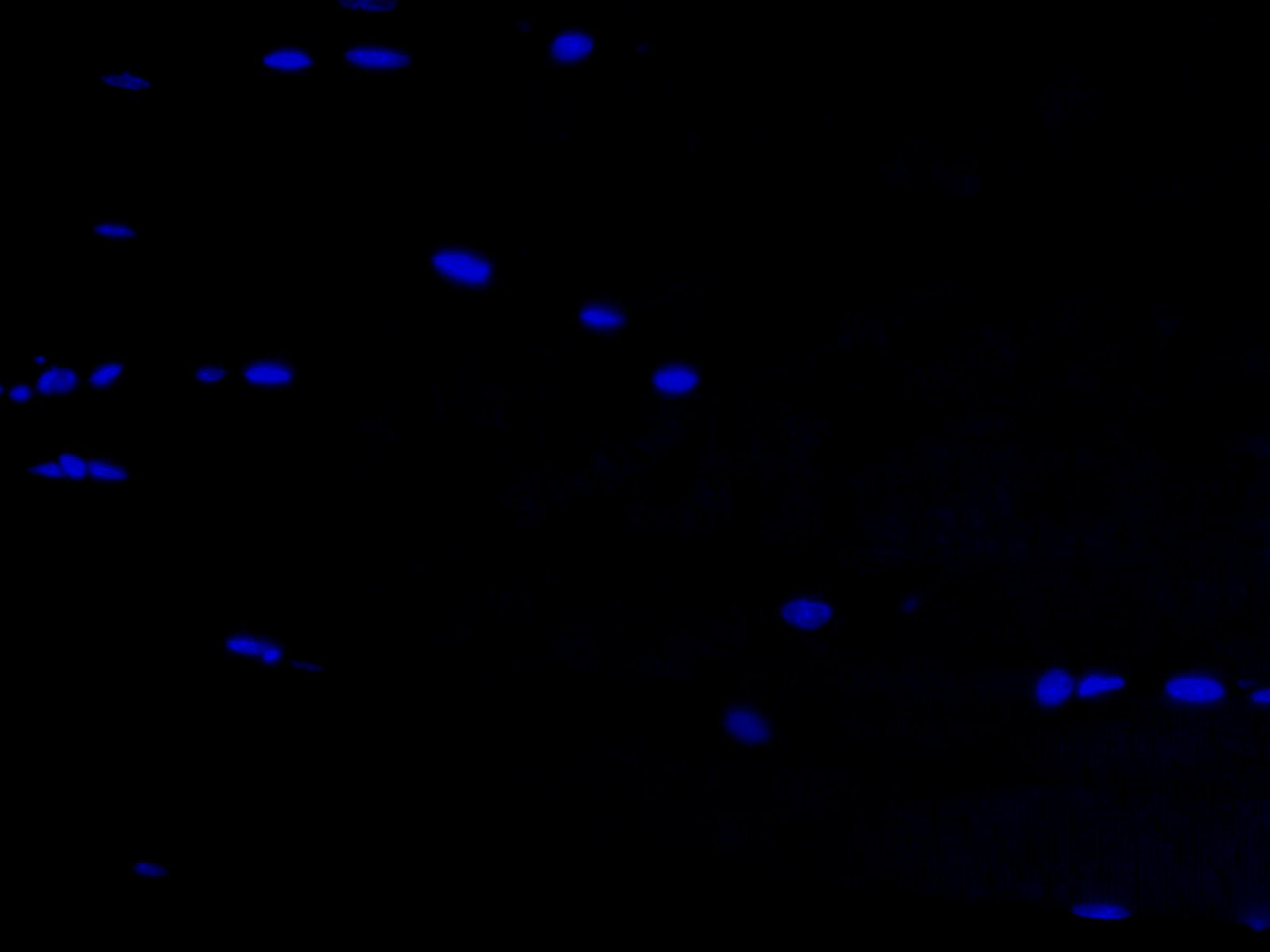

Supplement: Supplementary file 3 [file Data_Sheet_3.ZIP › microscopy images/IF/PKCα(red light)+Nox4(green light)/VC (3).jpg]

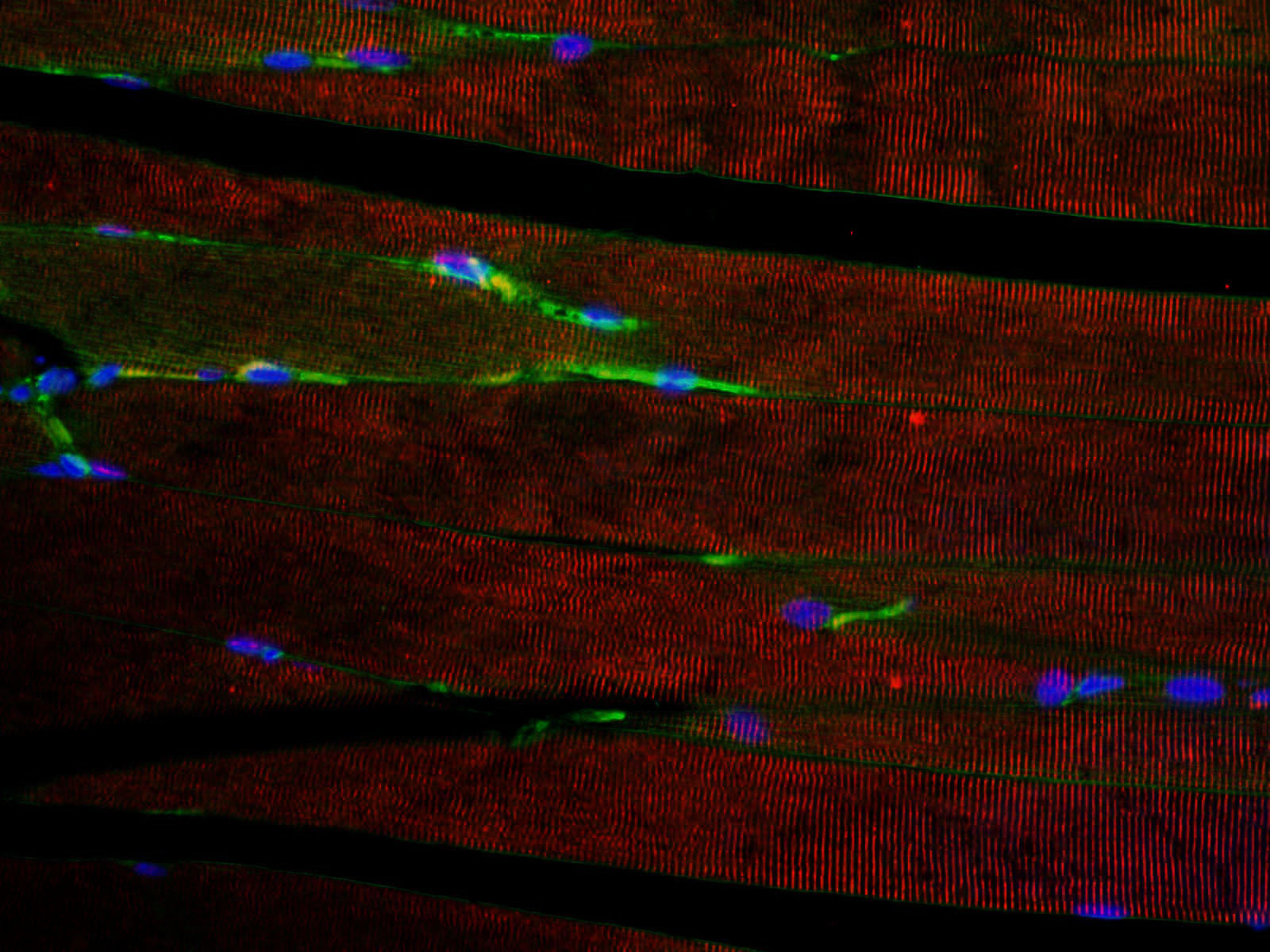

Supplement: Supplementary file 3 [file Data_Sheet_3.ZIP › microscopy images/IF/PKCα(red light)+Nox4(green light)/VC (4).jpg]

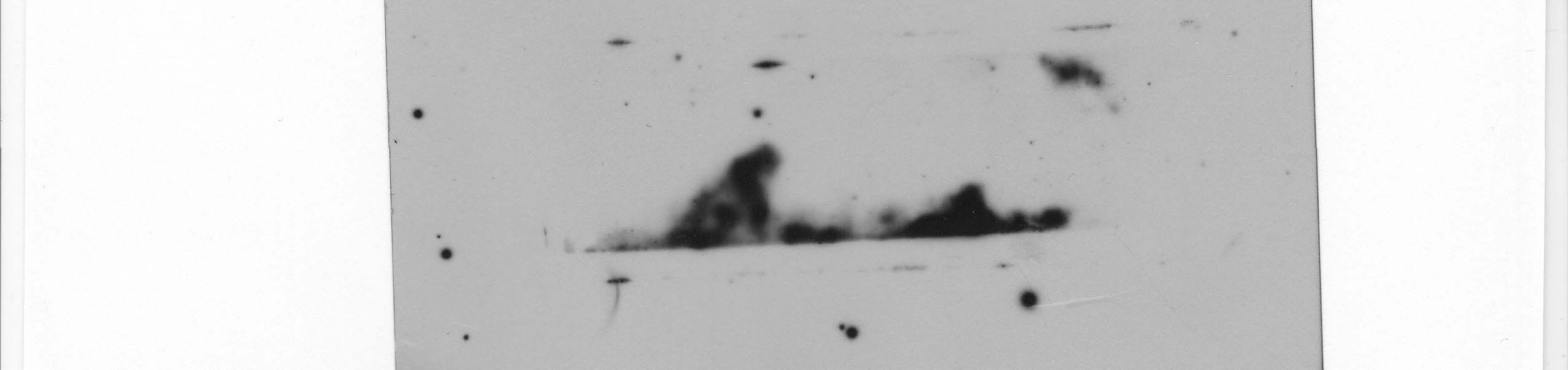

Supplement: Supplementary file 4 [file Data_Sheet_4.ZIP › Western blot figure 2/Immunoprecipitation technique/IgG-nox2.jpg]

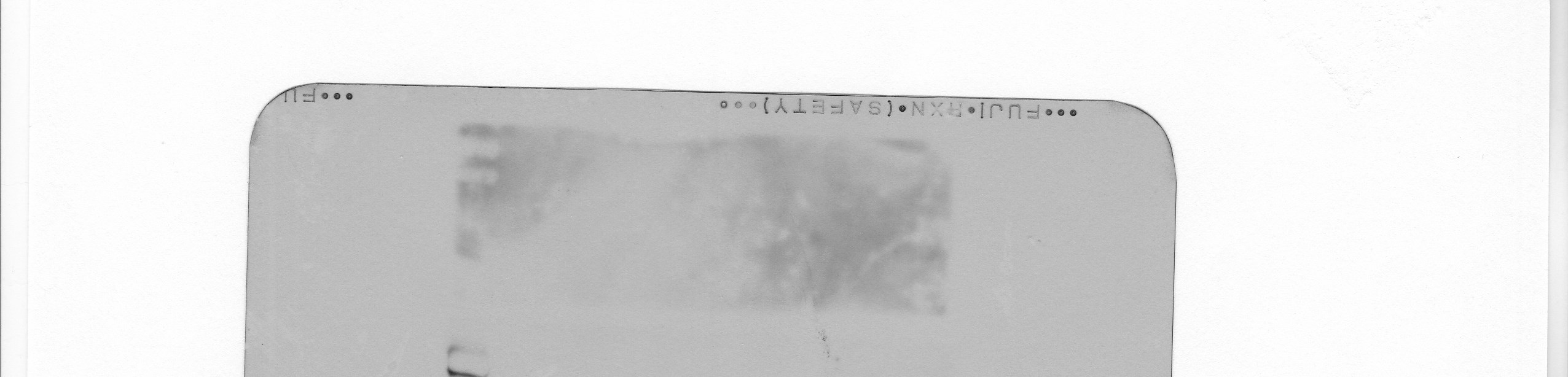

Supplement: Supplementary file 4 [file Data_Sheet_4.ZIP › Western blot figure 2/Immunoprecipitation technique/IgG-nox4.jpg]

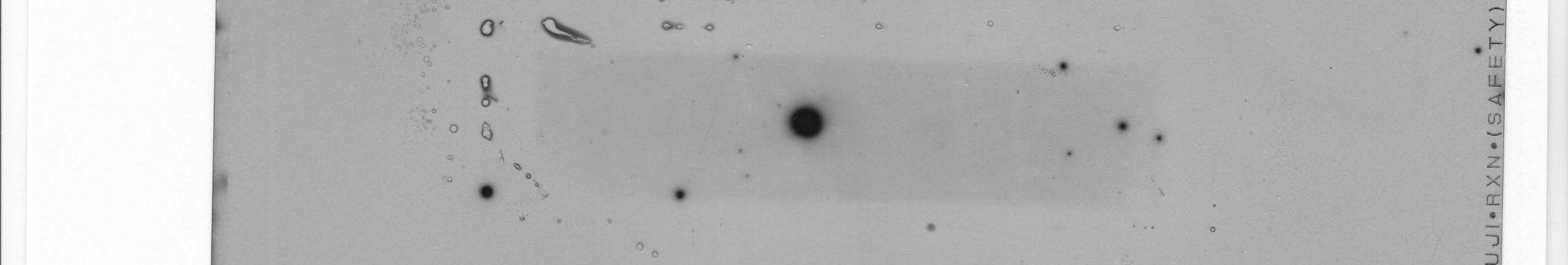

Supplement: Supplementary file 4 [file Data_Sheet_4.ZIP › Western blot figure 2/Immunoprecipitation technique/IgG-pkc.jpg]

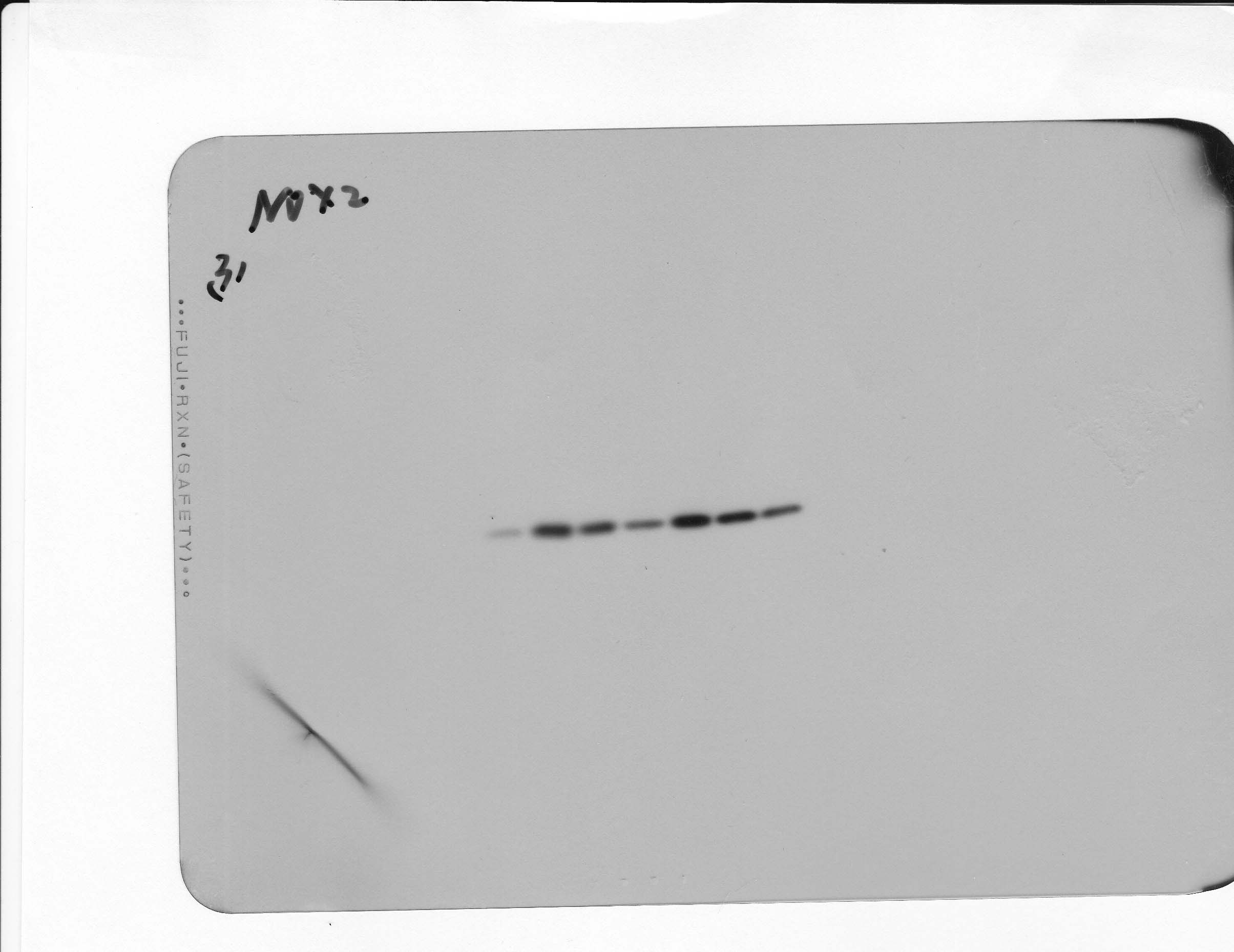

Supplement: Supplementary file 4 [file Data_Sheet_4.ZIP › Western blot figure 2/Immunoprecipitation technique/nox2.jpg]

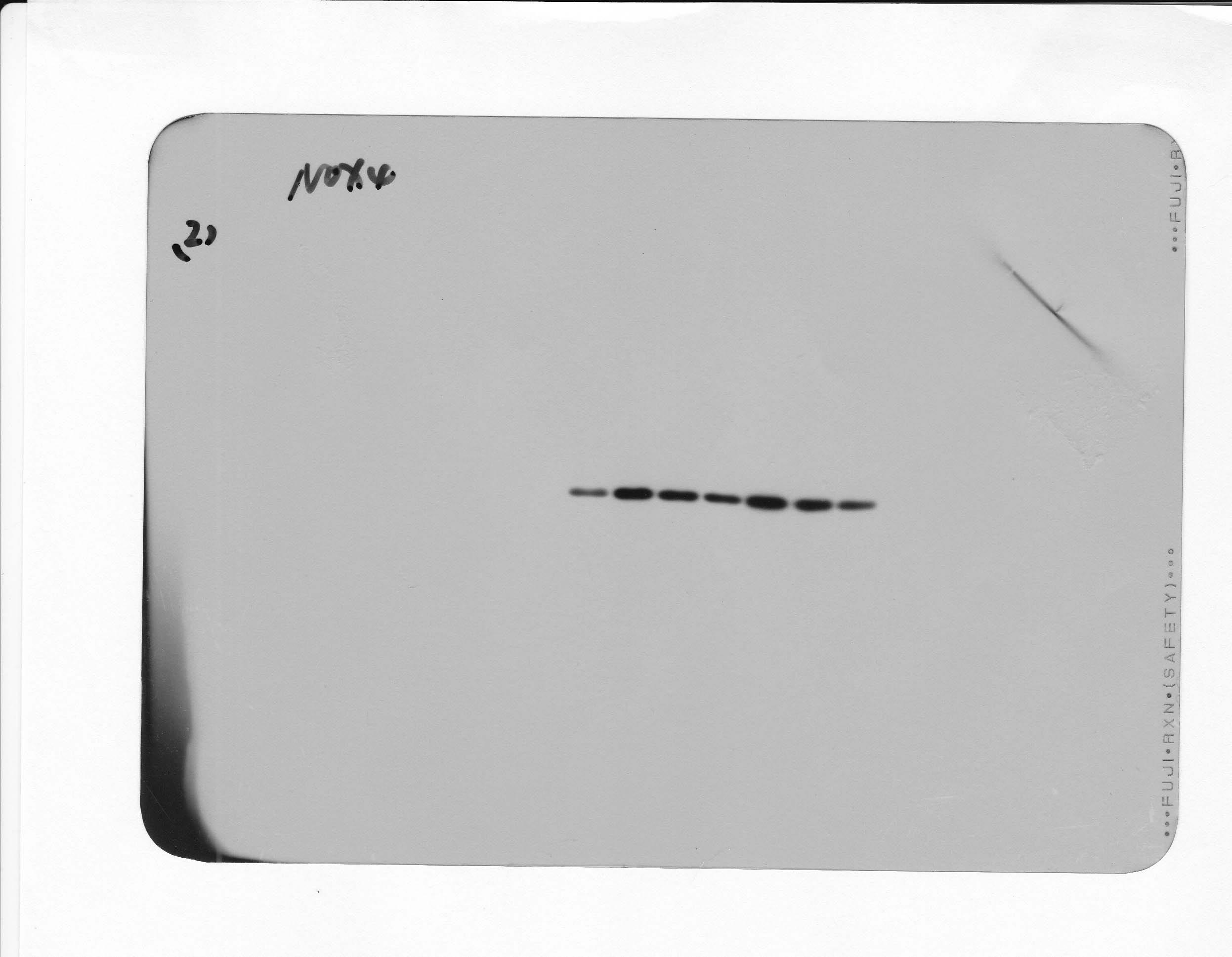

Supplement: Supplementary file 4 [file Data_Sheet_4.ZIP › Western blot figure 2/Immunoprecipitation technique/nox4.jpg]

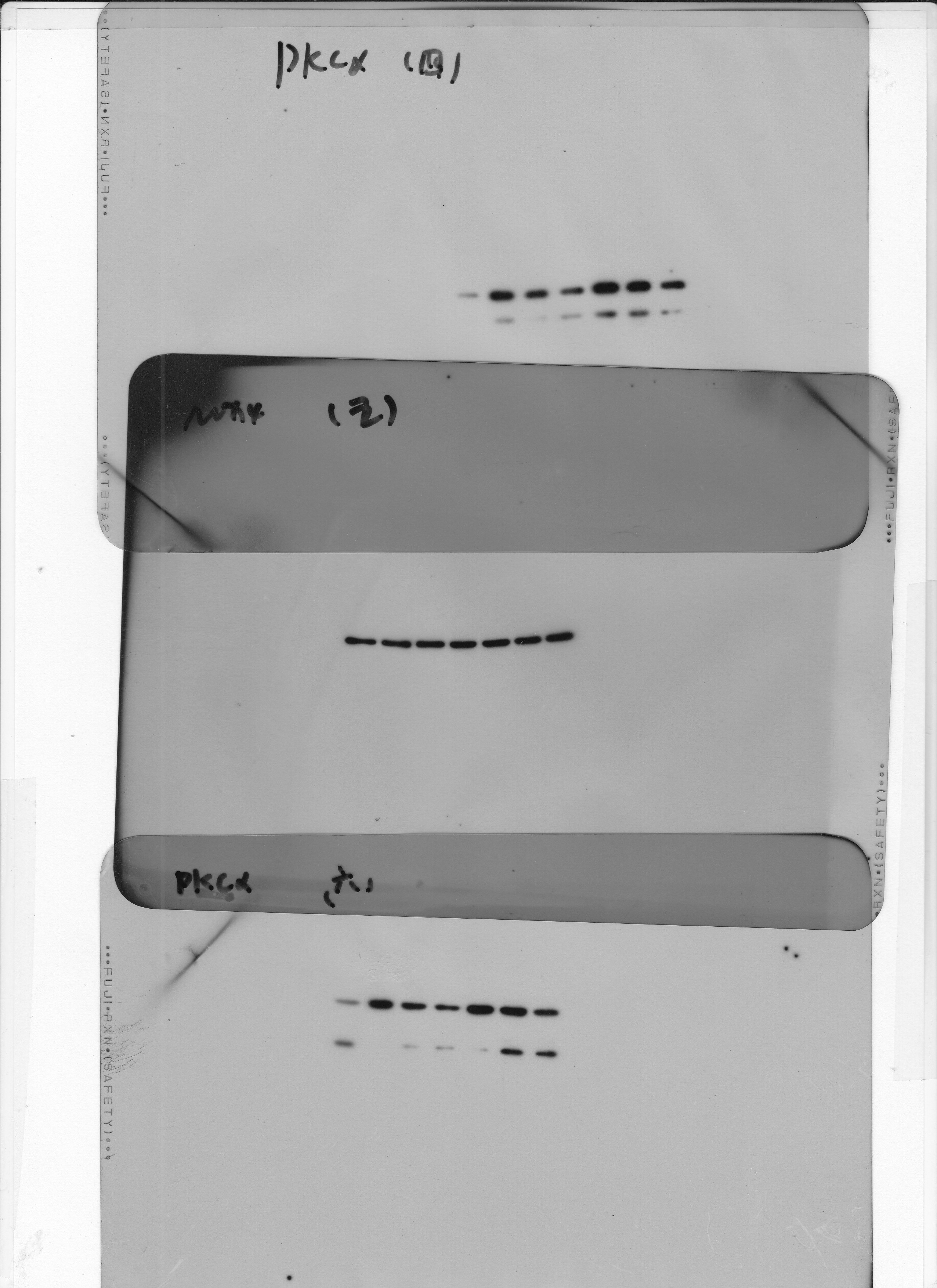

Supplement: Supplementary file 4 [file Data_Sheet_4.ZIP › Western blot figure 2/Immunoprecipitation technique/pkc-nox4 (2).jpg]

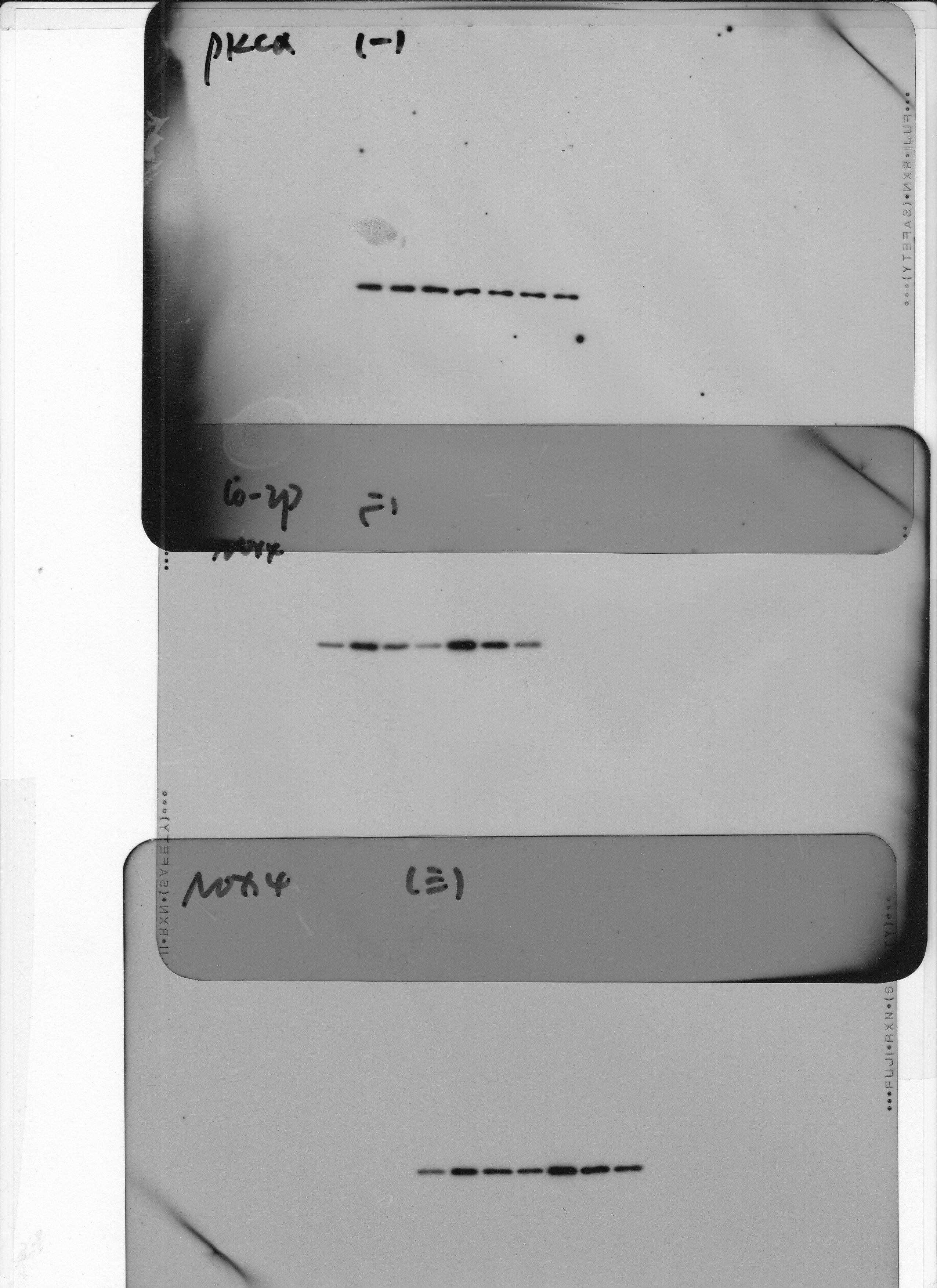

Supplement: Supplementary file 4 [file Data_Sheet_4.ZIP › Western blot figure 2/Immunoprecipitation technique/pkc-nox4.jpg]

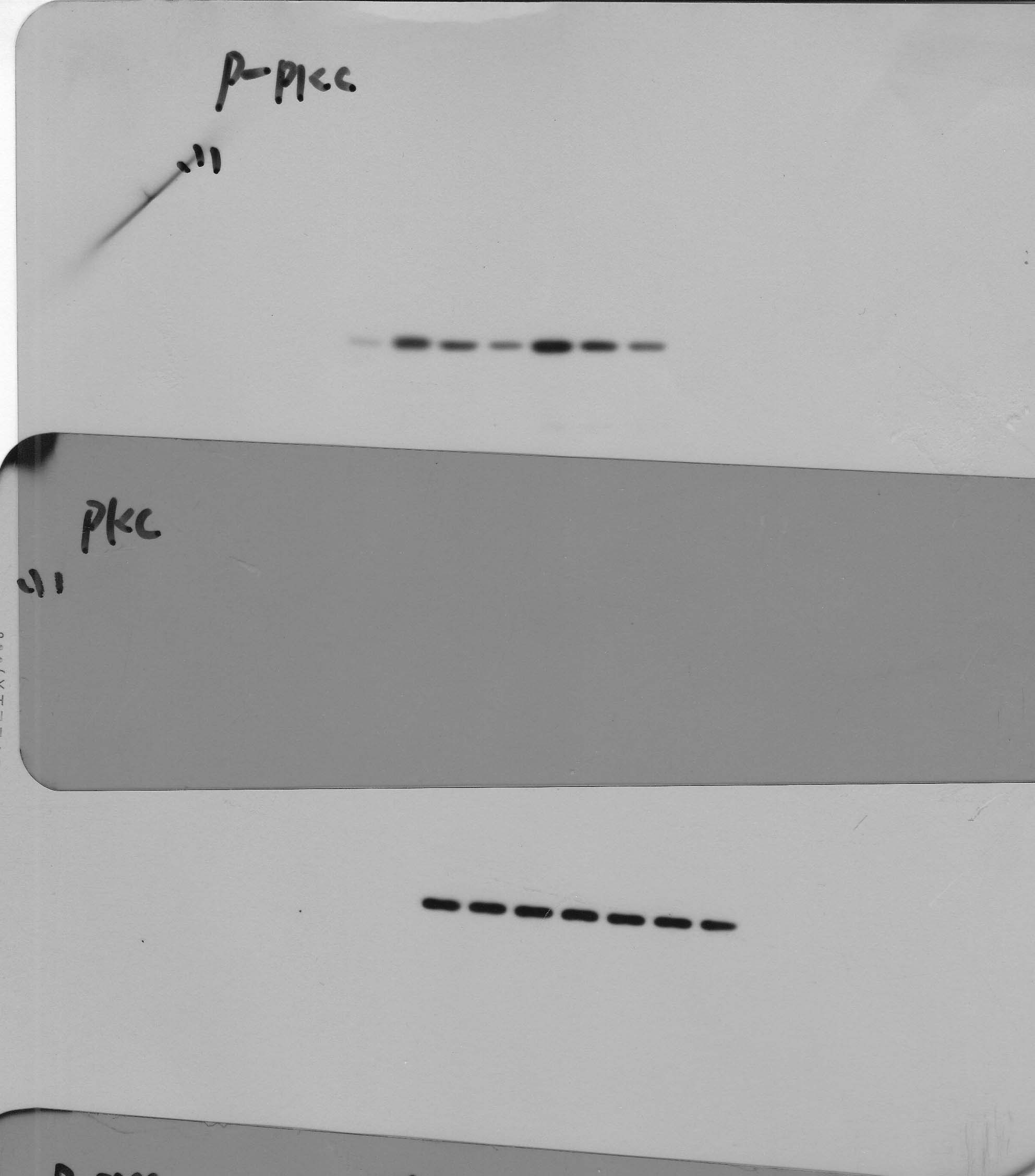

Supplement: Supplementary file 4 [file Data_Sheet_4.ZIP › Western blot figure 2/Immunoprecipitation technique/pkc.jpg]

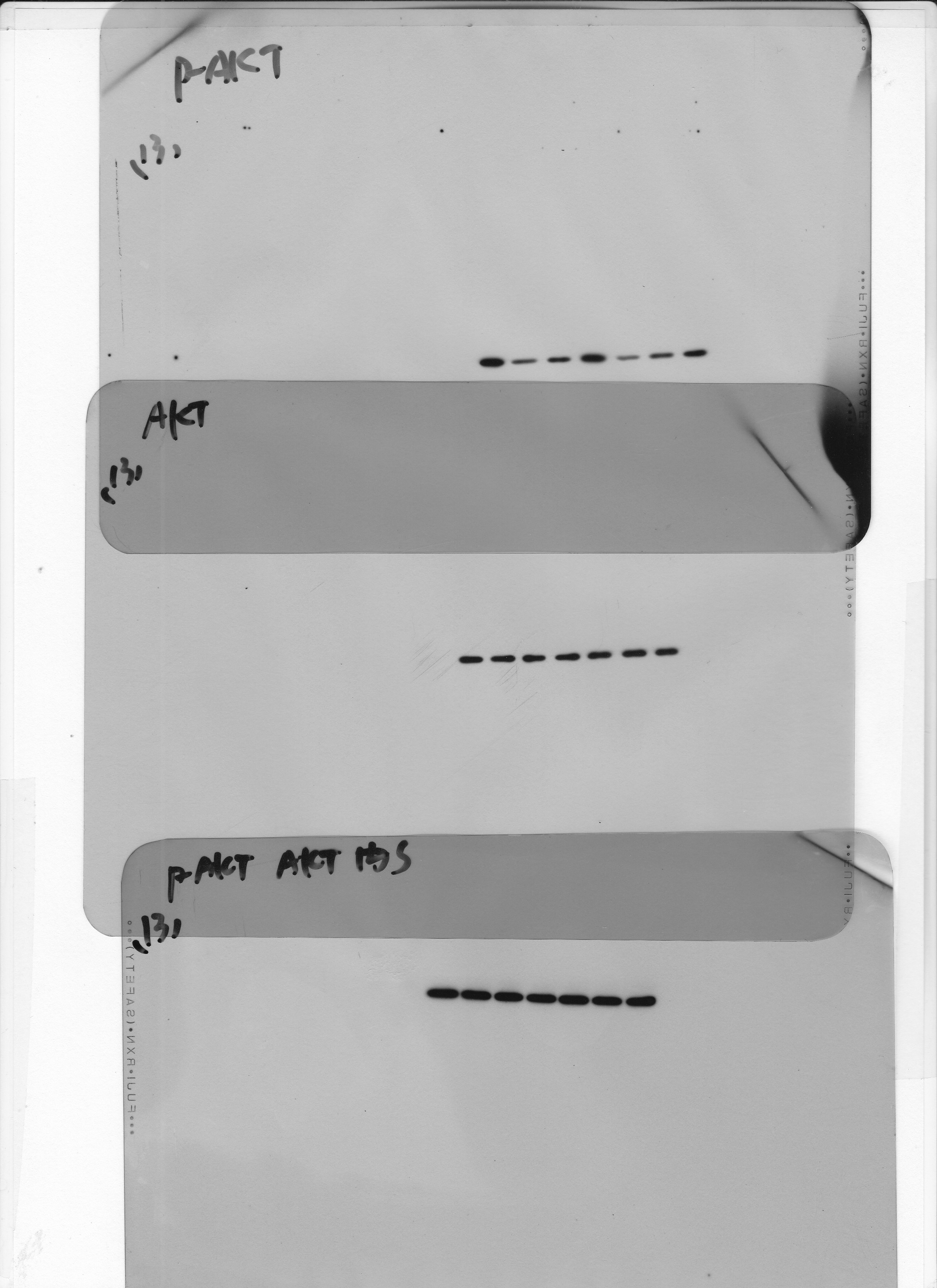

Supplement: Supplementary file 4 [file Data_Sheet_4.ZIP › Western blot figure 2/p-AKT,AKT.jpg]

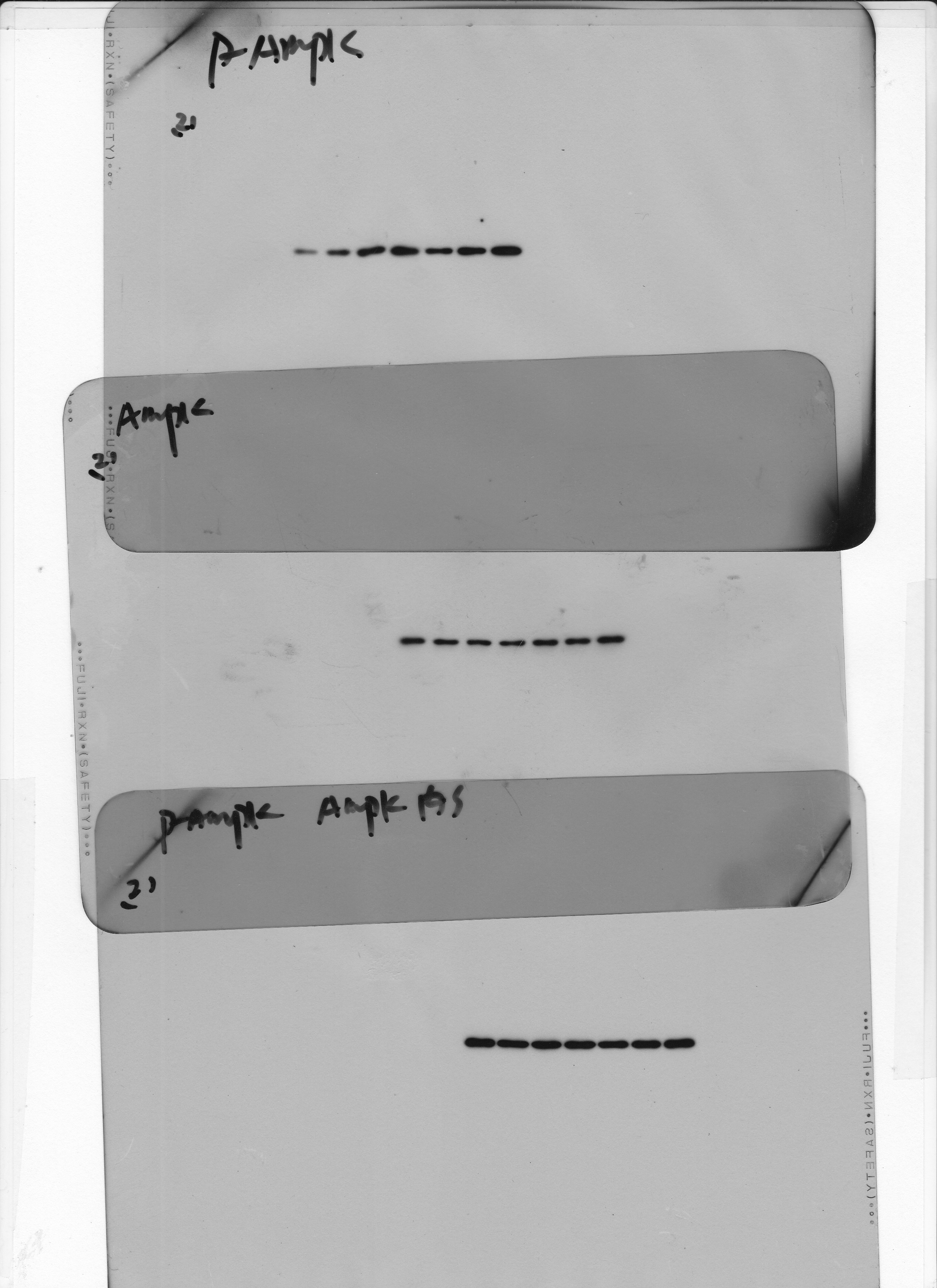

Supplement: Supplementary file 4 [file Data_Sheet_4.ZIP › Western blot figure 2/p-AMPK, AMPK.jpg]

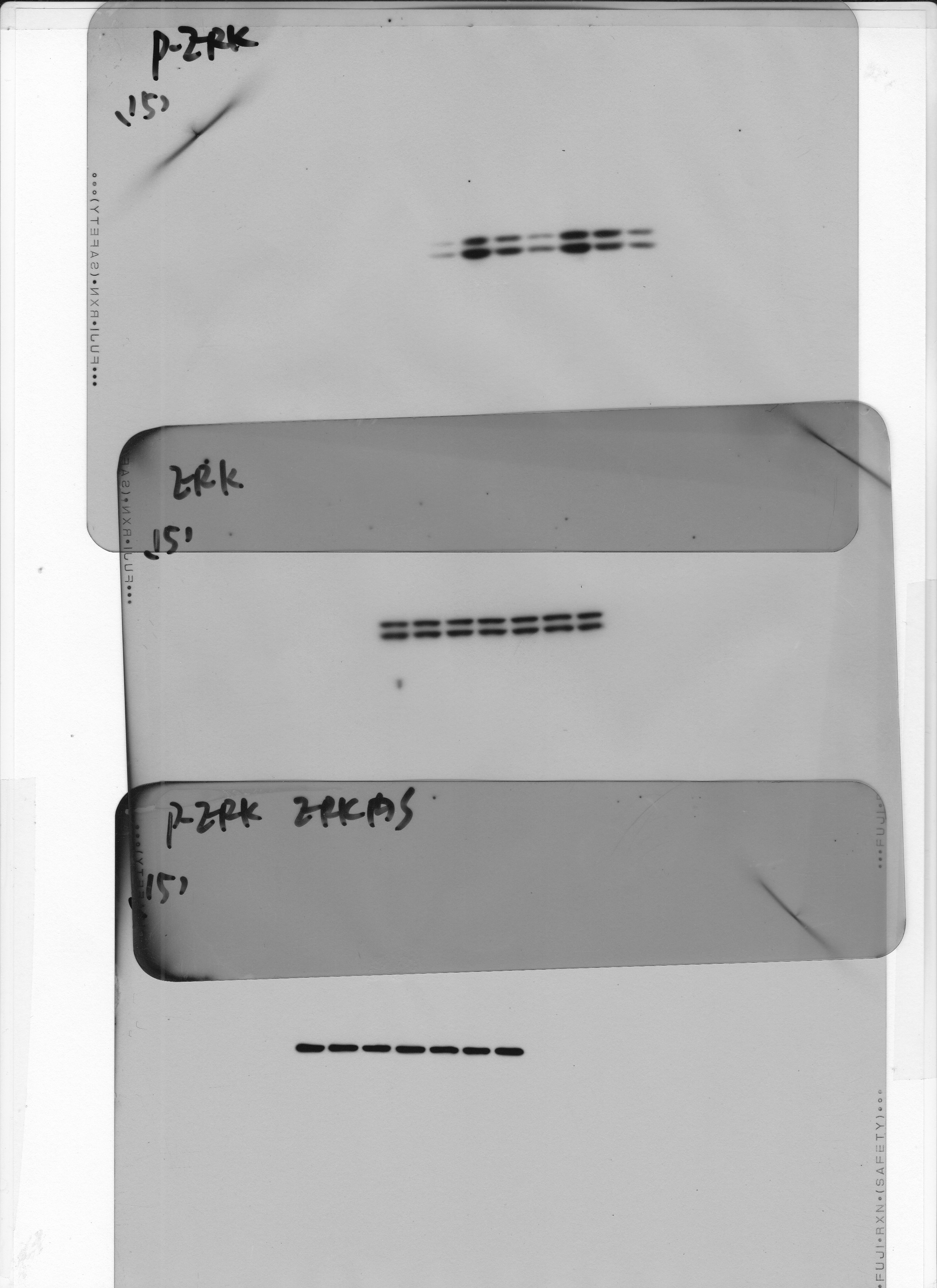

Supplement: Supplementary file 4 [file Data_Sheet_4.ZIP › Western blot figure 2/p-ERK, ERK.jpg]

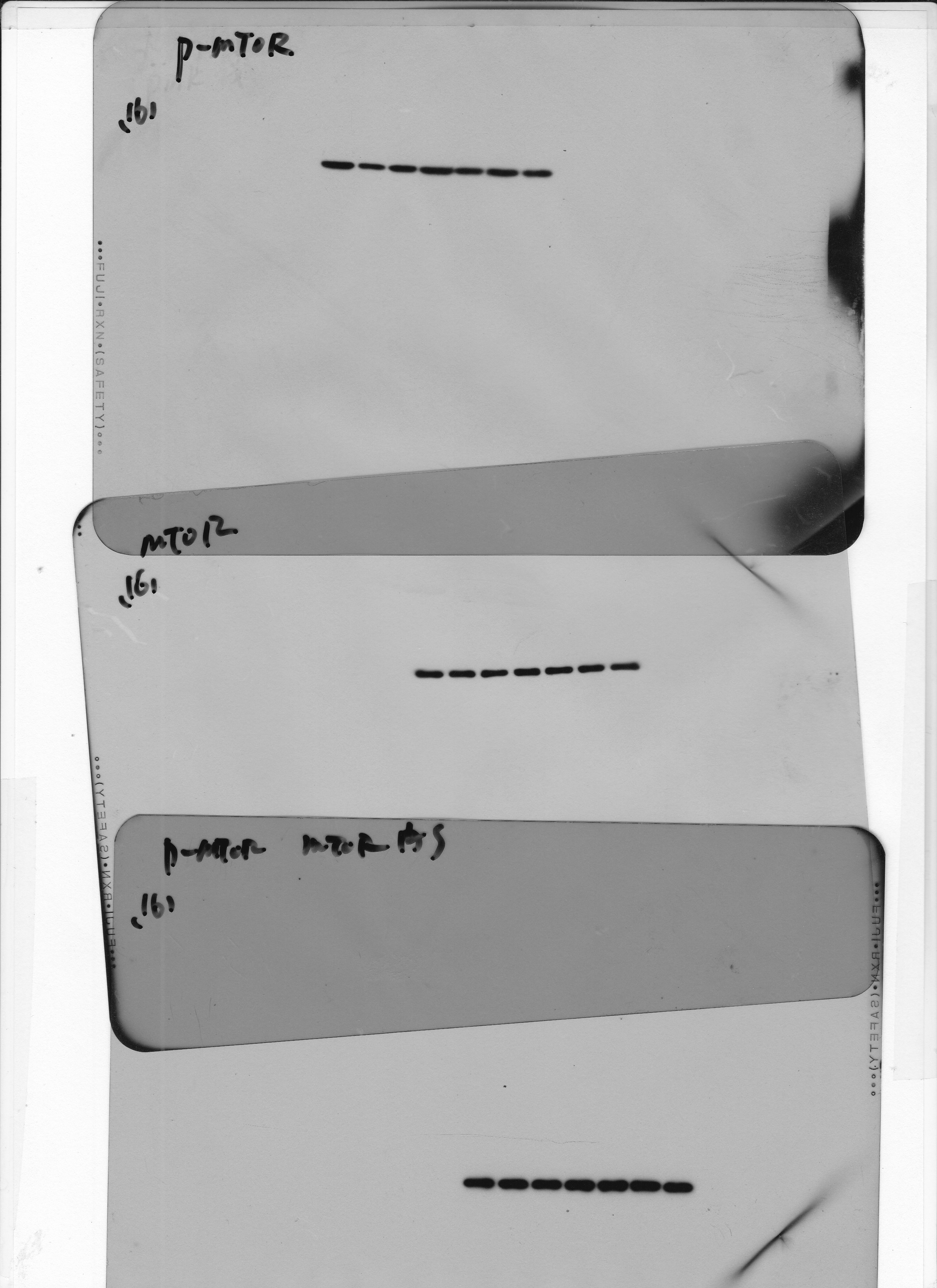

Supplement: Supplementary file 4 [file Data_Sheet_4.ZIP › Western blot figure 2/p-mTOR, mTOR.jpg]

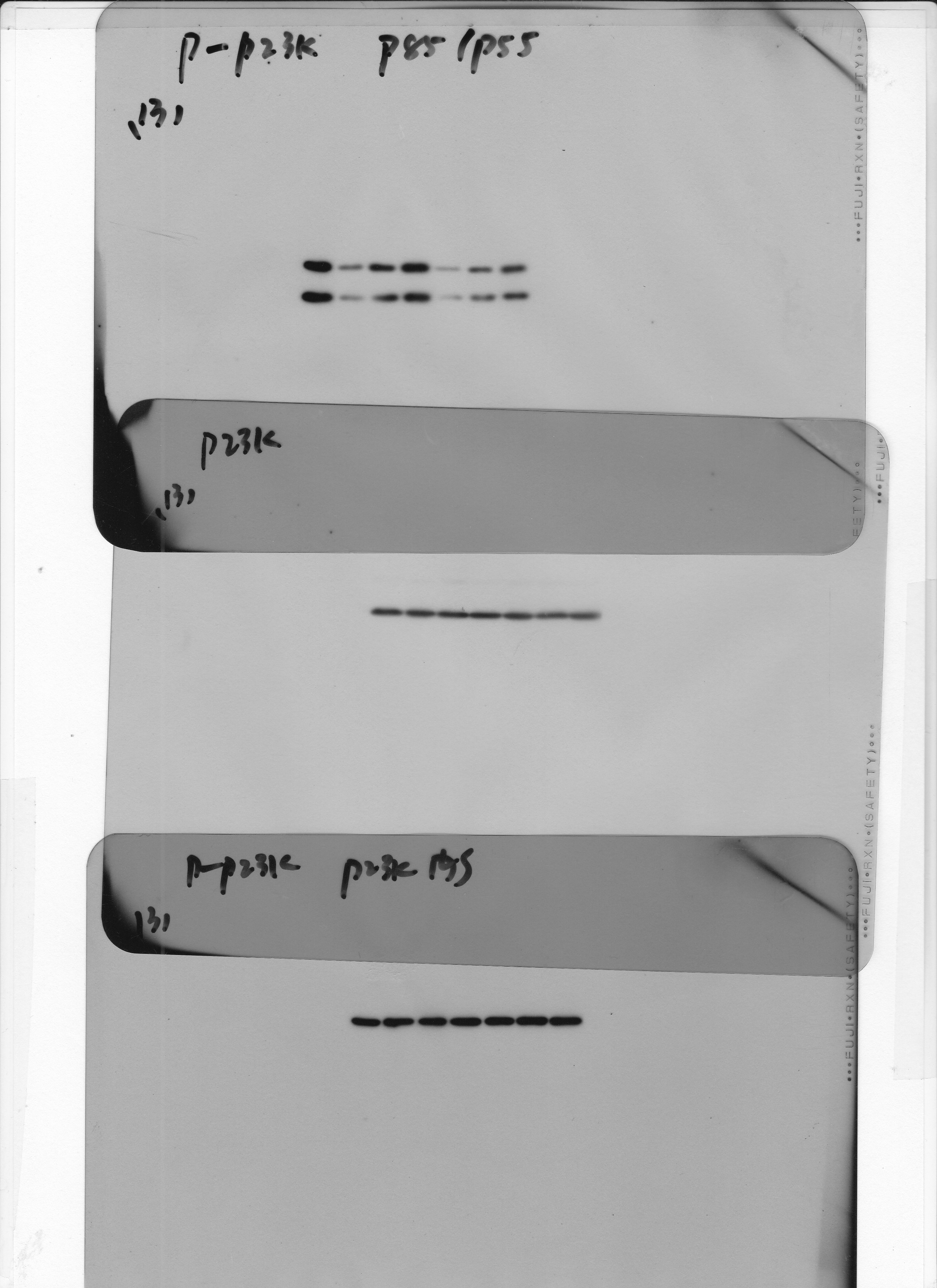

Supplement: Supplementary file 4 [file Data_Sheet_4.ZIP › Western blot figure 2/PI3K.jpg]

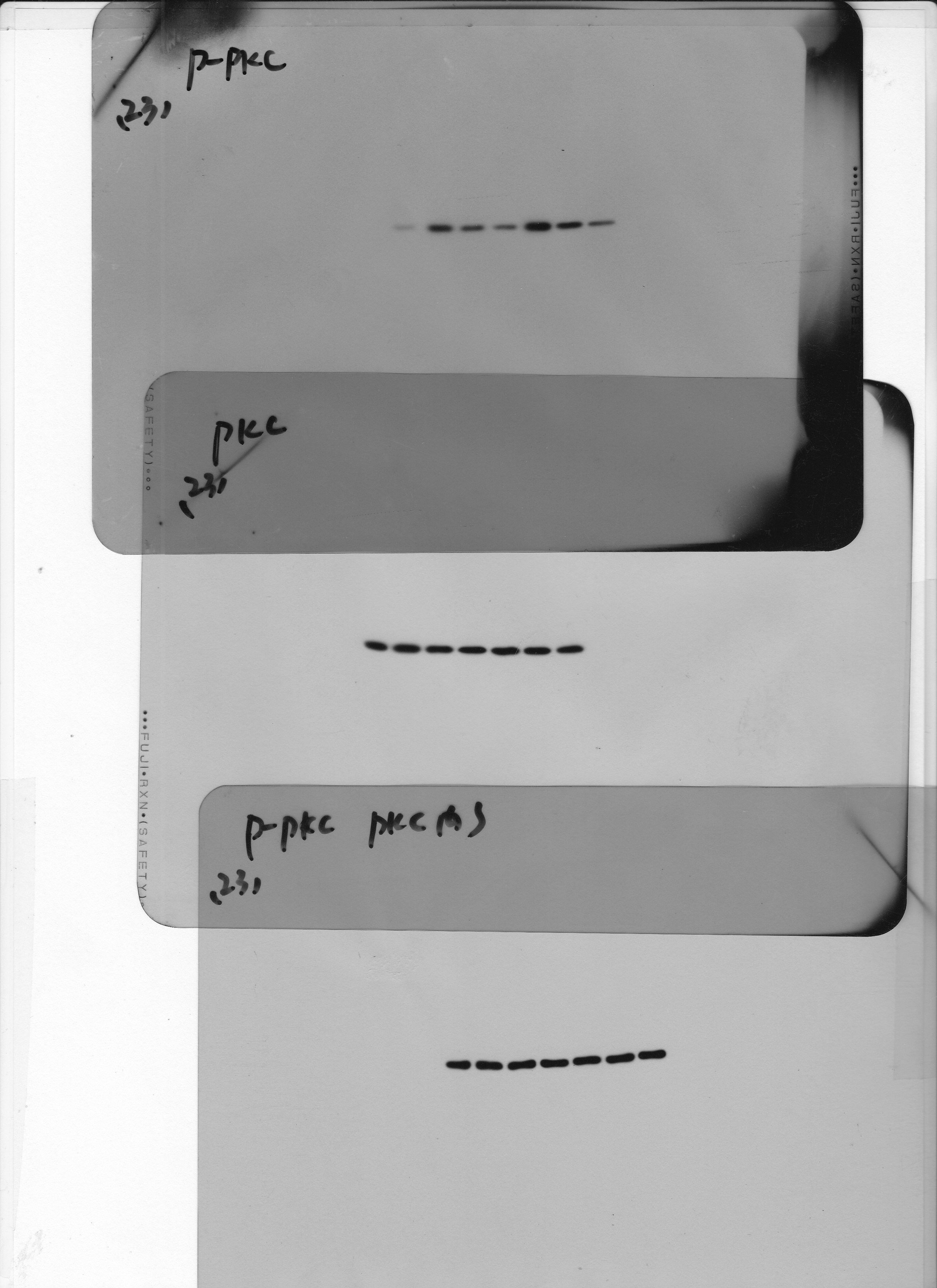

Supplement: Supplementary file 4 [file Data_Sheet_4.ZIP › Western blot figure 2/pkc (2).jpg]

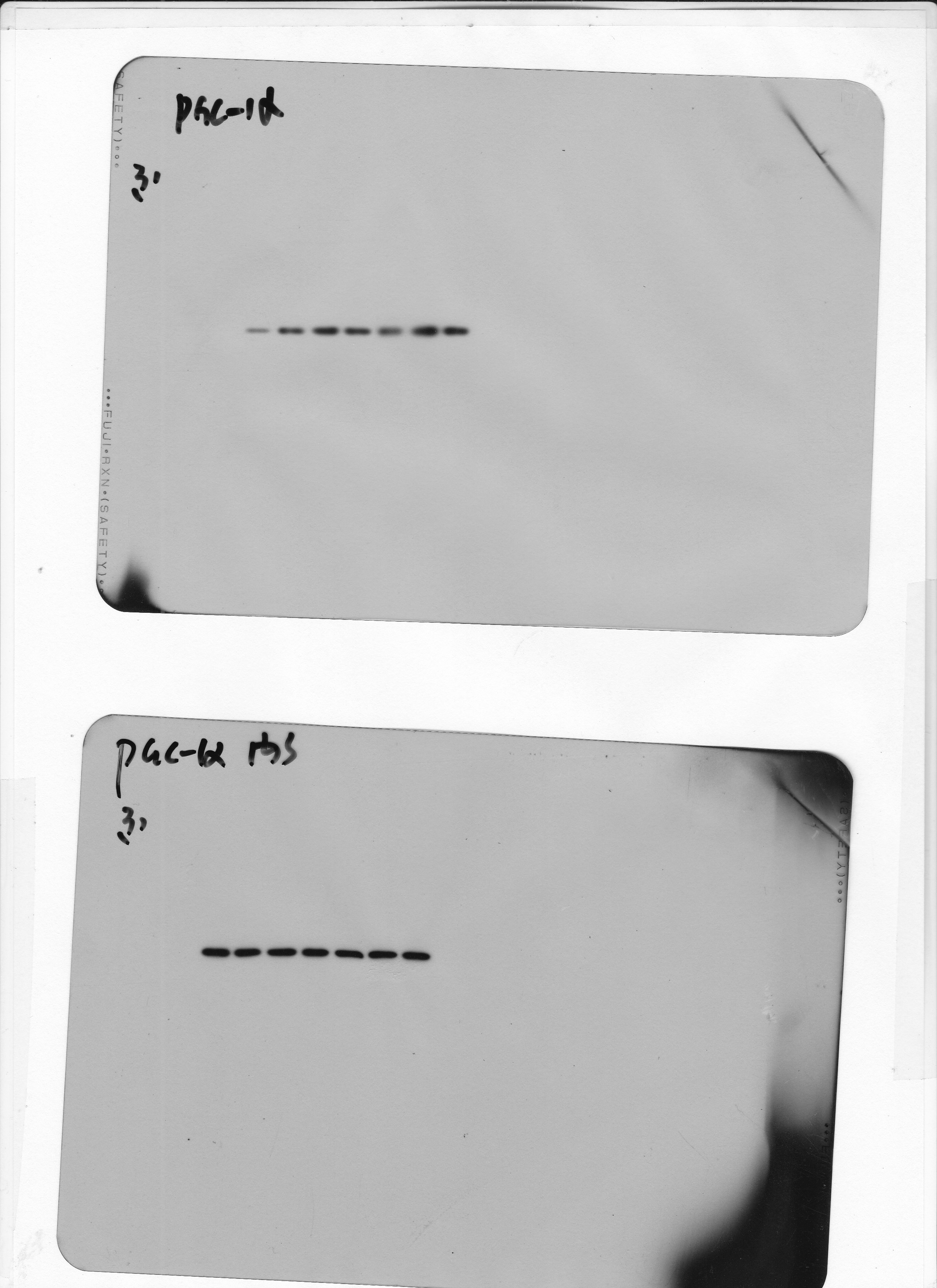

Supplement: Supplementary file 4 [file Data_Sheet_4.ZIP › Western blot figure 2/pkc.jpg]

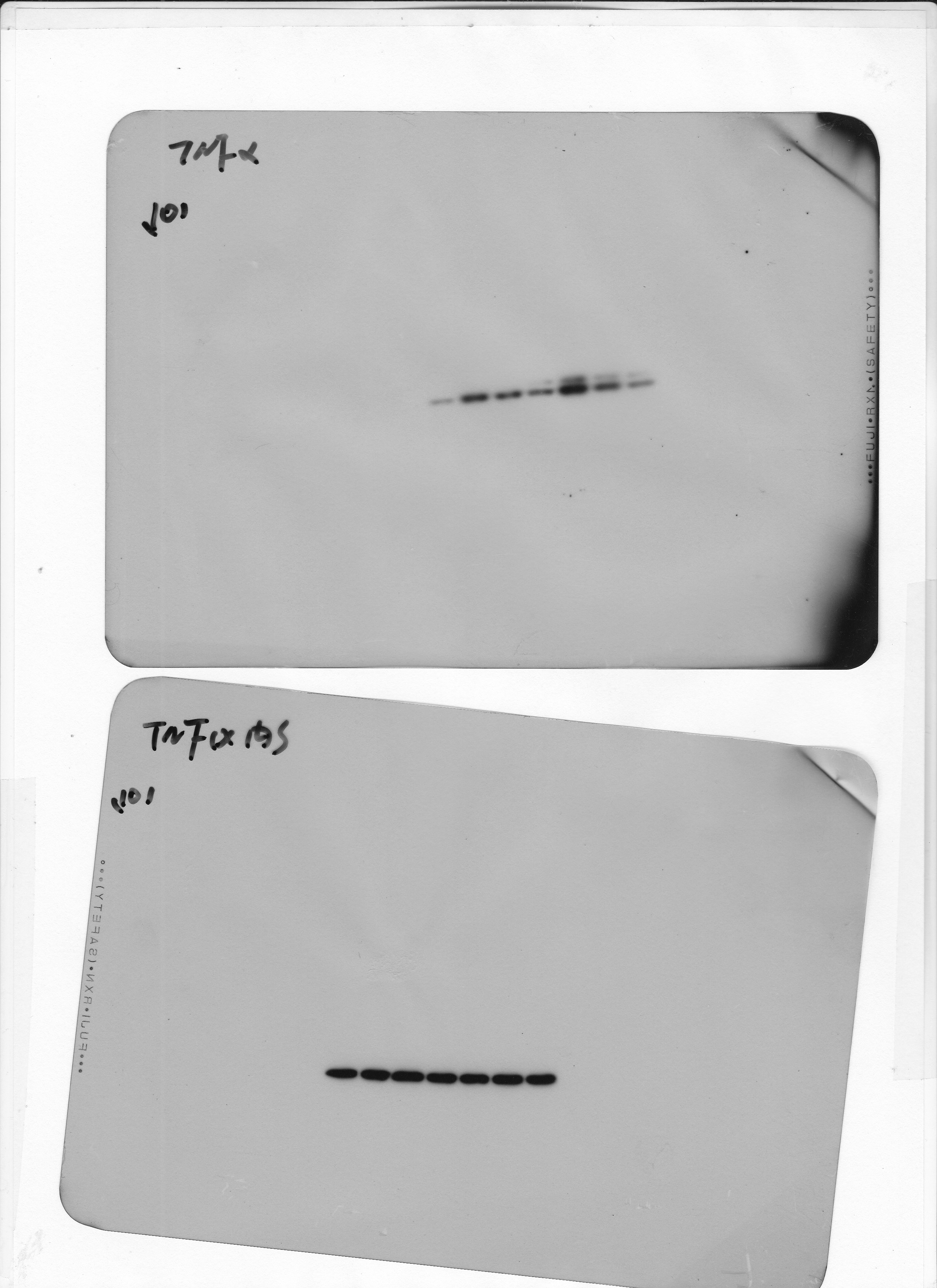

Supplement: Supplementary file 4 [file Data_Sheet_4.ZIP › Western blot figure 2/TNF-α.jpg]
